# Design, synthesis, and evaluation of 4-(3-(3,5-dimethylisoxazol-4-yl)benzyl)phthalazin-1(2H)-one derivatives: potent BRD4 inhibitors with anti-breast cancer activity

**DOI:** 10.3389/fphar.2023.1289003

**Published:** 2023-11-30

**Authors:** Yingpeng Li, Xinhong Chu, Yu Yin, Hongkun Li, Hui Fu, Xinchi Feng, Yanru Deng, Jun Ge

**Affiliations:** ^1^ College of Chinese Materia Medica, Tianjin University of Traditional Chinese Medicine, Tianjin, China; ^2^ College of Integrative Medicine, Tianjin University of Traditional Chinese Medicine, Tianjin, China

**Keywords:** BRD4, PARP1, BRD4 inhibitor, breast cancer, DNA damage

## Abstract

BRD4 inhibitors have demonstrated promising potential in cancer therapy. However, their therapeutic efficacy in breast cancer varies depending on the breast cancer subtype, particularly in the treatment of TNBC. In this study, we designed and synthesized 94 derivatives of 4-(3-(3,5-dimethylisoxazol-4-yl)benzyl)phthalazin-1(2H)-one to evaluate their inhibitory activities against BRD4. Notably, compound DDT26 exhibited the most potent inhibitory effect on BRD4, with an IC_50_ value of 0.237 ± 0.093 μM. DDT26 demonstrated significant anti-proliferative activity against both TNBC cell lines and MCF-7 cells. Intriguingly, the phthalazinone moiety of DDT26 mimicked the PAPR1 substrate, resulting in DDT26 displaying a moderate inhibitory effect on PARP1 with an IC_50_ value of 4.289 ± 1.807 μM. Further, DDT26 was shown to modulate the expression of c-MYC and γ-H2AX, induce DNA damage, inhibit cell migration and colony formation, and arrest the cell cycle at the G1 phase in MCF-7 cells. Our findings present potential lead compounds for the development of potent anti-breast cancer agents targeting BRD4.

## 1 Introduction

In 2022, breast cancer remained a significant health concern, with China and the United States reporting 429,105 and 259,827 new cases, respectively (Giaquinto et al., 2022; Xia et al., 2022). In both nations, breast cancer stands as the predominant cancer among women. Traditional chemotherapeutic agents, including platinum, anthracycline, and paclitaxel, continue to be the primary drugs for adjuvant and neo-adjuvant chemotherapy in breast cancer treatment. However, resistance to these drugs is not uncommon, often leading to a grim prognosis. Advanced patients with systemic recurrence or metastasis frequently require high-dose chemotherapy, which unfortunately can result in severe adverse reactions (Feng et al., 2023; Hussen et al., 2023; Obidiro et al., 2023). Given these challenges, there is a pressing need and significant clinical value in developing novel therapeutic agents with different mechanisms of action as alternatives to traditional chemotherapy.

Bromodomain-containing protein 4 (BRD4) possesses two bromodomains at its N-terminal, enabling it to bind acetylated histones and non-histones, thereby influencing gene transcription, cell cycle regulation, and apoptosis (Cochran et al., 2019). As a transcriptional coactivator, BRD4 has been implicated in the aberrant expression of several oncogenes in breast cancer cells, including c-MYC, p53, FOXM1, and NF-κB (Pérez-Salvia et al., 2017; Pérez-Peña et al., 2018; Yang et al., 2021; Zhou et al., 2022). Furthermore, BRD4 has been shown to upregulate PD-L1, aiding breast cancer cells in evading immune surveillance (Jing et al., 2020). Collectively, these findings position BRD4 as a promising therapeutic target with the potential to revolutionize breast cancer chemotherapy (Sahni and Keri, 2018).

**FIGURE 1:**
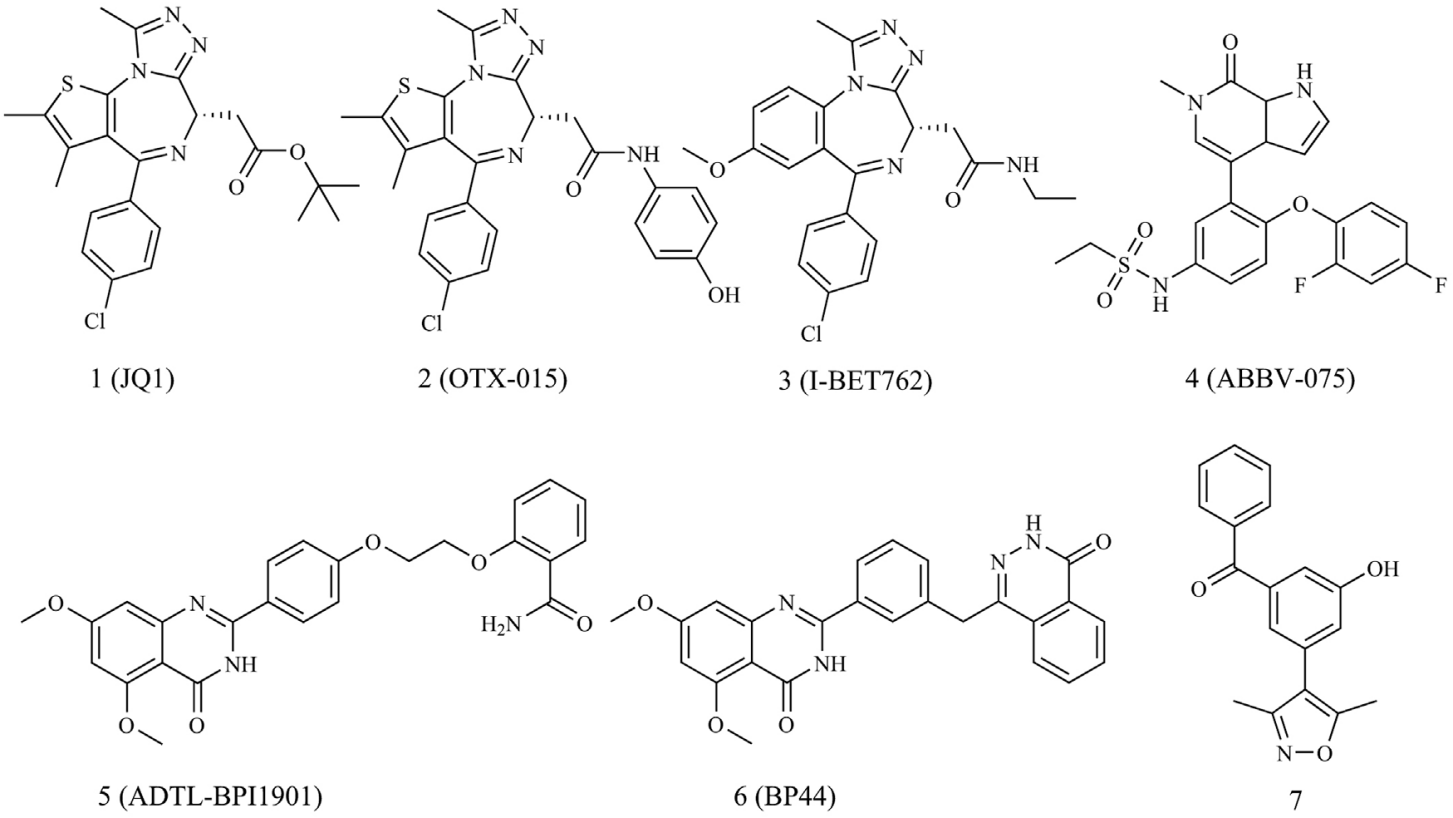
The structures of BRD4 inhibitors.

As illustrated in Figure 1, since the introduction of JQ1 by Mitsubishi in 2010 as the first efficient BRD4 inhibitor, both academic and industrial sectors have dedicated significant efforts to develop an array of BRD4 inhibitors for breast cancer treatment, with a particular focus on triple-negative breast cancer (TNBC) (Gajjela and Zhou, 2023). Regrettably, no BRD4 inhibitors have yet cleared clinical trials to gain approval as cancer chemotherapy agents. A few BRD4 inhibitors are currently in phase I/II clinical trial stages for breast cancer treatment, such as OTX-015, ABBV-075 and I-BET762.

Given the multifaceted roles of BRD4, combinations of BRD4 inhibitors with other drugs have showcased advantages, including potent therapeutic effects, wide safety index and reduced susceptibility to drug resistance in cancer treatments (Jin et al., 2022). Dual-target BRD4 inhibitors have exhibited synergistic effects in breast cancer therapy. For instance, Ouyang’s group reported dual-target inhibitors of PARP1 and BRD4, namely, ADTL-BPI1901 and BP44, which demonstrated excellent antitumor efficacy against TNBC with a high safety profile (Chang et al., 2021; Zhang et al., 2022).

In 2013, Hewings’s group designed and synthesized compound **7**, which was proven to be a modest BRD4 inhibitor with an IC_50_ value of 0.544 μM against the BD1 domain of BRD4 (Hewings et al., 2013). After analyzing the binding mode of compound **7** and BRD4 through molecular docking, we believed that proper structural modification of compound **7** could improve its binding ability with BRD4 to obtain potent BRD4 inhibitors, which was conducive to our next research on anti-breast cancer. The pursuit of BRD4 inhibitors with novel core structures can cover broader chemical space, potentially enhancing drug-like properties and deepening our understanding of structure-activity relationships. In this study, we chose compound **7** as the lead compound to design a series of novel BRD4 inhibitors featuring the 4-(3-(3,5-dimethylisoxazol-4-yl)benzyl)phthalazin-1(2H)-one scaffold. We further investigated their anti-breast cancer efficacies and underlying mechanisms. Our findings offer valuable insights for drug research and development aimed at breast cancer.

## 2 Results and discussion

### 2.1 Design

**FIGURE 2:**
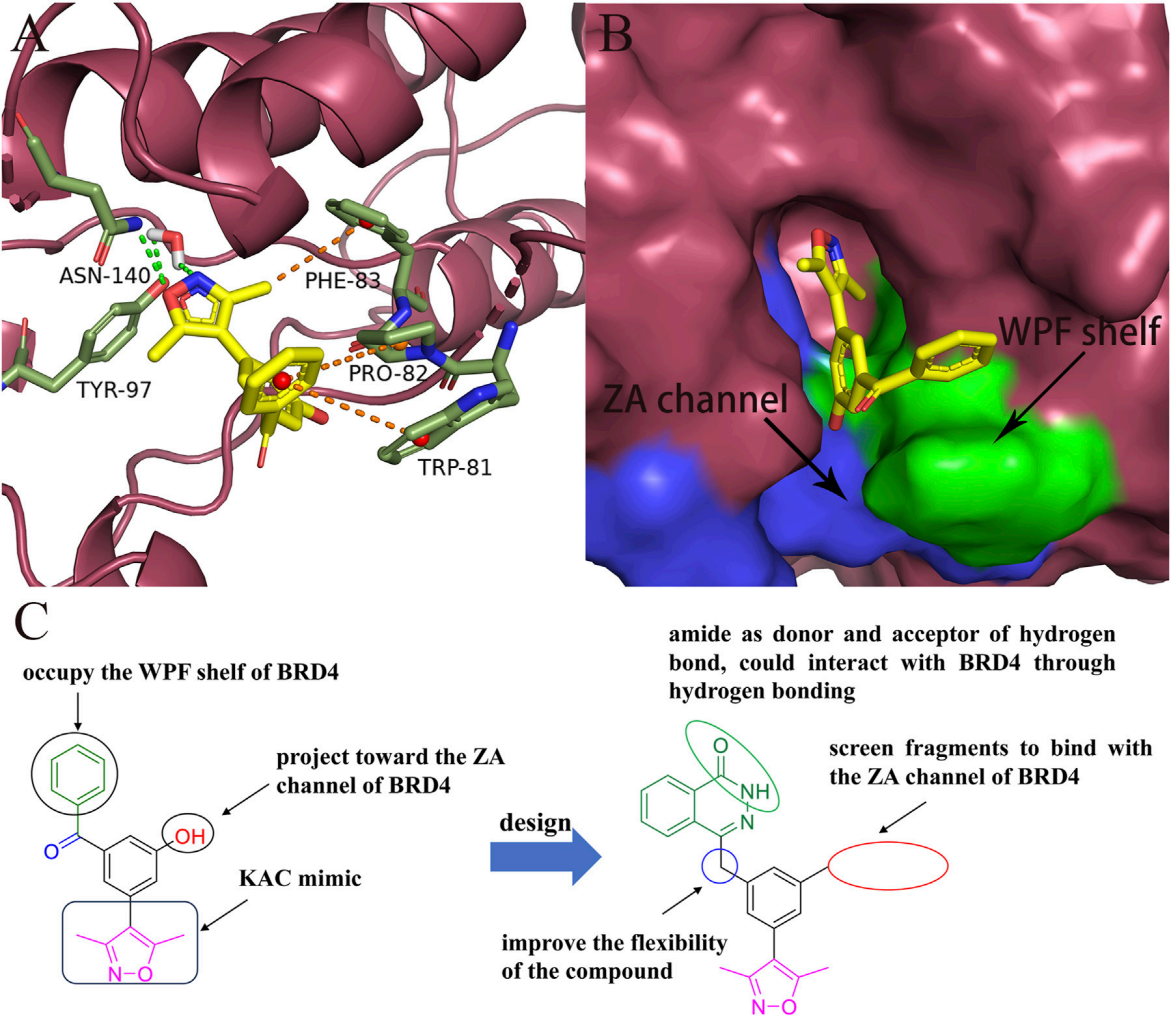
Design of novel BRD4 inhibitors based on lead compound **7**. **(A)** Binding mode of compound **7** in the BD1(PDB ID: 4J0S) domain of BRD4. **(B)** The locations of ZA channel and WPF shelf in BRD4 protein. **(C)** Design strategy of novel BRD4 inhibitors.

Analysis of the binding modes between compound **7** and BRD4 through molecular docking revealed several key interactions. As shown in Figures 2A, B, the 3,5-dimethylisoxazole moiety, serving as a ε-N-lysine acetylation (KAc) motif mimic, established a hydrogen bond with Asn140 and a water-bridging hydrogen bond with Tyr97. The benzoyl group of compound **7** engaged in hydrophobic interactions with the “WPF shelf”, a hydrophobic region in the binding pocket which was composed of Trp81, Pro82, and Phe83. Interestingly, the hydroxyl group of compound **7** did not interact with BRD4 and was oriented toward the ZA channel, a hydrophobic channel region which was composed of Pro82 to Leu91.

As shown in Figure 2C, to enhance the binding affinity of compound **7** to BRD4, we employed several strategies. First, we replaced the benzene ring in compound **7** with phthalazinone. The lactam moiety of phthalazinone, acting as both hydrogen bond donor and acceptor, could form hydrogen bonds with BRD4. Second, we substituted the carbonyl group of compound **7** with a methylene group. This modification was aimed at increasing the compound’s flexibility, thereby facilitating a more favorable conformation for phthalazinone to bind with BRD4. Finally, we replaced the hydroxyl group of compound **7** with various fragments to interact with ZA channel of BRD4.

### 2.2 Biological screening

#### 2.2.1 Enzymatic activities against BRD4

The inhibitory effects of all synthesized compounds on BRD4 were assessed at a concentration of 1 μM, and the structure-activity relationship (SAR) will be discussed in detail. As presented in Table 1, the amide compound **DDT01** displayed no inhibitory activity against BRD4. This observation prompted us to introduce various substituents on the nitrogen atom to enhance the inhibitory potential. When the methyl group in **DDT02** was substituted with cyclopentyl or cyclohexyl, there was a marked improvement in inhibition (as seen in **DDT03** and **DDT04**). Compounds **DDT05**-**DDT07** exhibited weak inhibitory effects, suggesting that the aromatic structure might be detrimental to potency. Modifying the methylene group in the cyclohexane ring of **DDT04** with either oxygen or sulfone led to decreased inhibitory effects (as observed in **DDT08** and **DDT09**). Introducing hydroxyl groups into the cyclohexane ring of **DDT04** yielded two optical isomers, **DDT10** and **DDT11**. Notably, **DDT10** demonstrated a higher inhibitory rate than **DDT11**, indicating the stereochemistry of the chiral carbon influenced the compound’s potency. Interestingly, both **DDT13** and **DDT14**, derived from the reduction of the carbonyl group in **DDT12**, exhibited enhanced inhibitory effects on BRD4. However, the stereochemistry of **DDT13** and **DDT14** did not seem to influence their inhibitory potential. Lastly, most compounds synthesized by substituting cyclohexylamine with either piperidine or piperazine lost their inhibitory effects on BRD4, with the exceptions being **DDT15** and **DDT16**.

**TABLE 1:** Inhibition rates of compounds DDT01-DDT21 against BRD4.

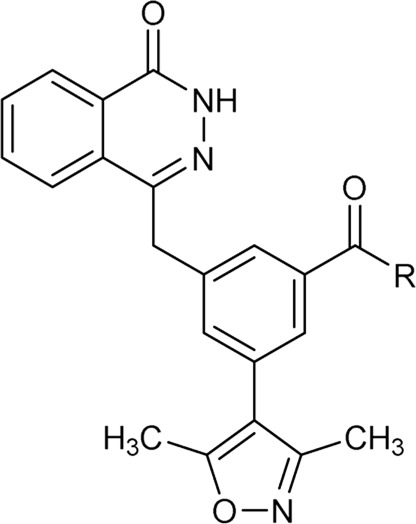

Compound	R	BRD4 inhibition rate (1 μmol/L, %)
DDT01	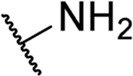	15
DDT02	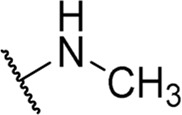	5
DDT03	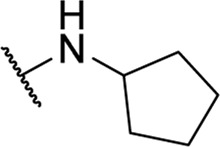	67
DDT04	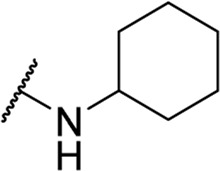	56
DDT05	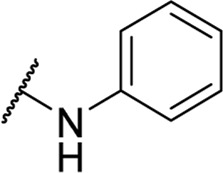	35
DDT06	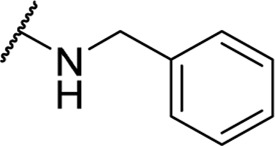	4
DDT07	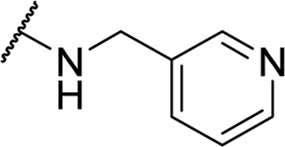	8
DDT08	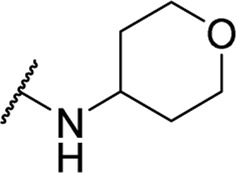	11
DDT09	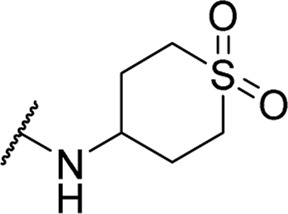	4
DDT10	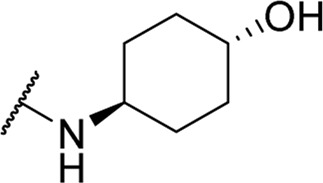	36
DDT11	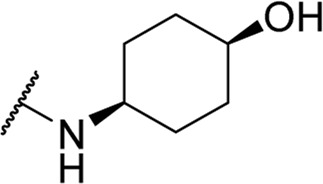	54
DDT12	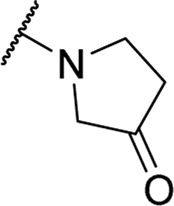	38
DDT13	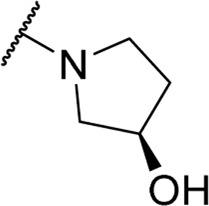	71
DDT14	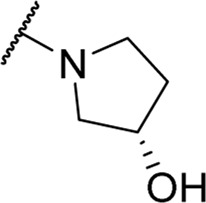	74
DDT15	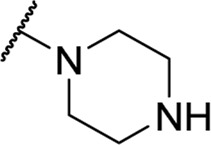	59
DDT16	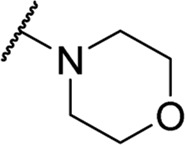	71
DDT17	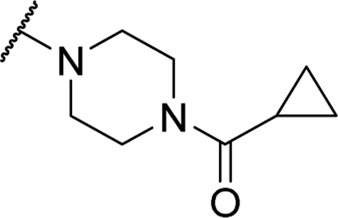	16
DDT18	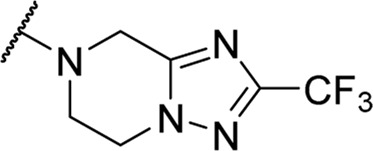	37
DDT19	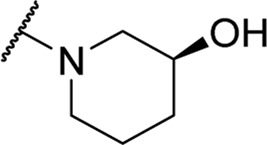	11
DDT20	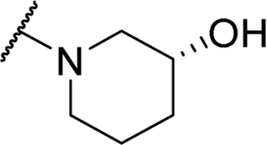	4
DDT21	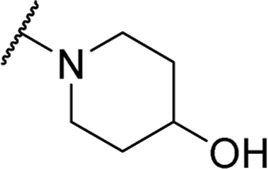	19

As depicted in Table 2, compounds synthesized by reversing the amide orientation in **DDT05** generally displayed high inhibitory rates against BRD4. The positions of the substituents had a more pronounced effect on inhibitory activity than their electronic properties. Specifically, ortho-substituted derivatives exhibited the most potent inhibitory activity. Upon removal of the carbonyl group’s oxygen in **DDT22**, a series of benzyl-substituted amine derivatives were synthesized. However, most of these derivatives showed weak inhibitory effects against BRD4, with the exceptions being **DDT47**-**DDT49**.

**TABLE 2:** Inhibition rates of compounds DDT22-DDT49 against BRD4.

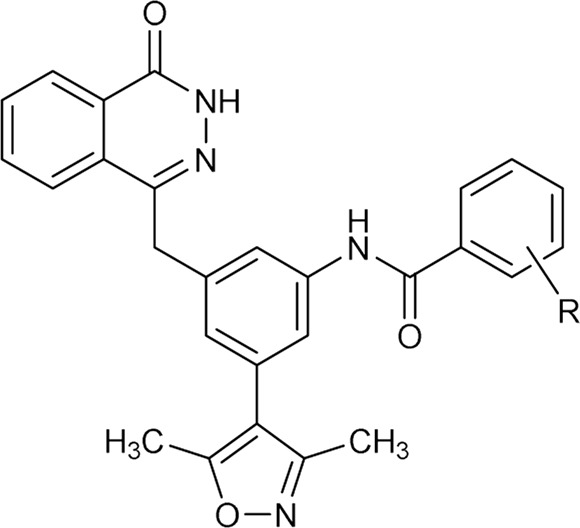 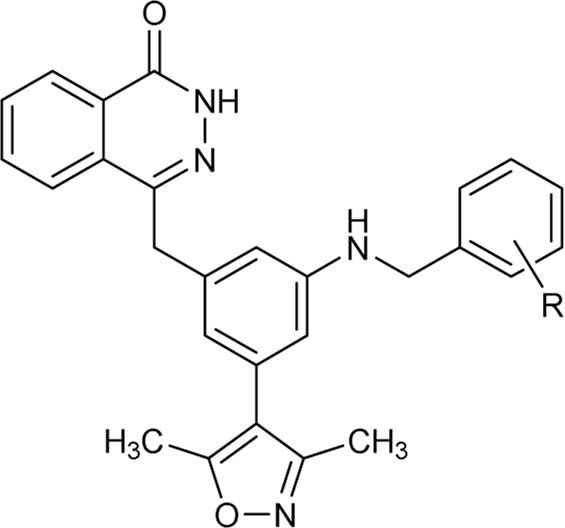
DDT22-DDT32 DDT33-DDT49

Compound	R	BRD4 inhibition rate (1 μmol/L, %)
DDT22	H	64
DDT23	2-CH_3_	97
DDT24	3-CH_3_	91
DDT25	4-CH_3_	76
DDT26	2-CF_3_	85
DDT27	3-CF_3_	60
DDT28	4-CF_3_	54
DDT29	3-NO_2_	65
DDT30	4-NO_2_	74
DDT31	3-NH_2_	71
DDT32	4-NH_2_	54
DDT33	H	3
DDT34	3-F	3
DDT35	4-F	3
DDT36	3-NO_2_	3
DDT37	4-NO_2_	8
DDT38	3-Cl	8
DDT39	4-Cl	16
DDT40	3-Br	3
DDT41	4-Br	17
DDT42	2-F,4-Br	3
DDT43	3-Br, 4-f	4
DDT44	3-CH_3_	3
DDT45	4-CH_3_	7
DDT46	4-CF_3_	39
DDT47	3-acetamino	78
DDT48	4-acetamino	96
DDT49	3-NH_2_	72

Table 3 presents a series of sulfonamide derivatives and their inhibitory effects on BRD4. Excluding **DDT50**, alkyl-substituted sulfonamides did not exhibit inhibitory activity against BRD4. A subset of benzene sulfonamide compounds was then evaluated. While the 3-methyl and 4-methyl substituted compounds demonstrated potent inhibitory effects, the 3,4-dimethyl and 3,5-dimethyl derivatives showed reduced activity (refer to **DDT58**-**DDT61**). Introducing larger substituents in place of the 4-methyl group led to decreased inhibitory effects (see **DDT62**-**DTT65**). **DDT66**, derived by substituting the benzene ring of **DDT59** with pyridine, showed a marked reduction in inhibitory activity. Among the methoxy-substituted derivatives, the 4-methoxy compound **DDT69** exhibited the highest inhibitory rate. Compounds **DDT76**-**DTT78** displayed low inhibitory rates, suggesting that inserting a methylene group between the benzene and sulfonyl groups hindered BRD4 binding. Compounds **DDT80**-**DDT83** and **DDT94**, with electron-withdrawing groups, showed weak inhibitory activities. Among halogenated derivatives, only the ortho and para chlorine-substituted compounds demonstrated potent inhibitory effects (refer to **DDT87** and **DDT89**). These findings suggested that, for benzene sulfonamide derivatives, electron-donating groups and para substituents enhanced the inhibitory potency against BRD4.

**TABLE 3:** Inhibition rates of compounds **DDT50**-**DDT94** against BRD4.

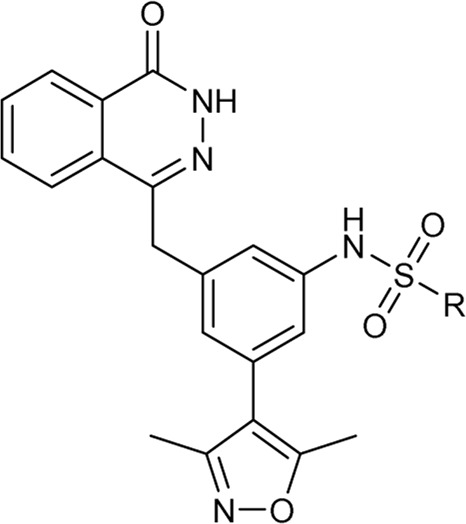 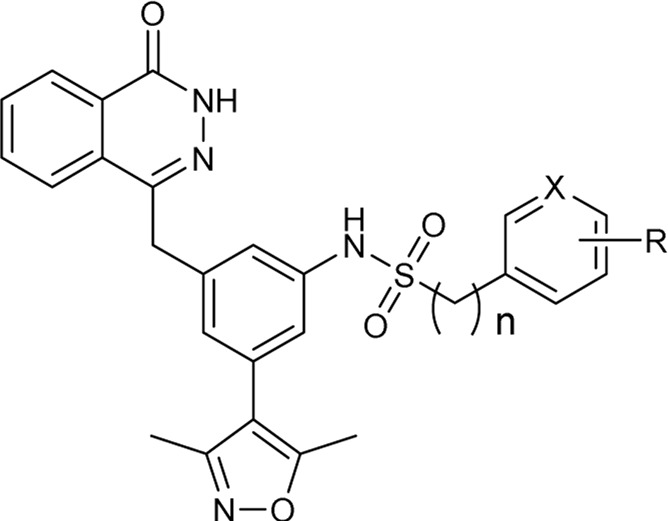

Compound	n	X	R	BRD4 inhibition rate (1 μmol/L, %)
DDT50	—	—	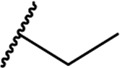	69
DDT51	—	—	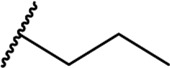	4
DDT52	—	—	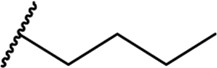	6
DDT53	—	—	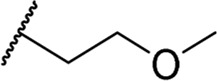	9
DDT54	—	—	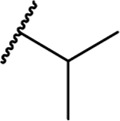	3
DDT55	—	—	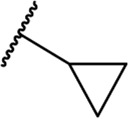	32
DDT56	—	—	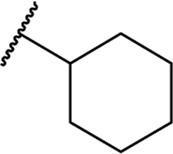	24
DDT57	0	CH	H	36
DDT58	0	CH	3-CH_3_	71
DDT59	0	CH	4-CH_3_	75
DDT60	0	CH	3,5-dimethyl	28
DDT61	0	CH	3,4-dimethyl	49
DDT62	0	CH	4-CH_2_CH_3_	67
DDT63	0	CH	4-n-propyl	51
DDT64	0	CH	4-isopropyl	69
DDT65	0	CH	4-tert-butyl	44
DDT66	0	N	4-CH_3_	8
DDT67	0	CH	2-OCH_3_	24
DDT68	0	CH	3-OCH_3_	59
DDT69	0	CH	4-OCH_3_	70
DDT70	0	CH	3,4-dimethoxy	32
DDT71	0	CH	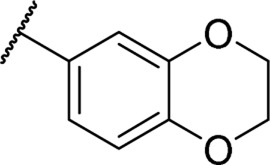	67
DDT72	0	CH	3-acetamino	4
DDT73	0	CH	4-acetamino	38
DDT74	0	CH	4-benzyloxy	34
DDT75	0	CH	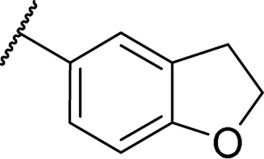	42
DDT76	1	CH	H	50
DDT77	1	CH	3-CH_3_	38
DDT78	1	CH	4-CH_3_	55
DDT79	0	CH	4-OH	7
DDT80	0	CH	2-CF_3_	15
DDT81	0	CH	3-CF_3_	20
DDT82	0	CH	4-CF_3_	24
DDT83	0	CH	4-NO_2_	28
DDT84	0	CH	2-F	14
DDT85	0	CH	3-F	5
DDT86	0	CH	4-F	7
DDT87	0	CH	2-Cl	72
DDT88	0	CH	3-Cl	17
DDT89	0	CH	4-Cl	72
DDT90	0	CH	2,4-dichloroyl	4
DDT91	0	CH	2-Br	8
DDT92	0	CH	3-Br	4
DDT93	0	CH	4-Br	26
DDT94	0	CH	3-methanesulfonyl	5

We determined the IC_50_ values of several synthesized compounds for their inhibitory effects on BRD4. As presented in Table 4, among the compounds tested, **DDT26** exhibited the most potent inhibitory activity against BRD4, with an IC_50_ value of 0.237 ± 0.093 μM. In subsequent investigations, we observed that **DDT26** could induce DNA damage in MCF-7 cells. This observation led us to hypothesize that **DDT26** might interact with targets other than BRD4, potentially interfering with cellular DNA damage repair mechanisms. Notably, the 4-benzylphthalazin-1(2H)-one structure in **DDT26** bore resemblance to Olaparib, a known PARP1 inhibitor. Supporting this hypothesis, biochemical assays revealed that **DDT26** indeed possessed a modest inhibitory effect on PARP1, with an IC_50_ value of 4.289 ± 1.807 μM. Interestingly, both **DDT14** and **DDT49** demonstrated stronger inhibitory activities against PARP1 than **DDT26** when tested at a concentration of 0.5 μM.

**TABLE 4:** IC_50_ values of target compounds against BRD4 and PARP1.

Compound	PARP1 IC_50_ (μM) or inhibition rate (0.5 μmol/L, %)	BRD4 IC_50_ (μM)
Olaparib	0.002 ± 0.001	—
JQ1	—	0.124 ± 0.042
DDT14	53%	1.101 ± 0.450
DDT23	—	3.604 ± 1.453
DDT24	—	1.879 ± 0.600
DDT26	4.289 ± 1.807	0.237 ± 0.093
DDT48	—	0.460 ± 0.170
DDT49	50%	0.585 ± 0.225
DDT59	8%	0.909 ± 0.396

—Not applicable.

#### 2.2.2 Cytotoxicity assay

Several compounds exhibiting strong inhibitory effects against BRD4 were evaluated for their anti-proliferative activities on three breast cancer cell lines, using JQ1 and Olaparib as positive controls. As detailed in Table 5, JQ1 demonstrated potent anti-proliferative effects on MDA-MB-231 and MDA-MB-468 cells. However, MCF-7 cells appeared to be resistant to JQ1. The anti-proliferative effects of Olaparib on three breast cancer cell lines were modest. Notably, among the synthesized compounds tested, **DDT26** displayed robust inhibitory effects on all three breast cancer cell lines, and its inhibitory potency on MCF-7 cells surpassed that of JQ1.

**TABLE 5:** *In vitro* anti-proliferative activity of target compounds against breast cancer cell lines.

Compound	Cytotoxicity IC_50_ (μM)		
	MDA-MB-231	MDA-MB-468	MCF-7
JQ1	4.83	3.24	18.37
Olaparib	8.17	8.09	8.70
DDT14	8.29	14.41	>20
DDT23	8.83	10.58	18.71
DDT24	8.23	9.09	12.84
DDT25	11.56	6.50	12.47
DDT26	7.55	3.96	4.90
DDT47	17.13	9.39	11.65
DDT48	8.77	9.09	>20
DDT59	7.44	10.96	>20

#### 2.2.3 Molecular docking study

**FIGURE 3:**
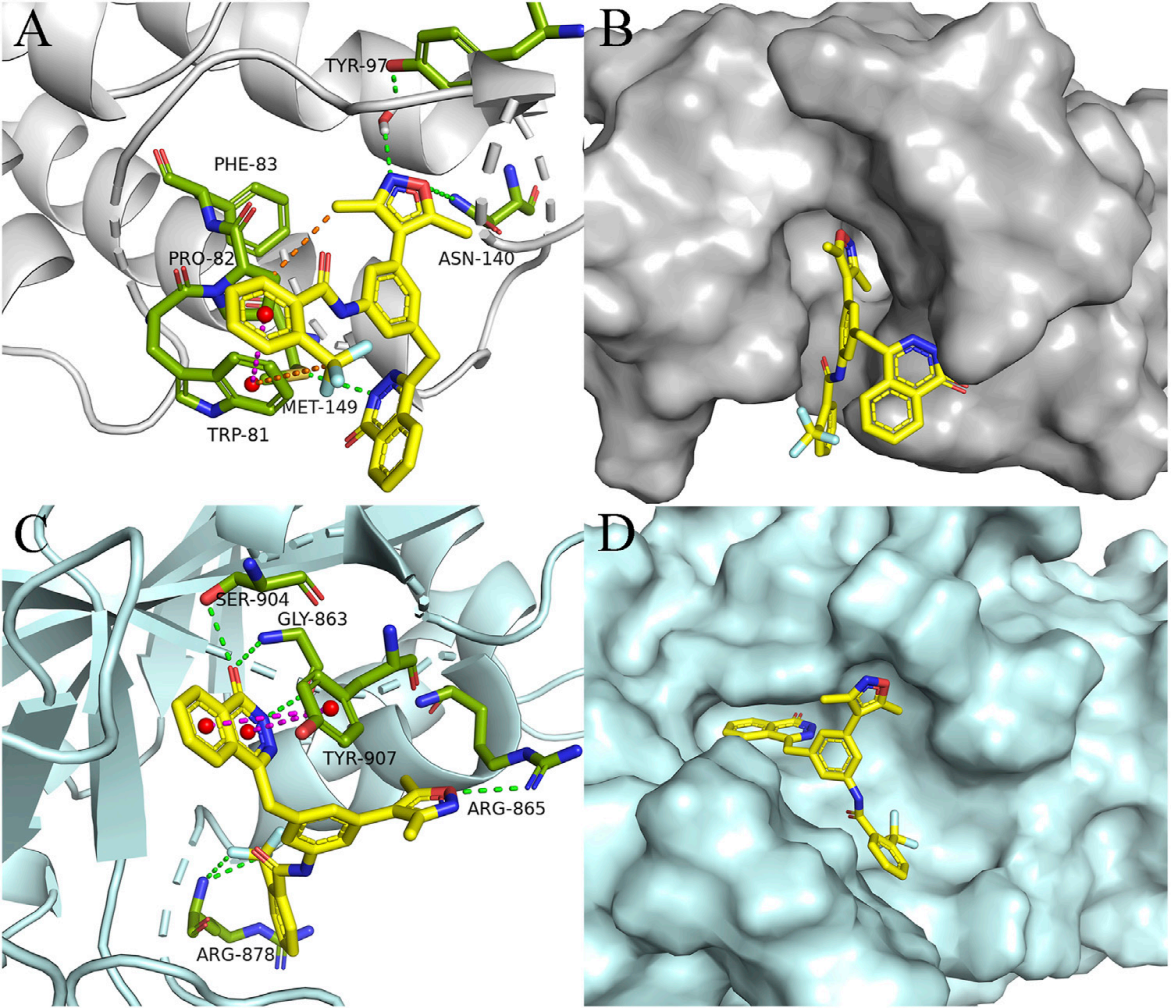
Binding mode analysis of **DDT26**. **(A)** Binding mode of **DDT26** in the BD1(PDB ID: 4J0S) domain of BRD4. **(B)** Conformation of **DDT26** at the binding site of BRD4. **(C)** Binding mode of **DDT26** with PARP1 (PDB ID: 5DS3). **(D)** Conformation of **DDT26** at the binding site of PARP1.

Given the impressive inhibitory performance of **DDT26** against both BRD4 and cell proliferation, we undertook a comprehensive analysis of this compound. Through molecular docking, we examined the binding modes of some compounds with BRD4 in Table 4. As shown in Figures 3A, B; Supplementary Table S1, the binding mode of **DDT26** with BRD4 was quite similar to that of compound 7. The 3,5-dimethylisoxazole moiety of **DDT26** snugly fit into the acetylated lysine binding pocket. The isoxazole’s oxygen formed a hydrogen bond with Asn140, its nitrogen engaged in a water-mediated hydrogen bond with Tyr97, and its methyl group participated in hydrophobic interactions with Pro82. Consistent with our design strategy, the 2-trifluoromethyl benzamide extended into the ZA channel where its benzene and trifluoromethyl groups established T-shaped pi-pi stacking and hydrophobic interactions with Trp81, respectively. Intriguingly, the phthalazinone moiety of **DDT26** did not align with the WPF shelf but formed a hydrogen bond with Met149.

Biochemical experiment showed that **DDT26** was a PARP1 inhibitor, which prompted us to analyze the binding mode of **DDT26** with PARP1. As shown in Figures 3C, D; Supplementary Table S2, the binding mode of **DDT26** with PARP1 closely resembled that of Olaparib. The phthalazinone moiety of **DDT26** formed hydrogen bonds with Gly863 and Ser904. Its aromatic ring engaged in face-to-face pi-pi stacking interaction with Tyr907. The isoxazole’s oxygen formed a hydrogen bond with Arg865, and the fluorine of trifluoromethyl group established a hydrogen bond with Arg878.

#### 2.2.4 DDT26 modulated proteins expression in MCF-7 cells

**FIGURE 4:**
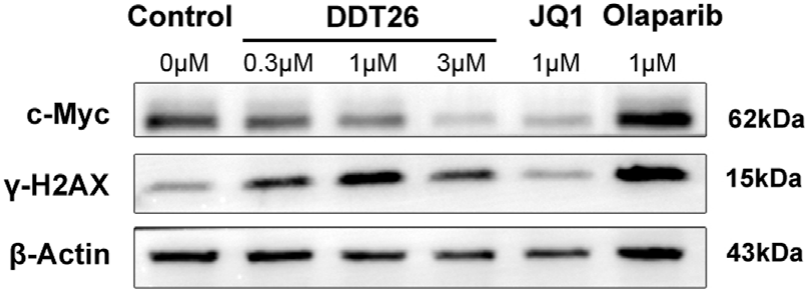
The effects of JQ1, Olaparib, and **DDT26** on the expression of c-MYC and γ-H2AX analyzed by Western blot.

c-Myc, a member of the Myc family of oncogenes, is known to promote cancer cell proliferation, metabolism, and protein synthesis. BRD4, functioning as a transcriptional regulator, plays a role in modulating the expression of c-MYC. As illustrated in Figure 4, Western blot analysis revealed that **DDT26** could downregulate the expression of c-MYC in MCF-7 cells in a concentration-dependent manner. γ-H2AX is recognized as a marker for DNA double-strand damage. Western blot results also indicated that **DDT26** induced the accumulation of γ-H2AX in MCF-7 cells in a concentration-dependent fashion.

#### 2.2.5 DDT26 induced G1 phase cell cycle arrest in MCF-7 cells

**FIGURE 5:**
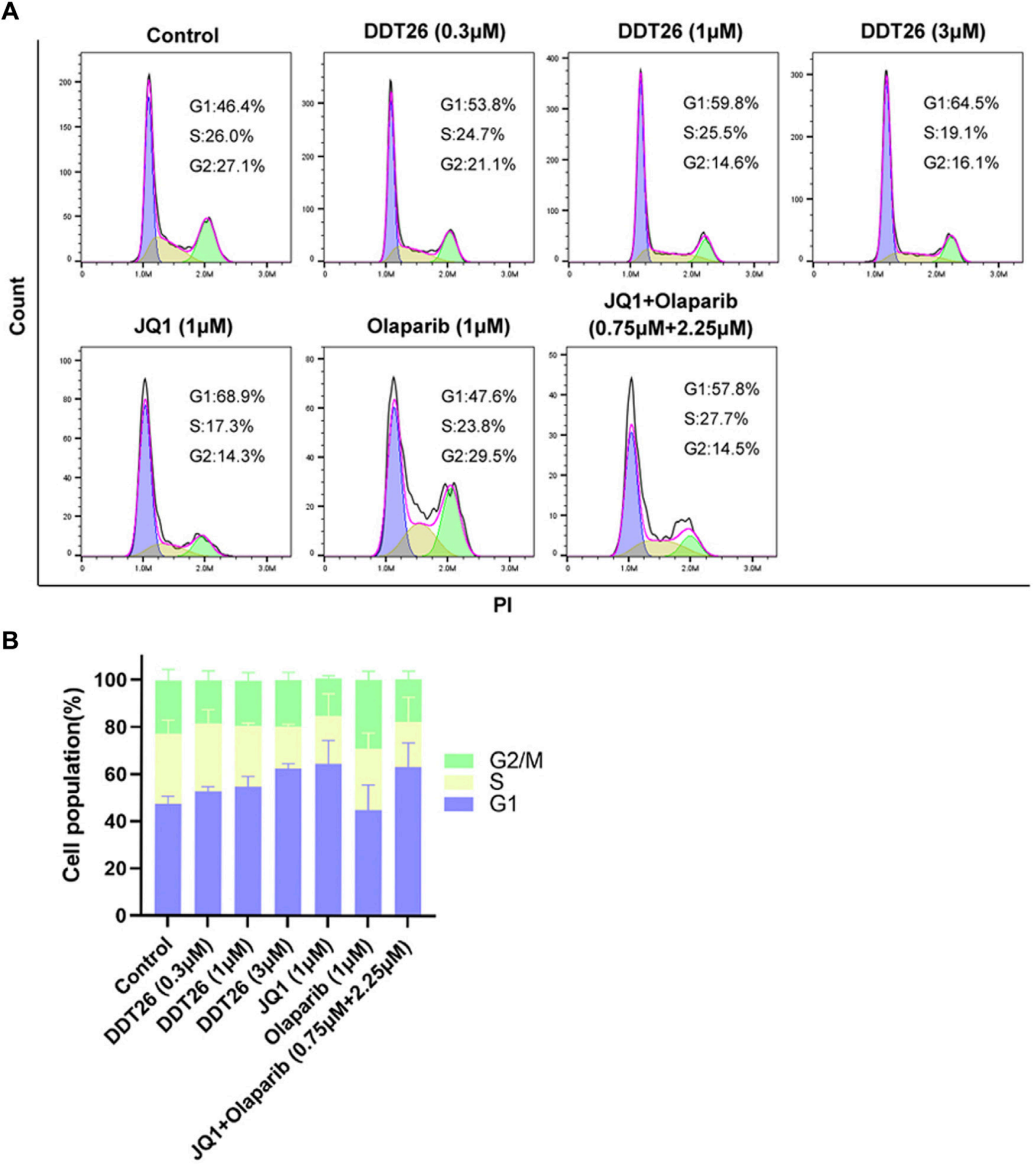
**DDT26** induced cell cycle of MCF-7 arrest in the G1 phase. **(A)** Effects of JQ1(1 μM), Olaparib (1 μM), JQ1+Olaparib (0.75 μM + 2.25 μM) and **DDT26** (0.3 μM, 1 μM, and 3 μM) on cell cycle progression in MCF-7 cells. **(B)** The percentage of cells in each population.

MCF-7 cells treated with compound **DDT26** at concentrations of 0.3, 1, and 3 μΜ for 48 h displayed distinct cell cycle effects. As depicted in Figure 5, the proportions of cells in the G1 phase for the **DDT26**-treated groups were 53.8%, 59.8%, and 64.5%, respectively. This indicated that **DDT26** induced a dose-dependent G1 phase cell cycle arrest in MCF-7 cells. At a concentration of 1 μΜ, the JQ1-treated group exhibited a more pronounced cell cycle arrest compared to the **DDT26** group. In contrast, the Olaparib-treated group did not influence the MCF-7 cell cycle. The combination treatment group displayed a slightly reduced cell cycle arrest capability compared to the JQ1-treated group, suggesting that concurrent inhibition of BRD4 and PARP1 did not synergistically enhance cell cycle arrest.

#### 2.2.6 DDT26 suppressed colony formation in MCF-7 cells

**FIGURE 6:**
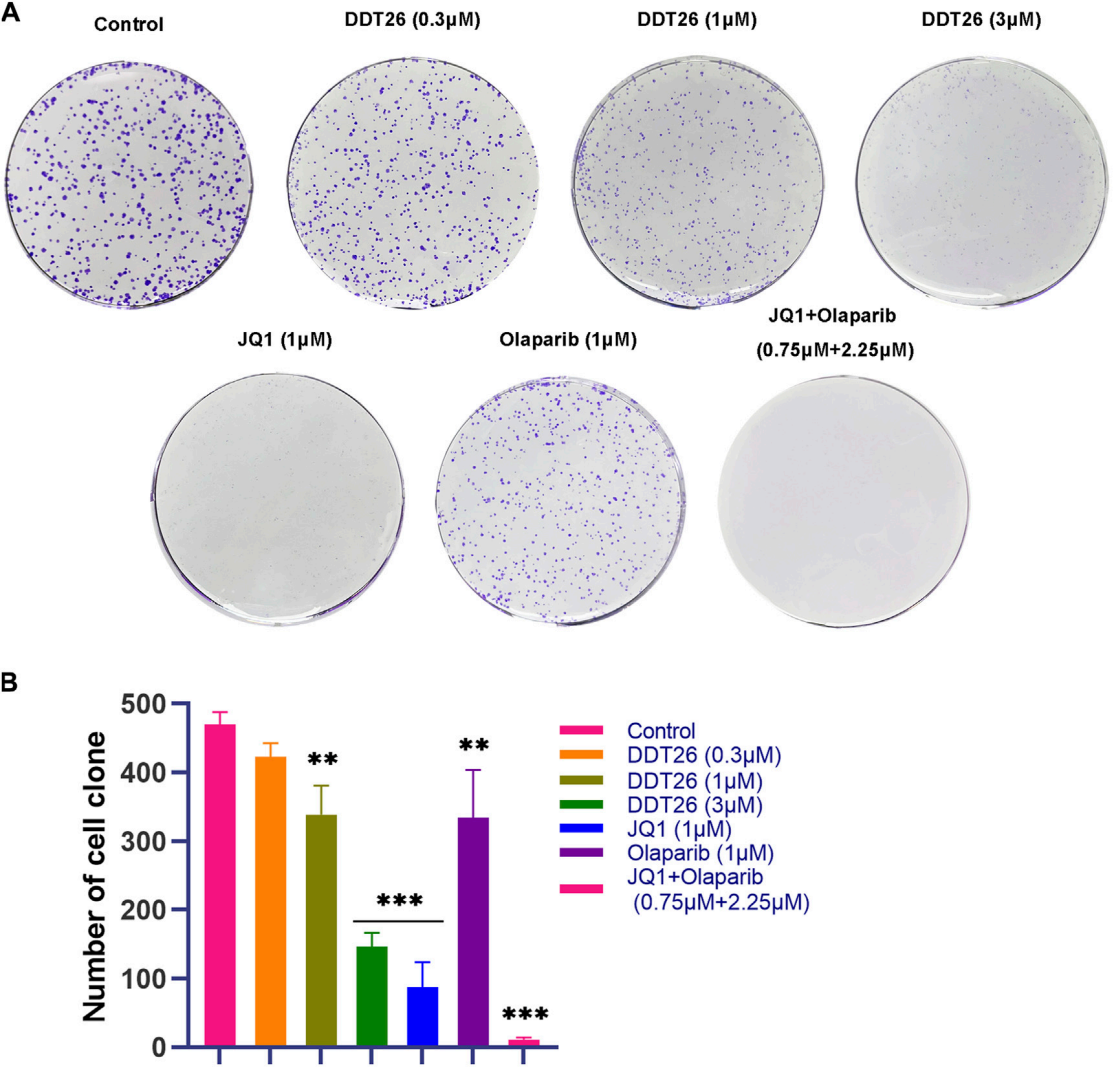
**DDT26** inhibited colony formation in MCF-7 Cells. **(A)** Colony formation assay of MCF-7 cells treated with JQ1(1 μM), Olaparib (1 μM), JQ1 + Olaparib (0.75 μM + 2.25 μM) and **DDT26** (0.3 μM, 1 μM, and 3 μM), respectively. **(B)** The number of cell clone in each population.

Colony formation assay is an effective method to directly observe the anti-proliferation effect of compounds on cancer cells. As shown in Figure 6, at a concentration of 1 μM, JQ1’s ability to suppress colony formation surpassed that of both **DDT26** and Olaparib. **DDT26** inhibited colony formation in MCF-7 cells in a dose-dependent manner. The results of colony formation assay showed that **DDT26** could effectively inhibit the proliferation of MCF-7 cells at concentrations above 3 μM.

#### 2.2.7 DDT26 inhibited MCF-7 cells migration

**FIGURE 7:**
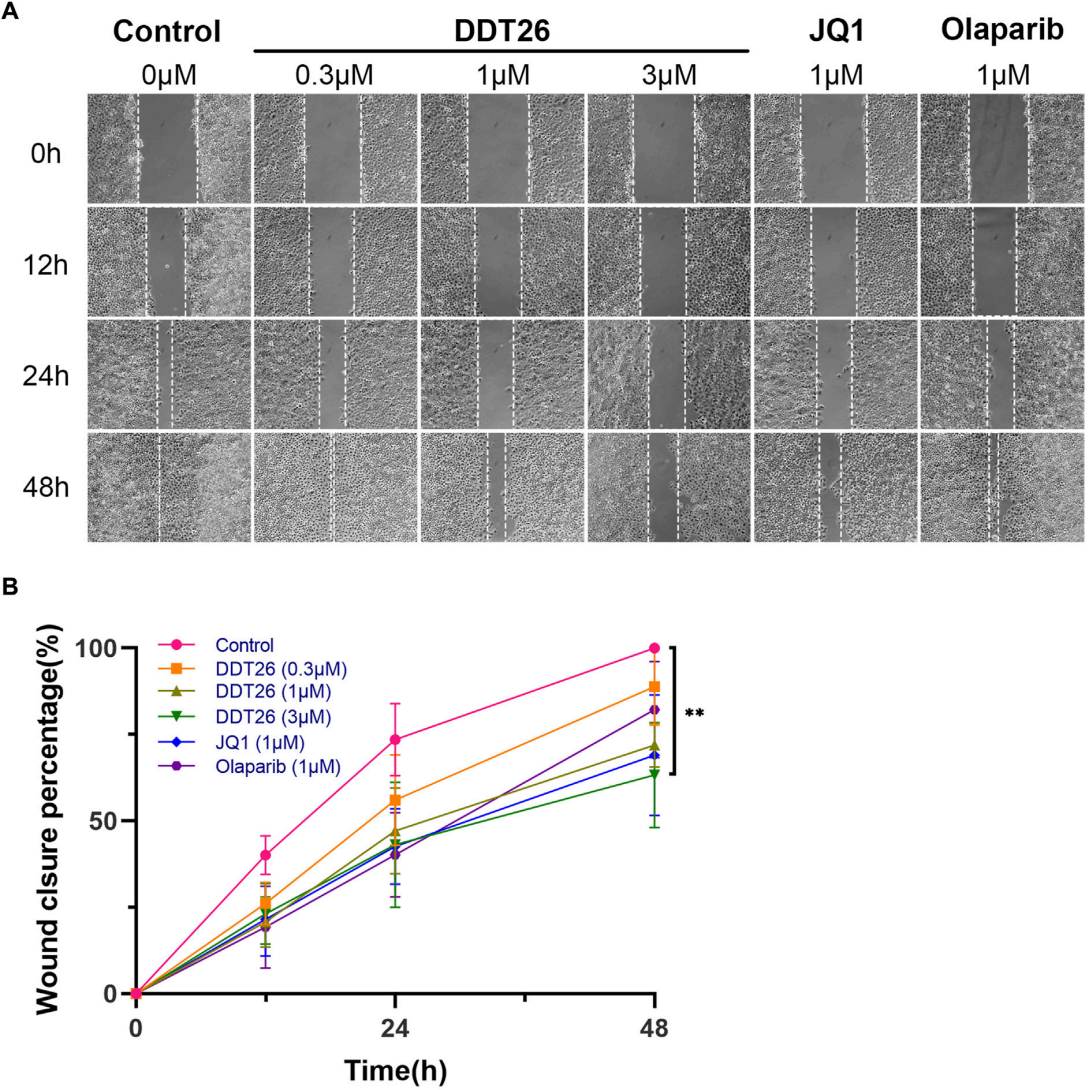
DDT26 inhibits the migration of MCF-7 cells in a concentration dependent manner. **(A)** Effects of **DDT26**, JQ1 and Olaparib on the migration of MCF-7 cells. **(B)** Wound closure percentage of **DDT26** (0.3 μM, 1 μM, 3 μM), JQ1 (1 μM) and Olaparib (1 μM) in MCF-7 cells.

Enhanced migratory capability can lead to the metastasis of cancer cells from the primary site to other tissues via the blood or lymphatic system, culminating in the formation of new tumor lesions. This is a critical indicator of the progression of many cancers. We evaluated the effects of **DDT26**, JQ1, and Olaparib on the migratory capability of MCF-7 cells using a wound scratch assay. As depicted in Figure 7, the control group demonstrated that MCF-7 cells completely filled the wound area 48 h post-scratching. At a concentration of 1 μM, JQ1 exhibited the most potent inhibitory effect on MCF-7 cell migration. Compared to JQ1, **DDT26**’s inhibitory effect on MCF-7 cells migration was slightly less pronounced, while Olaparib showed no inhibitory activity. The migration-inhibitory effect of **DDT26** on MCF-7 cells was further amplified at a concentration of 3 μM, underscoring **DDT26’s** concentration-dependent inhibitory action.

#### 2.2.8 DDT26 induced DNA damage of MCF-7 cells

**FIGURE 8:**
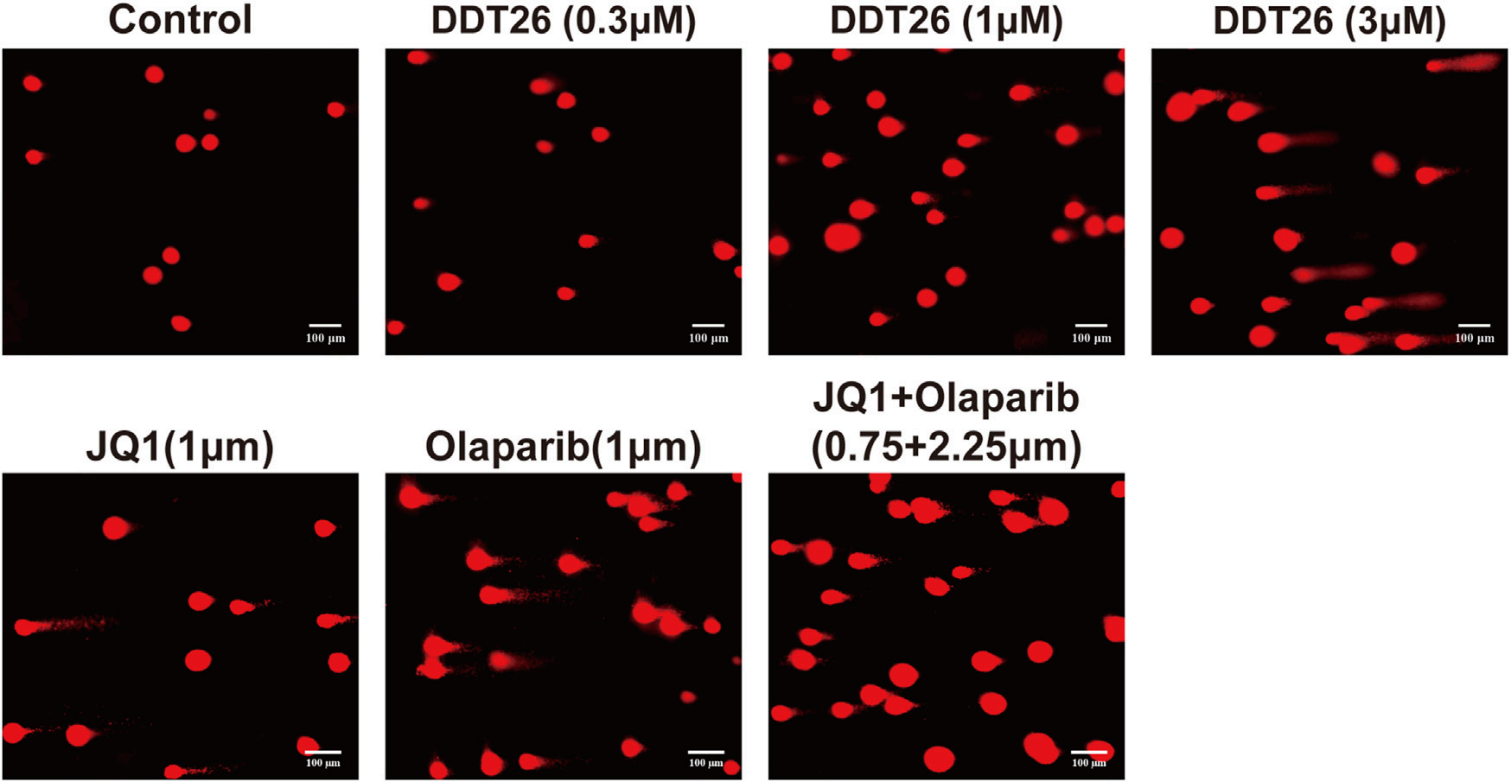
Effects of JQ1, Olaparib, JQ1+ Olaparib, **DDT26** on the DNA damage of MCF-7 cells.

PARP1 acts as a sensor for DNA single-strand damage, while BRD4 plays a role in regulating the transcription of homologous recombination related proteins. Given the pivotal roles of both PARP1 and BRD4 in DNA damage repair, we employed a comet assay to investigate **DDT26**’s potential to induce DNA damage in MCF-7 cells. As illustrated in Figure 8, **DDT26** induced DNA damage in MCF-7 cells in a dose-dependent manner. At a concentration of 3 μM, **DDT26** inflicted more pronounced DNA damage than the combination treatment. These findings suggested that **DDT26**’s ability to inhibit MCF-7 cell proliferation was intrinsically linked to its DNA damage-inducing properties.

## 3 Conclusion

Breast cancer imposes a significant psychological and economic burden on patients. Consequently, the quest for effective targeted therapies for breast cancer remains at the forefront of drug research and development. Given the intricate classifications of breast cancer, not all types are responsive to BRD4 inhibitors. This underscores the need to identify novel BRD4 inhibitors with distinct mechanisms of action to broaden their therapeutic applicability in breast cancer treatment (Sahni and Keri, 2018).

In this study, we designed and synthesized a series of novel 4-(3-(3,5-dimethylisoxazol-4-yl)benzyl)phthalazin-1(2H)-one derivatives as potential BRD4 inhibitors. Among them, **DDT26** emerged as the most potent, exhibiting robust inhibitory activity against BRD4 at sub-micromolar concentrations. Furthermore, biochemical analyses revealed that **DDT26** also mildly inhibited PARP1, a critical target for treating breast cancers with BRCA1/2 mutations. Molecular docking studies highlighted the phthalazinone moiety in **DDT26** as pivotal, facilitating interactions with Met149 of BRD4 and Gly863, Ser904, and Tyr907 of PARP1.

Interestingly, **DDT26** demonstrated a more pronounced inhibitory effect on MCF-7 cells proliferation compared to JQ1, even though its direct inhibitory activity against BRD4 was somewhat inferior. We postulate that **DDT26**’s enhanced cytotoxicity in MCF-7 cells may stem from its dual-targeting capability, simultaneously inhibiting both BRD4 and PARP1 (Sun et al., 2018; Mio et al., 2019). Subsequent investigations confirmed **DDT26**’s ability to downregulate c-MYC expression dose-dependently, induce γ-H2AX accumulation, inhibit both migration and colony formation, and arrest the cell cycle in the G1 phase in MCF-7 cells. Collectively, these findings position **DDT26** as a promising lead compound in the development of novel anti-breast cancer therapeutics.

## 4 Methods section

### 4.1 Chemistry

**FIGURE 9:**
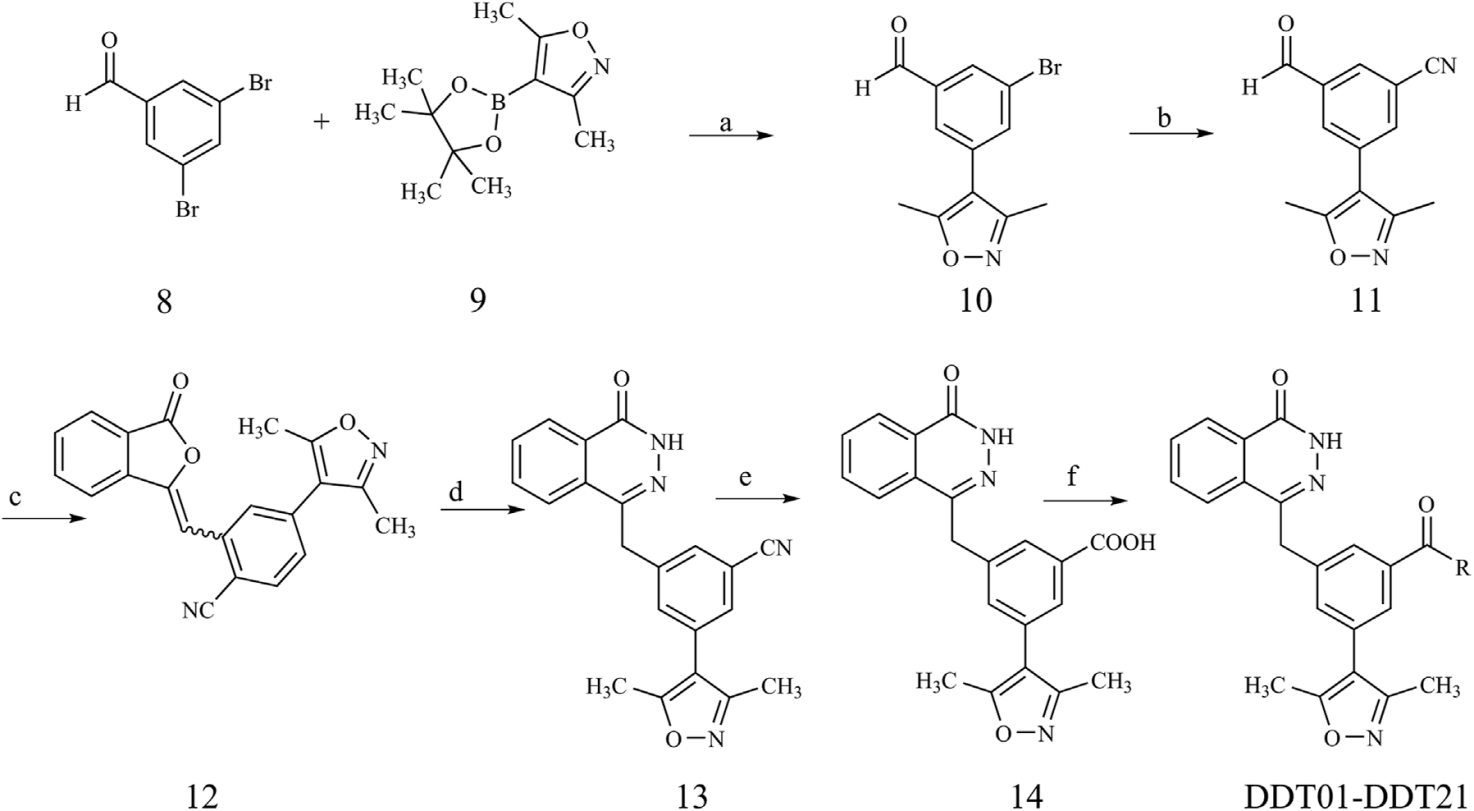
Synthetic route of compound **DDT01**-**DDT21**. Reagents and conditions: (a) Pd(PPh_3_)_4_, K_2_CO_3_, N_2_, dioxane/H_2_O (3:1), 90°C; (b) Zn(CN)_2_, Pd(PPh_3_)_4_, DMF, N_2_, 80°C; (c) Dimethyl (3-oxo-1,3-dihydroisobenzofuran-1-yl)phosphonate, Et_3_N, THF, rt; (d) NH_2_NH_2_·H_2_O, EtOH, reflux; (e) (i) NaOH, MeOH/H_2_O (1:1), 100°C (ii) HCl (2 N), rt; (f) EDCI, HOBT, DIPEA, DMF, rt.

The synthetic pathways for the target compounds are depicted in Figure 9. Starting with commercially available 3,5-dibromobenzaldehyde (compound **8**), it was coupled with boric ester (compound **9**) to yield intermediate **10** through Suzuki reaction. This was followed by a palladium-catalyzed cyanation of intermediate **10** to produce intermediate **11**. A subsequent Horner−Wadsworth−Emmons reaction between intermediate **11** and phosphonate resulted in olefin **12**. Refluxing olefin **12** with hydrazine hydrate in ethanol led to the formation of the phthalazinone in intermediate **13**. The pivotal intermediate **14** was synthesized by hydrolyzing the nitrile group of intermediate **13** under alkaline conditions. Ultimately, condensing intermediate **14** with the appropriate amines under standard conditions yielded amides **DDT01**-**DDT21**.

**FIGURE 10:**
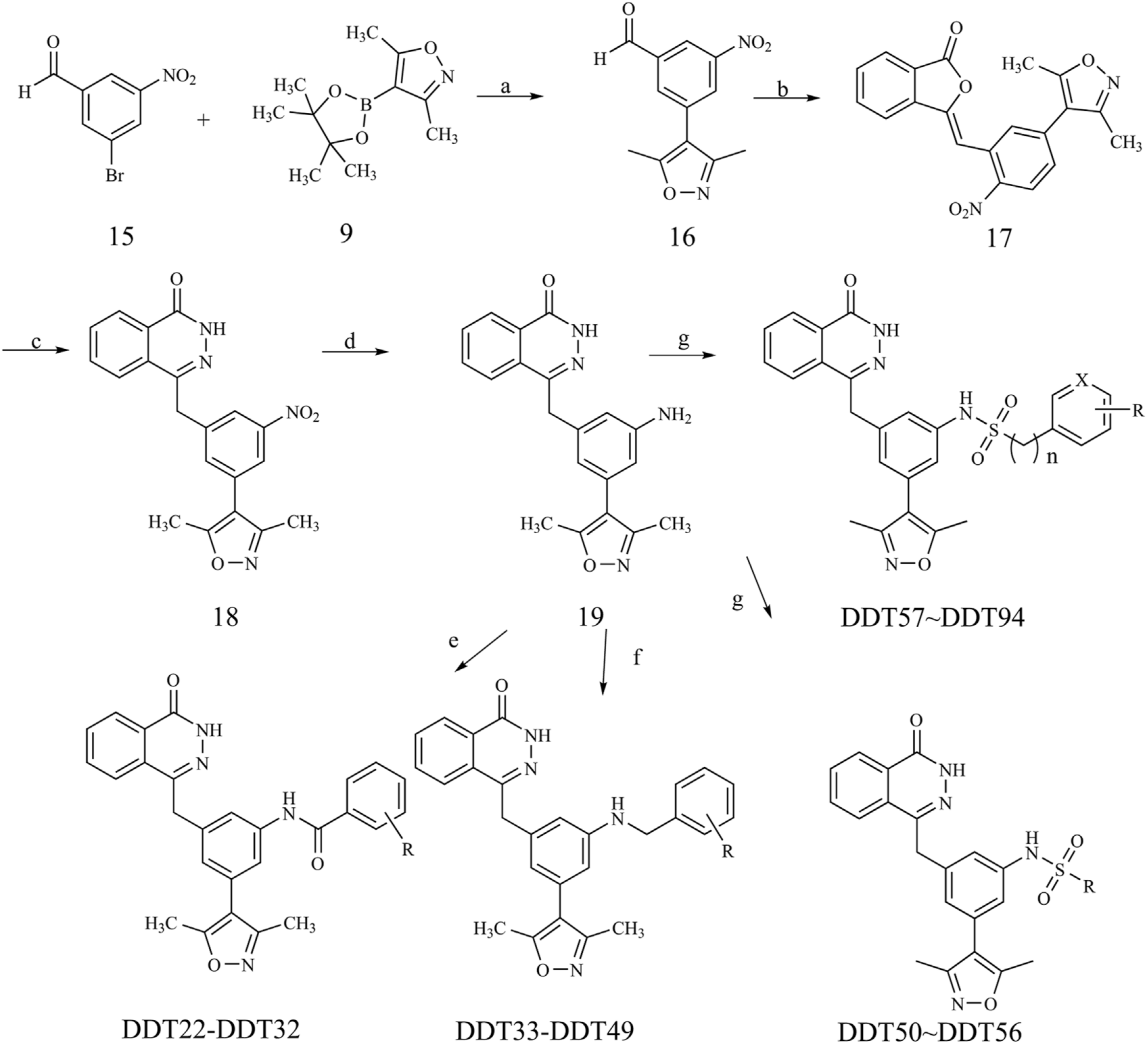
Synthetic route of compound **DDT22**-**DDT94**. Reagents and conditions: (a) Pd(PPh_3_)_4_, K_2_CO_3_, N_2_, dioxane/H_2_O (3:1), 90°C; (b) Dimethyl (3-oxo-1,3-dihydroisobenzofuran-1-yl)phosphonate, Et_3_N, THF, rt; (c) NH_2_NH_2_·H_2_O, EtOH, reflux; (d) Zn, NH_4_Cl, MeOH/H_2_O (3:1), 60°C; (e) DCM, DIPEA, rt; (f) NaBH_3_CN, MeOH, 80°C; (g) pyridine/DCM (1:1), rt.

The synthetic pathways for target compounds **DDT22**-**DDT94** are illustrated in Figure 10. Intermediate **18** was synthesized following a procedure analogous to that used for intermediate **13**. The essential intermediate **19** was derived by reducing the nitro group of intermediate **18** using zinc powder. This intermediate **19** was then reacted with the respective benzoyl chlorides to produce target compounds **DDT22**-**DDT32**. Additionally, NaBH_3_CN-mediated reductive amination reactions between intermediate **19** and the relevant benzaldehydes yielded target compounds **DDT33**-**DDT49**. Lastly, reactions between intermediate **19** and sulfonyl chlorides produced target compounds **DDT50**-**DDT94**. Detailed synthesis procedures and spectroscopic characterizations for compounds **DDT01**-**DDT94** can be found in the Supplementary Material.

### 4.2 Molecule docking

Crystal structures of BRD4 (PDB ID: 4J0S) and PARP1 (PDB ID: 5DS3) were chosen for molecular docking. The molecular docking studies were performed by the software Accelrys Discovery Studio (version 2016). The PARP1 protein structure was processed using the Protein Preparation module in this software, with all default parameters. The BRD4 protein structure was processed similarly to PARP1, except that a conserved water molecule (Water 311) was retained. The active sites were defined as spherical regions with a 10 Å diameter, centered on the ligand molecules. The Full Minimization module was employed to minimize the poses of compounds based on the CHARMm forcefield. Docking simulations were conducted using the CDOCKER module, in which twenty random conformations of per compound were generated. The top ten poses of per compound were saved based on CHARMm energy and the poses with the most favorable interactions were chosen for subsequent analysis.

### 4.3 Determination of inhibition rates and IC_50_ values against BRD4 and PARP1

The inhibitory effects of target compounds on BRD4 and PARP1 were assessed at Huawei Pharmaceutical Co. Ltd (Jinan, China).

The BRD4 activity assay utilized TR-FRET technology, employing a recombinant BRD4 (BD1 + BD2) and its corresponding ligand. The TR-FRET signal generated in the assay is proportional to the ligand’s binding to the bromodomains. All reactions contained a final DMSO concentration of 1%. All binding reactions were conducted at room temperature. Each 20 µL reaction mixture, in assay buffer, comprised bromodomains, the BET Ligand, and the specified inhibitor amount. For the negative control, 5 µL of assay buffer replaced the BET ligand. After a 120-min incubation with the ligand, the TR-FRET signal was captured using a Tecan Infinite M1000 plate reader. IC_50_ values were determined through nonlinear regression with a normalized dose-response fit in Prism GraphPad software.

The inhibition of PARP1 enzymatic activity by the tested compounds was gauged using an ELISA in 96-well plates. Each well was pre-coated with histone (20 μg/mL) in 100 μL of PBS buffer and incubated overnight at 4 °C. Subsequently, NAD^+^ (100 μM), biotinylated NAD^+^ (25 μM), and slDNA (200 nM) in 30 μL of reaction buffer were added to each well. This was followed by the addition of 5 μL of either the compound at varying concentrations or a solvent control. The reaction was initiated by adding 20 μL of PARP (50 ng/well) and incubating at 30°C for 1 h. Post-reaction, 50 µL of streptavidin-conjugated HRP was introduced, and the assay continued at 30°C for an additional 30 min. Finally, 100 μL of a solution containing H_2_O_2_ and luminol in 0.1 M citrate buffer (pH 5.4) was added, and the luminescent signal was recorded using a Molecular Devices SpectraMax M5 microplate reader. The inhibition rate of PARP1 enzymatic activity was determined as: (Lu control −Lu treated/Lu control) ×100%. IC_50_ values, representing the concentration required for 50% inhibition of PARP1 enzymatic activity, were determined using nonlinear regression with a normalized dose−response fit in Prism GraphPad software.

### 4.4 Cell culture

MDA-MB-468, MDA-MB-231, and MCF-7 cell lines were sourced from the National Collection of Authenticated Cell Culture, China. Cells were maintained in DMEM medium supplemented with 10% FBS and 1% penicillin/streptomycin, and incubated at 37°C in a humidified atmosphere containing 5% CO_2_.

### 4.5 Cell viability assay

The viability of cells was assessed using the MTT assay. Cells were seeded at a density of 3×10^3^ cells per well in a 96-well plate and treated with varying concentrations (0.05, 0.2, 0.8, 3.2, 6.4, 12.8 µM) of the compounds for 72 h. Post-incubation, 100 µL of MTT solution (0.5 mg/mL) was added to each well and further incubated for 4 h. The resulting formazan crystals were dissolved in 150 µL DMSO, and the absorbance was measured at 570 nm using a microplate reader.

### 4.6 Western blot analysis

For Western blotting, cells were treated with compound **DDT26**, JQ1, and Olaparib for a duration of 24 h. Cells were then lysed using RIPA buffer supplemented with protease and phosphatase inhibitors. Protein concentrations were determined using the BCA Protein Assay Kit. Equal amounts of protein (20–30 µg) were separated by SDS-polyacrylamide gel electrophoresis and subsequently transferred to a polyvinylidene fluoride (PVDF) membrane. The membrane was blocked using 5% milk in PBS prior to incubation with primary antibodies. Primary antibodies against p-H2AX (Cell Signaling Technology™, US), c-MYC, and β-actin (Proteintech, US) were used. After overnight incubation at 4°C, membranes were washed and incubated with HRP-conjugated secondary antibodies for 1 h. Protein bands were visualized using ECL reagents.

### 4.7 Cell cycle analysis

The cell cycle distribution was assessed using the DNA Content Quantitation Assay Kit (Solarbio, China). MCF-7 cells were seeded in six-well plates and exposed to different concentrations of **DDT26**, JQ1, Olaparib, or their combinations for 48 h. After treatment, cells were harvested, fixed in ice-cold 70% ethanol overnight at 4°C, and subsequently washed with PBS. Cells were then treated with 100 μL RNase at 37°C for 30 min and stained with 400 μL PI for 30 min at 4°C in the dark. The cell cycle distribution was analyzed using an Accuri C6 Plus flow cytometer (BD Bioscience, CA), and data interpretation was performed with FlowJo software.

### 4.8 Clonogenic assay

MCF-7 cells were seeded in 6-well plates at a density of 500 cells/well and allowed to grow for 8 days to facilitate colony formation. After incubation, cells were washed with PBS, fixed in methanol for 15 min, and stained with 1% crystal violet for an hour. Colonies were then imaged and counted.

### 4.9 Cell migration assay

MCF-7 cells were seeded in 6-well plates at a density of 5×10^5^ cells/well and incubated overnight. A scratch was made in the cell monolayer using a 10 µL pipette tip. After creating the scratch, cells were washed with PBS and treated with **DDT26**, JQ1, or Olaparib. Images were captured at 0, 12, 24, and 48-h intervals.

### 4.10 Comet assay

The comet assay was performed under alkaline conditions using the Comet Assay Kit (Beyotime, China) as per the manufacturer’s instructions. Briefly, after treating cells with **DDT26**, JQ1, Olaparib, or their combinations for 24 h, they were harvested and mixed with 0.7% low melting point agarose at a 1:7 (v/v) ratio. This mixture was spread onto microscope slides, which were then submerged in lysis solution for an hour. Electrophoresis was conducted in a horizontal apparatus at 25 V and 300 mA. Post-electrophoresis, slides were stained with PI for DNA visualization, and images were captured using a fluorescence inverted microscope.

## Data Availability

The datasets presented in this study can be found in online repositories. The names of the repository/repositories and accession number(s) can be found in the article/Supplementary Material.
