# Supplementary material for: Design, synthesis, and evaluation of 4-(3-(3,5-dimethylisoxazol-4-yl)benzyl)phthalazin-1(2H)-one derivatives: potent BRD4 inhibitors with anti-breast cancer activity
Source: Front Pharmacol. 2023 Nov 30;14:1289003. doi: 10.3389/fphar.2023.1289003 (PMC10720709; doi:10.3389/fphar.2023.1289003)
Supplement: Supplementary file 1 [file DataSheet1.PDF]

Supplementary Material

1 Synthesis procedures for compounds DDT01-DDT94

All reagents utilized in the experiments were sourced commercially and were directly employed without any further purification. The progress of all chemical reactions was monitored via thin-layer chromatography (TLC) under UV light at wavelengths of 254 and 365 nm. Compounds were purified using column chromatography on silica gel (200-300 mesh). Both ^{1}H NMR and ^{13}C NMR spectra for intermediates and final compounds were obtained using a Bruker Avance III (400 MHz) spectrometer, with tetramethylsilane (TMS) serving as the internal standard.

1.1 General synthesis of compounds 10 and 16

To a solvent mixture of 1,4-dioxane (9 mL) and water (3 mL), the following were added: 3,5-dibromobenzaldehyde (1.40 g, 5.0 mmol), 3,5-dimethylisoxazole-4-boronic acid pinacol ester (1.34 g, 6.0 mmol), K_2CO_3 (2.07 g, 15.0 mmol), and $Pd(PPh_3)_4$ (0.58 g, 0.5 mmol). The mixture was then stirred at 95°C for 4 hours under a nitrogen atmosphere. After cooling to room temperature, the mixture was filtered. The filtrate underwent extraction with dichloromethane (80 mL). The combined organic layers were subsequently dried over anhydrous Na_2SO_4, filtered, and the solvent was removed under reduced pressure. The resulting crude product was purified using column chromatography on silica gel, eluting with a petroleum ether/ethyl acetate mixture (10:1 ratio), yielding compound **10** as a white solid (55% yield). Compound **16** was synthesized following a similar procedure to that of compound **16**.

1.2 General synthesis of compounds 11

A solution of intermediate **10** (0.30 g, 1.1 mmol) in DMF (5 mL) was combined with $Pd(PPh_3)_4$ (0.06 g, 0.55 mmol) and $Zn(CN)_2$ (0.26 g, 2.2 mmol). The mixture was then heated to 80°C and maintained at this temperature for 5 hours under a nitrogen atmosphere. After cooling to room temperature, water was added to the reaction mixture, which was then filtered. The resulting filter cake was dried under reduced pressure and purified by column chromatography using a petroleum ether/ethyl acetate mixture (8:1 ratio) to yield intermediate **11** as a white solid. The yield was 68%.

1.3 General synthesis of compounds 12 and 17

To a solution of compound **11** (0.65 g, 2.9 mmol) in THF (15 mL), dimethyl (3-oxo-1,3-dihydroisobenzofuran-1-yl)phosphonate (0.72 g, 3.0 mmol) and triethylamine (Et_3N) (0.4 mL, 2.9 mmol) were added. The mixture was stirred at room temperature for 24 hours. The solvents were then removed under reduced pressure, and the filter cake was washed with water and dried under vacuum to yield compound **12** as a white solid. This compound was used directly in the subsequent step without further purification. Compound **17** was synthesized following a procedure similar to that for compound **5**.

1.4 General synthesis of compound 13 and 18

A solution of compound **12** (0.65 g, 1.9 mmol) in ethanol (15 mL) was treated with hydrazine hydrate (1.30 g, 26.6 mmol). The mixture was refluxed for 4 hours. After cooling to room temperature, the reaction mixture was filtered. The resulting filter cake was washed with ethanol to yield compound **13** directly, without further purification. The yield was 65%. Compound **18** was synthesized following a procedure similar to that for compound **13**.

1.5 General synthesis of compound 14

Intermediate **13** (1.50 g, 4.2 mmol) and NaOH (1.34 g, 33.6 mmol) were combined in a solvent mixture of methanol (5 mL) and water (5 mL). The mixture was refluxed for 8 hours. Upon cooling to room temperature, the pH of the reaction mixture was adjusted to 2 ~ 3 using 2N HCl. The mixture was then filtered, and the filter cake was washed with water and dried under vacuum to yield compound **14** directly, without further purification. The yield was 80%.

1.6 General synthesis of compounds DDT01-DDT21

Intermediate **14** (0.20 g, 0.53 mmol), EDCI (0.12 g, 0.64 mmol), HOBt (0.09 g, 0.64 mmol), and DIPEA (0.22 mL, 1.28 mmol) were dissolved in DMF (5 mL). The mixture was stirred at room temperature for 2 hours. Ammonia (0.4 mol/L in 1,4-Dioxane, 1.33 mL, 0.53 mmol) was then added, and the mixture was stirred for an additional 8 hours at room temperature. The mixture was then quenched with water (20 mL) and extracted with dichloromethane (30 mL). The organic layer was dried over anhydrous Na_2SO_4, filtered, and concentrated. The resulting crude product was purified by column chromatography using a dichloromethane/methanol mixture (10:1 ratio) to yield compound **DDT01** as a white solid. The yield was 22%. Compounds **DDT02**-**DDT21** were synthesized following a procedure analogous to that used for **DDT01**.

1.7 General synthesis of compound 19

Compound **18** (0.75 g, 2.0 mmol) and NH_4Cl (1.07 g, 20.0 mmol) were combined in a solvent mixture of methanol (15 mL) and water (5 mL). Zinc powder (1.63 g, 25.0 mmol) was incrementally added to the mixture. The reaction was maintained at 40°C for 5 hours. After concentration under vacuum, the residue was extracted with dichloromethane (100 mL). The organic layer was washed with brine, dried over Na_2SO_4, filtered, and concentrated. The resulting crude product was purified using column chromatography on silica gel, eluting with a dichloromethane/methanol mixture (60:1), to yield compound **19** as a white solid. The yield was 35%.

1.8 General synthesis of compounds DDT22-DDT32

Intermediate **19** (0.15 g, 0.43 mmol), benzoyl chloride (0.07 g, 0.47 mmol), and DIPEA (0.11 mL, 0.64 mmol) were dissolved in dichloromethane (5 mL). The mixture was stirred at room temperature for 12 hours. It was then quenched with water (20 mL) and extracted with dichloromethane (30 mL). The organic layer was dried over anhydrous Na_2SO_4, filtered, and concentrated. The resulting crude product was purified by column chromatography using a dichloromethane/methanol mixture (100:1) to yield compound **DDT22** as a yellow solid. The yield was 39%. Compounds **DDT23-DDT32** were synthesized following a procedure analogous to that used for **DDT22**.

1.9 General synthesis of compounds DDT33-DDT49

Intermediate **19** (0.69 g, 2.0 mmol) and benzaldehyde (0.21 g, 2.0 mmol) were dissolved in 1,2-dichloroethane (8 mL). $NaBH_3CN$ (0.25 g, 4.0 mmol) was added to the mixture at 0°C. The mixture was stirred overnight at room temperature. After concentration under vacuum, the residue was dissolved in dichloromethane (100 mL). The organic layer was washed with brine, dried over Na_2SO_4, filtered, and concentrated. The resulting crude product was purified using column chromatography on silica gel, eluting with a dichloromethane/methanol mixture (80:1), to yield compound DDT33 as a yellow solid. The yield was 38%. Compounds **DDT34-DDT49** were synthesized following a procedure analogous to that used for **DDT33**.

1.10 General synthesis of compounds DDT50-DDT94

Intermediate **19** (0.20 g, 0.58 mmol) and ethanesulfonyl chloride (0.08 g, 0.64 mmol) were combined in a solvent mixture of dichloromethane (2.5 mL) and pyridine (2.5 mL). The mixture was stirred under nitrogen protection at room temperature overnight. Following this, dichloromethane (100 mL) was added. The organic phase was sequentially washed with 2N HCl and brine, then dried over

Na_2SO_4, filtered, and concentrated under reduced pressure. The resulting crude product was purified using column chromatography on silica gel, eluting with a dichloromethane/methanol mixture (30:1), to yield compound **DDT50** as a white solid. The yield was 88%. Compounds **DDT51-DDT94** were synthesized following a procedure analogous to that used for **DDT50**.

2 Molecular docking study

Table S1. The docking scores and interactive residues of target compounds in protein BRD4 binding patterns

Molecular	-CDOCKER ENERGY	-CDOCKER INTERACTION ENERGY	Interactive residues
Compound 7	21.4171	34.4544	Pro82, Phe83, Val87, Leu92, Leu94, Cys136, Tyr139, Asn140, Ile146, Met149, Water 311
DDT14	13.1294	53.0869	Trp81, Pro82, Phe83, Pro86, Val87, Lys91, Leu92, Cys136, Asn140, Ile146, Met149
DDT23	23.2132	47.957	Pro82, Phe83, Val87, Lys91, Leu92, Leu94, Asn140, Ile146, Tyr139
DDT24	25.2438	48.33	Pro82, Phe83, Val87, Lys91, Leu92, Leu94, Cys136, Asn140, Ile146, Tyr139, Met149
DDT26	14.999	46.3379	Trp81, Pro82, Phe83, Val87, Leu92, Leu94, Tyr97, Asn140, Ile146, Met149, Water 311
DDT48	27.7724	47.5154	Trp81, Pro82, Phe83, Val87, Lys91, Leu92 Leu94, Tyr97, Asn140, Asp145, Ile146, Tyr139, Lys91
DDT49	28.6328	45.0512	Trp81, Pro82, Phe83, Val87, Leu92, Leu94, Asn140, Ile146, Tyr139, Lys91
DDT59	19.513	44.1582	Trp81, Pro82, Phe83, Gln85, Val87, Asp88, Lys91, Leu92, Leu94, Cys136, Asn140, Ile146

Table S2. The docking scores and interactive residues of target compounds in protein PARP1 binding patterns

Molecular	-CDOCKER ENERGY	-CDOCKER INTERACTION ENERGY	Interactive residues
Olaparib	36.3584	63.4788	His862, Gly863, Arg878, Gly894, Ile895, Tyr896, Ser904, Tyr907
DDT14	20.5334	52.5221	His862, Gly863, Arg865, Arg878, Ala898, Lys903, Ser904, Tyr907, His909
DDT23	27.5144	54.1185	His862, Gly863, Arg865, Leu877, Ala898, Lys903, Ser904, Tyr907, His909
DDT24	39.7999	60.8812	His862, Gly863, Arg865, Ile872, Arg878, Ala898, Lys903, Ser904, Tyr907, His909
DDT26	34.7953	60.1148	His862, Gly863, Arg865, Leu877, Arg878, Ala880, Tyr896, Ala898, Ser904, Tyr907
DDT48	44.6746	61.6054	His862, Gly863, Arg865, Leu877, Arg878, Ala898, Lys903, Ser904, Tyr907
DDT49	40.1729	54.3428	His862, Gly863, Arg865, Arg878, Ala898, Ser904, Tyr907
DDT59	35.2189	56.1704	His862, Gly863, Arg865, Ala880, Arg878, Ser904, Tyr907, His909

3 The purity of DDT26

As shown in Figure S1 and Table S3, the purity of **DDT26** was 99.4046% as determined by HPLC, which met the experimental requirements. Detailed HPLC experimental conditions were as following: chromatographic column: Waters®SPHERISORB®C18 (4.6×250 mm, 5μm); mobile phase system: acetonitrile: water (80: 20), isocratic elution; detection wavelength: 239 nm; flow rate: 1 mL/min; column temperature: 30 °C; input quantity: 20 μL, 200 μg/mL.

Figure S1. HPLC analysis of **DDT26** for purity assessment

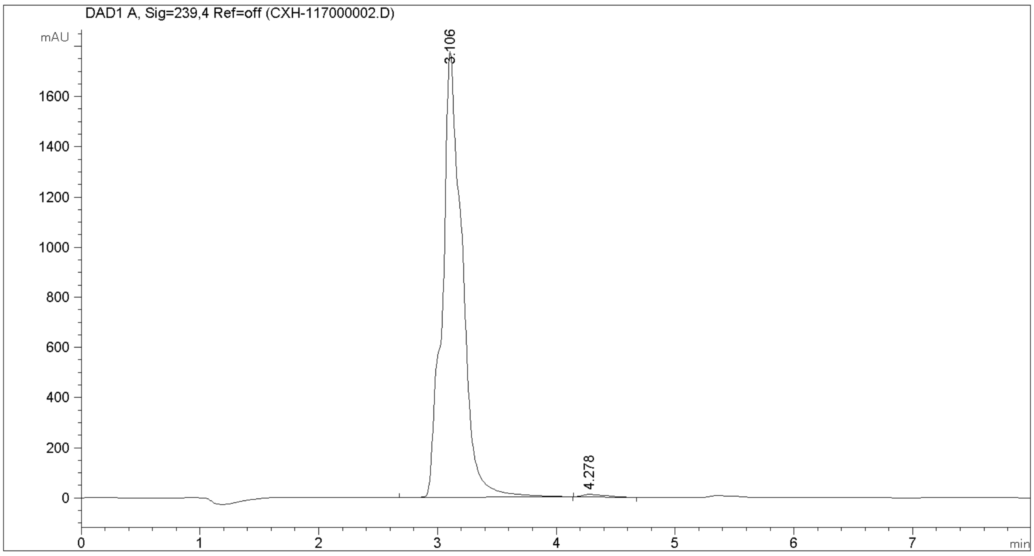

Table S3. Summary of HPLC peak parameters

Peak#	RT (min)	Peak Width (min)	Peak Height (mAU)	Peak Area (MAU*s)	Peak Area (%)
1	3.106	0.1548	1775.79602	20521.1	99.4046
2	4.278	0.1731	9.88457	122.9166	0.5954

4 The spectroscopic characterization of target compounds DDT01-DDT94

3-bromo-5-(3,5-dimethylisoxazol-4-yl)benzaldehyde (compound 10)

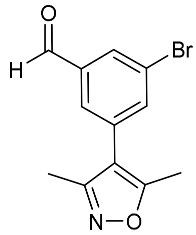

White solid, yield: 55%; mp: 126-127 °C; ^{1}H NMR (400 MHz, Chloroform-*d*) δ 10.01 (s, 1H), 8.01 (t, *J* = 1.5 Hz, 1H), 7.70 (t, *J* = 1.4 Hz, 1H), 7.66 (t, *J* = 1.7 Hz, 1H), 2.45 (s, 3H), 2.30 (s, 3H). ^{13}C NMR (101 MHz, Chloroform-*d*) δ 190.20, 166.20, 158.16, 138.32, 137.39, 133.72, 131.67, 128.45, 123.77, 114.58, 11.67, 10.76. HRMS (ESI): m/z calcd for $C_{12}H_{11}BrNO_2$ [M+H]$^{+}$ 279.9895, found 279.9961.

3-(3,5-dimethylisoxazol-4-yl)-5-formylbenzonitrile (compound 11)

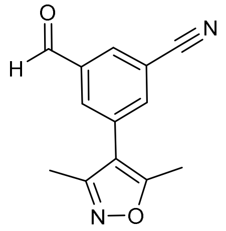

White solid, yield: 68%; mp: 195-196 °C; ^{1}H NMR (400 MHz, DMSO-*d*$_6$) δ 10.10 (s, 1H), 8.36 (t, *J* = 1.5 Hz, 1H), 8.26 (t, *J* = 1.7 Hz, 1H), 8.22 (t, *J* = 1.6 Hz, 1H), 2.47 (s, 3H), 2.28 (s, 3H). ^{13}C NMR (101 MHz, DMSO-*d*$_6$) δ 192.05, 167.13, 158.52, 137.79, 137.77, 134.48, 132.89, 132.17, 118.16, 114.26, 113.65, 11.81, 10.73. HRMS (ESI): m/z calcd for $C_{13}H_{11}N_2O_2$ [M+H]$^{+}$ 227.0742, found 227.0764.

3-(3,5-dimethylisoxazol-4-yl)-5-((4-oxo-3,4-dihydrophthalazin-1-yl)methyl)benzonitrile (compound 13)

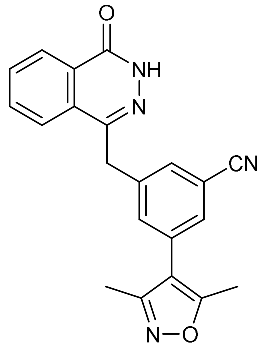

White solid, yield: 65%; mp: > 250 °C; ^{1}H NMR (400 MHz, DMSO-d_6) δ 12.59 (s, 1H), 8.28 (d, J = 7.8 Hz, 1H), 8.07 (d, J = 8.0 Hz, 1H), 7.94 (t, J = 7.5 Hz, 1H), 7.87 (d, J = 7.6 Hz, 1H), 7.83 (s, 1H), 7.79 (s, 1H), 7.72 (s, 1H), 4.46 (s, 2H), 2.38 (s, 3H), 2.20 (s, 3H). ^{13}C NMR (101 MHz, DMSO-d_6) δ 166.54, 159.83, 158.46, 144.89, 140.86, 134.84, 134.02, 132.08, 132.00, 131.83, 130.72, 129.60, 128.36, 126.56, 125.79, 118.98, 114.81, 112.59, 37.07, 11.79, 10.75. HRMS (ESI): m/z calcd for $C_{21}H_{17}N_4O_2$ $[M+H]^+$ 357.1273, found 357.1342.

3-(3,5-dimethylisoxazol-4-yl)-5-((4-oxo-3,4-dihydrophthalazin-1-yl)methyl)benzoic acid (compound 14)

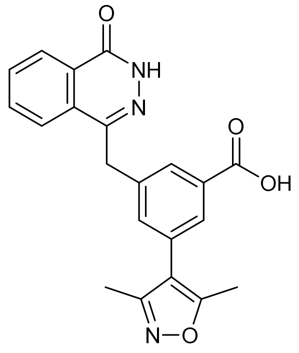

White solid, yield 80%; mp: >250 °C; ^{1}H NMR (400 MHz, DMSO-d_6) δ 12.56 (s, 1H), 8.20 (d, J = 7.5 Hz, 1H), 8.00 (d, J = 8.0 Hz, 1H), 7.85 (t, J = 7.4 Hz, 1H), 7.82 (s, 1H), 7.77 (t, J = 7.4 Hz, 1H), 7.67 (s, 1H), 7.59 (s, 1H), 4.39 (s, 2H), 2.30 (s, 3H), 2.12 (s, 3H). ^{13}C NMR (101 MHz, DMSO-d_6) δ 167.39, 165.98, 159.83, 158.42, 145.42, 139.90, 134.13, 134.00, 132.24, 132.07, 130.83, 129.58, 128.98, 128.35, 127.95, 126.56, 126.00, 115.60, 37.41, 11.77, 10.84. HRMS (ESI): m/z calcd for $C_{21}H_{18}N_3O_4$ $[M-H]^-$ 374.1219, found 374.1143.

3-(3,5-dimethylisoxazol-4-yl)-5-((4-oxo-3,4-dihydrophthalazin-1-yl)methyl)benzamide (DDT01)

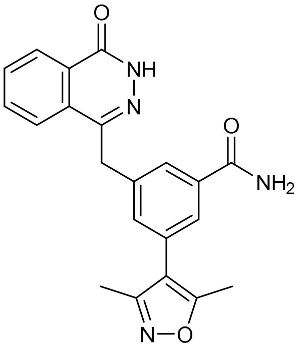

White solid, yield :12%; mp: >250 °C; ^{1}H NMR (400 MHz, DMSO-d_6) δ 12.61 (s, 1H), 8.28 (d, *J* = 7.7 Hz, 1H), 8.04 (d, J = 8.0 Hz, 1H), 8.01 (s, 1H), 7.90 (t, *J* = 7.6 Hz, 1H), 7.86 – 7.80 (m, 2H), 7.69 (s, 1H), 7.54 (s, 1H), 7.43 (s, 1H), 4.42 (s, 2H), 2.37 (s, 3H), 2.19 (s, 3H). ^{13}C NMR (101 MHz, DMSO-d_6) δ 167.82, 165.89, 159.87, 158.54, 145.36, 139.56, 135.56, 133.94, 132.56, 132.01, 130.48, 129.63, 128.40, 127.57, 126.55, 126.11, 126.02, 115.88, 37.73, 11.77, 10.83. HRMS (ESI): m/z calcd for $C_{21}H_{19}N_4O_3$ $[M+H]^+$ 375.1379, found 375.1457.

3-(3,5-dimethylisoxazol-4-yl)-N-methyl-5-((4-oxo-3,4-dihydrophthalazin-1-yl)methyl)benzamide (DDT02)

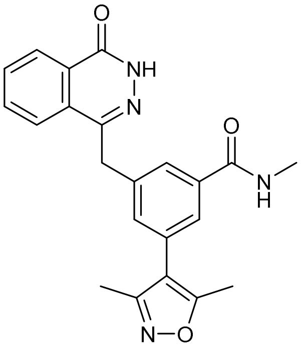

Yellow solid, yield: 41.3%; mp: >250 °C; ^{1}H NMR (400 MHz, DMSO-d_6) δ 12.61 (s, 1H), 8.45 (d, *J* = 4.4 Hz, 1H), 8.28 (d, *J* = 7.5 Hz, 1H), 8.04 (d, *J* = 7.9 Hz, 1H), 7.90 (t, *J* = 7.1 Hz, 1H), 7.83 (t, *J* = 7.4 Hz, 1H), 7.78 (s, 1H), 7.63 (s, 1H), 7.54 (s, 1H), 4.43 (s, 2H), 2.78 (d, *J* = 4.5 Hz, 3H), 2.37 (s, 3H), 2.19 (s, 3H). ^{13}C NMR (101 MHz, DMSO-d_6) δ 166.61, 165.88, 159.87, 158.51, 145.35, 139.62, 135.87, 133.93, 132.36, 132.00, 130.52, 129.62, 128.40, 127.19, 126.55, 126.01, 125.70, 115.87, 37.75, 26.72, 11.76, 10.84. HRMS (ESI): m/z calcd for $C_{22}H_{21}N_4O_3$ [M+H] + 389.1535, found 389.1608.

N-cyclopentyl-3-(3,5-dimethylisoxazol-4-yl)-5-((4-oxo-3,4-dihydrophthalazin-1-yl)methyl)benzamide (DDT03)

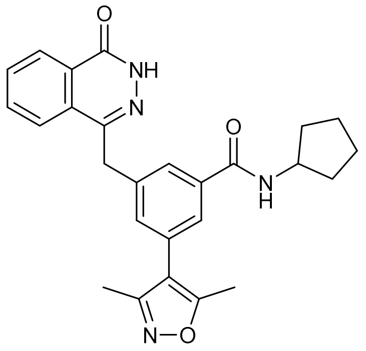

White solid, yield: 87.8%; mp: >250 °C; ^{1}H NMR (400 MHz, DMSO-d_6) δ 12.62 (s, 1H), 8.28 (d, J = 7.4 Hz, 2H), 8.05 (d, J = 8.0 Hz, 1H), 7.90 (t, J = 7.2 Hz, 1H), 7.85 (d, J = 7.6 Hz, 1H), 7.81 (s, 1H), 7.68 (s, 1H), 7.50 (s, 1H), 4.43 (s, 2H), 4.21 (d, J = 6.8 Hz, 1H), 2.36 (s, 3H), 2.17 (s, 3H), 1.88 (s, 2H), 1.68 (s, 2H), 1.52 (s, 4H). ^{13}C NMR (101 MHz, DMSO-d_6) δ 165.89, 165.82, 159.85, 158.53, 145.39, 139.44, 136.11, 133.92, 132.29, 132.01, 130.39, 129.61, 128.37, 127.48, 126.55, 126.01, 125.98, 115.93, 51.47, 37.73, 32.55, 24.12, 11.73, 10.83. HRMS (ESI): m/z calcd for $C_{26}H_{27}N_4O_3$ $[M+H]^+$ 443.2005, found 443.2076.

N-cyclohexyl-3-(3,5-dimethylisoxazol-4-yl)-5-((4-oxo-3,4-dihydrophthalazin-1-yl)methyl)benzamide(DDT04)

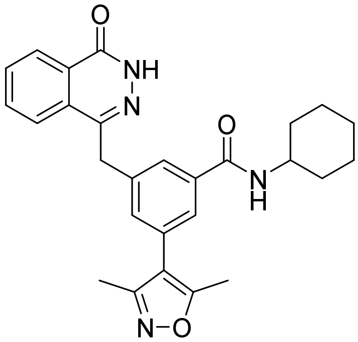

White solid, yield: 60.8%; mp: 178-179 °C; ^{1}H NMR (400 MHz, DMSO-d_6) δ 12.60 (s, 1H), 8.28 (d, J = 7.6 Hz, 1H), 8.19 (d, J = 7.8 Hz, 1H), 8.04 (d, J = 7.9 Hz, 1H), 7.90 (t, J = 7.2 Hz, 1H), 7.84 (d, J = 7.5 Hz, 1H), 7.81 (s, 1H), 7.67 (s, 1H), 7.49 (s, 1H), 4.42 (s, 2H), 3.75 (s, 1H), 2.35 (s, 3H), 2.17 (s, 3H), 1.76 (d, J = 34.7 Hz, 4H), 1.60 (d, J = 11.9 Hz, 1H), 1.29 (t, J = 9.6 Hz, 4H), 1.13 (s, 1H). ^{13}C NMR (101 MHz, DMSO-d_6) δ 165.82, 165.31, 159.85, 158.52, 145.38, 139.45, 136.20, 133.92, 132.30,

132.00, 130.40, 129.63, 128.38, 127.48, 126.55, 126.00, 125.95, 115.94, 48.91, 37.74, 32.85, 25.73, 25.39, 11.72, 10.81. HRMS (ESI): m/z calcd for $C_{27}H_{29}N_4O_3$ $[M+H]^+$ 457.2161, found 457.2235.

3-(3,5-dimethylisoxazol-4-yl)-5-((4-oxo-3,4-dihydrophthalazin-1-yl)methyl)-N-phenylbenzamide (DDT05)

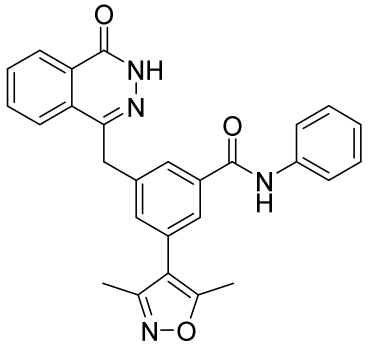

White solid, yield: 58%; mp: 192-193 °C; ^{1}H NMR (400 MHz, DMSO-d_6) δ 12.61 (s, 1H), 10.23 (s, 1H), 8.28 (d, J = 7.3 Hz, 1H), 8.08 (d, J = 8.0 Hz, 1H), 7.91 (d, J = 5.5 Hz, 2H), 7.84 (t, J = 7.5 Hz, 1H), 7.78 (s, 1H), 7.75 (s, 1H), 7.73 (s, 1H), 7.59 (s, 1H), 7.35 (t, J = 7.9 Hz, 2H), 7.11 (t, J = 7.4 Hz, 1H), 4.47 (s, 2H), 2.39 (s, 3H), 2.21 (s, 3H). ^{13}C NMR (101 MHz, DMSO-d_6) δ 165.99, 165.61, 159.86, 158.54, 145.34, 139.72, 139.45, 136.43, 133.95, 132.83, 132.02, 130.65, 129.65, 129.06, 128.42, 127.72, 126.58, 126.32, 126.00, 124.27, 120.99, 115.83, 37.70, 11.77, 10.84. HRMS (ESI): m/z calcd for $C_{27}H_{23}N_4O_3$ $[M+H]^+$ 451.1692, found 451.1770.

N-benzyl-3-(3,5-dimethylisoxazol-4-yl)-5-((4-oxo-3,4-dihydrophthalazin-1-yl)methyl)benzamide (DDT06)

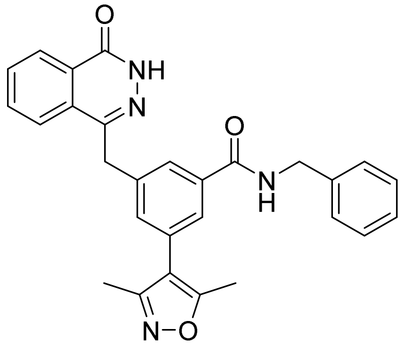

White solid, yield: 51.6%; mp: >250 °C; ^{1}H NMR (400 MHz, DMSO-d_6) δ 12.60 (s, 1H), 9.06 (t, J = 5.9 Hz, 1H), 8.27 (d, J = 6.8 Hz, 1H), 8.05 (d, J = 7.9 Hz, 1H), 7.91 (d, J = 7.2 Hz, 1H), 7.88 – 7.78 (m, 2H), 7.72 (s, 1H), 7.54 (s, 1H), 7.35 – 7.27 (m, 4H), 7.26 – 7.21 (m, 1H), 4.48 (d, J = 5.9 Hz, 2H), 4.43 (s, 2H), 2.37 (s, 3H), 2.19 (s, 3H). ^{13}C NMR (101 MHz, DMSO-d_6) δ 166.16, 165.90, 159.86, 158.53, 145.35, 139.99, 139.67, 135.61, 133.94, 132.62, 132.02, 130.59, 129.64, 128.75, 128.40, 127.69, 127.47, 127.23, 126.56, 126.01, 125.88, 115.87, 43.15, 37.73, 11.76, 10.83. HRMS (ESI): m/z calcd for $C_{28}H_{25}N_4O_3$ [M+H]$^+$ 465.1848, found 465.1929.

3-(3,5-dimethylisoxazol-4-yl)-5-((4-oxo-3,4-dihydrophthalazin-1-yl)methyl)-N-(pyridin-3-ylmethyl)benzamide(DDT07)

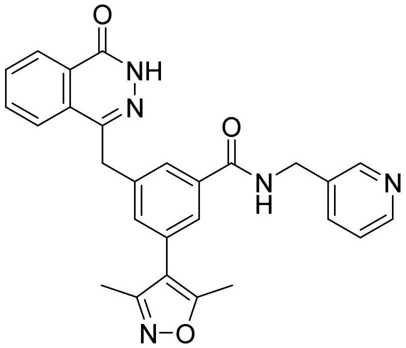

White solid, yield: 43.7%; mp: >250 °C; ^{1}H NMR (400 MHz, DMSO-d_6) δ 12.60 (s, 1H), 9.12 (t, J = 5.9 Hz, 1H), 8.55 (d, J = 1.8 Hz, 1H), 8.46 (d, J = 3.3 Hz, 1H), 8.27 (d, J = 6.8 Hz, 1H), 8.04 (d, J = 7.9 Hz, 1H), 7.90 (t, J = 6.9 Hz, 1H), 7.84 (d, J = 6.8 Hz, 2H), 7.71 (s, 2H), 7.55 (s, 1H), 7.38 – 7.31 (m, 1H), 4.49 (d, J = 5.8 Hz, 2H), 4.43 (s, 2H), 2.36 (s, 3H), 2.18 (s, 3H). ^{13}C NMR (101 MHz, DMSO-d_6) δ 166.32, 165.92, 159.85, 158.52, 149.30, 148.58, 145.34, 139.70, 135.60, 135.43, 135.39, 133.95, 132.74, 132.03, 130.62, 129.62, 128.38, 127.46, 126.56, 126.01, 125.90, 123.95, 115.84, 40.95, 37.71, 11.76, 10.83. HRMS (ESI): m/z calcd for $C_{27}H_{24}N_5O_3$ [M+H]$^+$ 466.1801, found 466.1871.

3-(3,5-dimethylisoxazol-4-yl)-5-((4-oxo-3,4-dihydrophthalazin-1-yl)methyl)-N-(tetrahydro-2H-pyran-4-yl)benzamide (DDT08)

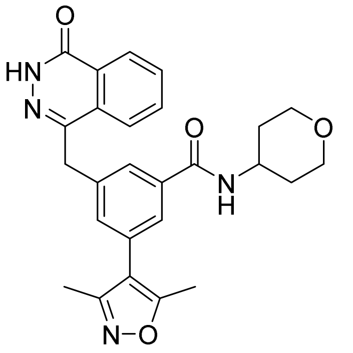

White solid, yield: 63%; mp: 153-154 °C; ^{1}H NMR (400 MHz, DMSO-d_6) δ 12.61 (s, 1H), 8.33 – 8.24 (m, 2H), 8.05 (d, *J* = 7.9 Hz, 1H), 7.90 (t, *J* = 7.0 Hz, 1H), 7.84 (d, *J* = 7.9 Hz, 1H), 7.81 (s, 1H), 7.67 (s, 1H), 7.51 (s, 1H), 4.43 (s, 2H), 4.05 – 3.93 (m, 1H), 3.87 (d, *J* = 13.4 Hz, 2H), 3.39 (d, *J* = 11.8 Hz, 2H), 2.35 (s, 3H), 2.17 (s, 3H), 1.75 (d, *J* = 12.6 Hz, 2H), 1.62 – 1.49 (m, 2H). ^{13}C NMR (101 MHz, DMSO-d_6) δ 165.86, 165.56, 159.85, 158.53, 145.39, 139.52, 135.97, 133.94, 132.48, 132.03, 130.45, 129.61, 128.37, 127.49, 126.56, 126.01, 125.96, 115.90, 66.62, 46.33, 37.72, 32.86, 11.73, 10.82. HRMS (ESI): m/z calcd for $C_{26}H_{27}N_4O_4$ $[M+H]^+$ 459.1954, found 459.2026.

3-(3,5-dimethylisoxazol-4-yl)-N-(1,1-dioxidotetrahydro-2H-thiopyran-4-yl)-5-((4-oxo-3,4-dihydrophthalazin-1-yl)methyl)benzamide (DDT09)

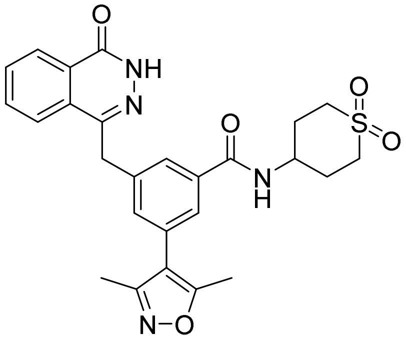

White solid, yield: 68.4%; mp: 189-190 °C; ^{1}H NMR (400 MHz, DMSO-d_6) δ 12.61 (s, 1H), 8.40 (d, *J* = 6.8 Hz, 1H), 8.28 (d, *J* = 7.1 Hz, 1H), 8.05 (d, *J* = 7.2 Hz, 1H), 7.90 (s, 1H), 7.82 (s, 2H), 7.68 (s, 1H), 7.52 (s, 1H), 4.43 (s, 2H), 4.20 (s, 1H), 3.30 – 3.26 (m, 2H), 3.11 (d, *J* = 12.0 Hz, 2H), 2.36 (s, 3H), 2.17 (s, 3H), 2.11 (s, 4H). ^{13}C NMR (101 MHz, DMSO-d_6) δ 165.88, 165.76, 159.85, 158.53, 145.38, 139.56, 135.66, 133.94, 132.65, 132.04, 130.50, 129.61, 128.37, 127.57, 126.56, 126.03, 125.99, 115.88, 49.23, 45.29, 37.71, 29.71, 11.72, 10.81. HRMS (ESI): m/z calcd for $C_{26}H_{27}N_4O_5S$ $[M+H]^+$ 507.1624, found 507.1698.

3-(3,5-dimethylisoxazol-4-yl)-N-((1r,4r)-4-hydroxycyclohexyl)-5-((4-oxo-3,4-dihydrophthalazin-1-yl)methyl)benzamide (DDT10)

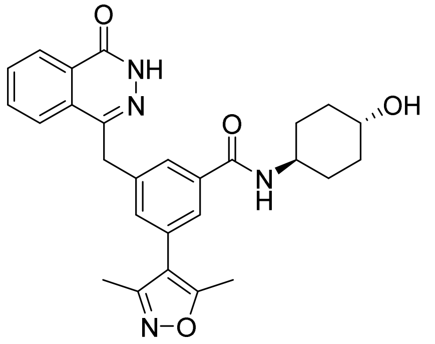

White solid, yield: 62.5%; mp: 199-200 °C; ^{1}H NMR (400 MHz, DMSO-d_6) δ 12.60 (s, 1H), 8.28 (d, J = 7.1 Hz, 1H), 8.18 (d, J = 7.8 Hz, 1H), 8.04 (d, J = 7.9 Hz, 1H), 7.90 (t, J = 8.1 Hz, 1H), 7.84 (d, J = 7.4 Hz, 1H), 7.81 (d, J = 6.4 Hz, 1H), 7.66 (s, 1H), 7.49 (s, 1H), 4.56 (d, J = 4.4 Hz, 1H), 4.42 (s, 2H), 3.82 – 3.64 (m, 1H), 3.42 – 3.37 (m, 1H), 2.35 (s, 3H), 2.16 (s, 3H), 1.83 (t, J = 14.4 Hz, 4H), 1.36 (q, J = 11.8, 11.2 Hz, 2H), 1.23 (q, J = 10.5 Hz, 2H). ^{13}C NMR (101 MHz, DMSO-d_6) δ 165.83, 165.50, 159.85, 158.53, 145.39, 139.46, 136.10, 133.93, 132.35, 132.01, 130.41, 129.62, 128.37, 127.46, 126.55, 126.00, 125.93, 115.92, 68.80, 48.50, 37.73, 34.68, 30.73, 11.72, 10.81. HRMS (ESI): m/z calcd for $C_{27}H_{29}N_4O_4$ [M+H]$^+$ 473.2111, found 473.2187.

3-(3,5-dimethylisoxazol-4-yl)-N-((1s,4s)-4-hydroxycyclohexyl)-5-((4-oxo-3,4-dihydrophthalazin-1-yl)methyl)benzamide (DDT11)

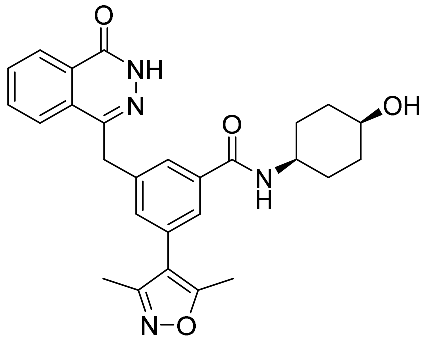

White solid, yield: 78.3%; mp: 214-215 °C; ^{1}H NMR (400 MHz, DMSO-d_6) δ 12.61 (s, 1H), 8.28 (d, J = 8.9 Hz, 1H), 8.21 (d, J = 7.7 Hz, 1H), 8.05 (d, J = 7.9 Hz, 1H), 7.95 – 7.86 (m, 1H), 7.88 – 7.80 (m, 2H), 7.69 (s, 1H), 7.48 (s, 1H), 4.42 (s, 2H), 4.37 (d, J = 2.7 Hz, 1H), 3.78 (s, 2H), 2.35 (s, 3H), 2.17 (s, 3H), 1.81 – 1.63 (m, 4H), 1.54 – 1.42 (m, 4H). ^{13}C NMR (101 MHz, DMSO-d_6) δ 165.81,

165.35, 159.85, 158.54, 145.41, 139.41, 136.16, 133.92, 132.27, 132.01, 130.38, 129.63, 128.38, 127.57, 126.55, 126.01, 125.99, 115.95, 64.01, 48.19, 37.73, 31.84, 26.97, 11.72, 10.82. HRMS (ESI): m/z calcd for $C_{27}H_{29}N_4O_4$ [M+H]$^+$ 473.2111, found 473.2184.

4-(3-(3,5-dimethylisoxazol-4-yl)-5-(3-oxopyrrolidine-1-carbonyl)benzyl)phthalazin-1(2H)-one (DDT12)

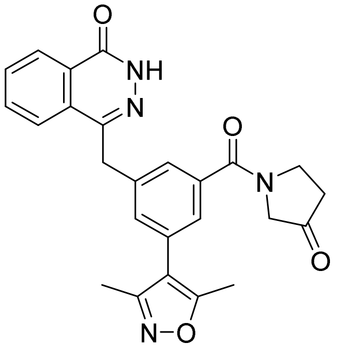

Yellow solid, yield: 75%; mp: 241-243 °C; ^{1}H NMR (400 MHz, DMSO-d_6) δ 12.61 (s, 1H), 8.28 (d, J = 6.8 Hz, 1H), 8.07 (d, J = 7.9 Hz, 1H), 7.91 (t, J = 7.3 Hz, 1H), 7.84 (t, J = 8.0 Hz, 1H), 7.51 (d, J = 12.4 Hz, 1H), 7.46 (s, 1H), 7.36 (s, 1H), 4.43 (s, 2H), 3.98 – 3.72 (m, 4H), 2.58 (t, J = 7.6 Hz, 2H), 2.38 (s, 3H), 2.20 (s, 3H). ^{13}C NMR (101 MHz, DMSO-d_6) δ 211.30, 168.75, 165.95, 159.85, 158.49, 145.36, 139.67, 136.76, 133.95, 132.04, 131.31, 130.53, 129.64, 128.41, 127.03, 126.57, 126.02, 125.83, 115.75, 52.65, 46.25, 42.76, 37.52, 11.82, 10.88. HRMS (ESI): m/z calcd for $C_{25}H_{23}N_4O_4$ [M+H]$^+$ 443.1641, found 443.1708.

(R)-4-(3-(3,5-dimethylisoxazol-4-yl)-5-(3-hydroxypyrrolidine-1-carbonyl)benzyl)phthalazin-1(2H)-one (DDT13)

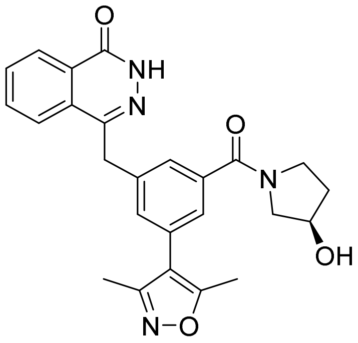

White solid, yield: 64%; mp: 235-236 °C; ^{1}H NMR (400 MHz, DMSO-d_6) δ 12.61 (s, 1H), 8.27 (d, J = 7.7 Hz, 1H), 8.08 (d, J = 8.0 Hz, 1H), 7.91 (t, J = 7.6 Hz, 1H), 7.84 (t, J = 7.5 Hz, 1H), 7.46 (s, 2H), 7.33 (s, 1H), 4.96 (d, J = 30.7 Hz, 1H), 4.42 (s, 2H), 4.27 (d, J = 40.6 Hz, 1H), 3.56 – 3.51 (m, 2H), 3.37 (s, 1H), 3.18 (d, J = 5.3 Hz, 1H), 2.36 (s, 3H), 2.19 (s, 3H), 1.98 – 1.72 (m, 2H). ^{13}C NMR (101 MHz, DMSO-d_6) δ 165.90, 159.83, 158.49, 145.45, 139.50, 138.21, 133.97, 132.06, 131.05, 130.34, 129.61, 128.37, 127.09, 126.97, 126.56, 126.05, 125.80, 115.79, 69.83, 68.47, 57.44, 54.82, 47.29, 44.59, 37.48, 34.82, 32.65, 11.82, 10.90. HRMS (ESI): m/z calcd for $C_{25}H_{25}N_4O_4$ $[M+H]^+$ 445.1798, found 445.1868.

(S)-4-(3-(3,5-dimethylisoxazol-4-yl)-5-(3-hydroxypyrrolidine-1-carbonyl)benzyl)phthalazin-1(2H)-one (DDT14)

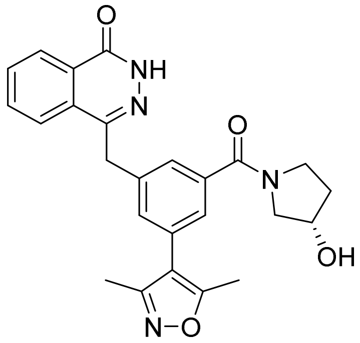

White solid, yield: 70%; mp: 204-205 °C; ^{1}H NMR (400 MHz, DMSO-d_6) δ 12.61 (s, 1H), 8.28 (d, J = 7.3 Hz, 1H), 8.08 (d, J = 8.0 Hz, 1H), 7.91 (t, J = 7.6 Hz, 1H), 7.84 (t, J = 7.5 Hz, 1H), 7.46 (s, 2H), 7.33 (s, 1H), 4.97 (d, J = 34.3 Hz, 1H), 4.42 (s, 2H), 4.27 (d, J = 41.3 Hz, 1H), 3.55 (d, J = 16.1 Hz, 2H), 3.37 (s, 1H), 3.19 (d, J = 10.9 Hz, 1H), 2.37 (s, 3H), 2.19 (s, 3H), 1.94 – 1.76 (m, 2H). ^{13}C NMR (101 MHz, DMSO-d_6) δ 168.35, 168.27, 165.90, 159.83, 158.48, 145.45, 139.50, 138.22, 133.96, 132.05, 131.05, 130.35, 129.62, 128.38, 127.09, 126.57, 126.04, 125.80, 115.79, 69.83, 68.47, 57.44, 54.82, 47.29, 44.59, 37.48, 34.83, 32.65, 11.82, 10.89. HRMS (ESI): m/z calcd for $C_{25}H_{25}N_4O_4$ $[M+H]^+$ 445.1798, found 445.1871

4-(3-(3,5-dimethylisoxazol-4-yl)-5-(piperazine-1-carbonyl)benzyl)phthalazin-1(2H)-one (DDT15)

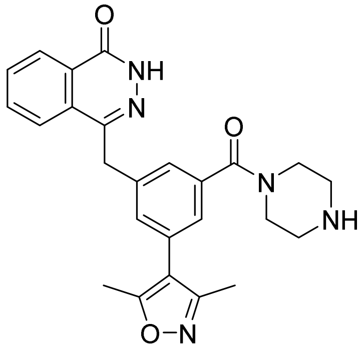

White solid, yield: 72.5%; mp: 190-191 °C; ^{1}H NMR (400 MHz, DMSO-d_6) δ 12.62 (s, 1H), 8.28 (d, J = 7.7 Hz, 1H), 8.06 (d, J = 8.0 Hz, 1H), 7.91 (t, J = 8.1 Hz, 1H), 7.84 (t, J = 7.5 Hz, 1H), 7.47 (s, 1H), 7.32 (s, 1H), 7.24 (s, 1H), 4.42 (s, 2H), 3.59 (s, 2H), 3.29 (s, 2H), 2.92 – 2.64 (m, 4H), 2.38 (s, 3H), 2.20 (s, 3H), 1.23 (s, 1H). ^{13}C NMR (101 MHz, DMSO-d_6) δ 168.86, 165.93, 159.84, 158.47, 145.38, 139.69, 137.09, 133.95, 132.05, 130.76, 130.70, 130.61, 129.62, 128.38, 126.68, 126.57, 126.01, 125.56, 115.69, 45.31, 37.49, 11.83, 10.88. HRMS (ESI): m/z calcd for $C_{25}H_{26}N_5O_3$ [M+H]$^+$ 444.1957, found 444.2020.

4-(3-(3,5-dimethylisoxazol-4-yl)-5-(morpholine-4-carbonyl)benzyl)phthalazin-1(2H)-one(DDT16)

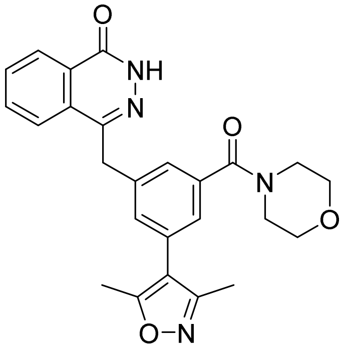

White solid, yield: 100%; mp: 241-242 °C; ^{1}H NMR (400 MHz, DMSO-d_6) δ 12.61 (s, 1H), 8.28 (d, J = 7.8 Hz, 1H), 8.06 (d, J = 7.9 Hz, 1H), 7.91 (t, J = 7.0 Hz, 1H), 7.84 (t, J = 7.5 Hz, 1H), 7.47 (s, 1H), 7.34 (s, 1H), 7.25 (s, 1H), 4.42 (s, 2H), 3.59 (s, 8H), 2.37 (s, 3H), 2.19 (s, 3H). ^{13}C NMR (101 MHz, DMSO-d_6) δ 168.96, 165.94, 159.84, 158.46, 145.34, 139.72, 136.79, 133.95, 132.04, 130.83, 130.65, 129.64, 128.40, 126.83, 126.57, 125.98, 125.66, 115.69, 66.45, 37.48, 11.81, 10.86. HRMS (ESI): m/z calcd for $C_{25}H_{25}N_4O_4$ [M+H]$^+$ 445.1798, found 445.1870.

4-(3-(4-(cyclopropanecarbonyl)piperazine-1-carbonyl)-5-(3,5-dimethylisoxazol-4-yl)benzyl)phthalazin-1(2H)-one (DDT17)

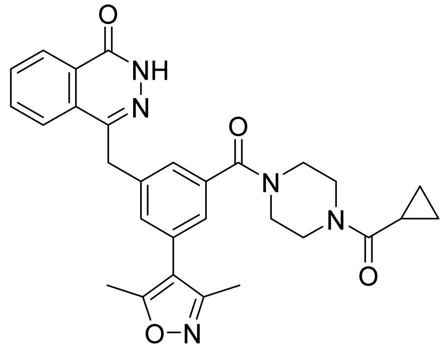

White solid, yield: 100%; mp: 154-155 °C; ^{1}H NMR (400 MHz, DMSO-d_6) δ 12.60 (s, 1H), 8.27 (d, J = 8.6 Hz, 1H), 8.06 (d, J = 8.0 Hz, 1H), 7.91 (t, J = 8.2 Hz, 1H), 7.84 (t, J = 7.2 Hz, 1H), 7.48 (s, 1H), 7.36 (s, 1H), 7.27 (s, 1H), 4.42 (s, 2H), 3.82 – 3.40 (m, 8H), 2.38 (s, 3H), 2.20 (s, 3H), 1.95 (s, 1H), 0.78 – 0.67 (m, 4H). ^{13}C NMR (101 MHz, DMSO-d_6) δ 171.75, 169.09, 165.98, 159.85, 158.49, 145.33, 139.74, 136.88, 133.97, 132.06, 130.90, 130.62, 129.65, 128.39, 126.88, 126.58, 126.00, 125.65, 115.68, 37.48, 11.85, 10.89, 10.82, 7.59. HRMS (ESI): m/z calcd for $C_{29}H_{30}N_5O_4$ [M+H]$^+$ 512.2220, found 512.2291.

4-(3-(3,5-dimethylisoxazol-4-yl)-5-(2-(trifluoromethyl)-5,6,7,8-tetrahydro-[1,2,4]triazolo[1,5-a]pyrazine-7-carbonyl)benzyl)phthalazin-1(2H)-one (DDT18)

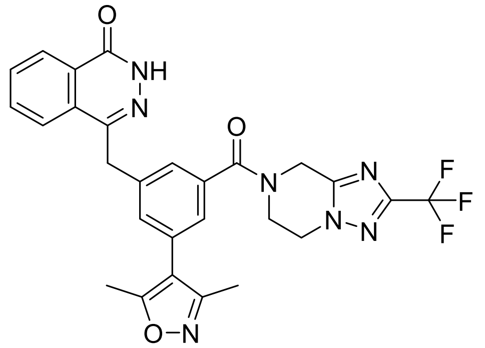

White solid, yield: 88%; mp: 161-162 °C; ^{1}H NMR (400 MHz, DMSO-d_6) δ 12.60 (s, 1H), 8.28 (d, J = 8.7 Hz, 1H), 8.07 (d, J = 7.9 Hz, 1H), 7.92 (t, J = 8.3 Hz, 1H), 7.84 (t, J = 7.5 Hz, 1H), 7.53 (s, 1H), 7.46 (s, 1H), 7.37 (s, 1H), 4.91 (s, 2H), 4.44 (s, 2ffH), 4.29 (s, 2H), 3.91 (s, 2H), 2.39 (s, 3H), 2.21 (s, 3H). ^{13}C NMR (101 MHz, DMSO-d_6) δ 169.82, 166.02, 159.86, 158.49, 151.92 (q, J = 31.1 Hz), 145.28, 139.89, 136.03, 133.96, 132.03, 131.44, 130.82, 129.66, 128.42, 126.98, 126.58, 125.96,

125.74, 119.91 (d, J = 269.3 Hz), 115.65, 55.35, 47.31, 37.52, 11.83, 10.87. HRMS (ESI): m/z calcd for $C_{27}H_{23}F_3N_7O_3$ $[M+H]^+$ 550.1736, found 550.1089.

(S)-4-(3-(3,5-dimethylisoxazol-4-yl)-5-(3-hydroxypiperidine-1-carbonyl)benzyl)phthalazin-1(2H)-one (DDT19)

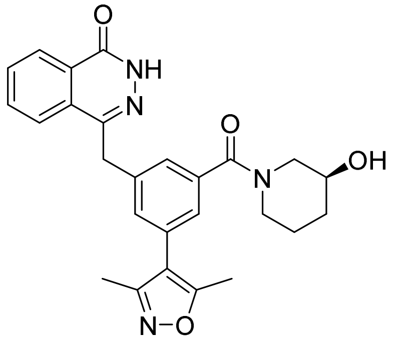

White solid, yield: 57.3%; mp: 157-158 °C; ^{1}H NMR (400 MHz, DMSO-d_6) δ 12.60 (s, 1H), 8.27 (d, J = 8.9 Hz, 1H), 8.06 (d, J = 7.8 Hz, 1H), 7.91 (t, J = 7.2 Hz, 1H), 7.84 (t, J = 7.9 Hz, 1H), 7.44 (s, 1H), 7.32 (d, J = 21.4 Hz, 1H), 7.23 (d, J = 16.6 Hz, 1H), 4.89 (d, J = 59.7 Hz, 1H), 4.41 (s, 2H), 3.93 (d, J = 164.6 Hz, 1H), 3.51 (s, 2H), 2.92 (d, J = 85.8 Hz, 2H), 2.37 (s, 3H), 2.19 (s, 3H), 1.79 (d, J = 30.9 Hz, 2H), 1.39 (d, J = 37.9 Hz, 2H). ^{13}C NMR (101 MHz, DMSO-d_6) δ 169.16, 165.90, 159.83, 158.46, 145.39, 139.62, 137.68, 133.96, 132.03, 130.47, 129.63, 128.39, 126.83, 126.57, 126.37, 126.01, 125.65, 125.34, 115.74, 65.40, 54.23, 49.19, 47.66, 42.16, 37.53, 33.33, 33.02, 23.63, 22.16, 11.81, 10.86. HRMS (ESI): m/z calcd for $C_{26}H_{27}N_4O_4$ $[M+H]^+$ 459.1954, found 459.2023.

(R)-4-(3-(3,5-dimethylisoxazol-4-yl)-5-(3-hydroxypiperidine-1-carbonyl)benzyl)phthalazin-1(2H)-one (DDT20)

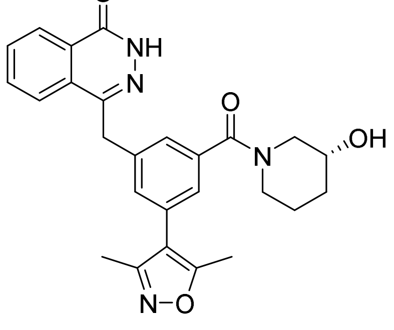

White solid, yield: 74.2%; mp: 143-144 °C; ^{1}H NMR (400 MHz, DMSO-d_6) δ 12.60 (s, 1H), 8.28 (d, J = 7.8 Hz, 1H), 8.07 (d, J = 7.8 Hz, 1H), 7.91 (t, J = 7.2 Hz, 1H), 7.84 (t, J = 7.5 Hz, 1H), 7.44 (s, 1H), 7.32 (d, J = 21.7 Hz, 1H), 7.23 (d, J = 16.6 Hz, 1H), 4.89 (d, J = 59.7 Hz, 1H), 4.41 (s, 2H), 3.95 (d, J = 168.8 Hz, 1H), 3.51 (s, 2H), 2.91 (d, J = 74.1 Hz, 2H), 2.37 (s, 3H), 2.19 (s, 3H), 1.79 (d, J = 29.5 Hz, 2H), 1.40 (d, J = 24.8 Hz, 2H). ^{13}C NMR (101 MHz, DMSO-d_6) δ 169.19, 165.90, 159.83, 158.46, 145.39, 139.61, 137.68, 133.96, 132.03, 130.47, 129.63, 128.39, 126.83, 126.57, 126.39, 126.01, 125.66, 115.74, 93.34, 65.40, 54.23, 49.17, 47.62, 42.22, 37.53, 33.38, 33.00, 23.58, 22.16, 11.81, 10.86. HRMS (ESI): m/z calcd for $C_{26}H_{27}N_4O_4$ $[M+H]^+$ 459.1954, found 459.2022.

4-(3-(3,5-dimethylisoxazol-4-yl)-5-(4-hydroxypiperidine-1-carbonyl)benzyl)phthalazin-1(2H)-one (DDT21)

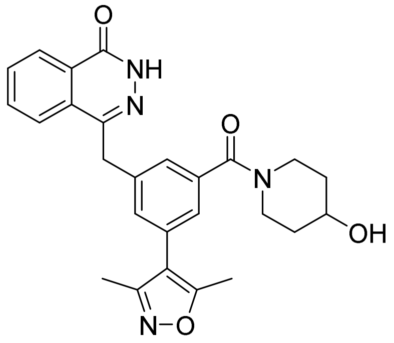

Yellow solid, yield: 11.5%; mp: 176-177 °C; ^{1}H NMR (400 MHz, DMSO-d_6) δ 12.60 (s, 1H), 8.27 (d, J = 7.6 Hz, 1H), 8.06 (d, J = 7.8 Hz, 1H), 7.90 (t, J = 7.3 Hz, 1H), 7.83 (t, J = 7.3 Hz, 1H), 7.45 (s, 1H), 7.30 (s, 1H), 7.20 (s, 1H), 4.78 (s, 1H), 4.41 (s, 2H), 3.97 (s, 1H), 3.72 (s, 1H), 3.44 (s, 1H), 3.24 – 3.01 (m, 2H), 2.36 (s, 3H), 2.19 (s, 3H), 1.82 – 1.57 (m, 2H), 1.38 – 1.12 (m, 2H). ^{13}C NMR (101 MHz, DMSO-d_6) δ 168.75, 165.90, 159.82, 158.45, 145.41, 139.65, 137.58, 133.93, 132.04, 130.59, 130.56, 129.62, 128.38, 126.57, 126.38, 126.01, 125.31, 115.72, 65.86, 45.03, 37.50, 34.54, 11.81, 10.86. HRMS (ESI): m/z calcd for $C_{26}H_{27}N_4O_4$ $[M+H]^+$ 459.1954, found 459.2020.

3-(3,5-dimethylisoxazol-4-yl)-5-nitrobenzaldehyde (compound 16)

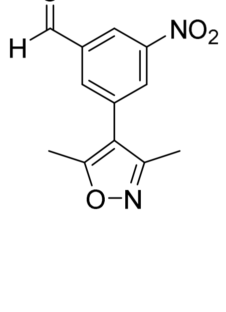

Yellow solid, yield: 60%; mp: 125-126 °C; ^{1}H NMR (400 MHz, DMSO-d_6) δ 10.19 (s, 1H), 8.69 – 8.63 (m, 1H), 8.49 (t, J = 2.0 Hz, 1H), 8.35 (t, J = 1.5 Hz, 1H), 2.49 (s, 3H), 2.30 (s, 3H). ^{13}C NMR (101 MHz, DMSO-d_6) δ 192.04, 167.32, 158.48, 149.28, 138.17, 135.97, 133.30, 128.74, 122.68, 114.21, 11.82, 10.76. HRMS (ESI): m/z calcd for $C_{12}H_{11}N_2O_4$ [M+H]$^+$ 247.0641, found 247.0713.

4-(3-(3,5-dimethylisoxazol-4-yl)-5-nitrobenzyl)phthalazin-1(2H)-one (compound 18)

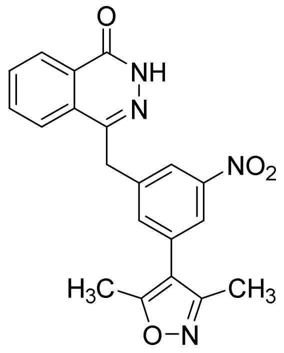

White solid, yield: 51.2%; mp: 243-244 °C; ^{1}H NMR (400 MHz, DMSO-d_6) δ 12.62 (s, 1H), 8.29 (d, J = 7.5 Hz, 1H), 8.22 (t, J = 1.9 Hz, 1H), 8.12 (d, J = 8.0 Hz, 1H), 8.07 (t, J = 1.9 Hz, 1H), 7.95 (t, J = 7.2 Hz, 1H), 7.88 (s, 1H), 7.85 (d, J = 6.9 Hz, 1H), 4.56 (s, 2H), 2.42 (s, 3H), 2.23 (s, 3H). ^{13}C NMR (101 MHz, DMSO-d_6) δ 166.75, 159.81, 158.43, 148.81, 145.01, 141.39, 136.51, 134.05, 132.15, 132.04, 129.57, 128.33, 126.57, 125.81, 123.08, 121.93, 114.73, 37.03, 11.80, 10.78. HRMS (ESI): m/z calcd for $C_{20}H_{17}N_4O_4$ [M+H]$^+$ 377.1172, found 377.1247.

4-(3-amino-5-(3,5-dimethylisoxazol-4-yl)benzyl)phthalazin-1(2H)-one (compound 19)

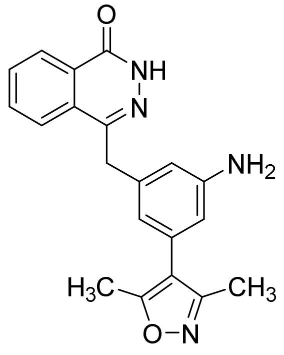

Yellow solid, yield: 25.6%; mp: >250 °C; ^{1}H NMR (400 MHz, DMSO-d_6) δ 12.58 (s, 1H), 8.26 (d, J = 7.6 Hz, 1H), 7.97 (d, J = 7.8 Hz, 1H), 7.90 – 7.84 (m, 1H), 7.81 (t, J = 7.4 Hz, 1H), 6.50 (t, J =

1.5 Hz, 1H), 6.43 (t, J = 1.8 Hz, 1H), 6.37 (t, J = 1.8 Hz, 1H), 5.15 (s, 2H), 4.18 (s, 2H), 2.33 (s, 3H), 2.15 (s, 3H). ^{13}C NMR (101 MHz, DMSO-d_6) δ 165.01, 159.94, 158.46, 149.68, 145.83, 139.92, 133.78, 131.88, 130.89, 129.76, 128.38, 126.44, 126.26, 117.26, 116.78, 113.22, 112.90, 38.29, 11.78, 10.98. HRMS (ESI): m/z calcd for $C_{20}H_{19}N_4O_2$ $[M+H]^+$ 347.1430, found 347.1505.

N-(3-(3,5-dimethylisoxazol-4-yl)-5-((4-oxo-3,4-dihydrophthalazin-1-yl)methyl)phenyl)benzamide (DDT22)

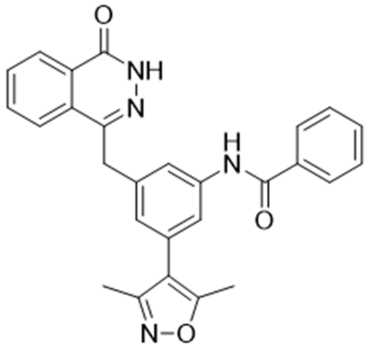

Yellow solid, yield:39%; mp:173-174℃; ^{1}H NMR (400 MHz, DMSO-d_6) δ 12.64 (s, 1H), 10.28 (s, 1H), 8.28 (d, J = 7.6 Hz, 1H), 8.02 (d, J = 7.9 Hz, 1H), 7.93 (d, J = 7.3 Hz, 1H), 7.88 (t, J = 7.5 Hz, 1H), 7.82 (t, J = 7.4 Hz, 1H), 7.72 (s, 1H), 7.71 (s, 1H), 7.64 – 7.56 (m, 1H), 7.54 (d, J = 11.7 Hz, 1H), 7.51 (d, J = 1.8 Hz, 1H), 7.50 – 7.47 (m, 1H), 7.18 (d, J = 1.6 Hz, 1H), 4.38 (s, 2H), 2.41 (s, 3H), 2.24 (s, 3H). ^{13}C NMR (101 MHz, DMSO-d_6) δ 166.17, 165.59, 159.93, 158.45, 145.47, 140.27, 139.89, 135.30, 133.86, 133.26, 132.07, 131.93, 130.69, 129.72, 129.70, 128.99, 128.81, 128.45, 128.12, 126.53, 126.12, 124.95, 38.22, 11.82, 10.98. HRMS(ESI):m/z calcd for $C_{27}H_{21}N_4O_3$$[M-H]^-$ 449.1692, found 449.1630.

N-(3-(3,5-dimethylisoxazol-4-yl)-5-((4-oxo-3,4-dihydrophthalazin-1-yl)methyl)phenyl)-2-methylbenzamide (DDT23)

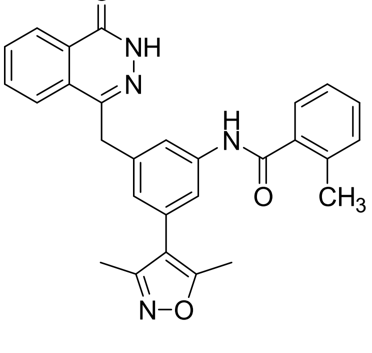

White solid,yield:53.8%; mp:>250°C;^{1}H NMR (400 MHz, DMSO-d_6) δ 12.62 (s, 1H), 10.31 (s, 1H), 8.28 (d, J = 7.6 Hz, 1H), 8.02 (d, J = 7.9 Hz, 1H), 7.89 (t, J = 7.1 Hz, 1H), 7.83 (t, J = 7.4 Hz, 1H), 7.71 (s, 1H), 7.63 (s, 1H), 7.43 (d, J = 7.4 Hz, 1H), 7.38 (t, J = 7.3 Hz, 1H), 7.29 (s, 1H), 7.27 (d, J = 8.0 Hz, 1H), 7.15 (s, 1H), 4.37 (s, 2H), 2.39 (s, 3H), 2.36 (s, 3H), 2.22 (s, 3H). ^{13}C NMR (101 MHz, DMSO-d_6) δ 168.39, 165.60, 159.89, 158.43, 145.47, 140.37, 140.00, 137.48, 135.72, 133.90, 131.97, 130.97, 130.72, 130.14, 129.69, 128.42, 127.69, 126.52, 126.16, 126.06, 124.77, 118.94, 118.50, 116.13, 38.14, 19.77, 11.82, 10.98. HRMS (ESI):m/z calcd for $C_{28}H_{23}N_4O_3$ [M-H]$^-$ 463.1848, found 463.1783.

N-(3-(3,5-dimethylisoxazol-4-yl)-5-((4-oxo-3,4-dihydrophthalazin-1-yl)methyl)phenyl)-3-methylbenzamide (DDT24)

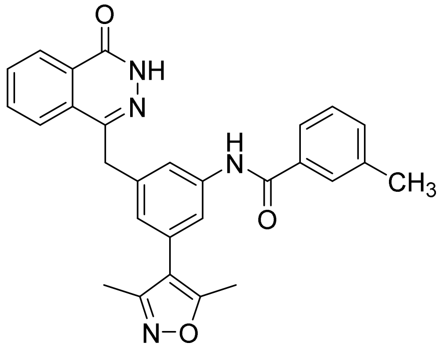

White solid,yield:65%; mp:>250°C;^{1}H NMR (400 MHz, DMSO-d_6) δ 12.65 (s, 1H), 10.24 (s, 1H), 8.28 (d, J = 8.9 Hz, 1H), 8.02 (d, J = 7.9 Hz, 1H), 7.93 – 7.86 (m, 1H), 7.83 (t, J = 7.5 Hz, 1H), 7.73 (s, 1H), 7.72 (s, 1H), 7.70 – 7.69 (m, 2H), 7.39 (d, J = 5.1 Hz, 2H), 7.18 (s, 1H), 4.37 (s, 2H), 2.41 (s, 3H), 2.39 (s, 3H), 2.24 (s, 3H). ^{13}C ^{13}C NMR (101 MHz, DMSO-d_6) δ 166.25, 165.61, 159.92, 158.47, 145.49, 140.29, 139.88, 138.16, 135.27, 133.91, 132.67, 131.98, 130.64, 129.68, 128.73, 128.58, 128.42, 126.53, 126.16, 125.29, 124.90, 119.58, 119.22, 116.17, 38.20, 21.41, 11.84, 11.01.HRMS(ESI):m/z calcd for $C_{28}H_{23}N_4O_3$ [M-H]$^-$ 463.1848, found 463.1784.

N-(3-(3,5-dimethylisoxazol-4-yl)-5-((4-oxo-3,4-dihydrophthalazin-1-yl)methyl)phenyl)-4-methylbenzamide (DDT25)

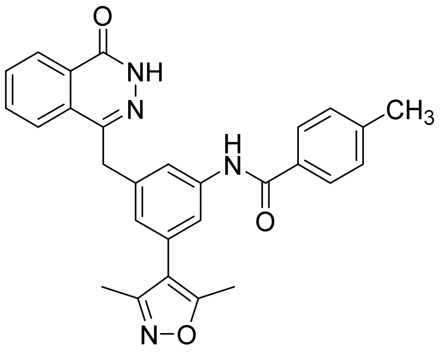

White solid,yield:69%;mp:>250°C;^{1}H NMR (400 MHz, DMSO-d_6) δ 12.64 (s, 1H), 10.19 (s, 1H), 8.28 (d, J = 7.2 Hz, 1H), 8.02 (d, J = 7.9 Hz, 1H), 7.89 (t, J = 7.7 Hz, 1H), 7.83 (m, J = 11.4, 7.8 Hz, 3H), 7.70 (s, 2H), 7.33 (s, 1H), 7.31 (s, 1H), 7.17 (s, 1H), 4.37 (s, 2H), 2.41 (s, 3H), 2.38 (s, 3H), 2.24 (s, 3H). ^{13}C NMR (101 MHz, DMSO-d_6) δ 165.95, 165.59, 159.92, 158.46, 145.49, 142.14, 140.33, 139.86, 133.89, 132.38, 131.96, 130.63, 129.68, 129.35, 128.43, 128.16, 126.53, 126.15, 124.84, 119.61, 119.25, 116.19, 38.21, 21.46, 11.83, 10.99. HRMS(ESI):m/z calcd for $C_{28}H_{23}N_4O_3$ $[M-H]^-$ 463.1848, found 463.1786.

N-(3-(3,5-dimethylisoxazol-4-yl)-5-((4-oxo-3,4-dihydrophthalazin-1-yl)methyl)phenyl)-2-(trifluoromethyl)benzamide (DDT26)

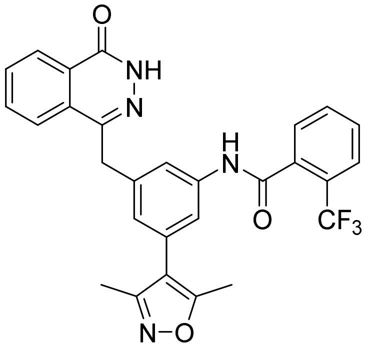

White solid, yield: 53.3%; mp:210-211°C; The purity of DDT26 was > 99.0% as determined by HPLC; ^{1}H NMR (400 MHz, DMSO-d6) δ 12.62 (s, 1H), 10.58 (s, 1H), 8.27 (d, J = 7.6 Hz, 1H), 8.03 (d, J = 7.9 Hz, 1H), 7.90 (t, J = 7.1 Hz, 1H), 7.85 (s, 1H), 7.82 (d, J = 6.3 Hz, 1H), 7.78 (d, J = 7.3 Hz, 1H), 7.70 (s, 1H), 7.68 (d, J = 7.3 Hz, 1H), 7.60 (s, 1H), 7.58 (s, 1H), 7.18 (s, 1H), 4.37 (s, 2H), 2.39 (s, 3H), 2.21 (s, 3H). ^{13}C NMR (101 MHz, DMSO-d_6) δ 166.11, 165.66, 159.88, 158.41, 145.45, 140.15, 139.97, 136.47, 133.92, 133.07, 132.00, 130.85, 130.58, 129.68, 129.02, 128.40, 126.78 (d, J = 4.6

Hz), 126.53, 126.25 (d, *J* = 31.5 Hz), 126.15, 125.18, 124.21 (d, *J* = 273.7 Hz), 118.96, 118.45, 116.04, 38.03, 11.81, 10.96. HRMS(ESI):m/z calcd for $C_{28}H_{20}F_3N_4O_3$ $[M-H]^-$ 517.1566, found 517.1496.

N-(3-(3,5-dimethylisoxazol-4-yl)-5-((4-oxo-3,4-dihydrophthalazin-1-yl)methyl)phenyl)-3-(trifluoromethyl)benzamide (DDT27)

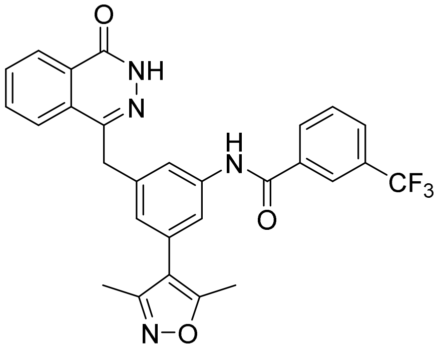

White solid, yield:66.7%; mp:231-232℃; ^{1}H NMR (400 MHz, DMSO-d6) δ 12.65 (s, 1H), 10.50 (s, 1H), 8.29 (d, J = 7.7 Hz, 1H), 8.26 (s, 1H), 8.24 (d, J = 8.1 Hz, 1H), 8.03 (d, J = 7.9 Hz, 1H), 7.96 (d, J = 7.7 Hz, 1H), 7.90 (t, J = 8.1 Hz, 1H), 7.83 (t, J = 7.5 Hz, 1H), 7.78 (t, J = 7.8 Hz, 1H), 7.72 (s, 1H), 7.66 (s, 1H), 7.23 (s, 1H), 4.39 (s, 2H), 2.42 (s, 3H), 2.25 (s, 3H). ^{13}C NMR (101 MHz, DMSO-*d*$_6$) δ 165.66, 164.67, 159.93, 158.45, 145.46, 139.99, 139.89, 136.14, 133.90, 132.31, 131.98, 130.76, 130.17, 129.67, 129.64 (d, *J* = 32.0 Hz), 128.65 (d, *J* = 3.8 Hz), 128.44, 126.54, 126.14, 125.38, 124.74 (q, *J* = 3.9 Hz), 124.42 (d, *J* = 272.7 Hz), 119.79, 119.49, 116.13, 38.16, 11.83, 10.98. HRMS(ESI):m/z calcd for $C_{28}H_{20}F_3N_4O_3$ $[M-H]^-$ 517.1566, found 517.1497.

N-(3-(3,5-dimethylisoxazol-4-yl)-5-((4-oxo-3,4-dihydrophthalazin-1-yl)methyl)phenyl)-4-(trifluoromethyl)benzamide (DDT28)

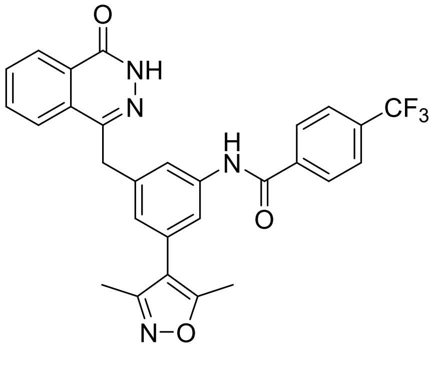

White solid, yield:73%; mp:>250°C; ^{1}H NMR (400 MHz, DMSO-d6) δ 12.65 (s, 1H), 10.50 (s, 1H), 8.28 (d, J = 7.0 Hz, 1H), 8.13 (s, 1H), 8.11 (s, 1H), 8.03 (d, J = 7.9 Hz, 1H), 7.90 (d, J = 8.0 Hz, 3H), 7.83 (t, J = 7.4 Hz, 1H), 7.71 (s, 1H), 7.69 (s, 1H), 7.23 (s, 1H), 4.39 (s, 2H), 2.42 (s, 3H), 2.24 (s, 3H). ^{13}C NMR (101 MHz, DMSO-d_6) δ 165.66, 164.99, 159.92, 158.45, 145.46, 140.01, 139.91, 139.09, 133.90, 131.98, 131.88 (d, J = 31.9 Hz), 130.76, 129.67, 129.07, 128.43, 126.53, 126.14, 125.83 (q, J = 3.7 Hz), 125.34, 124.37 (d, J = 272.4 Hz), 119.68, 119.33, 116.12. HRMS(ESI):m/z calcd for $C_{28}H_{20}F_3N_4O_3$ [M-H]$^-$ 517.1566, found 517.1497.

N-(3-(3,5-dimethylisoxazol-4-yl)-5-((4-oxo-3,4-dihydrophthalazin-1-yl)methyl)phenyl)-3-nitrobenzamide (DDT29)

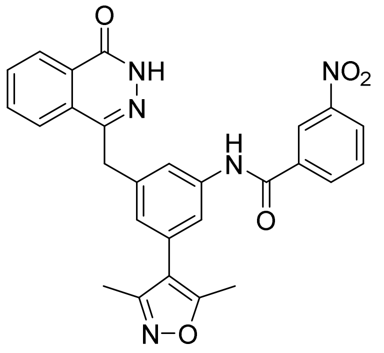

White solid,yield:79.5%;mp:>250°C; ^{1}H NMR (400 MHz, DMSO-d_6) δ 12.66 (s, 1H), 10.62 (s, 1H), 8.76 (s, 1H), 8.44 (d, J = 8.2 Hz, 1H), 8.37 (d, J = 7.8 Hz, 1H), 8.28 (d, J = 7.2 Hz, 1H), 8.04 (d, J = 7.9 Hz, 1H), 7.90 (t, J = 8.1 Hz, 1H), 7.83 (t, J = 7.9 Hz, 2H), 7.73 (s, 1H), 7.67 (s, 1H), 7.25 (s, 1H), 4.40 (s, 2H), 2.43 (s, 3H), 2.25 (s, 3H). ^{13}C NMR (101 MHz, DMSO-d_6) δ 165.68, 163.96, 159.93, 158.46, 148.19, 145.47, 140.03, 139.77, 136.61, 134.67, 133.92, 132.00, 130.78, 130.65, 129.66, 128.42, 126.70, 126.54, 126.15, 125.49, 122.89, 119.82, 119.49, 116.11, 38.14, 11.84, 11.00. HRMS(ESI): m/z calcd for $C_{27}H_{20}N_5O_5$ [M-H]$^-$ 494.1543, found 494.1478.

N-(3-(3,5-dimethylisoxazol-4-yl)-5-((4-oxo-3,4-dihydrophthalazin-1-yl)methyl)phenyl)-4-nitrobenzamide (DDT30)

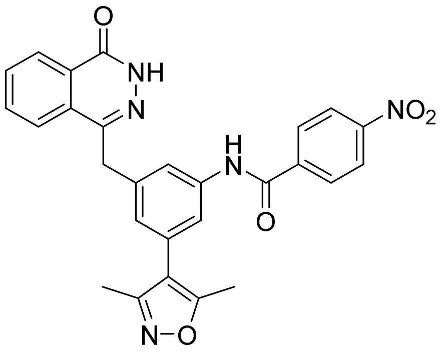

Yellow solid, yield:68%; mp:>250°C; ^{1}H NMR (400 MHz, DMSO-d_6) δ 12.65 (s, 1H), 10.59 (s, 1H), 8.35 (d, J = 7.6 Hz, 2H), 8.28 (d, J = 6.7 Hz, 1H), 8.15 (d, J = 7.8 Hz, 2H), 8.02 (d, J = 7.3 Hz, 1H), 7.92 – 7.82 (m, 2H), 7.69 (d, J = 15.1 Hz, 2H), 7.24 (s, 1H), 4.39 (s, 2H), 2.42 (s, 3H), 2.24 (s, 3H). ^{13}C NMR (101 MHz, DMSO-d_6) δ 165.68, 164.50, 159.92, 158.45, 149.66, 145.45, 140.92, 140.06, 139.79, 133.92, 132.00, 130.79, 129.69, 129.66, 128.43, 126.54, 126.14, 125.50, 123.99, 119.71, 119.37, 116.09, 38.14, 11.84, 10.99. HRMS(ESI): m/z calcd for $C_{27}H_{20}N_5O_5$ [M-H]$^-$ 494.1543, found 494.1477.

3-amino-N-(3-(3,5-dimethylisoxazol-4-yl)-5-((4-oxo-3,4-dihydrophthalazin-1-yl)methyl)phenyl)benzamide (DDT31)

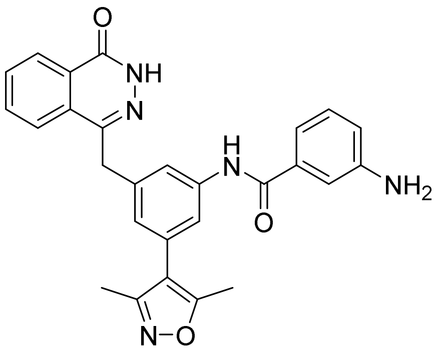

White solid, yield:86%; mp:236-237°C;^{1}H NMR (600 MHz, DMSO-d_6) δ 12.64 (s, 1H), 10.21 (s, 1H), 8.27 (d, J = 7.3 Hz, 1H), 8.01 (d, J = 7.4 Hz, 1H), 7.91 – 7.86 (m, 1H), 7.85 – 7.80 (m, 1H), 7.68 (s, 2H), 7.28 (s, 2H), 7.16 (s, 2H), 6.97 (s, 1H), 4.36 (s, 2H), 3.41 (s, 2H), 2.40 (s, 3H), 2.23 (s, 3H). ^{13}C NMR (151 MHz, DMSO-d_6) δ kk165.34, 164.53, 158.84, 157.39, 144.43, 139.24, 138.79, 135.28, 132.84, 130.91, 129.53, 128.60, 128.44, 127.34, 125.45, 125.10, 123.74, 118.46, 118.07, 115.10, 37.10, 10.77, 9.93. HRMS(ESI): m/z calcd for $C_{27}H_{22}N_5O_3$ [M-H]$^-$ 464.1801, found 464.1734.

4-amino-N-(3-(3,5-dimethylisoxazol-4-yl)-5-((4-oxo-3,4-dihydrophthalazin-1-yl)methyl)phenyl)benzamide (DDT32)

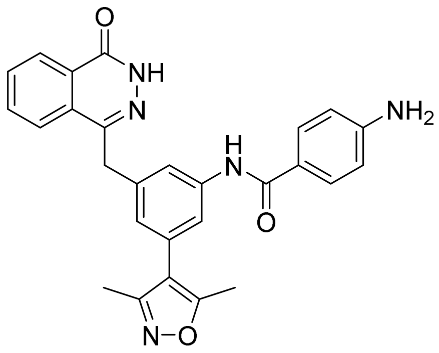

White solid,yield:50%;mp:188-189℃;^{1}H NMR (400 MHz, DMSO-d_6) δ 12.63 (s, 1H), 9.79 (s, 1H), 8.28 (d, J = 7.7 Hz, 1H), 8.01 (d, J = 7.9 Hz, 1H), 7.89 (t, J = 7.6 Hz, 1H), 7.82 (t, J = 7.5 Hz, 1H), 7.70 (s, 1H), 7.68 (s, 1H), 7.67 (d, J = 3.1 Hz, 1H), 7.66 (s, 1H), 7.10 (s, 1H), 6.60 (s, 1H), 6.58 (s, 1H), 5.76 (s, 2H), F4.35 (s, 2H), 3.34 (s, 2H), 2.40 (s, 3H), 2.23 (s, 3H). ^{13}C NMR (101 MHz, DMSO-d_6) δ 165.86, 165.52, 159.92, 158.47, 152.70, 145.53, 140.86, 139.69, 133.88, 131.95, 130.48, 129.85, 129.70, 128.42, 126.52, 126.17, 124.16, 121.35, 119.39, 119.04, 116.28, 113.00, 38.27, 11.83, 11.00. HRMS(ESI): m/z calcd for $C_{27}H_{22}N_5O_3$ $[M-H]^-$ 464.1801, found 464.1736.

4-(3-(benzylamino)-5-(3,5-dimethylisoxazol-4-yl)benzyl)phthalazin-1(2H)-one (DDT33)

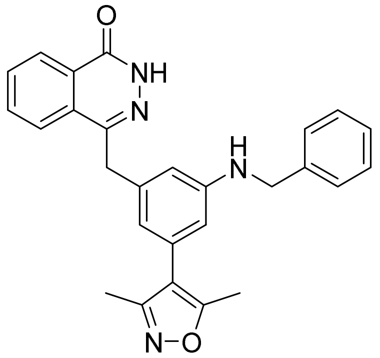

Yellow solid, yield: 38%; mp: 230-231 °C; ^{1}H NMR (400 MHz, DMSO-d_6) δ 12.58 (s, 1H), 8.26 (d, J = 9.1 Hz, 1H), 7.95 (d, J = 8.6 Hz, 1H), 7.88 – 7.74 (m, 2H), 7.34 – 7.24 (m, 4H), 7.19 (t, J = 6.8 Hz, 1H), 6.52 (d, J = 9.8 Hz, 2H), 6.42 (s, 1H), 6.28 (s, 1H), 4.21 (s, 2H), 4.19 (s, 2H), 2.21 (s, 3H), 2.04 (s, 3H). ^{13}C NMR (101 MHz, DMSO-d_6) δ 165.06, 159.90, 158.41, 149.47, 145.74, 140.45, 139.81, 133.76, 131.84, 130.81, 129.76, 128.72, 128.39, 127.68, 127.08, 126.44, 126.21, 117.23, 116.79,

112.31, 110.96, 46.93, 38.26, 11.65, 10.83. HRMS (ESI): m/z calcd for $C_{27}H_{25}N_4O_2$ $[M+H]^+$ 437.1899, found 437.1962.

4-(3-(3,5-dimethylisoxazol-4-yl)-5-((3-fluorobenzyl)amino)benzyl)phthalazin-1(2H)-one (DDT34)

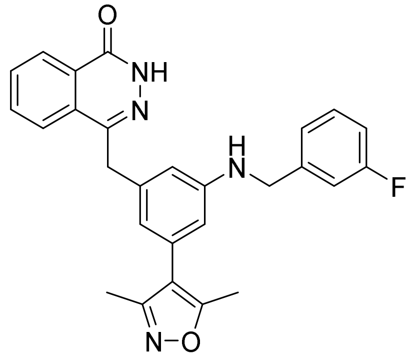

Yellow solid, yield: 64%; mp: 202-203 °C; ^{1}H NMR (400 MHz, DMSO-d_6) δ 12.58 (s, 1H), 8.27 (d, J = 6.1 Hz, 1H), 7.95 (d, J = 8.8 Hz, 1H), 7.89 – 7.76 (m, 2H), 7.36 – 7.29 (m, 1H), 7.14 (dd, J = 12.5, 9.3 Hz, 2H), 7.02 (t, J = 8.7 Hz, 1H), 6.52 (s, 2H), 6.48 (t, J = 6.0 Hz, 1H), 6.28 (s, 1H), 4.25 (d, J = 5.8 Hz, 2H), 4.20 (s, 2H), 2.22 (s, 3H), 2.05 (s, 3H). ^{13}C NMR (101 MHz, DMSO-d_6) δ 165.08, 162.80 (d, J = 243.3 Hz), 159.89, 158.40, 149.25, 145.71, 143.83 (d, J = 6.7 Hz), 139.90, 133.73, 131.84, 130.87, 130.64 (d, J = 8.2 Hz), 129.74, 128.38, 126.44, 126.18, 123.59 (d, J = 2.6 Hz), 117.46, 116.76, 114.14 (d, J = 21.4 Hz), 113.81 (d, J = 21.1 Hz), 112.46, 110.90, 46.33, 38.24, 11.63, 10.79. HRMS (ESI): m/z calcd for $C_{27}H_{24}FN_4O_2$ $[M+H]^+$ 455.1805, found 455.1876.

4-(3-(3,5-dimethylisoxazol-4-yl)-5-((4-fluorobenzyl)amino)benzyl)phthalazin-1(2H)-one (DDT35)

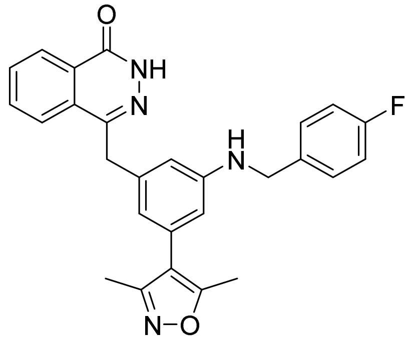

Yield: 52%; mp: 228-229 °C; ^{1}H NMR (400 MHz, DMSO-d_6) δ 12.58 (s, 1H), 8.26 (d, *J* = 7.9 Hz, 1H), 7.94 (d, *J* = 8.5 Hz, 1H), 7.87 – 7.83 (m, 1H), 7.82 – 7.79 (m, 1H), 7.36 – 7.29 (m, 2H), 7.09 (t, *J* = 8.9 Hz, 2H), 6.50 (d, *J* = 7.7 Hz, 2H), 6.42 (t, *J* = 5.9 Hz, 1H), 6.27 (s, 1H), 4.19 (d, *J* = 5.8 Hz, 4H), 2.22 (s, 3H), 2.05 (s, 3H). ^{13}C NMR (101 MHz, DMSO-d_6) δ 165.08, 161.50 (d, *J* = 241.9 Hz), 159.90, 158.41, 149.31, 145.74, 139.85, 136.56 (d, *J* = 2.8 Hz), 133.76, 131.86, 130.83, 129.74, 129.50 (d, *J* = 8.1 Hz), 128.37, 126.44, 126.21, 117.32, 116.77, 115.41 (d, *J* = 21.2 Hz), 112.27, 110.93, 46.08, 38.22, 11.66, 10.83. HRMS (ESI): m/z calcd for $C_{27}H_{24}FN_4O_2$ [M+H]$^+$ 455.1805, found 455.1877.

4-(3-(3,5-dimethylisoxazol-4-yl)-5-((3-nitrobenzyl)amino)benzyl)phthalazin-1(2H)-one (DDT36)

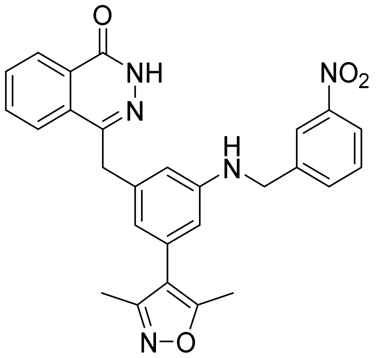

Yellow solid, yield: 46%; mp: 229-230 °C; ^{1}H NMR (400 MHz, DMSO-d_6) δ 12.56 (s, 1H), 8.25 (d, *J* = 6.8 Hz, 1H), 8.17 (s, 1H), 8.06 (d, *J* = 8.1 Hz, 1H), 7.92 (d, *J* = 8.3 Hz, 1H), 7.81 (m, *J* = 6.7, 4.1, 1.7 Hz, 2H), 7.76 (d, *J* = 7.9 Hz, 1H), 7.58 (t, *J* = 7.9 Hz, 1H), 6.61 (t, *J* = 6.0 Hz, 1H), 6.54 (s, 1H), 6.50 (s, 1H), 6.31 (s, 1H), 4.38 (d, *J* = 6.0 Hz, 2H), 4.19 (s, 2H), 2.21 (s, 3H), 2.04 (s, 3H). ^{13}C NMR (101 MHz, DMSO-d_6) δ 165.11, 159.87, 158.40, 149.01, 148.32, 145.68, 143.33, 140.01, 134.31, 133.71, 131.84, 130.97, 130.21, 129.70, 128.34, 126.42, 126.16, 122.11, 122.03, 117.70, 116.71, 112.50, 110.92, 45.99, 38.19, 11.64, 10.80. HRMS (ESI): m/z calcd for $C_{27}H_{24}N_5O_4$ [M+H]$^+$ 482.1750, found 482.1825.

4-(3-(3,5-dimethylisoxazol-4-yl)-5-((4-nitrobenzyl)amino)benzyl)phthalazin-1(2H)-one (DDT37)

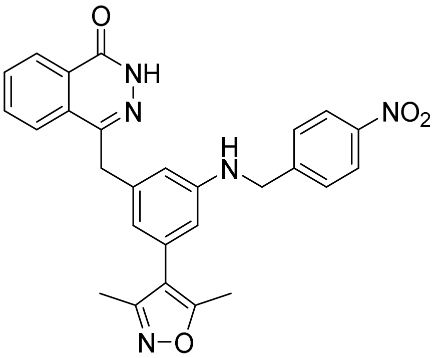

Yellow solid, yield: 51.8%; mp: 232-233 °C; ^{1}H NMR (400 MHz, DMSO-d_6) δ 12.56 (s, 1H), 8.23 (d, J = 7.4 Hz, 1H), 8.13 (d, J = 8.7 Hz, 2H), 7.91 (d, J = 7.4 Hz, 1H), 7.84 – 7.80 (m, 1H), 7.80 – 7.76 (m, 1H), 7.53 (d, J = 8.6 Hz, 2H), 6.62 (t, J = 6.0 Hz, 1H), 6.55 (s, 1H), 6.44 (s, 1H), 6.28 (s, 1H), 4.37 (d, J = 6.0 Hz, 2H), 4.18 (s, 2H), 2.23 (s, 3H), 2.05 (s, 3H). ^{13}C NMR (101 MHz, DMSO-d_6) δ 165.13, 159.86, 158.40, 149.14, 148.99, 146.78, 145.66, 140.02, 133.73, 131.80, 130.95, 129.68, 128.48, 128.34, 126.38, 126.16, 123.90, 117.65, 116.68, 112.21, 111.00, 46.31, 38.15, 11.67, 10.84. HRMS (ESI): m/z calcd for $C_{27}H_{24}N_5O_4$ [M+H]$^+$ 482.1750, found 482.1830.

4-(3-((3-chlorobenzyl)amino)-5-(3,5-dimethylisoxazol-4-yl)benzyl)phthalazin-1(2H)-one (DDT38)

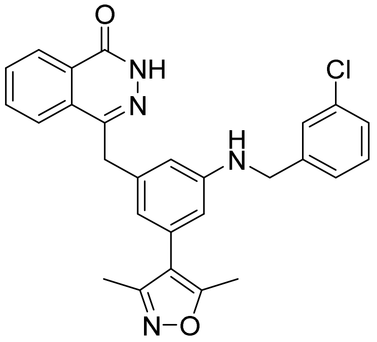

White solid, yield: 50%; mp: 235-236 °C; ^{1}H NMR (400 MHz, DMSO-d_6) δ 12.57 (s, 1H), 8.26 (d, J = 9.1 Hz, 1H), 7.95 (d, J = 8.7 Hz, 1H), 7.86 – 7.82 (m, 1H), 7.82 – 7.79 (m, 1H), 7.37 (s, 1H), 7.34 – 7.29 (m, 1H), 7.26 (t, J = 7.7 Hz, 2H), 6.52 (s, 2H), 6.48 (s, 1H), 6.27 (s, 1H), 4.24 (s, 2H), 4.19 (s, 2H), 2.21 (s, 3H), 2.04 (s, 3H). ^{13}C NMR (101 MHz, DMSO-d_6) δ 165.08, 159.89, 158.40, 149.19, 145.71, 143.39, 139.91, 133.75, 133.52, 131.86, 130.89, 130.59, 129.74, 128.38, 127.39, 127.03, 126.44, 126.31, 126.19, 117.50, 116.76, 112.53, 110.85, 46.22, 38.23, 11.64, 10.80. HRMS (ESI): m/z calcd for $C_{27}H_{24}ClN_4O_2$ [M+H]$^+$ 471.1510, found 471.1579.

4-(3-((4-chlorobenzyl)amino)-5-(3,5-dimethylisoxazol-4-yl)benzyl)phthalazin-1(2H)-one (DDT39)

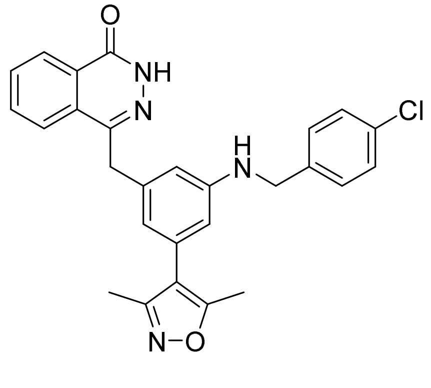

Yellow solid, yield: 59%; mp: 200-201 °C; ^{1}H NMR (400 MHz, DMSO-d_6) δ 12.58 (s, 1H), 8.34 – 8.21 (m, 1H), 7.98 – 7.91 (m, 1H), 7.88 – 7.77 (m, 2H), 7.32 (s, 4H), 6.51 (d, *J* = 7.5 Hz, 2H), 6.45 (t, *J* = 6.0 Hz, 1H), 6.27 (t, *J* = 1.8 Hz, 1H), 4.21 (d, *J* = 6.0 Hz, 2H), 4.19 (s, 2H), 2.23 (s, 3H), 2.05 (s, 3H). ^{13}C NMR (101 MHz, DMSO-d_6) δ 165.08, 159.90, 158.40, 149.25, 145.73, 139.88, 139.59, 133.75, 131.84, 131.57, 130.87, 129.74, 129.46, 128.65, 128.38, 126.45, 126.18, 117.40, 116.76, 112.29, 110.96, 46.13, 38.22, 11.65, 10.82. HRMS (ESI): m/z calcd for $C_{27}H_{24}ClN_4O_2$ [M+H]$^{+}$ 471.1510, found 471.1580.

4-(3-((3-bromobenzyl)amino)-5-(3,5-dimethylisoxazol-4-yl)benzyl)phthalazin-1(2H)-one (DDT40)

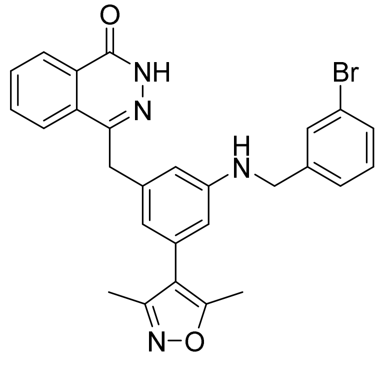

Yellow solid, yield: 53%; mp: 235-236 °C; ^{1}H NMR (400 MHz, DMSO-d_6) δ 12.57 (s, 1H), 8.26 (d, *J* = 9.1 Hz, 1H), 7.94 (d, *J* = 8.7 Hz, 1H), 7.88 – 7.74 (m, 2H), 7.52 (s, 1H), 7.39 (d, *J* = 7.8 Hz, 1H), 7.31 (d, *J* = 7.7 Hz, 1H), 7.24 (t, *J* = 7.7 Hz, 1H), 6.52 (d, *J* = 5.1 Hz, 2H), 6.47 (t, *J* = 6.0 Hz, 1H), 6.27 (s, 1H), 4.24 (d, *J* = 5.7 Hz, 2H), 4.19 (s, 2H), 2.21 (s, 3H), 2.04 (s, 3H). ^{13}C NMR (101 MHz,

DMSO-d_6) δ 165.09, 159.90, 158.41, 149.19, 145.72, 143.67, 139.90, 133.76, 131.86, 130.89, 130.30, 129.93, 129.74, 128.38, 126.70, 126.45, 126.18, 122.21, 117.51, 116.76, 112.56, 110.83, 46.18, 38.24, 11.64, 10.80. HRMS (ESI): m/z calcd for $C_{27}H_{24}BrN_4O_2$ $[M+H]^+$ 515.1004, found 515.1077.

4-(3-((4-bromobenzyl)amino)-5-(3,5-dimethylisoxazol-4-yl)benzyl)phthalazin-1(2H)-one (DDT41)

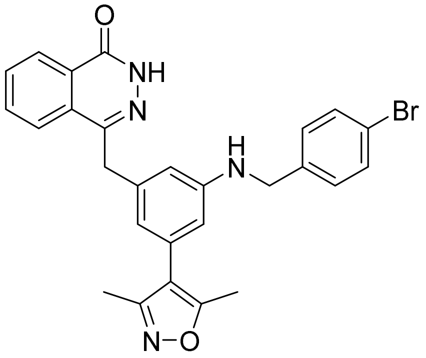

Yellow solid, yield: 56%; mp: 200-201 °C; ^{1}H NMR (400 MHz, DMSO-d_6) δ 12.57 (s, 1H), 8.26 (d, *J* = 7.1 Hz, 1H), 7.93 (d, *J* = 8.9 Hz, 1H), 7.87 – 7.77 (m, 2H), 7.46 (d, *J* = 8.4 Hz, 2H), 7.25 (d, *J* = 8.4 Hz, 2H), 6.51 (s, 1H), 6.49 (s, 1H), 6.45 (t, *J* = 6.0 Hz, 1H), 6.25 (s, 1H), 4.19 (s, 2H), 4.18 (s, 2H), 2.22 (s, 3H), 2.04 (s, 3H). ^{13}C NMR (101 MHz, DMSO-d_6) δ 165.08, 159.90, 158.40, 149.23, 145.73, 140.04, 139.88, 133.77, 131.85, 131.57, 130.86, 129.86, 129.73, 128.37, 126.45, 126.19, 120.01, 117.40, 116.75, 112.28, 110.96, 46.18, 38.21, 11.65, 10.82. HRMS (ESI): m/z calcd for $C_{27}H_{24}BrN_4O_2$ $[M+H]^+$ 515.1004, found 515.1079.

4-(3-((4-bromo-2-fluorobenzyl)amino)-5-(3,5-dimethylisoxazol-4-yl)benzyl)phthalazin-1(2H)-one (DDT42)

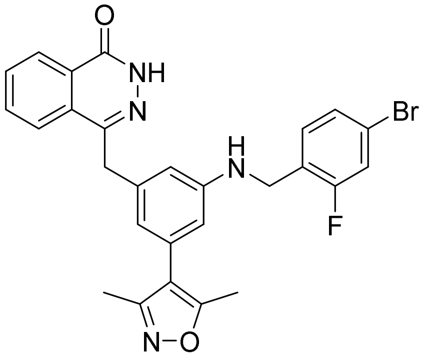

White solid, yield: 53%; mp: 230-231 °C; ^{1}H NMR (400 MHz, DMSO-d_6) δ 12.58 (s, 1H), 8.27 (d, J = 6.9 Hz, 1H), 7.92 (d, J = 8.7 Hz, 1H), 7.87 – 7.79 (m, 2H), 7.45 (d, J = 9.6 Hz, 1H), 7.34 – 7.18 (m, 2H), 6.54 (s, 1H), 6.48 (s, 1H), 6.39 (t, J = 6.0 Hz, 1H), 6.32 (s, 1H), 4.22 (d, J = 6.1 Hz, 2H), 4.20 (s, 2H), 2.26 (s, 3H), 2.09 (s, 3H). ^{13}C NMR (101 MHz, DMSO-d_6) δ 165.13, 160.62 (d, J = 248.8 Hz), 159.90, 158.41, 149.02, 145.68, 140.05, 133.74, 131.83, 131.50 (d, J = 5.3 Hz), 130.99, 129.72, 128.37, 127.86 (d, J = 3.5 Hz), 126.75, 126.60, 126.45, 126.16, 120.35 (d, J = 9.6 Hz), 118.91 (d, J = 25.1 Hz), 117.67, 116.72, 111.90, 110.85, 38.21, 11.69, 10.86. HRMS (ESI): m/z calcd for $C_{27}H_{23}BrFN_4O_2$ $[M+H]^+$ 533.0910, found 533.0984.

4-(3-((3-bromo-4-fluorobenzyl)amino)-5-(3,5-dimethylisoxazol-4-yl)benzyl)phthalazin-1(2H)-one (DDT43)

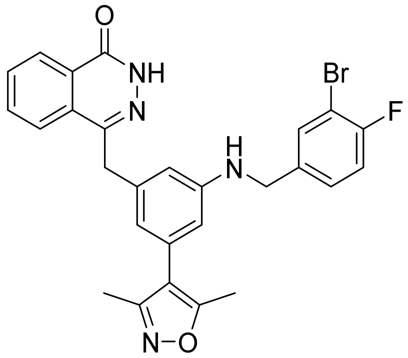

White solid, yield: 43.5%; mp: >250 °C; ^{1}H NMR (400 MHz, DMSO-d_6) δ 12.56 (s, 1H), 8.25 (d, J = 7.3 Hz, 1H), 7.93 (d, J = 7.7 Hz, 1H), 7.85 – 7.78 (m, 2H), 7.63 (d, J = 6.8 Hz, 1H), 7.36 – 7.33 (m, 1H), 7.27 (t, J = 8.6 Hz, 1H), 6.57 – 6.43 (m, 3H), 6.28 (s, 1H), 4.22 (s, 2H), 4.19 (s, 2H), 2.22 (s, 3H), 2.05 (s, 3H). ^{13}C NMR (101 MHz, DMSO-d_6) δ 165.10, 159.89, 158.41, 157.49 (d, J = 243.3 Hz), 149.06, 145.71, 139.94, 138.91 (d, J = 3.4 Hz), 133.74, 132.38, 131.85, 130.93, 129.73, 128.86 (d, J = 7.3 Hz), 128.37, 126.44, 126.18, 117.61, 116.92 (d, J = 22.1 Hz), 116.76, 112.56, 110.87, 108.23 (d, J = 21.1 Hz), 45.52, 38.21, 11.65, 10.81.HRMS (ESI): m/z calcd for $C_{27}H_{23}BrFN_4O_2$ $[M+H]^+$ 533.0910, found 533.0980.

4-(3-(3,5-dimethylisoxazol-4-yl)-5-((3-methylbenzyl)amino)benzyl)phthalazin-1(2H)-one (DDT44)

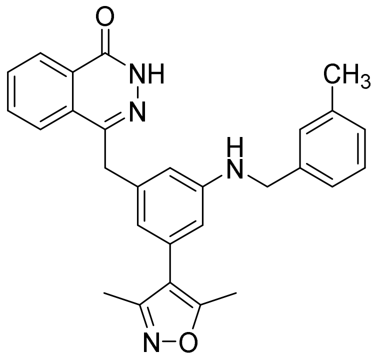

White solid, yield: 71%; mp: 206-207 °C; ^{1}H NMR (400 MHz, DMSO-d_6) δ 12.57 (s, 1H), 8.26 (d, J = 7.3 Hz, 1H), 7.95 (d, J = 7.5 Hz, 1H), 7.88 – 7.76 (m, 2H), 7.15 (dd, J = 13.8, 6.3 Hz, 2H), 7.08 (d, J = 7.5 Hz, 1H), 7.00 (d, J = 7.5 Hz, 1H), 6.52 (s, 1H), 6.50 (s, 1H), 6.37 (t, J = 5.8 Hz, 1H), 6.28 (s, 1H), 4.19 (s, 2H), 4.17 (d, J = 5.9 Hz, 2H), 2.24 (s, 3H), 2.22 (s, 3H), 2.05 (s, 3H). ^{13}C NMR (101 MHz, DMSO-d_6) δ 165.06, 159.89, 158.42, 149.56, 145.74, 140.41, 139.79, 137.77, 133.75, 131.85, 130.79, 129.76, 128.62, 128.38, 128.28, 127.75, 126.44, 126.22, 124.78, 117.19, 116.80, 112.34, 110.86, 46.94, 38.27, 21.49, 11.65, 10.82. HRMS (ESI): m/z calcd for $C_{28}H_{27}N_4O_2$ $[M+H]^+$ 451.2056, found 451.2129.

4-(3-(3,5-dimethylisoxazol-4-yl)-5-((4-methylbenzyl)amino)benzyl)phthalazin-1(2H)-one (DDT45)

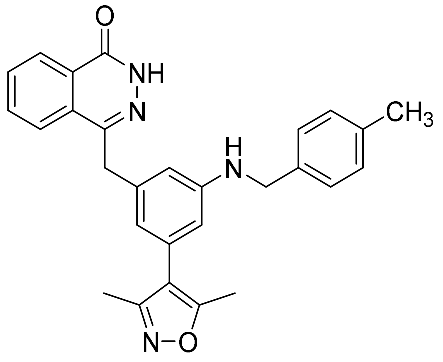

White solid, yield: 73%; mp: 215-216 °C; ^{1}H NMR (400 MHz, DMSO-d_6) δ 12.57 (s, 1H), 8.26 (d, J = 9.0 Hz, 1H), 7.94 (d, J = 8.5 Hz, 1H), 7.87 – 7.75 (m, 2H), 7.18 (s, 1H), 7.16 (s, 1H), 7.08 (s, 1H), 7.06 (s, 1H), 6.50 (s, 2H), 6.35 (t, J = 5.8 Hz, 1H), 6.28 (s, 1H), 4.18 (s, 2H), 4.15 (d, J = 5.8 Hz, 2H), 2.24 (d, J = 5.2 Hz, 3H), 2.23 (s, 3H), 2.06 (s, 3H). ^{13}C NMR (101 MHz, DMSO-d_6) δ 165.06, 159.90, 158.43, 149.52, 145.74, 139.78, 137.31, 136.07, 133.75, 131.83, 130.77, 129.75, 129.26,

128.38, 127.64, 126.42, 126.21, 117.14, 116.80, 112.18, 111.02, 46.67, 38.26, 21.11, 11.67, 10.85. HRMS (ESI): m/z calcd for $C_{28}H_{27}N_4O_2$ [M+H]$^+$ 451.2056, found 451.2128.

4-(3-(3,5-dimethylisoxazol-4-yl)-5-((4-(trifluoromethyl)benzyl)amino)benzyl)phthalazin-1(2H)-one (DDT46)

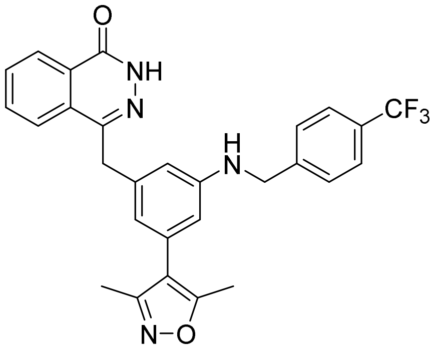

White solid, yield: 64.5%; mp: 220-221 °C; ^{1}H NMR (400 MHz, DMSO-d_6) δ 12.58 (s, 1H), 8.25 (d, J = 9.1 Hz, 1H), 7.94 (d, J = 7.3 Hz, 1H), 7.85 – 7.77 (m, 2H), 7.64 (d, J = 8.1 Hz, 2H), 7.52 (d, J = 8.1 Hz, 2H), 6.56 (t, J = 6.0 Hz, 1H), 6.52 (s, 2H), 6.22 (s, 1H), 4.32 (d, J = 5.9 Hz, 2H), 4.19 (s, 2H), 2.18 (s, 3H), 2.01 (s, 3H). ^{13}C NMR (101 MHz, DMSO-d_6) δ 165.07, 159.89, 158.38, 149.12, 145.72, 145.69, 139.93, 133.75, 131.85, 129.73, 129.62 (d, J = 252.4 Hz), 128.28, 127.82 (d, J = 31.7 Hz), 126.43, 126.20, 126.17, 125.67, 125.62 (d, J = 3.9 Hz), 125.56, 123.47, 117.50, 116.73, 112.37, 110.86, 46.36, 38.17, 11.58, 10.74. HRMS (ESI): m/z calcd for $C_{28}H_{24}F_3N_4O_2$ [M+H]$^+$ 505.1773, found 505.1846.

N-(3-(((3-(3,5-dimethylisoxazol-4-yl)-5-((4-oxo-3,4-dihydrophthalazin-1-yl)methyl)phenyl)amino)methyl)phenyl)acetamide (DDT47)

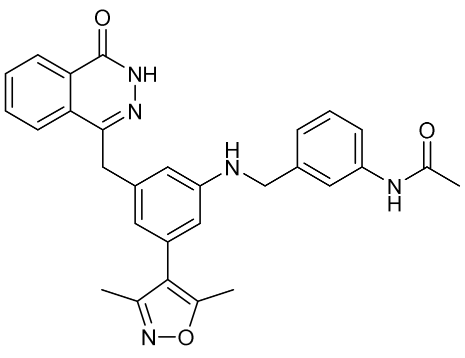

White solid,yield:57%;mp:155-156℃;^{1}H NMR (400 MHz, DMSO-d_6) δ 12.56 (s, 1H), 9.85 (s, 1H), 8.26 (d, J = 9.1 Hz, 1H), 7.95 (d, J = 7.5 Hz, 1H), 7.87 – 7.82 (m, 1H), 7.82 – 7.78 (m, 1H), 7.52 (s, 1H), 7.45 (d, J = 8.1 Hz, 1H), 7.19 (t, J = 7.8 Hz, 1H), 6.97 (d, J = 7.6 Hz, 1H), 6.52 (s, 1H), 6.49 (s, 1H), 6.42 (s, 1H), 6.23 (s, 1H), 4.18 (s, 4H), 2.20 (s, 3H), 2.03 (s, 3H), 2.01 (s, 3H). ^{13}C NMR (101 MHz, DMSO-d_6) δ 168.67, 165.06, 159.89, 158.40, 149.54, 145.75, 141.15, 139.91, 139.78, 133.79, 131.86, 130.79, 129.76, 129.04, 128.37, 126.44, 126.22, 122.28, 118.04, 117.92, 117.17, 116.78, 112.44, 110.70, 47.16, 38.24, 24.45, 11.64, 10.82. HRMS (ESI):m/z calcd for $C_{29}H_{28}N_5O_3$ [M+H]$^+$ 494.2114, found 494.2187.

N-(4-(((3-(3,5-dimethylisoxazol-4-yl)-5-((4-oxo-3,4-dihydrophthalazin-1-yl)methyl)phenyl)amino)methyl)phenyl)acetamide (DDT48)

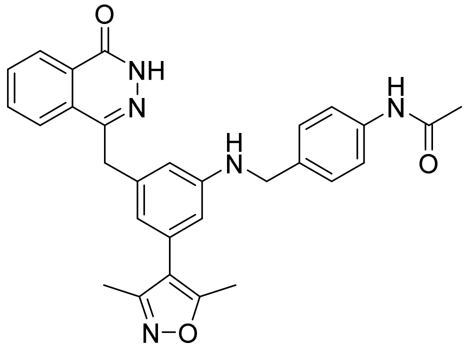

White solid,yield:23.3%;mp:155-156℃;^{1}H NMR (400 MHz, DMSO-d_6) δ 12.55 (s, 1H), 9.84 (s, 1H), 8.26 (d, J = 7.3 Hz, 1H), 7.94 (d, J = 7.7 Hz, 1H), 7.85 (d, J = 6.5 Hz, 1H), 7.79 (d, J = 7.1 Hz, 1H), 7.35 (d, J = 94.8 Hz, 2H), 7.35 (d, J = 111.3 Hz, 2H), 6.52 (s, 1H), 6.48 (s, 1H), 6.31 (s, 1H), 6.29 (s, 1H), 4.18 (s, 2H), 4.14 (d, J = 5.0 Hz, 2H), 2.22 (s, 3H), 2.05 (s, 3H), 2.02 (s, 3H). ^{13}C NMR (101 MHz, DMSO-d_6) δ 168.55, 165.06, 159.89, 158.41, 149.54, 145.75, 139.78, 138.42, 134.87, 133.77, 131.84, 130.80, 129.76, 128.38, 128.01, 126.44, 126.21, 119.47, 117.18, 116.80, 112.36, 110.90, 46.60, 38.27, 24.41, 11.69, 10.86. HRMS (ESI):m/z calcd for $C_{29}H_{28}N_5O_3$ [M+H]$^+$ 494.2114,found 494.2189.

4-(3-((3-aminobenzyl)amino)-5-(3,5-dimethylisoxazol-4-yl)benzyl)phthalazin-1(2H)-one (DDT49)

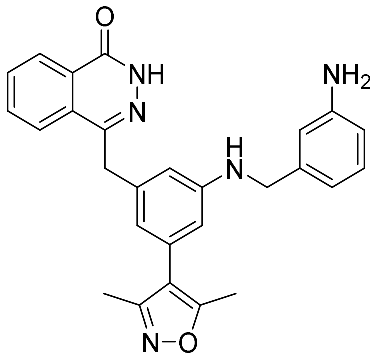

White solid, yield: 64%; mp: 205-206 °C; ^{1}H NMR (400 MHz, DMSO-d_6) δ 12.57 (s, 1H), 8.27 (d, J = 7.5 Hz, 1H), 7.96 (d, J = 7.8 Hz, 1H), 7.86 (t, J = 6.9 Hz, 1H), 7.81 (t, J = 7.4 Hz, 1H), 6.93 (t, J = 7.7 Hz, 1H), 6.54 (d, J = 4.9 Hz, 2H), 6.46 (d, J = 8.8 Hz, 2H), 6.42 (d, J = 7.8 Hz, 1H), 6.27 (s, 2H), 5.01 (s, 2H), 4.19 (s, 2H), 4.06 (s, 2H), 2.23 (s, 3H), 2.05 (s, 3H). ^{13}C NMR (101 MHz, DMSO-d_6) δ 165.05, 159.90, 158.43, 149.75, 149.04, 145.77, 141.07, 139.68, 133.79, 131.84, 130.75, 129.79, 129.23, 128.39, 126.44, 126.22, 116.97, 116.85, 115.29, 113.07, 112.96, 112.36, 110.76, 47.37, 38.29, 11.66, 10.84. HRMS (ESI): m/z calcd for $C_{27}H_{26}N_5O_2$ [M+H]$^+$ 452.2008, found 452.2079.

N-(3-(3,5-dimethylisoxazol-4-yl)-5-((4-oxo-3,4-dihydrophthalazin-1-yl)methyl)phenyl)ethanesulfonamide (DDT50)

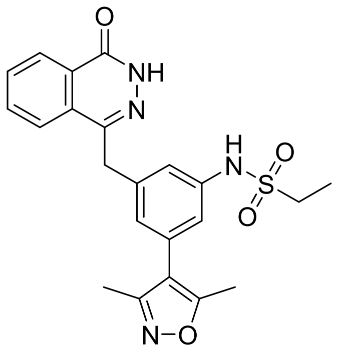

White solid,yield:88%;mp:195-196°C;^{1}H NMR (400 MHz, DMSO-d_6) δ 12.62 (s, 1H), 9.82 (s, 1H), 8.28 (d, J = 7.8 Hz, 1H), 7.99 (d, J = 7.9 Hz, 1H), 7.89 (t, J = 7.6 Hz, 1H), 7.83 (t, J = 6.9 Hz, 1H), 7.14 – 7.09 (m, 2H), 7.06 (t, J = 1.8 Hz, 1H), 4.35 (s, 2H), 3.08 (d, J = 7.3 Hz, 1H), 3.05 (d, J = 7.3 Hz, 1H), 2.37 (s, 3H), 2.19 (s, 3H), 1.14 (t, J = 7.3 Hz, 3H).^{13}C NMR (101 MHz, DMSO-d_6) δ 165.75, 159.86, 158.37, 145.37, 140.67, 139.50, 133.88, 132.00, 131.31, 129.64, 128.37, 126.52, 126.10, 124.77, 118.70, 117.94, 115.84, 45.80, 37.86, 11.83, 10.96, 8.43.HRMS(ESI):m/z calcd for $C_{22}H_{23}N_4O_4S$[M+H]$^+$439.1362,found 439.1430.

N-(3-(3,5-dimethylisoxazol-4-yl)-5-((4-oxo-3,4-dihydrophthalazin-1-yl)methyl)phenyl)propane-1-sulfonamide (DDT51)

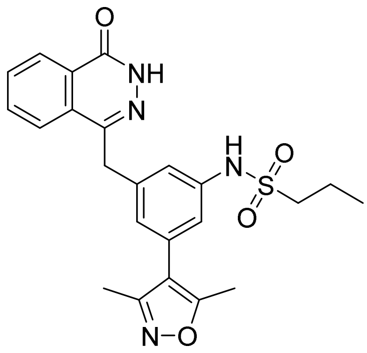

White solid,yield:89%; mp:165-166°C;^{1}H NMR (400 MHz, DMSO-d_6) δ 12.62 (s, 1H), 9.81 (s, 1H), 8.28 (d, J = 7.6 Hz, 1H), 7.98 (d, J = 7.7 Hz, 1H), 7.88 (t, J = 6.8 Hz, 1H), 7.83 (t, J = 7.0 Hz, 1H), 7.14 (t, J = 1.6 Hz, 1H), 7.09 (t, J = 1.8 Hz, 1H), 7.05 (t, J = 1.9 Hz, 1H), 4.35 (s, 2H), 3.05 – 2.98 (m, 2H), 2.37 (s, 3H), 2.19 (s, 3H), 1.63 (d, J = 7.5 Hz, 1H), 1.59 (d, J = 7.5 Hz, 1H), 0.86 (t, J = 7.4 Hz, 3H).^{13}C NMR (101 MHz, DMSO-d_6) δ 165.75, 159.86, 158.37, 145.39, 140.69, 139.49, 133.87, 131.99, 131.31, 129.62, 128.38, 126.52, 126.10, 124.75, 118.59, 117.96, 115.85, 53.04, 37.89, 17.25, 12.94, 11.83, 10.96. HRMS(ESI):m/z calcd for $C_{23}H_{25}N_4O_4S[M+H]^+$453.1518,found 453.1595.

N-(3-(3,5-dimethylisoxazol-4-yl)-5-((4-oxo-3,4-dihydrophthalazin-1-yl)methyl)phenyl)butane-1-sulfonamide (DDT52)

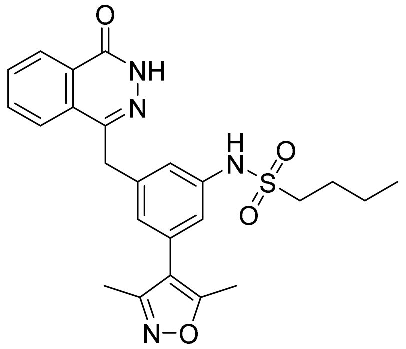

White solid, yield:83%; mp:147-148°C; ^{1}H NMR (400 MHz, DMSO-d_6) δ 12.62 (s, 1H), 9.80 (s, 1H), 8.27 (d, J = 6.5 Hz, 1H), 7.97 (d, J = 7.6 Hz, 1H), 7.87 (t, J = 7.6 Hz, 1H), 7.82 (t, J = 7.3 Hz, 1H), 7.14 (t, J = 1.5 Hz, 1H), 7.09 (d, J = 2.2 Hz, 1H), 7.04 (d, J = 1.9 Hz, 1H), 4.34 (s, 2H), 3.04 – 2.98 (m,2H), 2.37 (s, 3H), 2.18 (s, 3H), 1.55 (p, J = 7.6 Hz, 2H), 1.26 (d, J = 7.5 Hz, 1H), 1.22 (d, J =

7.4 Hz, 1H), 0.73 (t, *J* = 7.3 Hz, 3H). ^{13}C NMR (101 MHz, DMSO-*d*$_6$) δ 165.73, 159.87, 158.36, 145.37, 140.71, 139.50, 133.84, 131.97, 131.32, 129.62, 128.40, 126.51, 126.08, 124.80, 118.59, 118.03, 115.87, 50.94, 37.92, 25.51, 21.02, 13.77, 11.80, 10.93. HRMS (ESI):m/z calcd for $C_{24}H_{27}N_4O_4S$ $[M+H]^+$ 467.1675,found 467.1748.

N-(3-(3,5-dimethylisoxazol-4-yl)-5-((4-oxo-3,4-dihydrophthalazin-1-yl)methyl)phenyl)-2-methoxyethane-1-sulfonamide (DDT53)

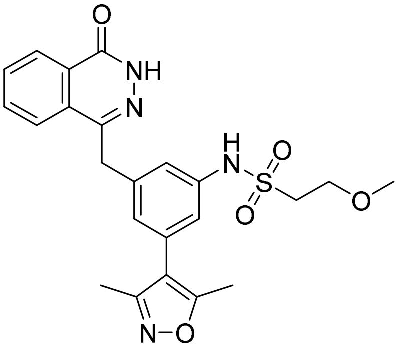

White solid, yield:97%; mp:181-182°C; ^{1}H NMR (400 MHz, DMSO-*d*$_6$) δ 12.62 (s, 1H), 9.80 (s, 1H), 8.28 (d, *J* = 7.7 Hz, 1H), 7.99 (d, *J* = 7.6 Hz, 1H), 7.91 – 7.86 (m, 1H), 7.83 (t, *J* = 6.9 Hz, 1H), 7.14 (t, *J* = 1.6 Hz, 1H), 7.11 (t, *J* = 1.8 Hz, 1H), 7.05 (t, *J* = 1.8 Hz, 1H), 4.35 (s, 2H), 3.60 (t, *J* = 6.0 Hz, 2H), 3.32 (t, *J* = 6.0 Hz, 2H), 3.09 (s, 3H), 2.37 (s, 3H), 2.19 (s, 3H). ^{13}C NMR (101 MHz, DMSO-*d*$_6$) δ 165.73, 159.88, 158.37, 145.38, 140.57, 139.34, 133.86, 131.98, 131.22, 129.64, 128.39, 126.51, 126.10, 124.88, 118.96, 118.26, 115.88, 66.16, 58.31, 51.09, 37.90, 11.80, 10.93. HRMS(ESI):m/z calcd for $C_{23}H_{25}N_4O_5S$ $[M+H]^+$469.1467,found 469.1535.

N-(3-(3,5-dimethylisoxazol-4-yl)-5-((4-oxo-3,4-dihydrophthalazin-1-yl)methyl)phenyl)propane-2-sulfonamide (DDT54)

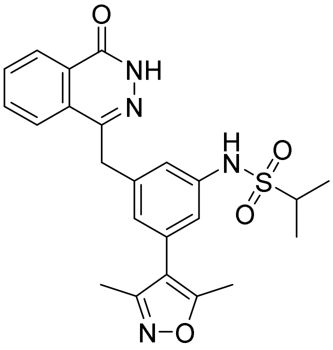

White solid, yield:66%; mp:191-192°C; ^{1}H NMR (400 MHz, DMSO-d_6) δ 12.62 (s, 1H), 9.77 (s, 1H), 8.27 (d, J = 7.7 Hz, 1H), 7.97 (d, J = 8.0 Hz,1H), 7.87 (d, J = 7.6 Hz, 1H), 7.84 (d, J = 7.5 Hz, 1H), 7.09 (d, J = 17.4 Hz, 3H), 4.35 (s, 2H), 3.21 – 3.13 (m, 1H), 2.37 (s, 3H), 2.19 (s, 3H), 1.18 (s, 3H), 1.17 (s, 3H). ^{13}C NMR (101 MHz, DMSO-d_6) δ 165.73, 159.87, 158.36, 145.38, 140.66, 139.71, 133.86, 131.98, 131.27, 129.63, 128.37, 126.51, 126.12, 124.63, 118.58, 117.85, 115.84, 52.12, 37.89, 16.54, 11.83, 10.96. HRMS (ESI):m/z calcd for $C_{23}H_{25}N_4O_4S$ [M+H]$^+$453.1518, found 453.1590.

N-(3-(3,5-dimethylisoxazol-4-yl)-5-((4-oxo-3,4-dihydrophthalazin-1-yl)methyl)phenyl)cyclopropanesulfonamide (DDT55)

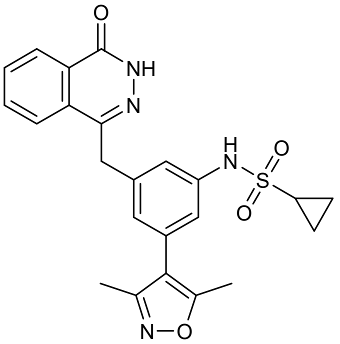

White solid,yield:70%;mp:140-141°C;^{1}H NMR (400 MHz, DMSO-d_6) δ 12.63 (s, 1H), 9.77 (s, 1H), 8.27 (d, J = 6.6 Hz, 1H), 7.99 (d, J = 7.7 Hz, 1H), 7.91 – 7.85 (m, 1H), 7.83 (t, J = 7.0 Hz, 1H), 7.15 (d, J = 1.5 Hz, 1H), 7.13 (t, J = 1.8 Hz, 1H), 7.06 (t, J = 1.8 Hz, 1H), 4.35 (s, 2H), 2.62 – 2.55 (m, 1H), 2.38 (s, 3H), 2.20 (s, 3H), 0.89 – 0.80 (m, 4H).^{13}C NMR (101 MHz, DMSO-d_6) δ 165.72, 159.87, 158.38, 145.41, 140.52, 139.43, 133.87, 131.99, 131.21, 129.62, 128.40, 126.52, 126.11, 125.00, 119.43, 118.83, 115.88, 37.91, 30.16, 11.82, 10.94, 5.40. HRMS(ESI):m/z calcd for $C_{23}H_{23}N_4O_4S$ [M+H]$^+$451.1362, found 451.1432.

N-(3-(3,5-dimethylisoxazol-4-yl)-5-((4-oxo-3,4-dihydrophthalazin-1-yl)methyl)phenyl)cyclohexanesulfonamide (DDT56)

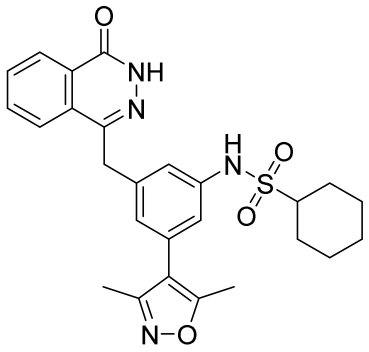

Red solid, yield:49%; mp:144-145℃; ^{1}H NMR (400 MHz, DMSO-d_6) δ 12.63 (s, 1H), 9.76 (s, 1H), 8.27 (d, *J* = 6.6 Hz, 1H), 7.96 (d, *J* = 7.7 Hz, 1H), 7.90 – 7.85 (m, 1H), 7.85 – 7.80 (m, 1H), 7.14 (t, *J* = 1.5 Hz, 1H), 7.10 (t, *J* = 1.8 Hz, 1H), 7.05 (t, *J* = 1.8 Hz, 1H), 4.35 (s, 2H), 2.92 – 2.82 (m, 1H), 2.38 (s, 3H), 2.20 (s, 3H), 1.91 (d, *J* = 11.8 Hz, 2H), 1.67 (d, *J* = 11.9 Hz, 2H), 1.53 (d, *J* = 8.7 Hz, 1H), 1.38 – 1.21 (m, 2H), 1.14 – 0.96 (m, 3H). ^{13}C NMR (101 MHz, DMSO-d_6) δ 165.72, 159.87, 158.36, 145.41, 140.66, 139.73, 133.81, 131.96, 131.26, 129.62, 128.41, 126.51, 126.10, 124.60, 118.45, 117.95, 115.88, 59.99, 37.95, 26.42,25.12,24.79,11.82,10.94. HRMS(ESI):m/z calcd for $C_{26}H_{29}N_4O_4S$ $[M+H]^+$493.1831, found 493.1910.

N-(3-(3,5-dimethylisoxazol-4-yl)-5-((4-oxo-3,4-dihydrophthalazin-1-yl)methyl)phenyl)benzenesulfonamide (DDT57)

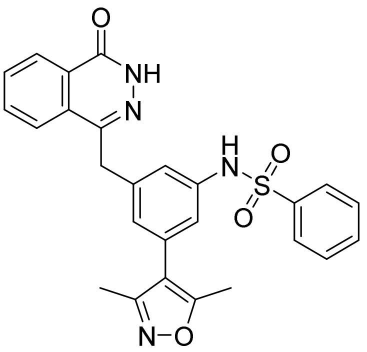

White solid, yield:57%; mp:171-172℃; ^{1}H NMR (400 MHz, DMSO-d_6) δ 12.65 (s, 1H), 10.36 (s, 1H), 8.32 – 8.25 (m, 1H), 7.93 – 7.84 (m, 1H), 7.85 (d, *J* = 2.2 Hz, 1H), 7.83 (d, *J* = 3.7 Hz, 1H), 7.65 (s, 1H), 7.63 (s, 1H), 7.53 (t, *J* = 7.4 Hz, 1H), 7.42 (t, *J* = 7.6 Hz, 2H), 7.13 (s, 1H), 7.00 (d, *J* = 1.8 Hz, 1H), 6.85 (t, *J* = 1.9 Hz, 1H), 4.27 (s, 2H), 2.28 (s, 3H), 2.10 (s, 3H). ^{13}C NMR (101 MHz, DMSO-d_6) δ 165.69, 159.90, 158.28, 145.32, 140.57, 139.69, 138.73, 133.85, 133.32, 131.98, 131.17,

129.58, 129.51, 128.40, 127.04, 126.51, 126.00, 125.23, 118.95, 118.61, 115.71, 37.80, 11.72, 10.83. HRMS(ESI):m/z calcd for $C_{26}H_{23}N_4O_4S$ $[M+H]^+$487.1362, found 487.1439.

N-(3-(3,5-dimethylisoxazol-4-yl)-5-((4-oxo-3,4-dihydrophthalazin-1-yl)methyl)phenyl)-3-methylbenzenesulfonamide (DDT58)

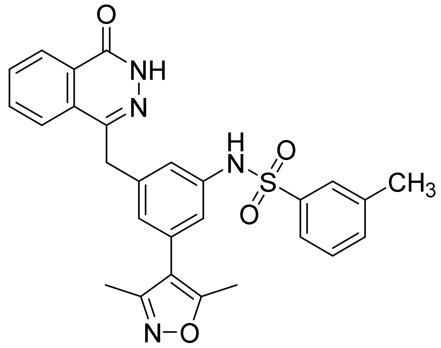

White solid, yield:66%; mp:146-147°C; ^{1}H NMR (400 MHz, DMSO-d_6) δ 12.63 (s, 1H), 10.28 (s, 1H), 8.31 – 8.25 (m, 1H), 7.93 – 7.88 (m, 1H), 7.85 (d, J = 2.0 Hz, 1H), 7.84 – 7.81 (m, 1H), 7.49 (s, 1H), 7.41 (d, J = 7.7 Hz, 1H), 7.33 (d, J = 7.6 Hz, 1H), 7.28 (t, J = 7.6 Hz, 1H), 7.12 (s, 1H), 6.98 (t, J = 1.8 Hz, 1H), 6.86 (t, J = 1.9 Hz, 1H), 4.27 (s, 2H), 2.27 (s, 3H), 2.24 (s, 3H), 2.09 (s, 3H). ^{13}C NMR (101 MHz, DMSO-d_6) δ 165.68, 159.88, 158.27, 145.29, 140.55, 139.68, 139.39, 138.79, 133.96, 133.82, 131.97, 131.13, 129.51, 129.35, 128.40, 127.18, 126.51, 126.00, 125.27, 124.22, 119.21, 118.66,115.75,37.81,21.15,11.67,10.79. HRMS(ESI):m/z calcd for $C_{27}H_{25}N_4O_4S$ $[M+H]^+$ 501.1518, found 501.1593.

N-(3-(3,5-dimethylisoxazol-4-yl)-5-((4-oxo-3,4-dihydrophthalazin-1-yl)methyl)phenyl)-4-methylbenzenesulfonamide (DDT59)

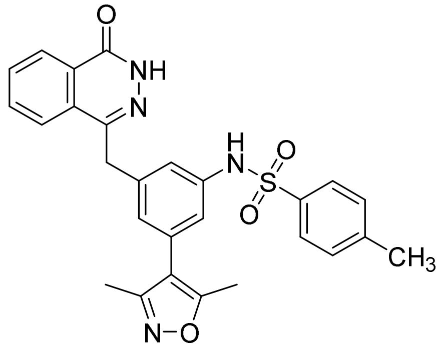

White solid, yield:72%; mp:157-158°C;^{1}H NMR (400 MHz, DMSO-d_6) δ 12.64 (s, 1H), 10.28 (s, 1H), 8.31 – 8.25 (m, 1H), 7.92 – 7.86 (m, 1H), 7.84 (d, J = 2.1 Hz, 1H), 7.84 – 7.82 (m, 1H), 7.52 (s, 1H), 7.50 (s, 1H), 7.23 (s, 1H), 7.21 (s, 1H), 7.11 (t, J = 1.6 Hz, 1H), 6.99 (t, J = 1.8 Hz, 1H), 6.83 (t, J = 1.8 Hz, 1H), 4.27 (s, 2H), 2.29 (s, 3H), 2.28 (s, 3H), 2.10 (s, 3H).^{13}C NMR (101 MHz, DMSO-d_6) δ 165.68, 159.90, 158.28, 145.33, 143.74, 140.54, 138.88, 136.87, 133.83, 131.95, 131.14, 130.03, 129.52, 128.40, 127.10, 126.49, 126.03, 125.03, 118.59, 118.33, 115.73, 37.83, 21.37, 11.71, 10.83. HRMS(ESI):m/z calcd for $C_{27}H_{25}N_4O_4S$ $[M+H]^+$ 501.1518, found 501.1588.

N-(3-(3,5-dimethylisoxazol-4-yl)-5-((4-oxo-3,4-dihydrophthalazin-1-yl)methyl)phenyl)-3,5-dimethylbenzenesulfonamide (DDT60)

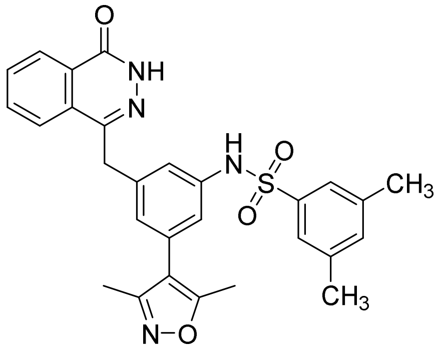

Yellow solid, yield:88%; mp:155-156°C;^{1}H NMR (400 MHz, DMSO-d_6) δ 12.59 (s, 1H), 10.19 (s, 1H), 8.30 – 8.25 (m, 1H), 7.93 – 7.88 (m, 1H), 7.85 (d, J = 5.8 Hz, 1H), 7.82 (d, J = 4.5 Hz, 1H), 7.26 (d, J = 1.6 Hz, 2H), 7.13 (s, 1H), 7.11 (d, J = 1.5 Hz, 1H), 6.97 (t, J = 1.8 Hz, 1H), 6.88 (t, J = 1.8 Hz, 1H), 4.26 (s, 2H), 2.27 (s, 3H), 2.18 (s, 6H), 2.09 (s, 3H).^{13}C NMR (101 MHz, DMSO-d_6) δ 165.65, 159.85, 158.26, 145.25, 140.51, 139.72, 139.12, 138.88, 134.62, 133.78, 131.94, 131.09, 129.52, 128.40, 126.50, 125.97, 125.33, 124.44, 119.52, 118.75, 115.81, 37.84, 21.04, 11.62, 10.74. HRMS(ESI):m/z calcd for $C_{28}H_{27}N_4O_4S$$[M+H]^+$ 515.1675,found 515.1755.

N-(3-(3,5-dimethylisoxazol-4-yl)-5-((4-oxo-3,4-dihydrophthalazin-1-yl)methyl)phenyl)-3,4-dimethylbenzenesulfonamide (DDT61)

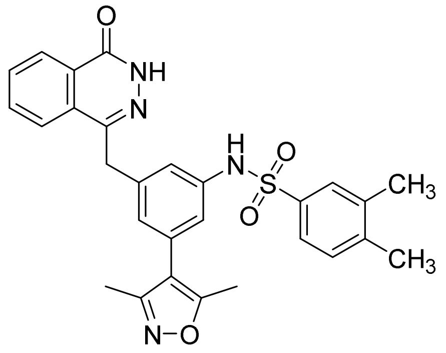

White solid, yield:79%; mp:158-159℃; ^{1}H NMR (400 MHz, DMSO-d_6) δ 12.60 (s, 1H), 10.20 (s, 1H), 8.31 – 8.24 (m, 1H), 7.93 – 7.87 (m, 1H), 7.85 (d, *J* = 5.2 Hz, 1H), 7.83 – 7.81 (m, 1H), 7.44 (d, *J* = 2.0 Hz, 1H), 7.33 (d, *J* = 6.5 Hz, 1H), 7.16 (d, *J* = 8.0 Hz, 1H), 7.09 (d, *J* = 1.6 Hz, 1H), 7.00 (t, *J* = 1.7 Hz, 1H), 6.84 (t, *J* = 1.9 Hz, 1H), 4.27 (s, 2H), 2.26 (s, 3H), 2.20 (s, 3H), 2.15 (s, 3H), 2.08 (s, 3H). ^{13}C NMR (101 MHz, DMSO-d_6) δ 165.65, 159.87, 158.26, 145.30, 142.58, 140.52, 138.98, 138.03, 137.17, 133.80, 131.93, 131.10, 130.34, 129.54, 128.39, 127.61, 126.50, 126.01, 125.04, 124.65, 118.92, 118.33, 115.78, 37.85, 19.81, 19.70, 11.65, 10.76. HRMS(ESI):m/z calcd for $C_{28}H_{27}N_4O_4S$ $[M+H]^+$ 515.1675, found 515.1757.

N-(3-(3,5-dimethylisoxazol-4-yl)-5-((4-oxo-3,4-dihydrophthalazin-1-yl)methyl)phenyl)-4-ethylbenzenesulfonamide (DDT62)

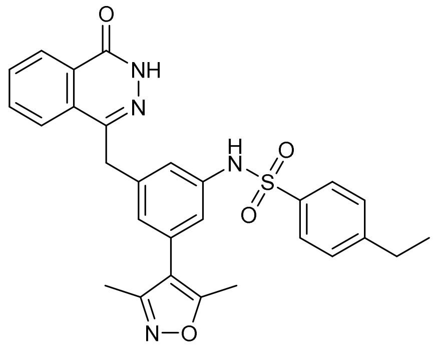

White solid, yield:71%; mp:202-203℃;^{1}H NMR (400 MHz, DMSO-d_6) δ 12.67 (s, 1H), 10.33 (s, 1H), 8.31 – 8.27 (m, 1H), 7.94 – 7.90 (m, 1H), 7.87 – 7.83 (m, 1H), 7.82 (d, *J* = 7.2 Hz, 1H), 7.56 (s, 1H), 7.54 (s, 1H), 7.27 (s, 1H), 7.25 (s, 1H), 7.13 (s, 1H), 7.04 (d, *J* = 1.8 Hz, 1H), 6.81 (t, *J* = 1.8 Hz, 1H), 4.28 (s, 2H), 2.61 (d, *J* = 7.5 Hz, 1H), 2.57 (d, *J* = 7.6 Hz, 1H), 2.27 (s, 3H), 2.09 (s, 3H), 1.11 (t, *J* = 7.6 Hz, 3H).^{13}C NMR (101 MHz, DMSO-d_6) δ 165.67, 159.91, 158.28, 149.71, 145.38, 140.54, 138.90, 137.11, 133.84, 131.96, 131.14, 129.52, 128.92, 128.39, 127.24, 126.49, 126.05, 124.95,

118.38, 118.13, 115.73, 37.82, 28.36, 15.36, 11.72, 10.84. HRMS(ESI):m/z calcd for $C_{28}H_{27}N_4O_4S[M+H]^+$515.1675, found 515.1752.

N-(3-(3,5-dimethylisoxazol-4-yl)-5-((4-oxo-3,4-dihydrophthalazin-1-yl)methyl)phenyl)-4-propylbenzenesulfonamide (DDT63)

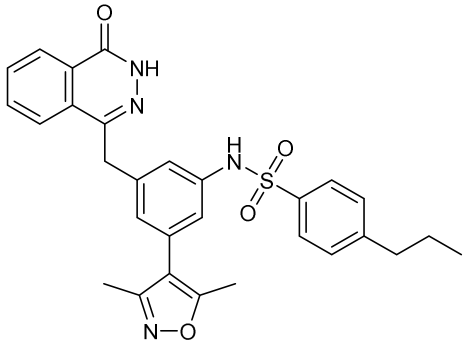

White solid, yield:87%; mp:148-149°C;^{1}H NMR (400 MHz, DMSO-d_6) δ 12.65 (s, 1H), 10.29 (s, 1H), 8.30 – 8.26 (m, 1H), 7.93 – 7.89 (m, 1H), 7.84 (d, J = 2.1 Hz, 1H), 7.84 – 7.81 (m, 1H), 7.56 – 7.54 (m, 1H), 7.53 (d, J = 1.8 Hz, 1H), 7.24 (s, 1H), 7.23 (d, J = 1.8 Hz, 1H), 7.11 (t, J = 1.5 Hz, 1H), 7.04 (t, J = 1.8 Hz, 1H), 6.80 (t, J = 1.8 Hz, 1H), 4.27 (s, 2H), 2.54 (d, J = 7.4 Hz, 2H), 2.26 (s, 3H), 2.08 (s, 3H), 1.53 (d, J = 7.4 Hz, 1H), 1.49 (d, J = 7.4 Hz, 1H), 0.82 (t, J = 7.3 Hz, 3H).^{13}C NMR (101 MHz, DMSO-d_6) δ 165.65, 159.90, 158.26, 148.14, 145.35, 140.52, 138.94, 137.18, 133.82, 131.93, 131.13, 129.54, 129.43, 128.41, 127.15, 126.49, 126.02, 124.99, 118.59, 118.26, 115.75, 37.83, 37.28, 23.95, 13.92, 11.70, 10.81. HRMS(ESI):m/z calcd for $C_{29}H_{29}N_4O_4S[M+H]^+$529.1831, found 529.1912.

N-(3-(3,5-dimethylisoxazol-4-yl)-5-((4-oxo-3,4-dihydrophthalazin-1-yl)methyl)phenyl)-4-isopropylbenzenesulfonamide (DDT64)

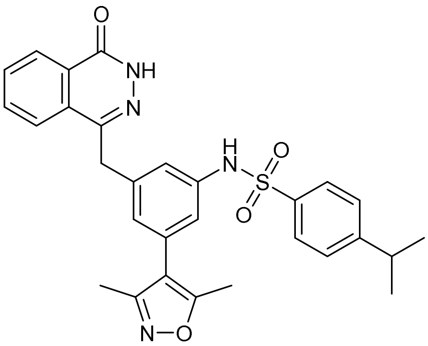

White solid, yield:80%; mp:158-159℃; ^{1}H NMR (400 MHz, DMSO-d_6) δ 12.69 (s, 1H), 10.34 (s, 1H), 8.32 – 8.26 (m, 1H), 7.97 – 7.91 (m, 1H), 7.87 – 7.84 (m, 1H), 7.84 – 7.81 (m, 1H), 7.58 (d, *J* = 1.8 Hz, 1H), 7.56 (d, *J* = 1.8 Hz, 1H), 7.31 (d, *J* = 2.0 Hz, 1H), 7.30 (d, *J* = 1.8 Hz, 1H), 7.13 (d, *J* = 1.6 Hz, 1H), 7.08 (t, *J* = 1.8 Hz, 1H), 6.79 (t, *J* = 1.8 Hz, 1H), 4.28 (s, 2H), 2.88 (hept, *J* = 7.3 Hz, 1H), 2.26 (s, 3H), 2.08 (s, 3H), 1.14 (s, 3H), 1.12 (s, 3H). ^{13}C NMR (101 MHz, DMSO-d_6) δ 165.66, 159.91, 158.28, 154.20, 145.42, 140.54, 138.91, 137.26, 133.84, 131.97, 131.15, 129.53, 128.40, 127.57, 127.32, 126.51, 126.07, 124.89, 118.20, 117.96, 115.73, 37.81, 33.75, 23.78, 11.72, 10.83.HRMS(ESI):m/z calcd for $C_{29}H_{29}N_4O_4S[M+H]^+$529.1831, found 529.1912.

4-(tert-butyl)-N-(3-(3,5-dimethylisoxazol-4-yl)-5-((4-oxo-3,4-dihydrophthalazin-1-yl)methyl)phenyl)benzenesulfonamide (DDT65)

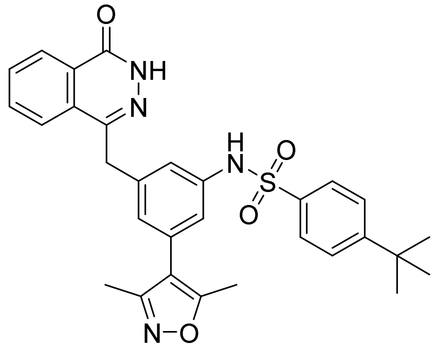

White solid, yield:68%; mp:154-155℃; ^{1}H NMR (400 MHz, DMSO-d_6) δ 12.68 (s, 1H), 10.35 (s, 1H), 8.30 – 8.25 (m, 1H), 7.99 – 7.92 (m, 1H), 7.87 – 7.84 (m, 1H), 7.84 – 7.79 (m, 1H), 7.59 (s, 1H), 7.57 (s, 1H), 7.47 (s, 1H), 7.45 (s, 1H), 7.12 (t, *J* = 1.5 Hz, 1H), 7.10 (t, *J* = 1.8 Hz, 1H), 6.77 (t, *J* = 1.8 Hz, 1H), 4.28 (s, 2H), 2.25 (s, 3H), 2.07 (s, 3H), 1.22 (s, 9H). ^{13}C NMR (101 MHz, DMSO-d_6) δ 165.64, 159.90, 158.26, 156.44, 145.43, 140.54, 138.98, 137.07, 133.84, 131.95, 131.16, 129.56, 128.41, 127.05, 126.52, 126.49, 126.07, 124.83, 118.18, 117.90, 115.76, 37.81, 35.27, 31.14, 11.71, 10.82. HRMS(ESI):m/z calcd for $C_{30}H_{31}N_4O_4S$ $[M+H]^+$543.1988, found 543.2062.

N-(3-(3,5-dimethylisoxazol-4-yl)-5-((4-oxo-3,4-dihydrophthalazin-1-yl)methyl)phenyl)-6-methylpyridine-3-sulfonamide (DDT66)

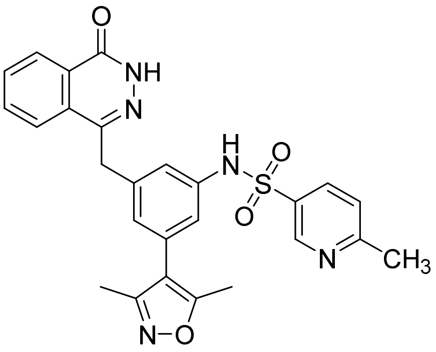

White solid, yield:8%; mp:141-142°C; ^{1}H NMR (400 MHz, DMSO-d_6) δ 12.61 (s, 1H), 10.47 (s, 1H), 8.67 (d, J = 2.3 Hz, 1H), 8.29 – 8.25 (m, 1H), 7.91 – 7.87 (m, 1H), 7.86 – 7.84 (m, 1H), 7.84 – 7.81 (m, 2H), 7.31 (d, J = 8.2 Hz, 1H), 7.14 (t, J = 1.5 Hz, 1H), 7.01 (t, J = 1.8 Hz, 1H), 6.87 (t, J = 1.8 Hz, 1H), 4.28 (s, 2H), 2.47 (s, 3H), 2.27 (s, 3H), 2.09 (s, 3H). ^{13}C NMR (101 MHz, DMSO-d_6) δ 165.74, 163.47, 159.87, 158.26, 147.12, 145.28, 140.74, 138.32, 135.18, 133.82, 133.37, 131.95, 131.33, 129.50, 128.37, 126.52, 125.96, 125.71, 123.88, 119.50, 119.05, 115.64, 37.77, 24.53, 11.69, 10.79. HRMS(ESI):m/z calcd for $C_{26}H_{24}N_5O_4S$ $[M+H]^+$ 502.1471, found 502.1545.

N-(3-(3,5-dimethylisoxazol-4-yl)-5-((4-oxo-3,4-dihydrophthalazin-1-yl)methyl)phenyl)-2-methoxybenzenesulfonamide (DDT67)

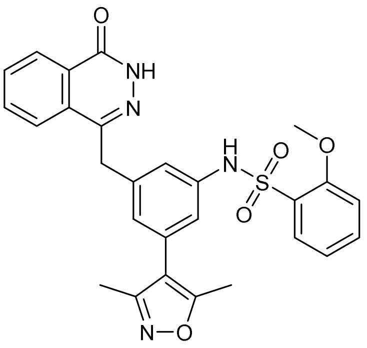

White solid, yield:84%; mp:163-164°C; ^{1}H NMR (400 MHz, DMSO-d_6) δ 12.64 (s, 1H), 10.03 (s, 1H), 8.31 – 8.24 (m, 1H), 7.86 – 7.82 (m, 2H), 7.82 (s, 1H), 7.63 (d, J = 6.2 Hz, 1H), 7.50 – 7.41 (m, 1H), 7.06 (t, J = 1.5 Hz, 1H), 7.04 – 6.97 (m, 1H), 6.97 – 6.92 (m, 1H), 6.94 – 6.87 (m, 2H), 4.23 (s, 2H), 3.73 (s, 3H),2.27 (s, 3H), 2.09 (s, 3H). ^{13}C NMR (101 MHz, DMSO-d_6) δ 165.62, 159.92, 158.29, 156.68, 145.28, 140.35, 138.96, 135.46, 133.83, 131.94, 130.90, 130.69, 129.53, 128.41, 126.48, 126.45, 126.00, 124.73, 120.38, 118.45, 117.97, 115.82, 113.11, 56.39, 37.80, 11.75, 10.87. HRMS(ESI):m/z calcd for $C_{27}H_{25}N_4O_5S$ $[M+H]^+$ 517.1467,found 517.1547.

N-(3-(3,5-dimethylisoxazol-4-yl)-5-((4-oxo-3,4-dihydrophthalazin-1-yl)methyl)phenyl)-3-methoxybenzenesulfonamide (DDT68)

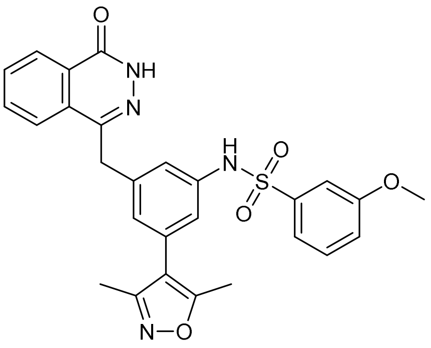

White solid, yield:19%; mp:184-185°C; ^{1}H NMR (400 MHz, DMSO-d_6) δ 12.62 (s, 1H), 10.30 (s, 1H), 8.31 – 8.25 (m, 1H), 7.92 – 7.88 (m, 1H), 7.86 – 7.84 (m, 1H), 7.84 – 7.82 (m, 1H), 7.32 (t, J = 8.0 Hz, 1H), 7.19 (d, J = 7.9 Hz, 1H), 7.16 – 7.14 (m, 1H), 7.14 (s, 1H), 7.11 – 7.06 (m, 1H), 7.01 (t, J = 1.8 Hz, 1H), 6.87 (t, J = 1.8 Hz, 1H), 4.27 (s, 2H), 3.68 (s, 3H), 2.28 (s, 3H), 2.10 (s, 3H). ^{13}C NMR (101 MHz, DMSO-d_6) δ 165.70, 159.88, 159.74, 158.27, 145.29, 140.85, 140.58, 138.72, 133.81, 131.97, 131.14, 130.72, 129.50, 128.39, 126.51, 125.98, 125.42, 119.43, 119.15, 118.90, 115.72, 111.92, 55.93, 37.80, 11.68, 10.80. HRMS(ESI):m/z calcd for $C_{27}H_{25}N_4O_5S$ $[M+H]^+$517.1467,found 517.1544.

N-(3-(3,5-dimethylisoxazol-4-yl)-5-((4-oxo-3,4-dihydrophthalazin-1-yl)methyl)phenyl)-4-methoxybenzenesulfonamide (DDT69)

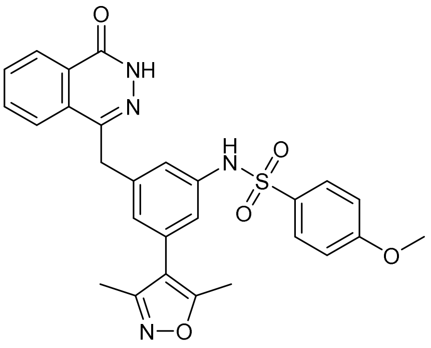

White solid, yield:100%; mp:155-156°C; ^{1}H NMR (400 MHz, DMSO-d_6) δ 12.67 (s, 1H), 10.23 (s, 1H), 8.32 – 8.24 (m, 1H), 7.94 – 7.89 (m, 1H), 7.85 (d, J = 2.4 Hz, 1H), 7.83 (d, J = 5.8 Hz, 1H), 7.58 (s, 1H), 7.55 (s, 1H), 7.11 (t, J = 1.6 Hz, 1H), 7.02 (t, J = 1.9 Hz, 1H), 6.95 (s, 1H), 6.92 (s, 1H),

6.82 (t, *J* = 1.9 Hz, 1H), 4.27 (s, 2H), 3.78 (s, 3H), 2.29 (s, 3H), 2.11 (s, 3H). ^{13}C NMR (101 MHz, DMSO-d_6) δ 165.68, 162.87, 159.92, 158.29, 145.42, 140.52, 139.01, 133.85, 131.99, 131.30, 131.13, 129.52, 129.32, 128.38, 126.50, 126.06, 124.85, 118.28, 118.07, 115.75, 114.72, 56.08, 37.80, 11.75, 10.86. HRMS(ESI):m/z calcd for $C_{27}H_{25}N_4O_5S$ $[M+H]^+$ 517.1467,found 517.1546.

N-(3-(3,5-dimethylisoxazol-4-yl)-5-((4-oxo-3,4-dihydrophthalazin-1-yl)methyl)phenyl)-3,4-dimethoxybenzenesulfonamide (DDT70)

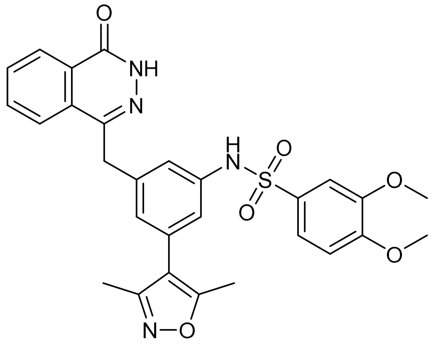

Yellow solid, yield:73%; mp:165-166°C; ^{1}H NMR (400 MHz, DMSO-d_6) δ 12.64 (s, 1H), 10.14 (s, 1H), 8.29 – 8.25 (m, 1H), 7.93 – 7.88 (m, 1H), 7.84 (s, 1H), 7.82 (d, *J* = 4.0 Hz, 1H), 7.20 (d, *J* = 8.4 Hz, 1H), 7.15 (d, *J* = 2.2 Hz, 1H), 7.11 (d, *J* = 1.6 Hz, 1H), 7.05 (d, *J* = 1.8 Hz, 1H), 6.93 (d, *J* = 8.5 Hz, 1H), 6.85 (t, *J* = 1.8 Hz, 1H), 4.27 (s, 2H), 3.78 (s, 3H), 3.64 (s, 3H), 2.28 (s, 3H), 2.09 (s, 3H). ^{13}C NMR (101 MHz, DMSO-d_6) δ 165.66, 159.90, 158.26, 152.68, 149.06, 145.38, 140.49, 139.07, 133.80, 131.99, 131.12, 131.09, 129.53, 128.38, 126.49, 126.01, 125.06, 121.13, 118.94, 118.56, 115.76, 111.21, 109.57, 56.24, 56.06, 37.81, 11.68, 10.80.HRMS(ESI):m/z calcd for $C_{28}H_{27}N_4O_6S$ $[M+H]^+$ 547.1573, found 547.1653.

N-(3-(3,5-dimethylisoxazol-4-yl)-5-((4-oxo-3,4-dihydrophthalazin-1-yl)methyl)phenyl)-2,3-dihydrobenzo[b][1,4]dioxine-6-sulfonamide (DDT71)

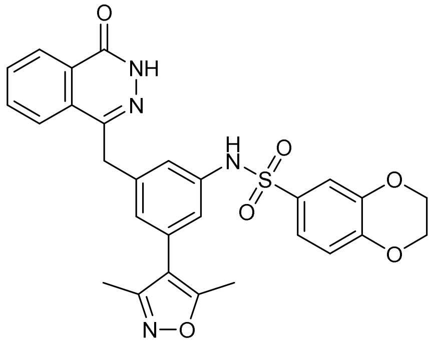

White solid, yield:79%; mp:153-154°C; ^{1}H NMR (400 MHz, DMSO-d_6) δ 12.60 (s, 1H), 10.20 (s, 1H), 8.30 – 8.24 (m, 1H), 7.94 – 7.90 (m, 1H), 7.86 – 7.82 (m, 2H), 7.14 (d, *J* = 2.2 Hz, 1H), 7.11 – 7.07 (m, 2H), 7.03 (t, *J* = 1.8 Hz, 1H), 6.86 (d, *J* = 8.3 Hz, 2H), 4.28 (s, 2H), 4.27 – 4.25 (m, 2H), 4.24 – 4.21 (m, 2H), 2.29 (s, 3H), 2.11 (s, 3H). ^{13}C NMR (101 MHz, DMSO-d_6) δ 165.68, 159.88, 158.28, 147.64, 145.33, 143.70, 140.57, 138.94, 133.83, 132.10, 131.95, 131.14, 129.55, 128.40, 126.52, 126.02, 125.07, 120.72, 118.84, 118.25, 117.85, 116.04, 115.77, 64.81, 64.48, 37.83, 11.71, 10.83. HRMS(ESI):m/z calcd for $C_{28}H_{25}N_4O_6S$ $[M+H]^+$ 545.1417,found 545.1497.

N-(3-(N-(3-(3,5-dimethylisoxazol-4-yl)-5-((4-oxo-3,4-dihydrophthalazin-1-yl)methyl)phenyl)sulfamoyl)phenyl)acetamide (DDT72)

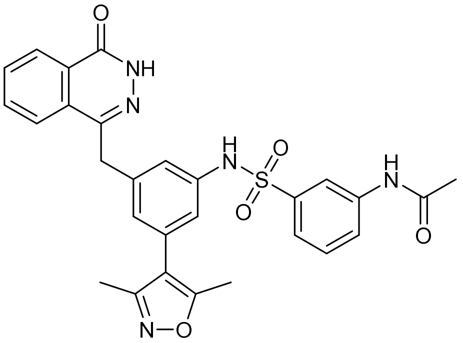

Yellow solid, yield:50%; mp:192-193°C; ^{1}H NMR (400 MHz, DMSO-d_6) δ 12.58 (s, 1H), 10.38 (s, 1H), 10.18 (s, 1H), 8.31 – 8.24 (m, 1H), 8.20 (t, *J* = 2.0 Hz, 1H), 7.91 – 7.84 (m, 1H), 7.83 (s, 1H), 7.82 (d, *J* = 4.1 Hz, 1H), 7.61 (d, *J* = 8.0 Hz, 1H), 7.35 (t, *J* = 7.9 Hz, 1H), 7.29 (d, *J* = 7.8 Hz, 1H), 7.07 (d, *J* = 1.6 Hz, 1H), 7.00 (t, *J* = 1.8 Hz, 1H), 6.85 (t, *J* = 1.8 Hz, 1H), 4.27 (s, 2H), 2.26 (s, 3H), 2.08 (s, 3H), 2.04 (s, 3H). ^{13}C NMR (101 MHz, DMSO-d_6) δ 169.23, 165.68, 159.87, 158.28, 145.28, 140.52, 140.43, 140.36, 138.75, 133.81, 131.95, 131.13, 129.94, 129.55, 128.38, 126.50, 125.97, 125.11, 123.19, 121.40, 119.14, 118.39, 117.08, 115.76, 37.82, 24.45, 11.67, 10.78. HRMS(ESI):m/z calcd for $C_{28}H_{26}N_5O_5S[M+H]^+$ 544.1576, found 544.1645.

N-(4-(N-(3-(3,5-dimethylisoxazol-4-yl)-5-((4-oxo-3,4-dihydrophthalazin-1-yl)methyl)phenyl)sulfamoyl)phenyl)acetamide (DDT73)

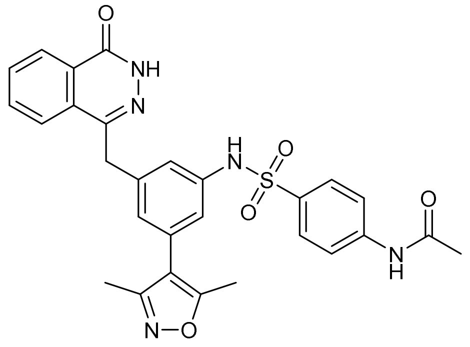

Yellow solid, yield:63%; mp:185-186°C; ^{1}H NMR (400 MHz, DMSO-d_6) δ 12.61 (s, 1H), 10.29 (s, 1H), 10.24 (s, 1H), 8.30 – 8.26 (m, 1H), 7.86 (t, J = 3.9 Hz, 1H), 7.84 (d, J = 2.3 Hz, 1H), 7.83 – 7.81 (m, 1H), 7.64 (d, J = 9.0 Hz, 2H), 7.59 (d, J = 9.0 Hz, 2H), 7.06 (t, J = 1.5 Hz, 1H), 7.03 (t, J = 1.8 Hz, 1H), 6.84 (t, J = 1.9 Hz, 1H), 4.28 (s, 2H), 2.26 (s, 3H), 2.09 (s, 3H), 2.08 (s, 3H). ^{13}C NMR (101 MHz, DMSO-d_6) δ 169.45, 165.65, 159.87, 158.26, 145.29, 143.62, 140.54, 138.98, 133.79, 133.31, 131.91, 131.12, 129.54, 128.39, 128.28, 126.53, 125.98, 124.98, 119.03, 118.34, 115.76, 37.85, 24.57, 11.70, 10.81. HRMS(ESI):m/z calcd for $C_{28}H_{26}N_5O_5S$ [M+H]$^+$ 544.1576, found 544.1646.

4-(benzyloxy)-N-(3-(3,5-dimethylisoxazol-4-yl)-5-((4-oxo-3,4-dihydrophthalazin-1-yl)methyl)phenyl)benzenesulfonamide (DDT74)

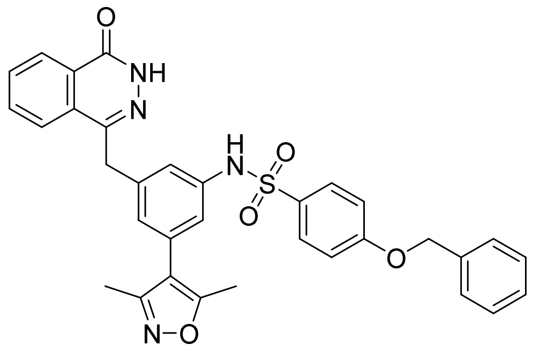

White solid, yield:58%; mp:148-149°C; ^{1}H NMR (400 MHz, DMSO-d_6) δ 12.66 (s, 1H), 10.24 (s, 1H), 8.30 – 8.25 (m, 1H), 7.94 – 7.89 (m, 1H), 7.88 – 7.78 (m, 2H), 7.59 (s, 1H), 7.57 (s, 1H), 7.46 – 7.43 (m, 2H), 7.42 – 7.37 (m, 2H), 7.35 (d, J = 7.0 Hz, 1H), 7.10 (t, J = 1.6 Hz, 1H), 7.04 (s, 1H), 7.02 (d, J = 2.0 Hz, 2H), 6.82 (t, J = 1.8 Hz, 1H), 5.13 (s, 2H), 4.27 (s, 2H), 2.27 (s, 3H), 2.09 (s, 3H). ^{13}C NMR (101 MHz, DMSO-d_6) δ 165.66, 161.99, 159.93, 158.28, 145.40, 140.52, 139.03, 136.67, 133.84, 131.96, 131.60, 131.15, 129.55, 129.32, 128.98, 128.56, 128.40, 128.30, 126.50, 126.05,

124.87, 118.42, 118.11, 115.77, 115.49, 70.13, 37.83, 11.73, 10.84. HRMS(ESI):m/z calcd for $C_{33}H_{29}N_4O_5S$ $[M+H]^+$ 593.1780, found 593.1855.

N-(3-(3,5-dimethylisoxazol-4-yl)-5-((4-oxo-3,4-dihydrophthalazin-1-yl)methyl)phenyl)-2,3-dihydrobenzofuran-5-sulfonamide (DDT75)

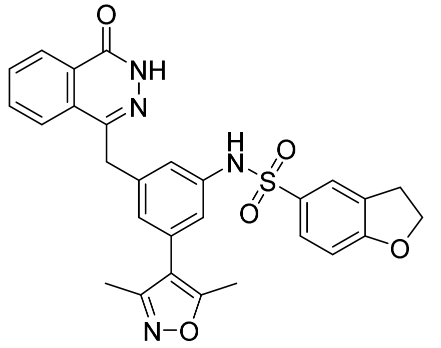

White solid, yield:49%; mp:162-163℃; ^{1}H NMR (400 MHz, DMSO-d_6) δ 12.62 (s, 1H), 10.15 (s, 1H), 8.31 – 8.24 (m, 1H), 7.93 – 7.89 (m, 1H), 7.87 – 7.84 (m, 1H), 7.84 – 7.81 (m, 1H), 7.52 (d, J = 2.0 Hz, 1H), 7.40 (d, J = 8.4 Hz, 1H), 7.09 (t, J = 1.5 Hz, 1H), 7.01 (d, J = 1.7 Hz, 1H), 6.85 (d, J = 1.8 Hz, 1H), 6.73 (d, J = 8.4 Hz, 1H), 4.58 (t, J = 8.8 Hz, 2H), 4.27 (s, 2H), 3.13 (t, J = 8.8 Hz, 2H), 2.28 (s, 3H), 2.10 (s, 3H). ^{13}C NMR (101 MHz, DMSO-d_6) δ 165.66, 163.71, 159.88, 158.29, 145.36, 140.52, 139.11, 133.82, 131.96, 131.38, 131.11, 129.55, 129.36, 128.58, 128.37, 126.52, 126.03, 124.87, 124.38, 118.67, 118.11, 115.81, 109.31, 72.68, 37.83, 28.78, 11.70, 10.81. HRMS(ESI):m/z calcd for $C_{28}H_{25}N_4O_5S[M+H]^+$ 529.1467, found 529.1548.

N-(3-(3,5-dimethylisoxazol-4-yl)-5-((4-oxo-3,4-dihydrophthalazin-1-yl)methyl)phenyl)-1-phenylmethanesulfonamide (DDT76)

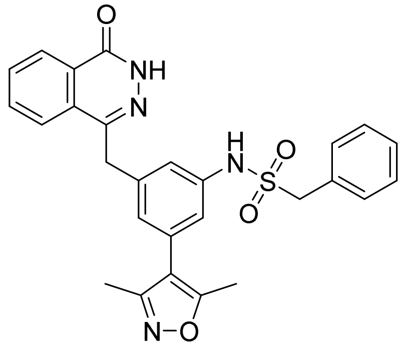

White solid, yield:27%; mp:158-159°C; ^{1}H NMR (400 MHz, DMSO-d_6) δ 12.64 (s, 1H), 9.85 (s, 1H), 8.29 (d, J = 7.5 Hz, 1H), 8.00 (d, J = 7.9 Hz, 1H), 7.90 (t, J = 7.1 Hz, 1H), 7.83 (t, J = 7.3 Hz, 1H), 7.27 (d, J = 7.2 Hz, 2H), 7.24 (s, 1H), 7.18 – 7.16 (m, 1H), 7.15 (d, J = 1.7 Hz, 1H), 7.11 (s, 1H), 7.07 (t, J = 1.8 Hz, 1H), 6.99 (t, J = 1.8 Hz, 1H), 4.41 (s, 2H), 4.35 (s, 2H), 2.37 (s, 3H), 2.19 (s, 3H). ^{13}C NMR (101 MHz, DMSO-d_6) δ 165.71, 159.90, 158.39, 145.38, 140.60, 139.57, 133.89, 132.00, 131.33, 131.26, 129.83, 129.68, 128.74, 128.63, 128.42, 126.54, 126.14, 124.51, 118.19, 117.60, 115.92, 57.58, 37.96, 11.84, 10.97. HRMS(ESI):m/z calcd for $C_{27}H_{25}N_4O_4S[M+H]^+$ 501.1518, found 501.1596.

N-(3-(3,5-dimethylisoxazol-4-yl)-5-((4-oxo-3,4-dihydrophthalazin-1-yl)methyl)phenyl)-1-(m-tolyl)methanesulfonamide (DDT77)

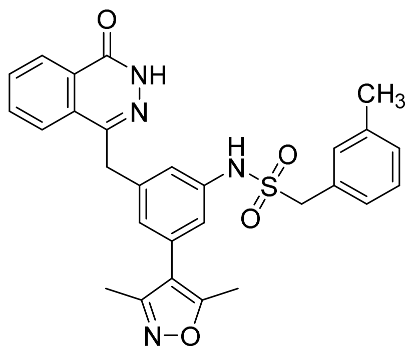

White solid, yield:43%; mp:154-155°C; ^{1}H NMR (400 MHz, DMSO-d_6) δ 12.63 (s, 1H), 9.85 (s, 1H), 8.28 (d, J = 8.9 Hz, 1H), 8.00 (d, J = 7.8 Hz, 1H), 7.89 (t, J = 7.6 Hz, 1H), 7.83 (t, J = 7.0 Hz, 1H), 7.10 (d, J = 7.1 Hz, 2H), 7.09 – 7.06 (m, 2H), 6.98 (s, 1H), 6.97 (d, J = 1.8 Hz, 1H), 6.93 (d, J = 7.2 Hz, 1H), 4.37 (s, 2H), 4.35 (s, 2H), 2.37 (s, 3H), 2.19 (s, 3H), 2.17 (s, 3H). ^{13}C NMR (101 MHz, DMSO-d_6) δ 165.67, 159.89, 158.37, 145.37, 140.56, 139.66, 137.90, 133.86, 131.98, 131.87, 131.23, 129.68, 129.64, 129.25, 128.63, 128.44, 126.54, 126.12, 124.41, 118.05, 117.48, 115.92, 57.67, 37.98, 21.23, 11.82, 10.95. HRMS(ESI):m/z calcd for $C_{28}H_{27}N_4O_4S[M+H]^+$ 515.1675, found 515.1750.

N-(3-(3,5-dimethylisoxazol-4-yl)-5-((4-oxo-3,4-dihydrophthalazin-1-yl)methyl)phenyl)-1-(p-tolyl)methanesulfonamide (DDT78)

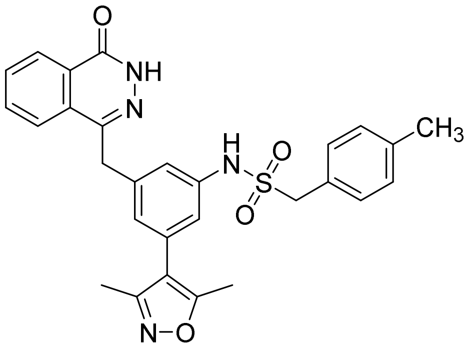

White solid, yield:84%; mp:161-162°C; ^{1}H NMR (400 MHz, DMSO-*d*6) δ 12.65 (s, 1H), 9.81 (s, 1H), 8.29 (d, *J* = 7.7 Hz, 1H), 8.03 – 7.96 (m, 1H), 7.90 (t, *J* = 6.9 Hz, 1H), 7.84 (t, *J* = 7.1 Hz, 1H), 7.11 (t, *J* = 1.5 Hz, 1H), 7.04 (d, *J* = 1.9 Hz, 1H), 7.02 (s, 4H), 6.95 (t, *J* = 1.8 Hz, 1H), 4.35 (s, 2H), 4.34 (s, 2H), 2.37 (s, 3H), 2.23 (s, 3H), 2.19 (s, 3H). ^{13}C NMR (101 MHz, DMSO-*d*6) δ 165.69, 159.90, 158.39, 145.39, 140.57, 139.61, 138.06, 133.90, 132.02, 131.22, 129.67, 129.31, 128.43, 126.75, 126.54, 126.15, 124.40, 118.11, 117.46, 115.93, 57.24, 37.94, 21.12, 11.83, 10.97. HRMS(ESI):m/z calcd for $C_{28}H_{27}N_4O_4S$ $[M+H]^+$515.1675, found 515.1756.

N-(3-(3,5-dimethylisoxazol-4-yl)-5-((4-oxo-3,4-dihydrophthalazin-1-yl)methyl)phenyl)-4-hydroxybenzenesulfonamide (DDT79)

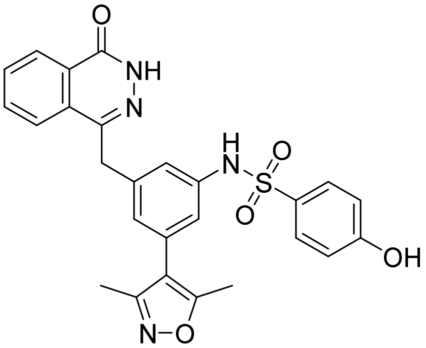

White solid, yield:93%; mp:238-239 °C; ^{1}H NMR (400 MHz, DMSO-*d*6) δ 12.65 (s, 1H), 10.44 (s, 1H), 10.14 (s, 1H), 8.31 – 8.25 (m, 1H), 7.91 – 7.88 (m, 1H), 7.86 – 7.83 (m, 1H), 7.83 – 7.80 (m, 1H), 7.49 (s, 1H), 7.47 (s, 1H), 7.07 (d, *J* = 1.6 Hz, 1H), 7.04 (t, *J* = 1.8 Hz, 1H), 6.82 (t, *J* = 1.8 Hz, 1H), 6.77 (s, 1H), 6.75 (s, 1H), 4.28 (s, 2H), 2.28 (s, 3H), 2.09 (s, 3H). ^{13}C NMR (101 MHz, DMSO-*d*6) δ 165.64, 161.78, 159.89, 158.28, 145.38, 140.48, 139.19, 133.83, 131.97, 131.07, 129.65, 129.55, 129.49, 128.39, 126.54, 126.03, 124.69, 118.50, 117.97, 115.99, 115.78, 37.85, 11.71, 10.83. HRMS(ESI): m/z calcd for $C_{26}H_{23}N_4O_5S$ $[M+H]^+$ 503.1311, found 503.1375.

N-(3-(3,5-dimethylisoxazol-4-yl)-5-((4-oxo-3,4-dihydrophthalazin-1-yl)methyl)phenyl)-2-(trifluoromethyl)benzenesulfonamide (DDT80)

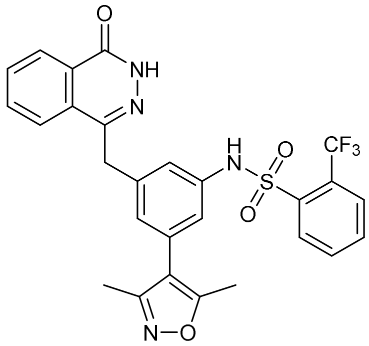

White solid, yield:29%; mp:197-198℃; ^{1}H NMR (400 MHz, DMSO-d_6) δ 12.62 (s, 1H), 10.71 (s, 1H), 8.32 – 8.23 (m, 1H), 7.97 (d, J = 7.5 Hz, 1H), 7.89 (d, J = 7.6 Hz, 1H), 7.87 – 7.84 (m, 1H), 7.83 (d, J = 3.6 Hz, 1H), 7.82 (d, J = 4.1 Hz, 1H), 7.77 (d, J = 7.4 Hz, 1H), 7.74 – 7.68 (m, 1H), 7.16 (s, 1H), 6.94 (d, J = 1.8 Hz, 1H), 6.88 (t, J = 1.8 Hz, 1H), 4.28 (s, 2H), 2.28 (s, 3H), 2.09 (s, 3H). ^{13}C NMR (101 MHz, DMSO-d$_6$) δ 165.73, 159.88, 158.24, 145.35, 143.55, 140.75, 138.19, 133.82, 133.06 (d, J = 32.4 Hz), 131.98, 131.32, 129.51, 128.35, 128.10, 126.96 (d, J = 3.8 Hz), 126.50, 125.99, 125.72, 123.73 (d, J = 273.0 Hz), 119.26, 118.75, 115.62, 37.71, 11.66, 10.76. HRMS(ESI):m/z calcd for $C_{27}H_{21}F_3N_4O_4S[M+H]^+$555.1236, found 555.1308.

N-(3-(3,5-dimethylisoxazol-4-yl)-5-((4-oxo-3,4-dihydrophthalazin-1-yl)methyl)phenyl)-3-(trifluoromethyl)benzenesulfonamide (DDT81)

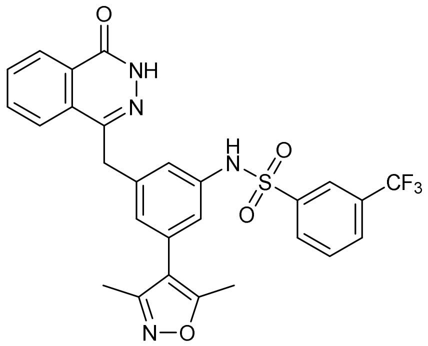

White solid, yield:58%; mp246-247:℃; ^{1}H NMR (400 MHz, DMSO-d_6) δ 12.62 (s, 1H), 10.46 (s, 1H), 8.31 – 8.25 (m, 1H), 7.94 (d, J = 8.0 Hz, 1H), 7.91 – 7.86 (m, 3H), 7.84 (d, J = 3.2 Hz, 1H), 7.82 (d, J = 3.3 Hz, 1H), 7.68 (t, J = 8.1 Hz, 1H), 7.18 (d, J = 1.6 Hz, 1H), 7.00 (t, J = 1.7 Hz, 1H), 6.87 (t, J = 1.8 Hz, 1H), 4.28 (s, 2H), 2.26 (s, 3H), 2.07 (s, 3H). ^{13}C NMR (101 MHz, DMSO-d_6) δ

165.72, 159.86, 158.22, 145.22, 140.79, 140.67, 138.12, 133.78, 131.97, 131.32, 131.25, 130.07(d,*J* = 65Hz) ,131.02, 130.10 (d,*J*=3.8Hz), 129.46, 128.38, 126.51, 126.11, 125.92, 123.53(d,*J*=4.5Hz), 122.61(d, *J* = 271Hz), 120.19, 119.53, 115.62, 37.73,11.59,10.69. HRMS(ESI):m/z calcd for $C_{27}H_{22}F_3N_4O_4S$ $[M+H]^+$ 555.1236, found 555.1312.

N-(3-(3,5-dimethylisoxazol-4-yl)-5-((4-oxo-3,4-dihydrophthalazin-1-yl)methyl)phenyl)-4-(trifluoromethyl)benzenesulfonamide (DDT82)

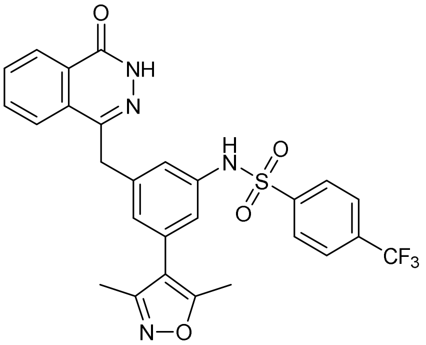

White solid, yield:55%; mp:193-194°C; ^{1}H NMR (400 MHz, DMSO-d_6) δ 12.67 (s, 1H), 10.61 (s, 1H), 8.30 – 8.25 (m, 1H), 7.94 – 7.90 (m, 1H), 7.87 (s, 4H), 7.85 – 7.84 (m, 1H), 7.84 – 7.81 (m, 1H), 7.16 (d, *J* = 1.5 Hz, 1H), 7.06 (t, *J* = 1.8 Hz, 1H), 6.82 (t, *J* = 1.8 Hz, 1H), 4.30 (s, 2H), 2.26 (s, 3H), 2.07 (s, 3H). ^{13}C NMR (101 MHz, DMSO-d_6) δ 165.73, 159.88, 158.24, 145.35, 143.55, 140.75, 138.19, 133.82, 133.06 (d, *J* = 32.4 Hz), 131.98, 131.32, 129.51, 128.35, 128.10, 126.96 (d, *J* = 3.8 Hz), 126.50, 125.99, 125.72,123.73(d,*J*=273.0Hz),119.26,118.75,115.62,37.71,11.66,10.76. HRMS(ESI):m/z calcd for $C_{27}H_{22}F_3N_4O_4S[M+H]^+$ 555.1236,found 555.1307.

N-(3-(3,5-dimethylisoxazol-4-yl)-5-((4-oxo-3,4-dihydrophthalazin-1-yl)methyl)phenyl)-4-nitrobenzenesulfonamide (DDT83)

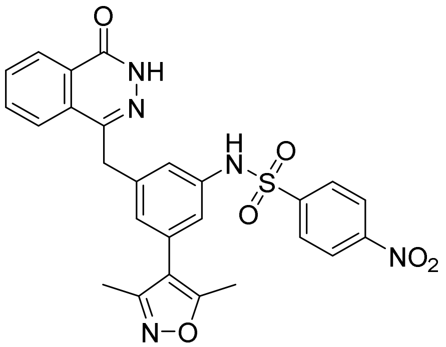

Yellow solid, yield:65%; mp:225-226°C;^{1}H NMR (400 MHz, DMSO-d_6) δ 12.62 (s, 1H), 10.69 (s, 1H), 8.26 (t, *J* = 7.8 Hz, 3H), 7.89 (d, *J* = 8.6 Hz, 3H), 7.84 – 7.77 (m, 2H), 7.17 (s, 1H), 6.98 (s, 1H), 6.90 (s, 1H), 4.28 (s, 2H), 2.30 (s, 3H), 2.11 (s, 3H). ^{13}C NMR (101 MHz, DMSO-d_6) δ 165.80, 159.84, 158.28, 150.22, 145.26, 145.15, 140.80, 138.01, 133.81, 131.94, 131.41, 129.48, 128.63, 128.33, 126.47, 125.96, 125.86, 125.03, 119.32, 118.97, 115.60, 37.68, 11.76, 10.86. HRMS(ESI):m/z calcd for $C_{26}H_{22}N_5O_6S$ $[M+H]^+$ 532.1213, found 532.1287.

N-(3-(3,5-dimethylisoxazol-4-yl)-5-((4-oxo-3,4-dihydrophthalazin-1-yl)methyl)phenyl)-2-fluorobenzenesulfonamide (DDT84)

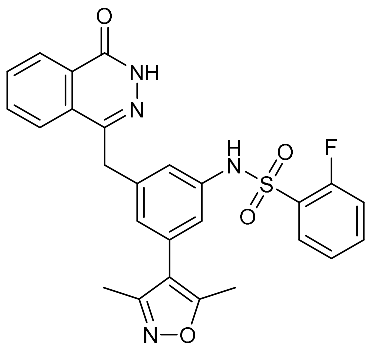

White solid, yield:64%; mp:133-134°C; ^{1}H NMR (400 MHz, DMSO-d_6) δ 12.63 (s, 1H), 10.68 (s, 1H), 8.31 – 8.26 (m, 1H), 7.90 – 7.83 (m, 1H), 7.84 – 7.81 (m, 2H), 7.73 – 7.67 (m, 1H), 7.65 – 7.57 (m, 1H), 7.30 (d, *J* = 10.5 Hz, 1H), 7.25 (t, *J* = 8.0 Hz, 1H), 7.12 (t, *J* = 1.5 Hz, 1H), 6.97 (t, *J* = 1.8 Hz, 1H), 6.90 (t, *J* = 1.8 Hz, 1H), 4.26 (s, 2H), 2.29 (s, 3H), 2.11 (s, 3H). ^{13}C NMR (101 MHz, DMSO-d_6) δ 165.73, 159.90, 158.48(d, *J* = 254Hz),158.28, 145.26, 140.64, 138.23, 136.37 (d, *J* = 8.6 Hz), 133.85, 131.97, 131.22, 130.77, 129.49, 128.39, 127.23 (d, *J* = 13.6 Hz), 126.50, 125.96, 125.28, 125.27(d, *J* = 7 Hz),118.45, 118.11, 117.65 (d, *J* = 20.9 Hz), 115.70, 37.75, 11.73, 10.83. HRMS(ESI):m/z calcd for $C_{26}H_{22}FN_4O_4S[M+H]^+$ 505.1268, found 505.1342.

N-(3-(3,5-dimethylisoxazol-4-yl)-5-((4-oxo-3,4-dihydrophthalazin-1-yl)methyl)phenyl)-3-fluorobenzenesulfonamide (DDT85)

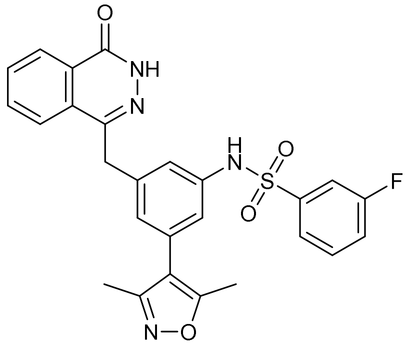

White solid, yield:60%; mp:137-138°C; ^{1}H NMR (400 MHz, DMSO-d_6) δ 12.62 (s, 1H), 10.43 (s, 1H), 8.30 – 8.26 (m, 1H), 7.91 – 7.87 (m, 1H), 7.84 (d, J = 1.4 Hz, 1H), 7.84 – 7.81 (m, 1H), 7.53 – 7.46 (m, 2H), 7.47 – 7.41 (m, 1H), 7.44 – 7.37 (m, 1H), 7.16 (d, J = 1.6 Hz, 1H), 6.99 (t, J = 1.8 Hz, 1H), 6.88 (t, J = 1.8 Hz, 1H), 4.28 (s,2H), 2.28 (s, 3H), 2.10 (s, 3H). ^{13}C NMR (101 MHz, DMSO-d_6) δ 165.73, 162.01 (d, J = 249.0 Hz), 159.87, 158.26, 145.26, 141.68 (d, J = 6.8 Hz), 140.70, 138.33, 133.80, 132.00 (d, J = 7.3 Hz), 131.28, 129.49, 128.40, 126.52, 125.95, 125.74, 123.34, 123.31, 120.59 (d, J = 20.9 Hz), 119.62, 119.15, 115.68, 114.02 (d, J = 24.4 Hz), 37.77, 11.68, 10.78. HRMS(ESI):m/z calcd for $C_{26}H_{22}FN_4O_4S[M+H]^+$ 505.1268, found 505.1345.

N-(3-(3,5-dimethylisoxazol-4-yl)-5-((4-oxo-3,4-dihydrophthalazin-1-yl)methyl)phenyl)-4-fluorobenzenesulfonamide (DDT86)

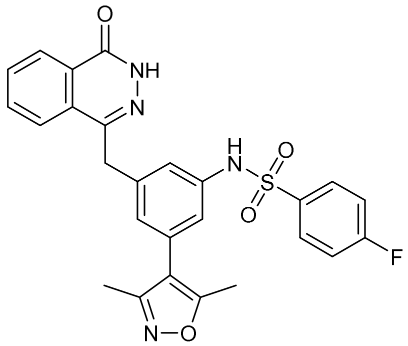

White solid, yield:52%; mp:151-152°C; ^{1}H NMR (400 MHz, DMSO-d_6) δ 12.64 (s, 1H), 10.38 (s, 1H), 8.30 – 8.26 (m, 1H), 7.90 – 7.86 (m, 1H), 7.85 (d, J = 2.2 Hz, 1H), 7.84 – 7.82 (m, 1H), 7.71 (d, J = 5.1 Hz, 1H), 7.69 (d, J = 5.1 Hz, 1H), 7.28 (t, J = 8.8 Hz, 2H), 7.14 (t, J = 1.5 Hz, 1H), 6.98 (t, J = 1.8 Hz, 1H), 6.86 (t, J = 1.8 Hz, 1H), 4.28 (s, 2H), 2.29 (s, 3H), 2.11 (s, 3H). ^{13}C NMR (101 MHz, DMSO-d_6) δ 165.72,164.7(d, J = 250Hz), 159.89, 158.28, 145.33, 140.65, 138.58, 136.06 (d, J = 3.1 Hz), 133.83, 131.98, 131.25, 130.12 (d, J = 9.6 Hz), 129.50, 128.37, 126.51, 125.99, 125.40, 119.05,

118.70, 116.82 (d, J = 22.8 Hz), 115.70, 37.77, 11.71, 10.82. HRMS(ESI):m/z calcd for $C_{26}H_{22}FN_4O_4S[M+H]^+$ 505.1268, found 505.1338.

2-chloro-N-(3-(3,5-dimethylisoxazol-4-yl)-5-((4-oxo-3,4-dihydrophthalazin-1-yl)methyl)phenyl)benzenesulfonamide (DDT87)

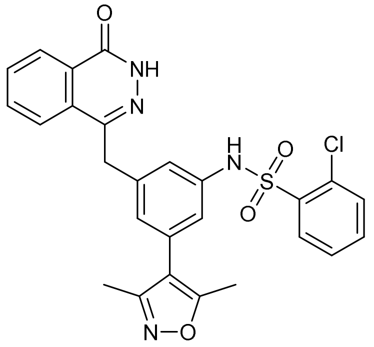

White solid, yield:66%; mp:174-175°C; ^{1}H NMR (400 MHz, DMSO-d_6) δ 12.63 (s, 1H), 10.66 (s, 1H), 8.33 – 8.24 (m, 1H), 7.90 (d, J = 7.9 Hz, 1H), 7.84 (d, J = 3.5 Hz, 1H), 7.83 (s, 1H), 7.83 – 7.80 (m, 1H), 7.57 – 7.47 (m, 2H), 7.43 – 7.37 (m, 1H), 7.11 (t, J = 1.5 Hz, 1H), 6.97 (t, J = 1.8 Hz, 1H), 6.88 (t, J = 1.8 Hz, 1H), 4.25 (s, 2H), 2.28 (s, 3H), 2.10 (s, 3H). ^{13}C NMR (101 MHz, DMSO-d_6) δ 165.73, 159.91, 158.28, 145.25, 140.64, 138.09, 136.63, 135.06, 133.85, 132.23, 131.98, 131.96, 131.20, 131.09, 129.49, 128.39, 128.04, 126.50, 125.97, 125.04, 118.03, 117.70, 115.69, 37.74, 11.76, 10.86. HRMS(ESI):m/z calcd for $C_{26}H_{22}ClN_4O_4S[M+H]^+$521.0972, found 521.1046.

3-chloro-N-(3-(3,5-dimethylisoxazol-4-yl)-5-((4-oxo-3,4-dihydrophthalazin-1-yl)methyl)phenyl)benzenesulfonamide (DDT88)

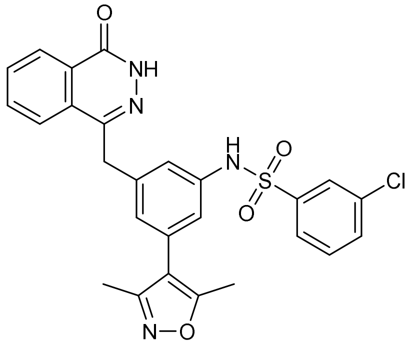

Yellow solid, yield:73%; mp:142-143°C; ^{1}H NMR (400 MHz, DMSO-d_6) δ 12.63 (s, 1H), 10.43 (s,1H), 8.32 – 8.24 (m, 1H), 7.92 – 7.88 (m, 1H), 7.85 (d, J = 2.2 Hz, 1H), 7.84 – 7.82 (m, 1H), 7.65

(t, J = 1.9 Hz, 1H), 7.64 – 7.60 (m, 1H), 7.57 (d, J = 8.0 Hz, 1H), 7.46 (t, J = 7.9 Hz, 1H), 7.17 (d, J = 1.6 Hz, 1H), 7.00 (t, J = 1.8 Hz, 1H), 6.87 (t, J = 1.8 Hz, 1H), 4.29 (s, 2H), 2.29 (s, 3H), 2.10 (s, 3H). ^{13}C NMR (101 MHz, DMSO-d_6) δ 165.74, 159.88, 158.26, 145.26, 141.44, 140.75, 138.27, 134.29, 133.83, 133.36, 131.99, 131.63, 131.30, 129.49, 128.40, 126.59, 126.53, 125.95, 125.87, 125.73, 119.78, 119.20, 115.68, 37.76, 11.68, 10.78. HRMS(ESI):m/z calcd for $C_{26}H_{22}ClN_4O_4S$ $[M+H]^+$521.0972, found 521.1048.

4-chloro-N-(3-(3,5-dimethylisoxazol-4-yl)-5-((4-oxo-3,4-dihydrophthalazin-1-yl)methyl)phenyl)benzenesulfonamide (DDT89)

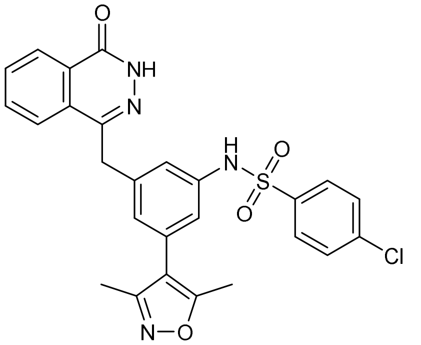

White solid, yield:93%; mp:147-148℃; ^{1}H NMR (400 MHz, DMSO-d_6) δ 12.65 (s, 1H), 10.44 (s, 1H), 8.32 – 8.26 (m, 1H), 7.91 – 7.87 (m, 1H), 7.85 (d, J = 2.1 Hz, 1H), 7.84 – 7.82 (m, 1H), 7.64 (d, J = 1.8 Hz, 1H), 7.63 (d, J = 1.9 Hz, 1H), 7.53 (d, J = 1.9 Hz, 1H), 7.52 (d, J = 1.8 Hz, 1H), 7.14 (t, J = 1.6 Hz, 1H), 7.00 (t, J = 1.8 Hz, 1H), 6.84 (t, J = 1.8 Hz, 1H), 4.28 (s, 2H), 2.28 (s, 3H), 2.10 (s, 3H). ^{13}C NMR (101 MHz, DMSO-d_6) δ 165.73, 159.89, 158.27, 145.34, 140.69, 138.51, 138.45, 138.34, 133.84, 131.99, 131.27, 129.82, 129.50, 128.99, 128.37, 126.53, 126.00, 125.47, 119.02, 118.64, 115.66, 37.75, 11.71, 10.82. HRMS(ESI):m/z calcd for $C_{26}H_{22}ClN_4O_4S$ $[M+H]^+$ 521.0972, found 521.1048.

2,4-dichloro-N-(3-(3,5-dimethylisoxazol-4-yl)-5-((4-oxo-3,4-dihydrophthalazin-1-yl)methyl)phenyl)benzenesulfonamide (DDT90)

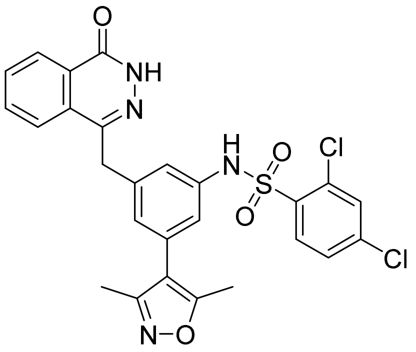

White solid, yield:74%; mp:151-152℃; ^{1}H NMR (400 MHz, DMSO-d_6) δ 12.65 (s, 1H), 10.78 (s, 1H), 8.30 – 8.27 (m, 1H), 7.88 (d, J = 8.6 Hz, 1H), 7.86 – 7.84 (m, 1H), 7.83 (d, J = 3.5 Hz, 1H), 7.82 (d, J = 3.8 Hz, 1H), 7.71 (d, J = 2.1 Hz, 1H), 7.48 (d, J = 8.5 Hz, 1H), 7.14 (t, J = 1.5 Hz, 1H), 6.98 (t, J = 1.8 Hz, 1H), 6.88 (t, J = 1.8 Hz, 1H), 4.26 (s, 2H), 2.30 (s, 3H), 2.12 (s, 3H). ^{13}C NMR (101 MHz, DMSO-d_6) δ 165.76, 159.91, 158.28, 145.28, 140.73, 139.16, 137.86, 135.67, 133.83, 133.24, 132.37, 131.98, 131.77, 131.31, 129.47, 128.37, 128.21, 126.52, 125.96, 125.22, 118.03, 117.79, 115.65,37.71,11.76,10.86. HRMS(ESI):m/z calcd for $C_{26}H_{21}Cl_2N_4O_4S[M+H]^+$ 555.0582, found 555.0662.

2-bromo-N-(3-(3,5-dimethylisoxazol-4-yl)-5-((4-oxo-3,4-dihydrophthalazin-1-yl)methyl)phenyl)benzenesulfonamide (DDT91)

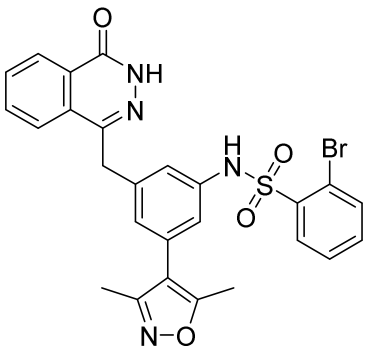

White solid, yield:43%; mp:171-172℃; ^{1}H NMR (400 MHz, DMSO-d_6) δ 12.64 (s, 1H), 10.66 (s, 1H), 8.33 – 8.24 (m, 1H), 7.97 – 7.91 (m, 1H), 7.88 – 7.83 (m, 2H), 7.85 – 7.79 (m, 1H), 7.72 – 7.65 (m, 1H), 7.48 – 7.41 (m, 2H), 7.11 (d, J = 1.5 Hz, 1H), 6.97 (t, J = 1.8 Hz, 1H), 6.88 (t, J = 1.9 Hz, 1H), 4.25 (s, 2H), 2.29 (s, 3H), 2.11 (s, 3H). ^{13}C NMR (101 MHz, DMSO-d_6) δ 165.72, 159.92, 158.29, 145.25, 140.61, 138.38, 138.13, 135.81, 134.97, 133.85, 132.18, 131.98, 131.19, 129.50,

128.55, 128.39, 126.50, 125.97, 124.95, 119.59, 118.01, 117.69, 115.71, 37.76, 11.77, 10.87. HRMS(ESI):m/z calcd for $C_{26}H_{22}BrN_4O_4S[M+H]^+$ 565.0467, found 565.0540.

3-bromo-N-(3-(3,5-dimethylisoxazol-4-yl)-5-((4-oxo-3,4-dihydrophthalazin-1-yl)methyl)phenyl)benzenesulfonamide (DDT92)

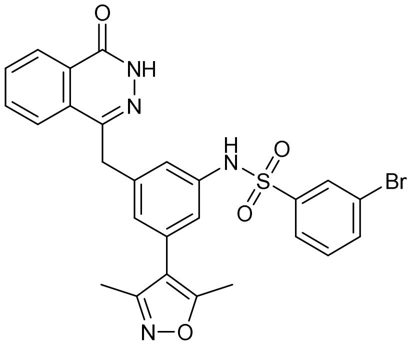

White solid, yield:76%; mp:147-148℃; ^{1}H NMR (400 MHz, DMSO-d_6) δ 12.62 (s, 1H), 10.41 (s, 1H), 8.30 – 8.26 (m, 1H), 7.91 – 7.87 (m, 1H), 7.85 (d, J = 2.2 Hz, 1H), 7.84 – 7.82 (m, 1H), 7.78 (d, J = 1.7 Hz, 1H), 7.77 – 7.73 (m,1H), 7.61 (d, J = 8.5 Hz, 1H), 7.39 (t, J = 7.9 Hz, 1H), 7.16 (t, J = 1.6 Hz, 1H), 7.00 (t, J = 1.8 Hz, 1H), 6.87 (t, J = 1.8 Hz, 1H), 4.29 (s, 2H), 2.28 (s, 3H), 2.10 (s, 3H). ^{13}C NMR (101 MHz, DMSO-d_6) δ 165.73, 159.88, 158.26, 145.26, 141.56, 140.75, 138.28, 136.24, 133.85, 132.00, 131.82, 131.29, 129.49, 129.39, 128.40, 126.53, 126.06, 125.95, 125.86, 122.54, 119.82, 119.17, 115.68, 37.77, 11.70, 10.80. HRMS(ESI):m/z calcd for $C_{26}H_{22}BrN_4O_4S$ $[M+H]^+$ 565.0467, found 565.0541.

4-bromo-N-(3-(3,5-dimethylisoxazol-4-yl)-5-((4-oxo-3,4-dihydrophthalazin-1-yl)methyl)phenyl)benzenesulfonamide (DDT93)

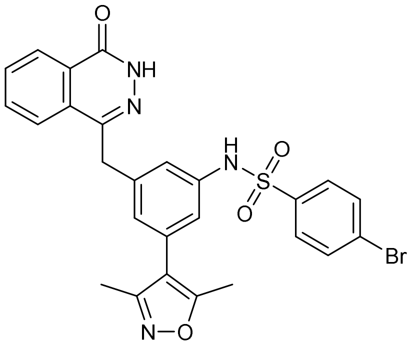

White solid, yield:66%; mp:151-152°C; ^{1}H NMR (400 MHz, DMSO-d_6) δ 12.65 (s, 1H), 10.45 (s, 1H), 8.31 – 8.27 (m, 1H), 7.91 – 7.88 (m, 1H), 7.85 (d, J = 2.1 Hz, 1H), 7.85 – 7.82 (m, 1H), 7.68 (d, J = 1.9 Hz, 1H), 7.66 (d, J = 2.0 Hz, 1H), 7.57 (d, J = 2.0 Hz, 1H), 7.55 (d, J = 1.9 Hz, 1H), 7.14 (t, J = 1.5 Hz, 1H), 7.02 (t, J = 1.8 Hz, 1H), 6.83 (t, J = 1.8 Hz, 1H), 4.29 (s, 2H), 2.28 (s, 3H), 2.10 (s, 3H). ^{13}C NMR (101 MHz, DMSO-d_6) δ 165.73, 159.89, 158.27, 145.34, 140.69, 138.94, 138.45, 133.84, 132.77, 131.99, 131.28, 129.51, 129.06, 128.37, 127.34, 126.54, 125.99, 125.46, 119.03, 118.63, 115.67, 37.76, 11.71, 10.81. HRMS(ESI):m/z calcd for $C_{26}H_{22}BrN_4O_4S$[M+H]$^+$ 565.0467, found 565.0543.

N-(3-(3,5-dimethylisoxazol-4-yl)-5-((4-oxo-3,4-dihydrophthalazin-1-yl)methyl)phenyl)-3-(methylsulfonyl)benzenesulfonamide (DDT94)

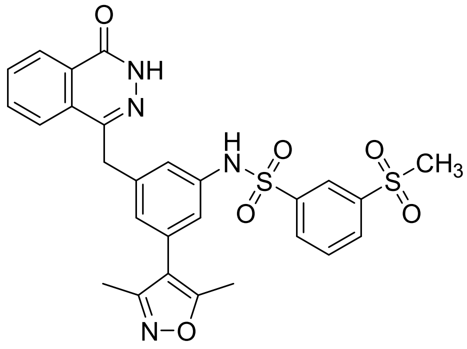

White solid, yield:70%; mp:243-244°C; ^{1}H NMR (400 MHz, DMSO-d_6) δ 12.61 (s, 1H), 10.52 (s, 1H), 8.30 – 8.25 (m, 1H), 8.21 (t, J = 1.8 Hz, 1H), 8.16 (d, J = 7.9 Hz, 1H), 7.92 (d, J = 8.2 Hz, 1H), 7.89 – 7.87 (m, 1H), 7.87 – 7.84 (m, 1H), 7.83 – 7.80 (m, 1H), 7.73 (t, J = 7.9 Hz, 1H), 7.14 (d, J = 1.6 Hz, 1H), 7.00 (t, J = 1.8 Hz, 1H), 6.88 (t, J = 1.8 Hz, 1H), 4.28 (s, 2H), 3.24 (s, 3H), 2.26 (s, 3H), 2.08 (s,3H). ^{13}C NMR (101 MHz, DMSO-d_6) δ 165.76, 159.87, 158.27, 145.26, 142.36, 140.80, 140.77, 138.13, 133.91, 132.03, 131.87, 131.72, 131.35, 131.33, 129.52, 128.35, 126.52, 125.99, 125.96, 125.39, 120.07, 119.34, 115.62, 43.68, 37.70, 11.70, 10.80. HRMS(ESI):m/z calcd for $C_{27}H_{25}N_4O_6S_2$[M+H]$^+$ 565.1137, found 565.1213.

5 ^{1}H-NMR, ^{13}C-NMR Spectra of Target Compounds DDT01-DDT94

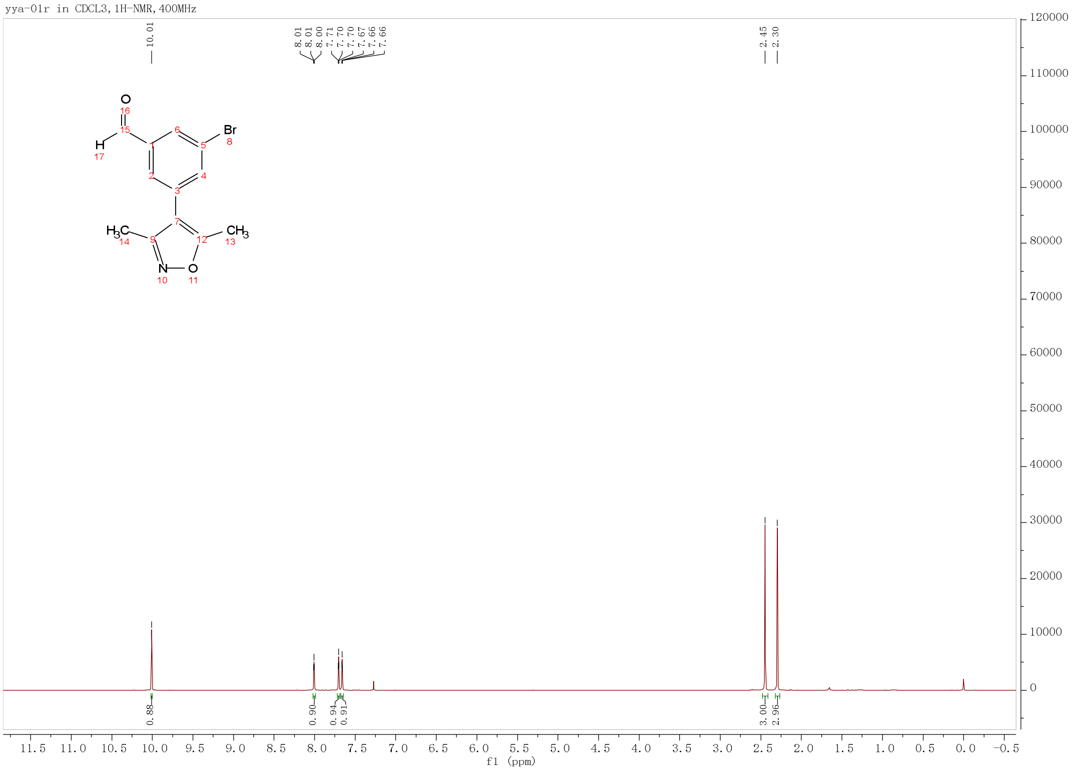

^{1}H-NMR spectrum of compound 10

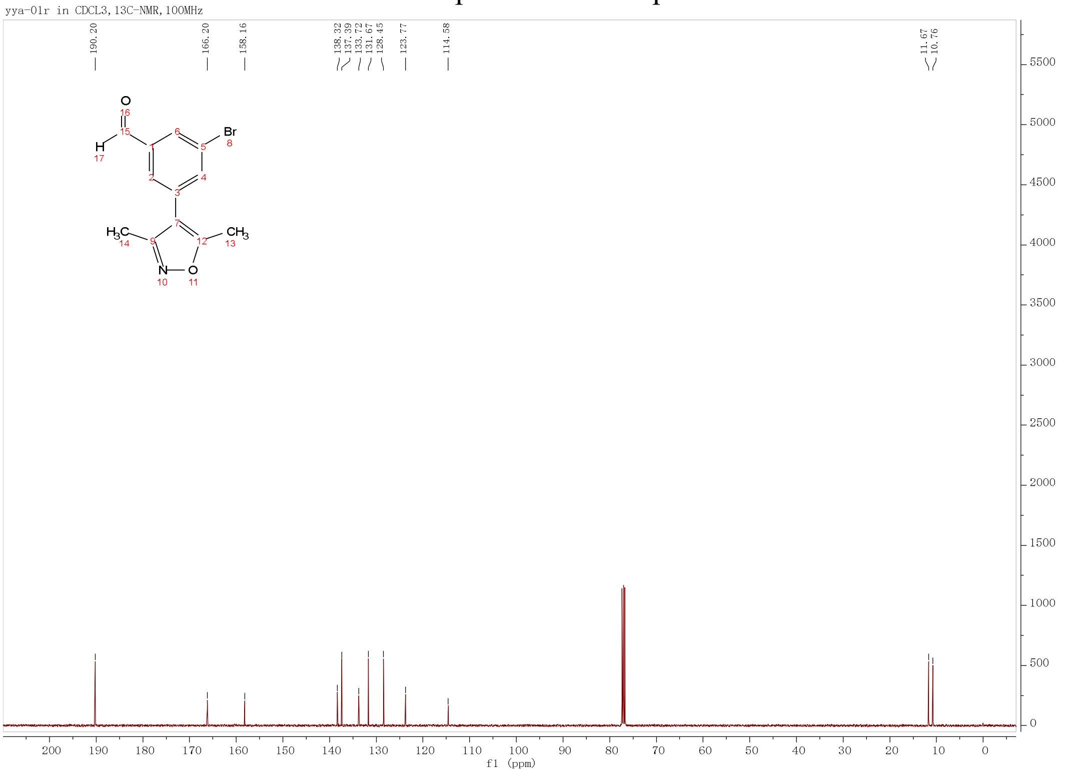

^{13}C-NMR spectrum of compound 10

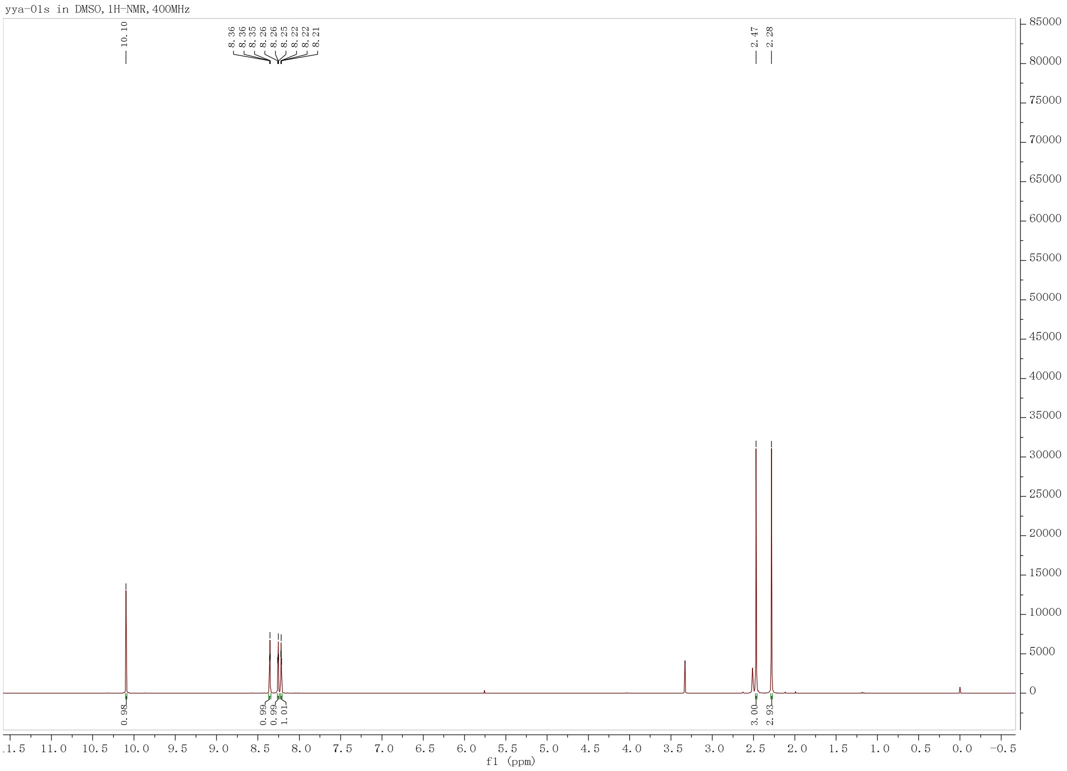

^{1}H-NMR spectrum of compound 11

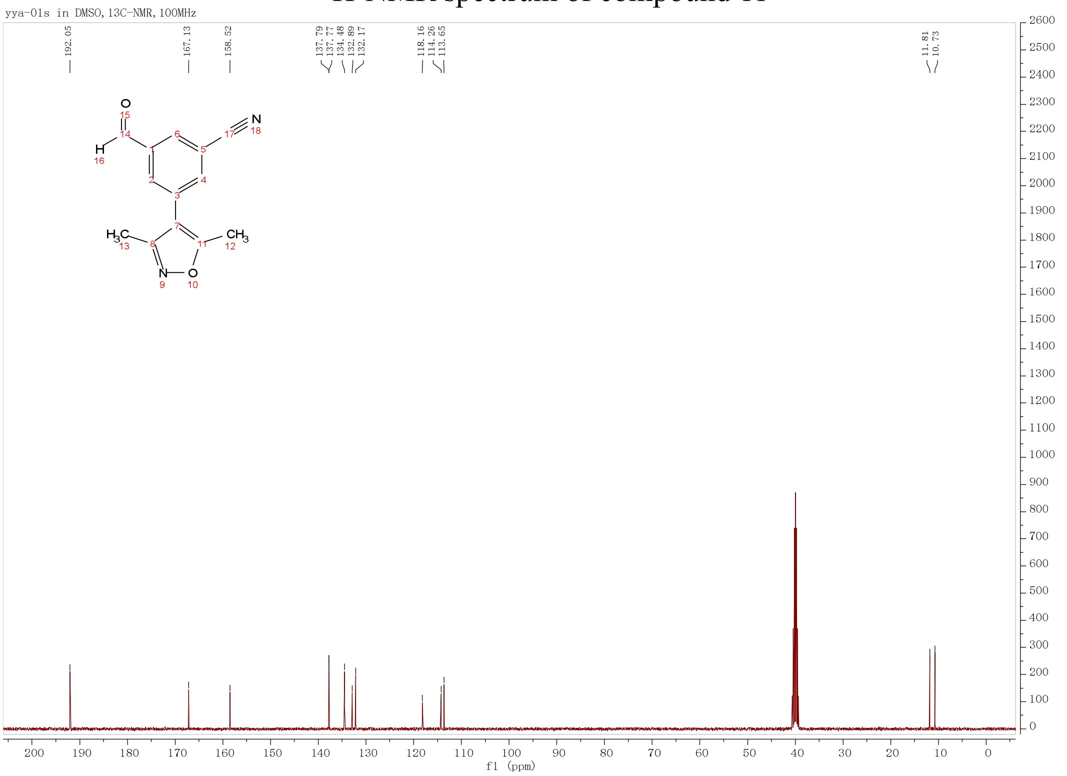

^{13}C-NMR spectrum of compound 11

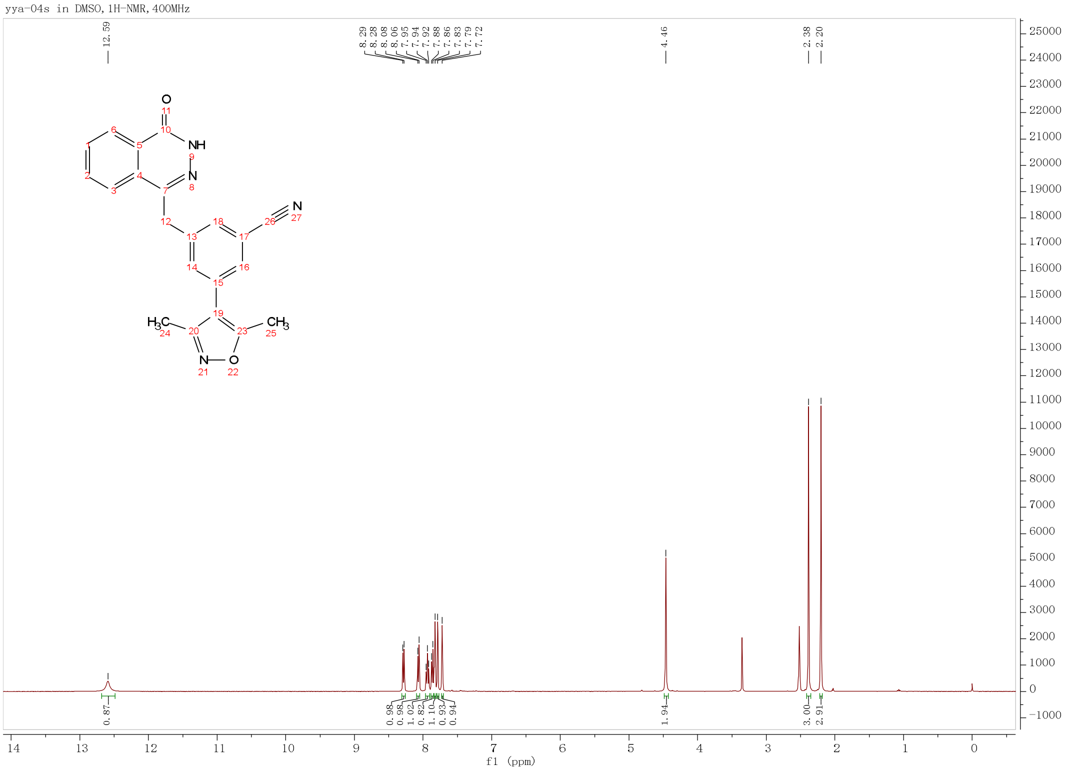

^{1}H-NMR spectrum of compound 13

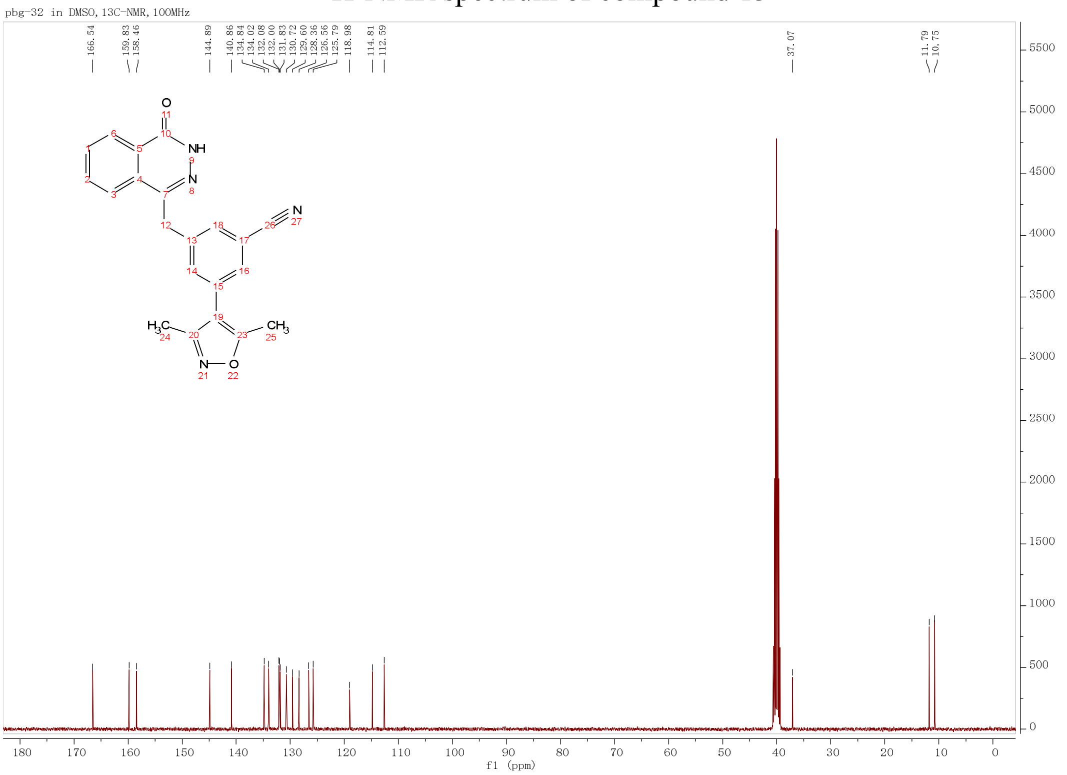

^{13}C-NMR spectrum of compound 13

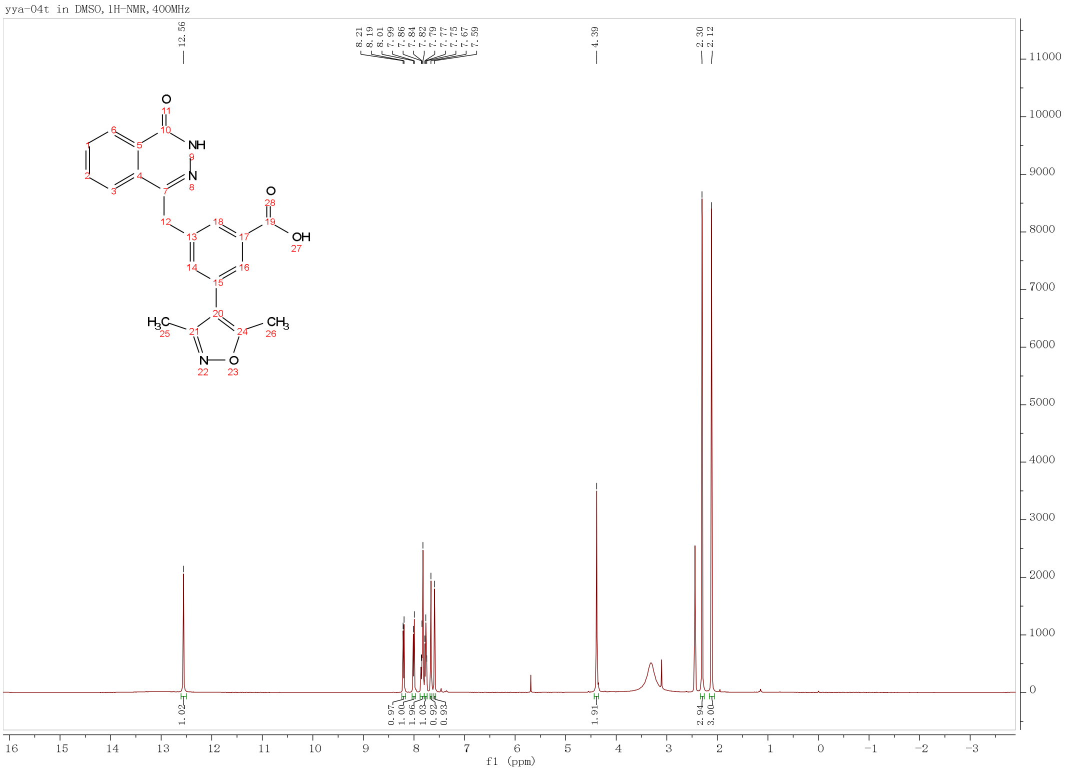

^{1}H-NMR spectrum of compound 14

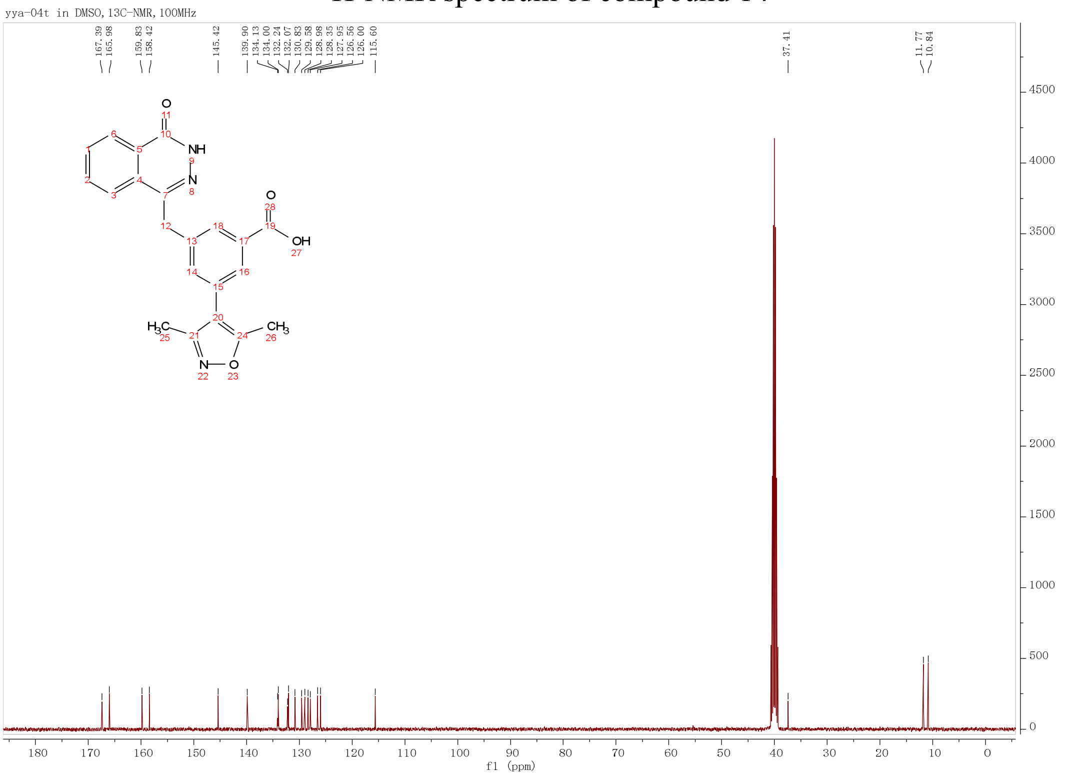

^{13}C-NMR spectrum of compound 14

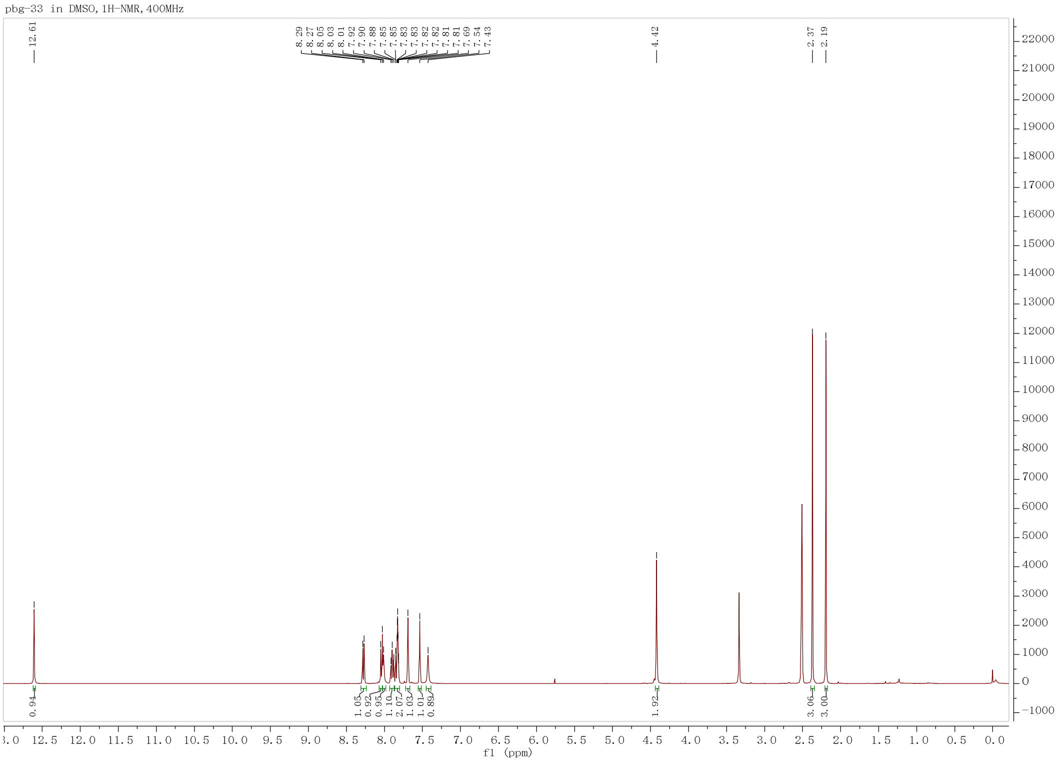

^{1}H-NMR spectrum of compound DDT01

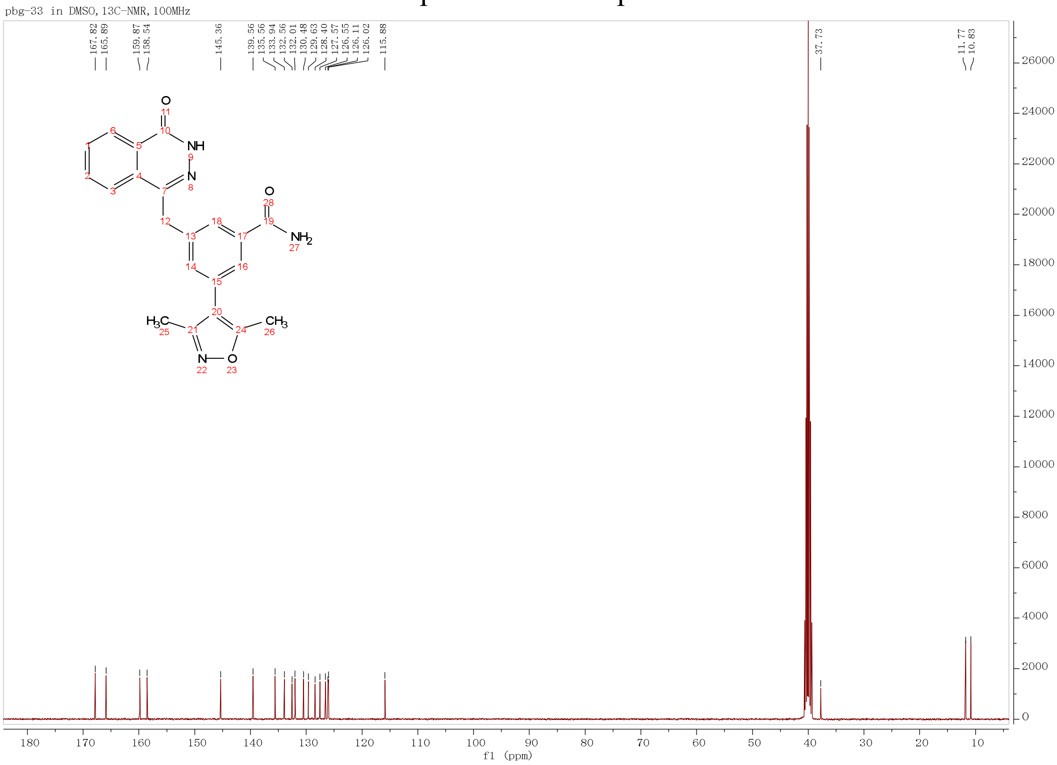

^{13}C-NMR spectrum of compound DDT01

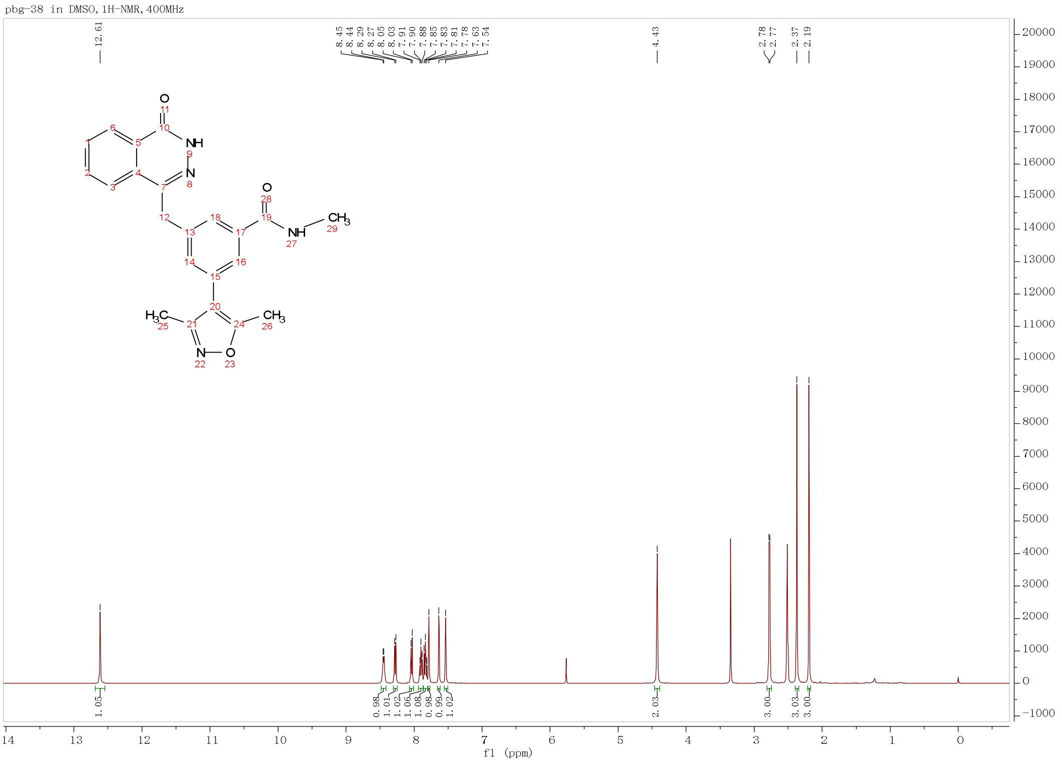

^{1}H-NMR spectrum of compound DDT02

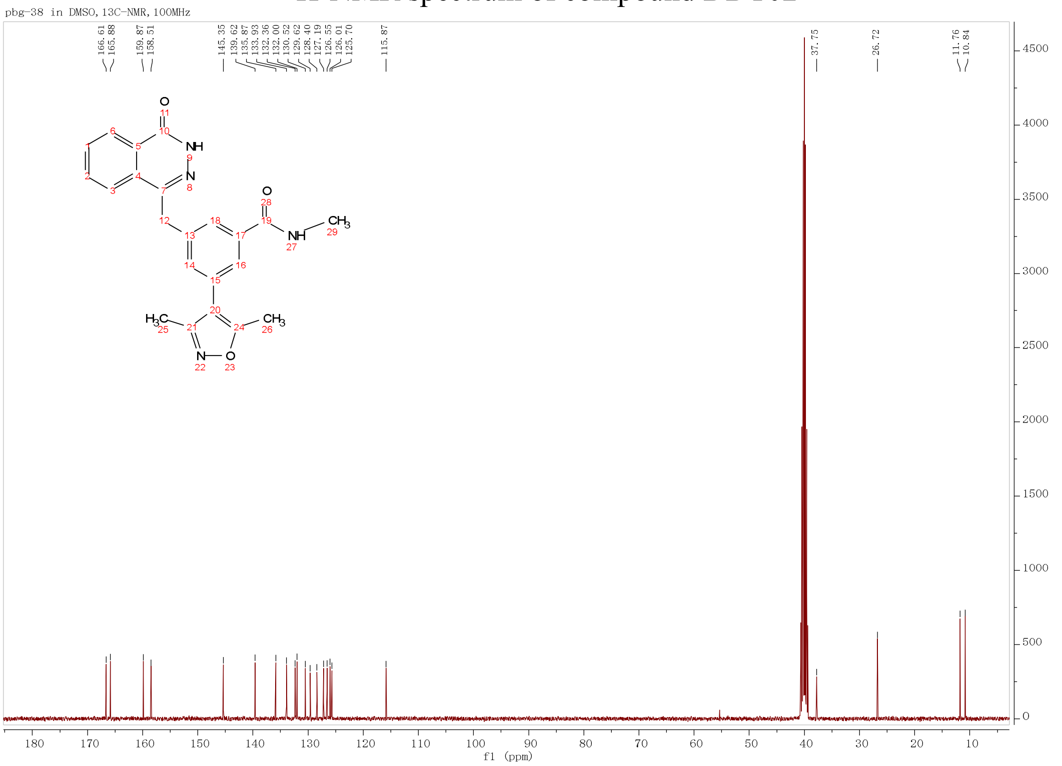

^{13}C-NMR spectrum of compound DDT02

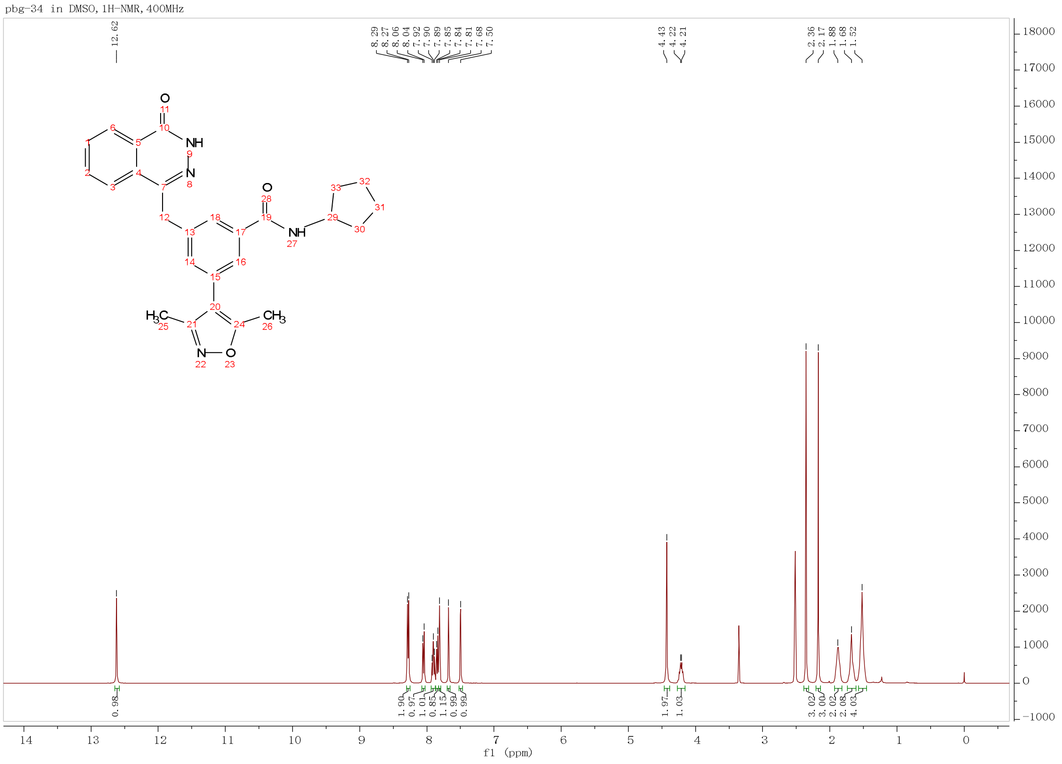

^{1}H-NMR spectrum of compound DDT03

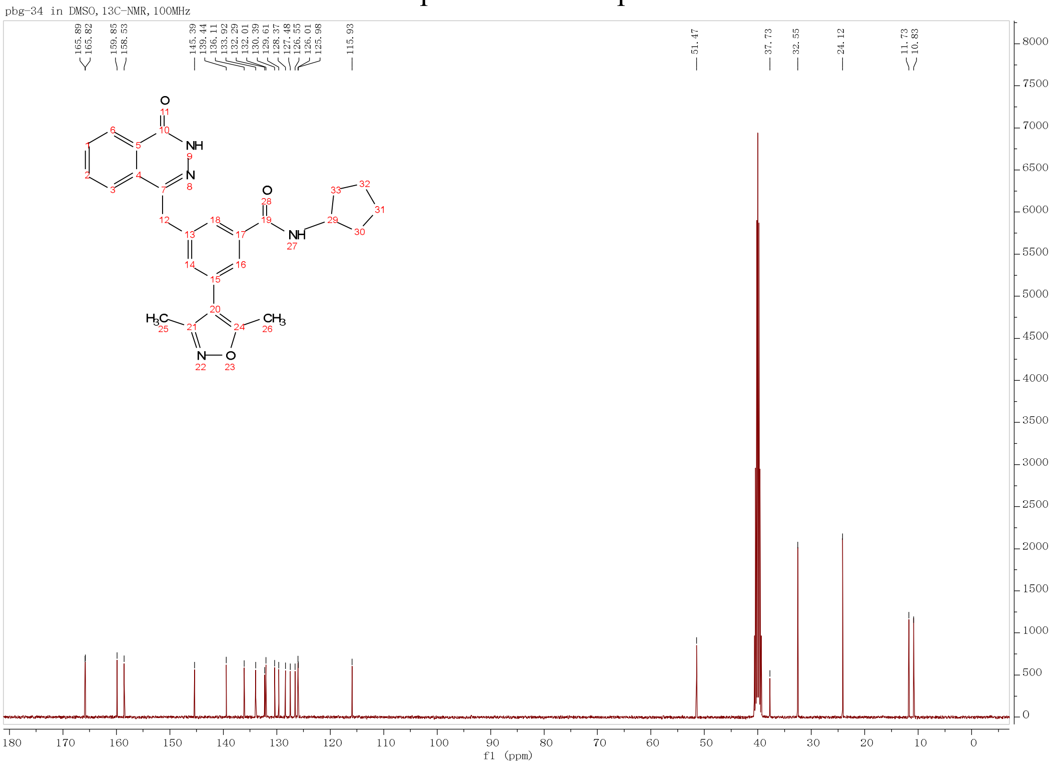

^{13}C-NMR spectrum of compound DDT03

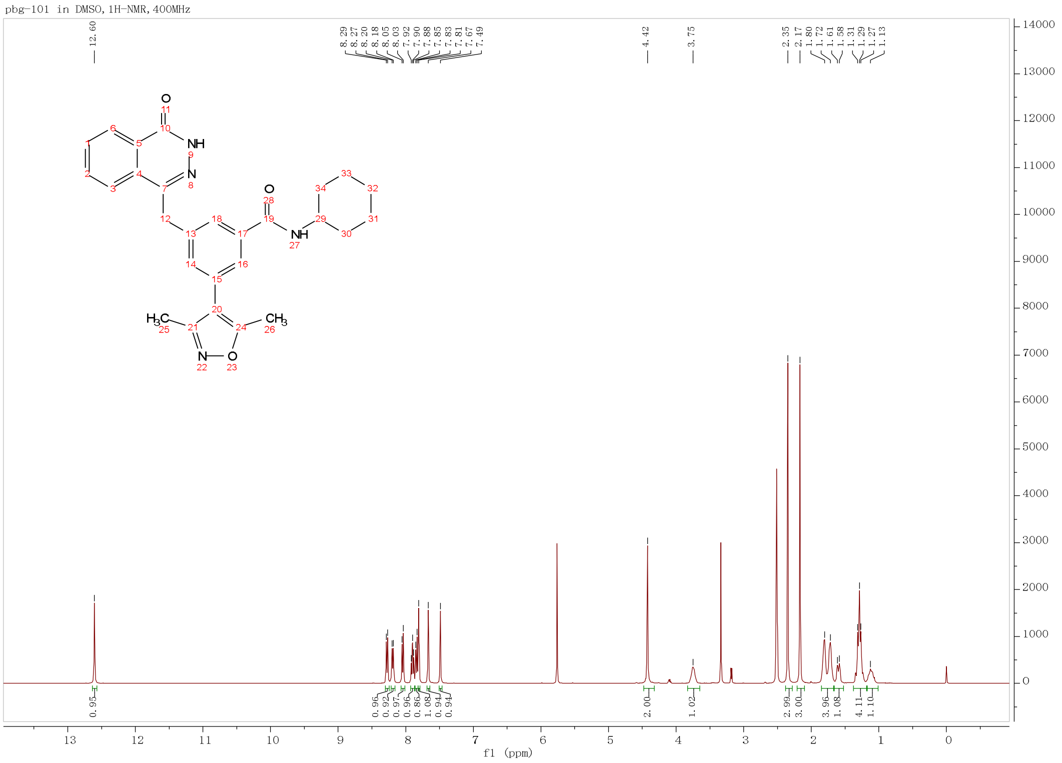

^{1}H-NMR spectrum of compound DDT04

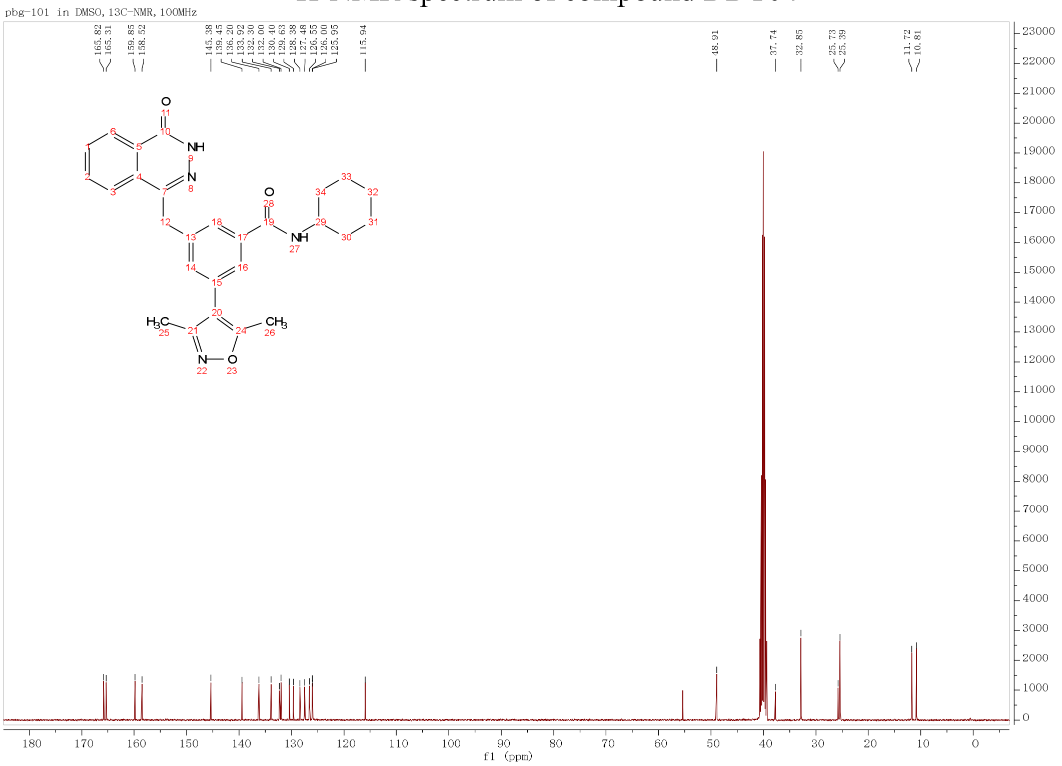

^{13}C-NMR spectrum of compound DDT04

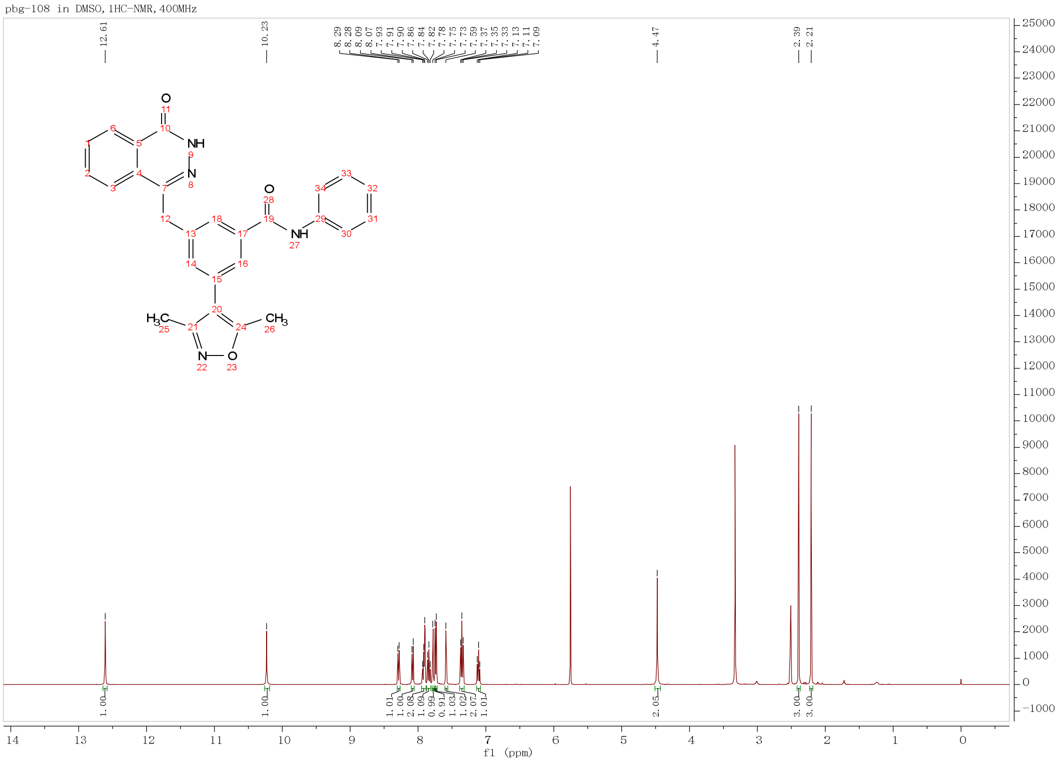

^{1}H-NMR spectrum of compound DDT05

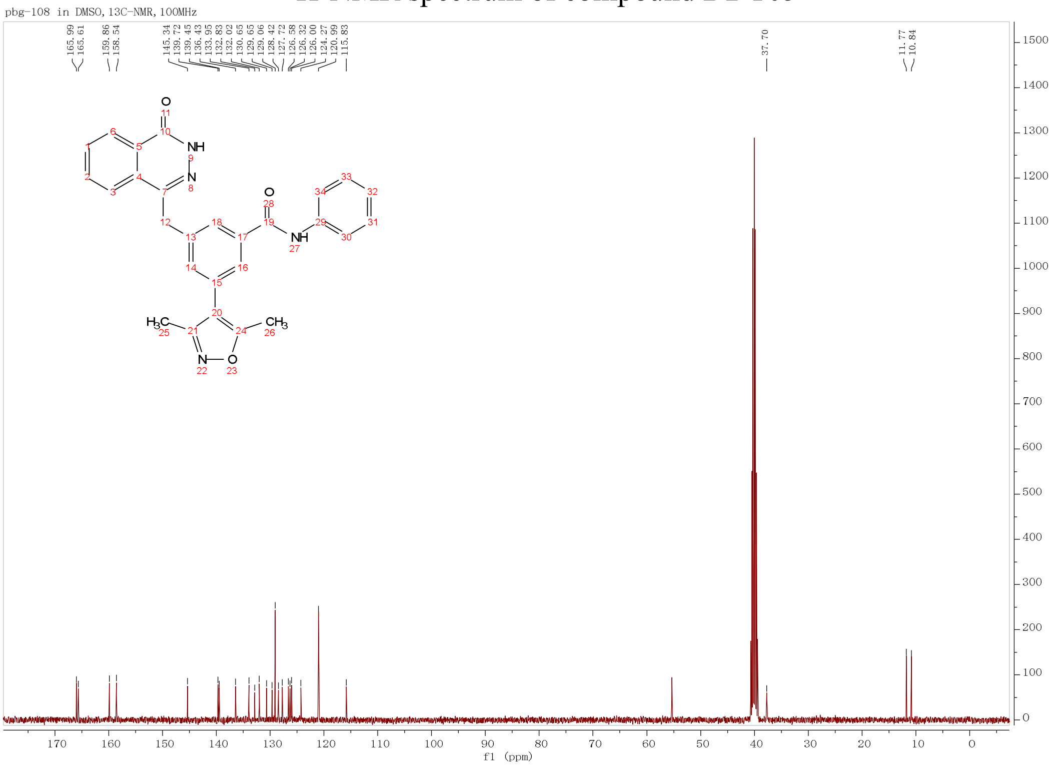

^{13}C-NMR spectrum of compound DDT05

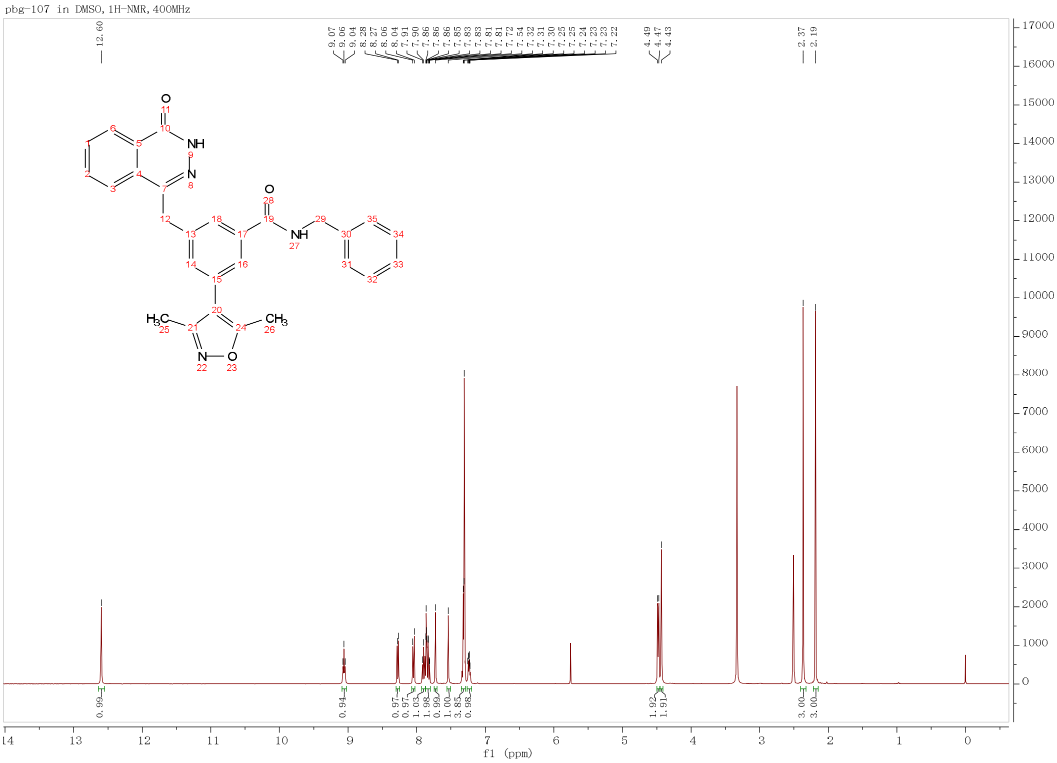

^{1}H-NMR spectrum of compound DDT06

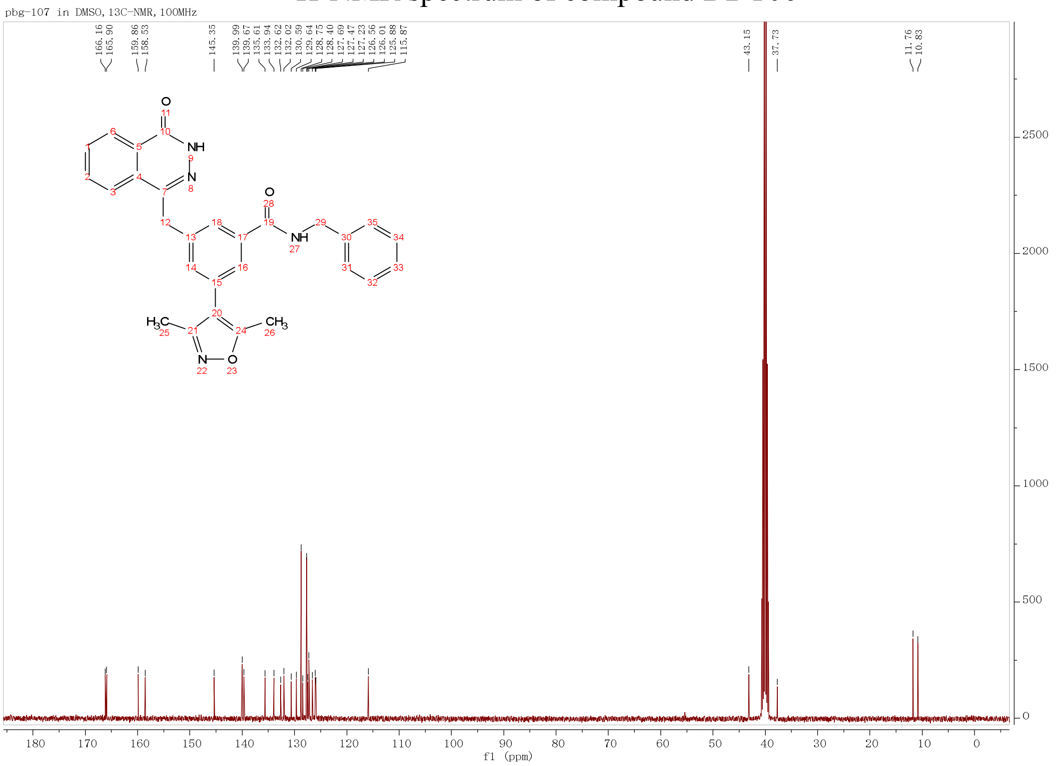

^{13}C-NMR spectrum of compound DDT06

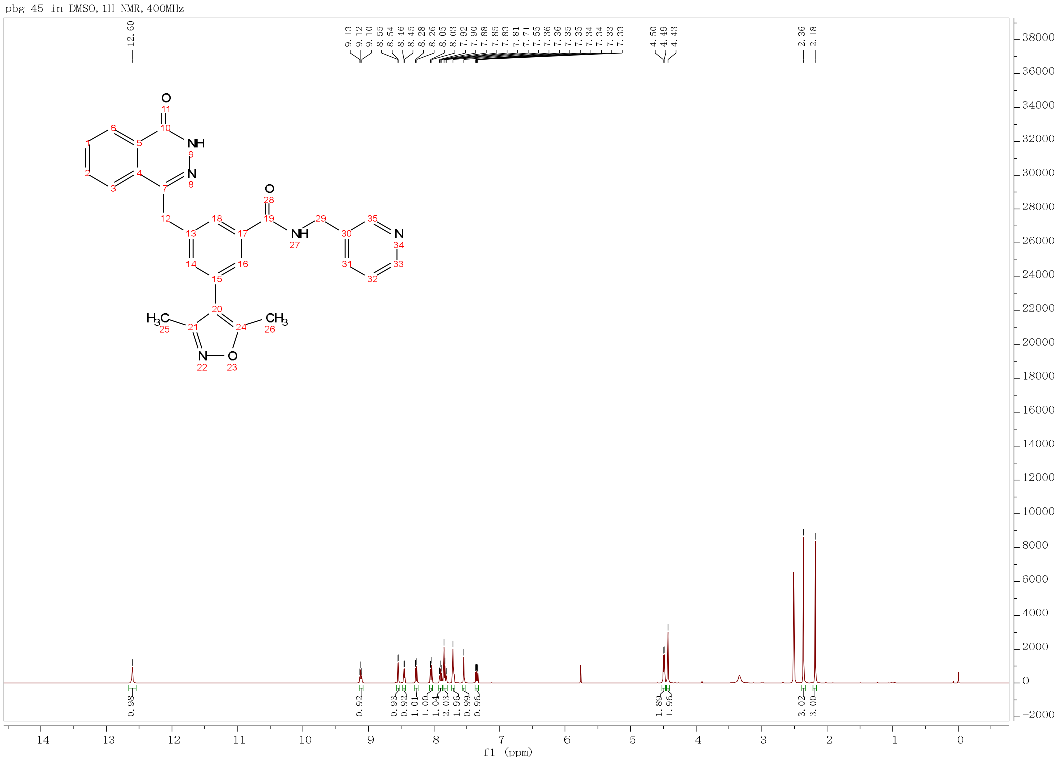

^{1}H-NMR spectrum of compound DDT07

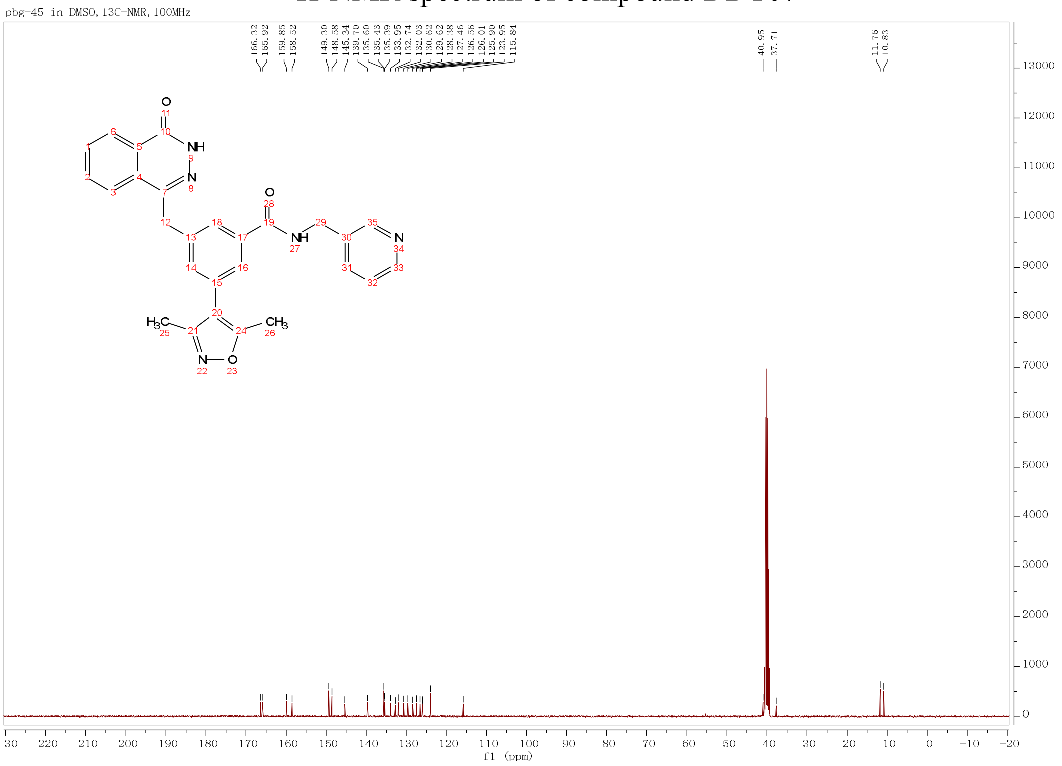

^{13}C-NMR spectrum of compound DDT07

pbg-43 in DMSO, 1H-NMR, 400MHz

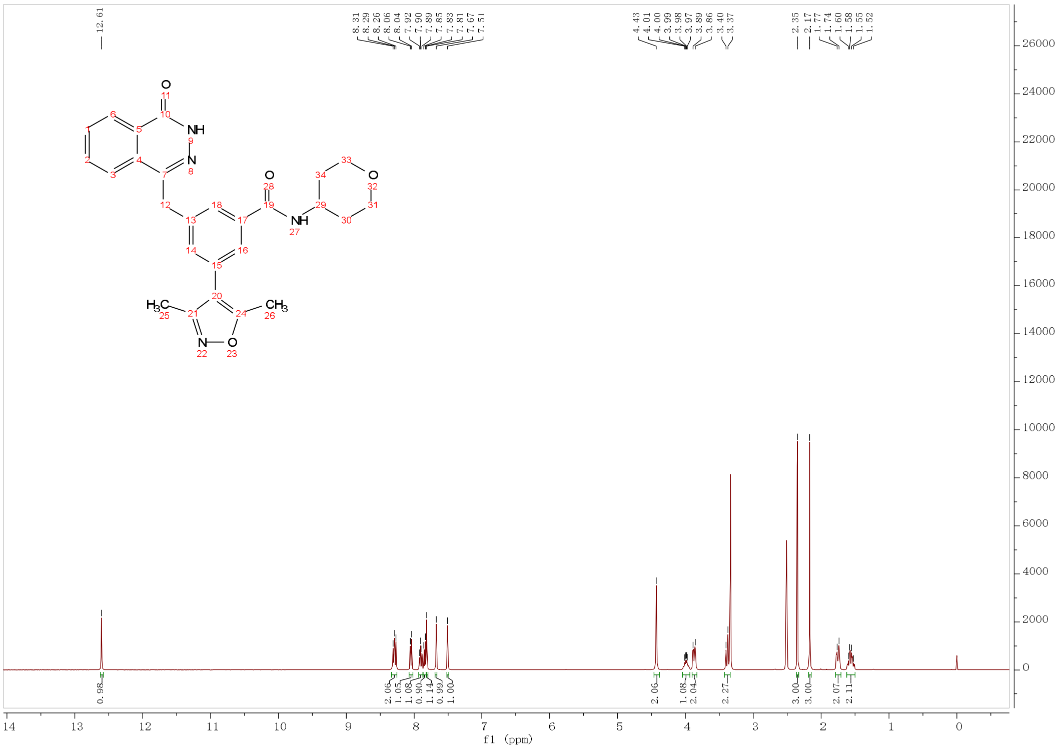

^{1}H-NMR spectrum of compound DDT08

pbg-43 in DMSO, 13C-NMR, 100MHz

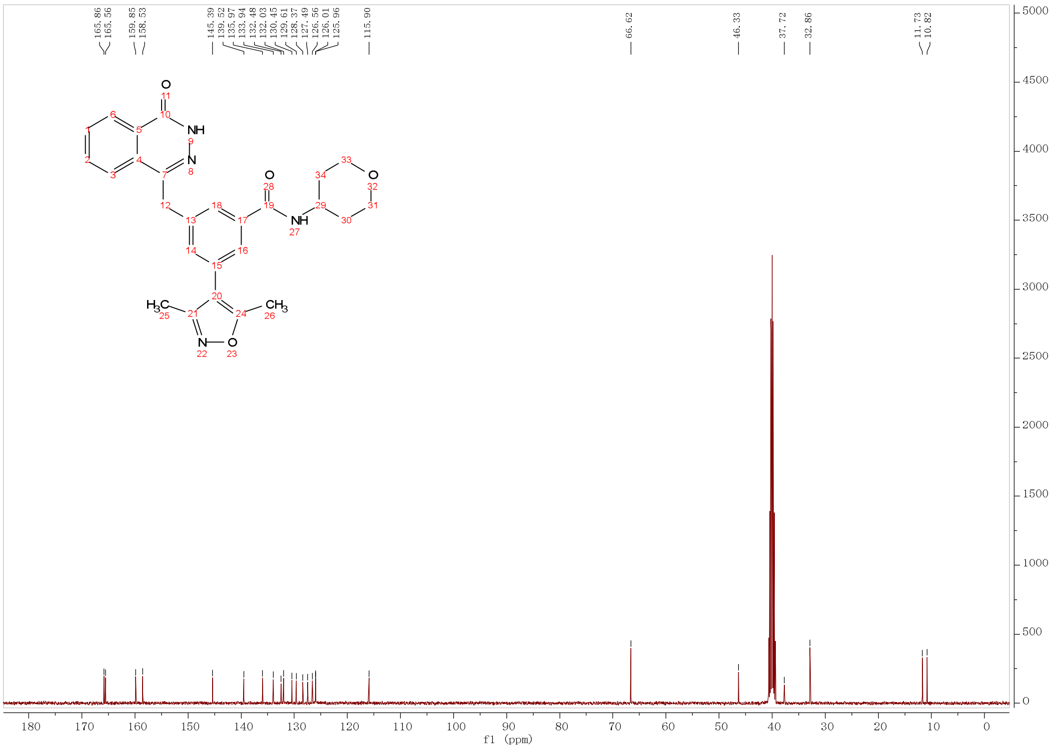

^{13}C-NMR spectrum of compound DDT08

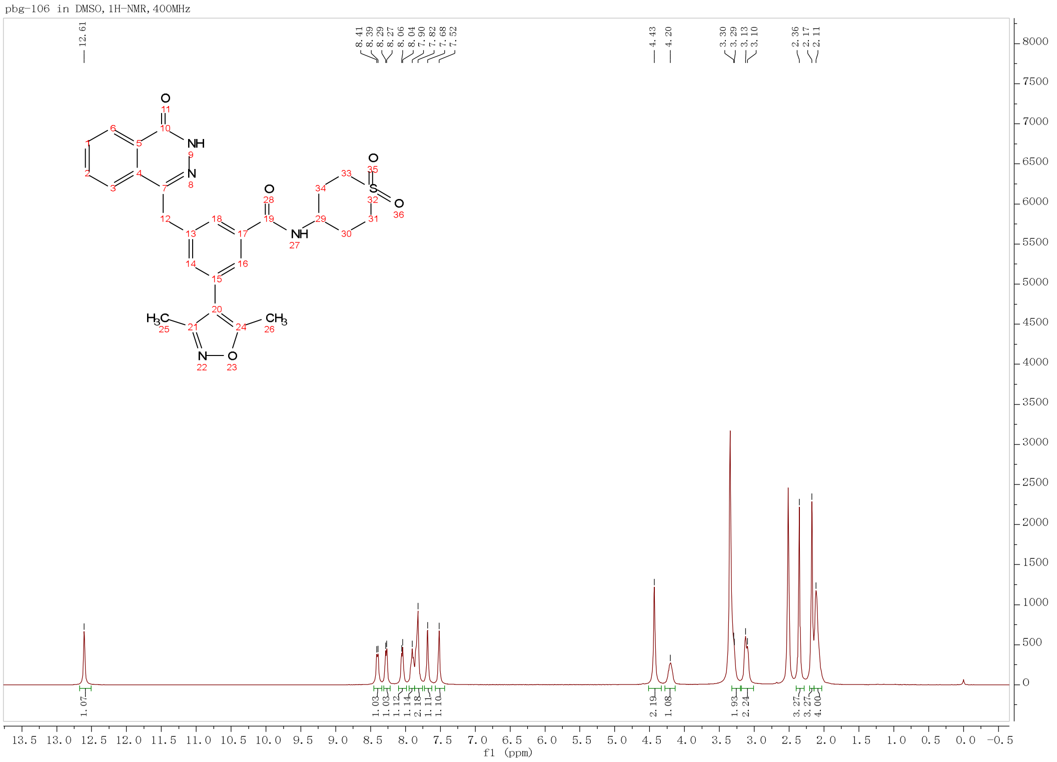

^{1}H-NMR spectrum of compound DDT09

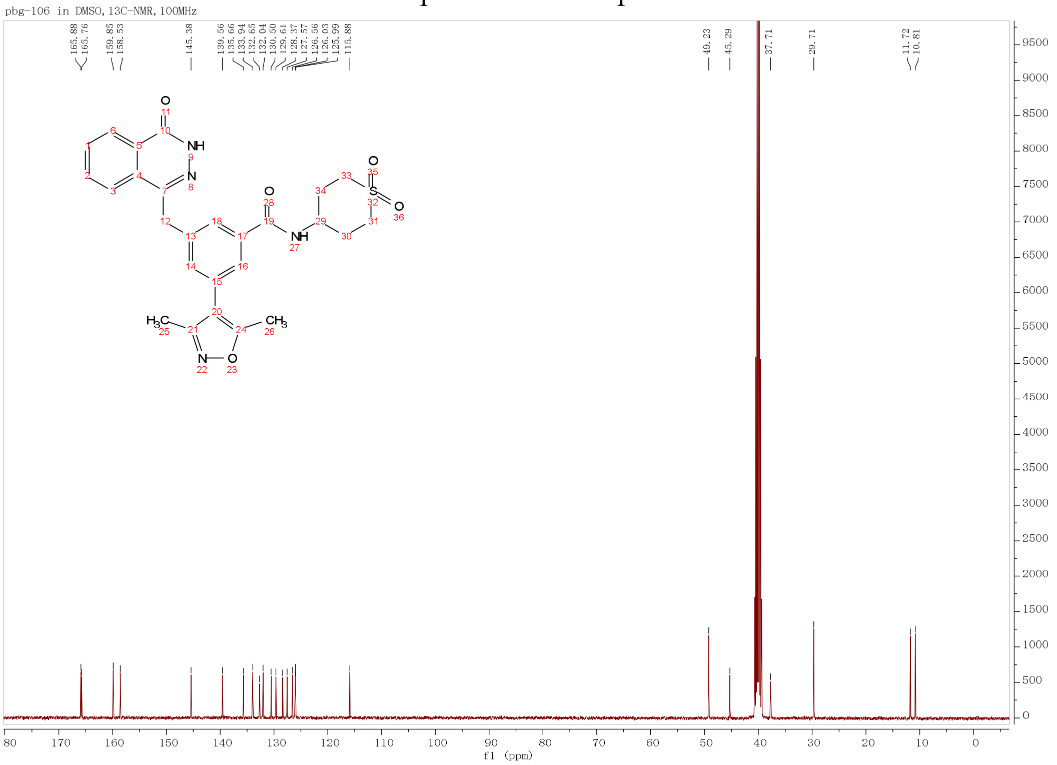

^{13}C-NMR spectrum of compound DDT09

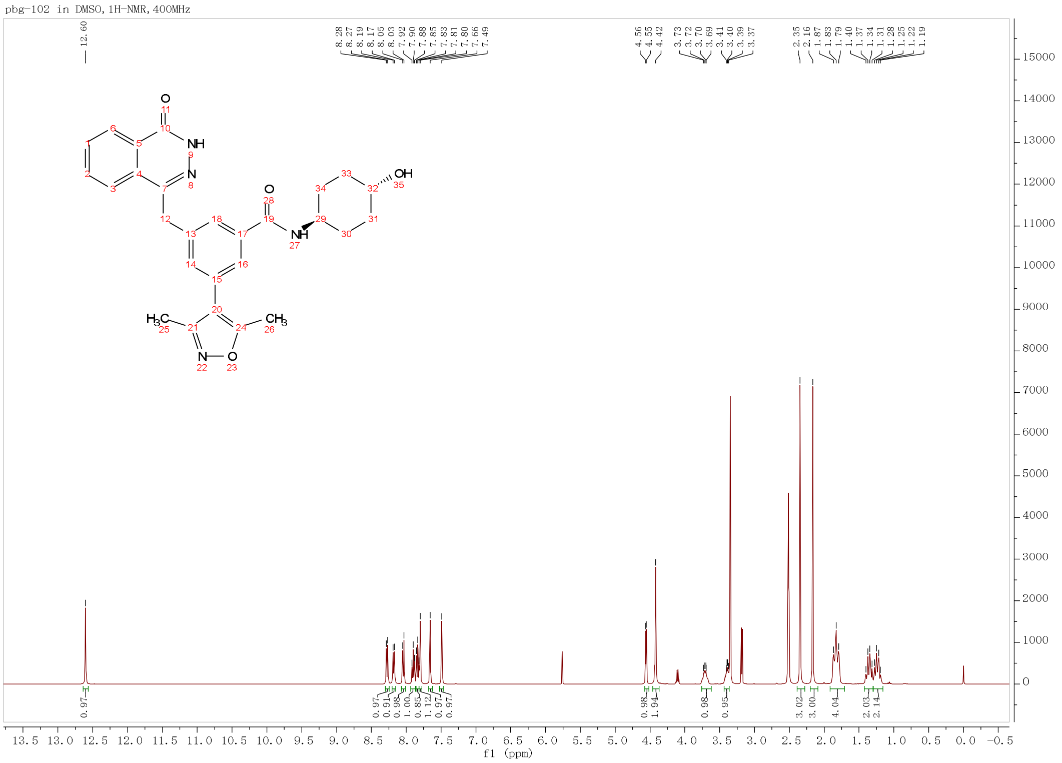

^{1}H-NMR spectrum of compound DDT10

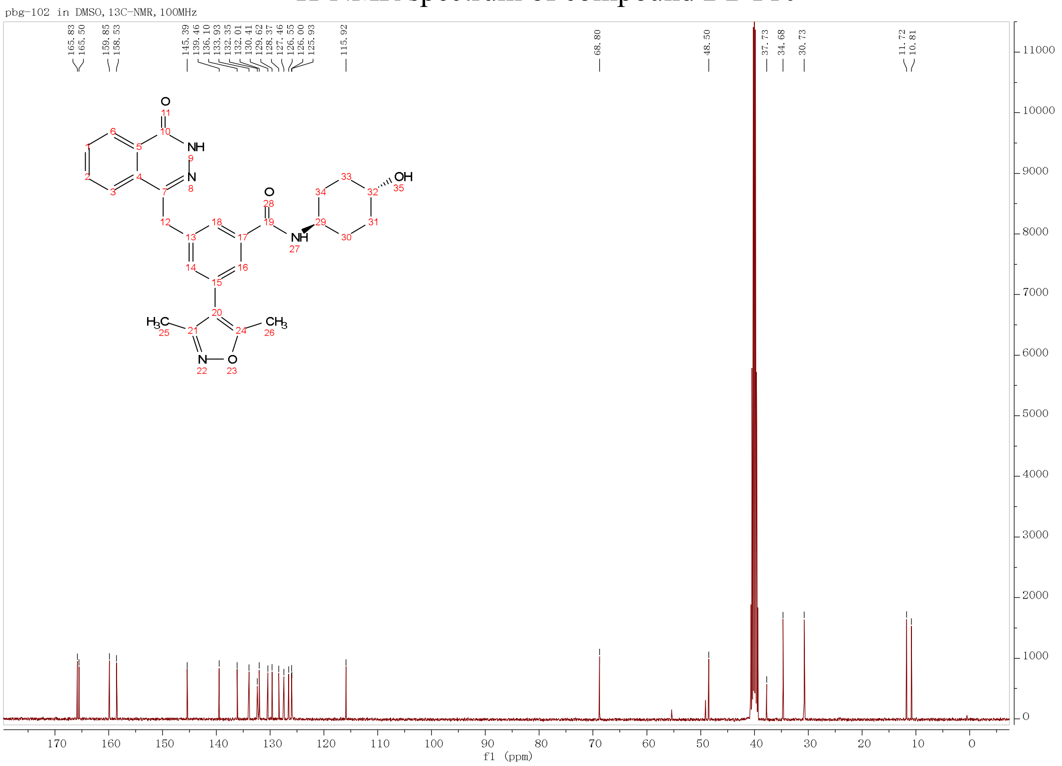

^{13}C-NMR spectrum of compound DDT10

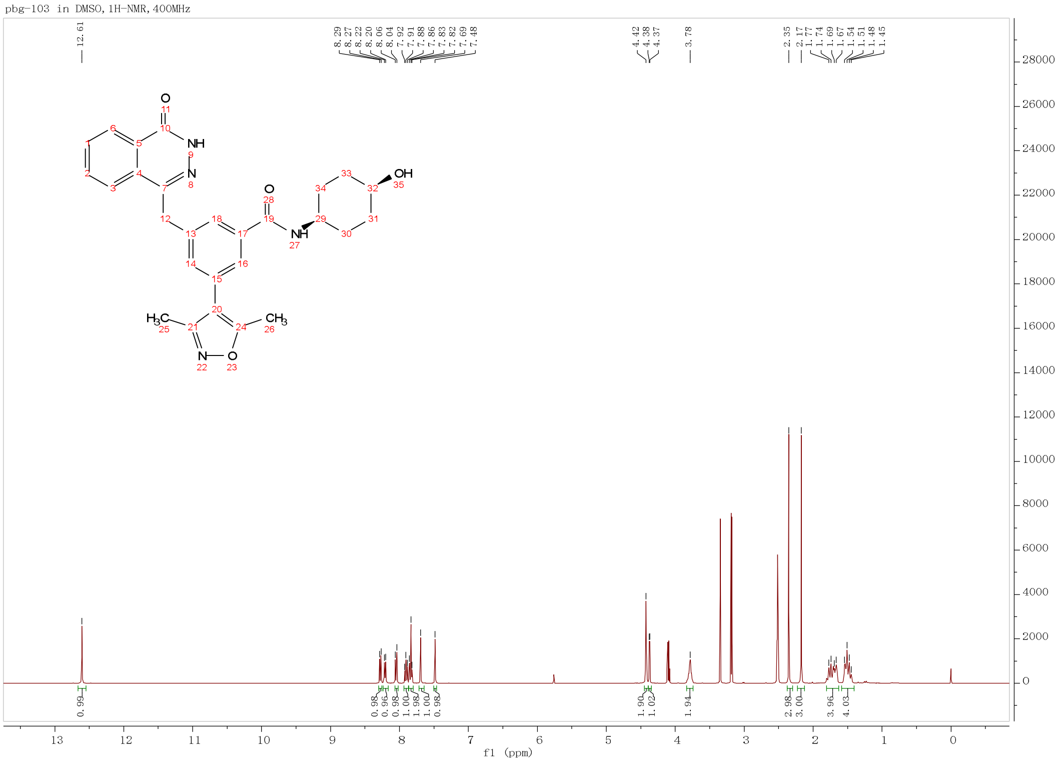

^{1}H-NMR spectrum of compound DDT11

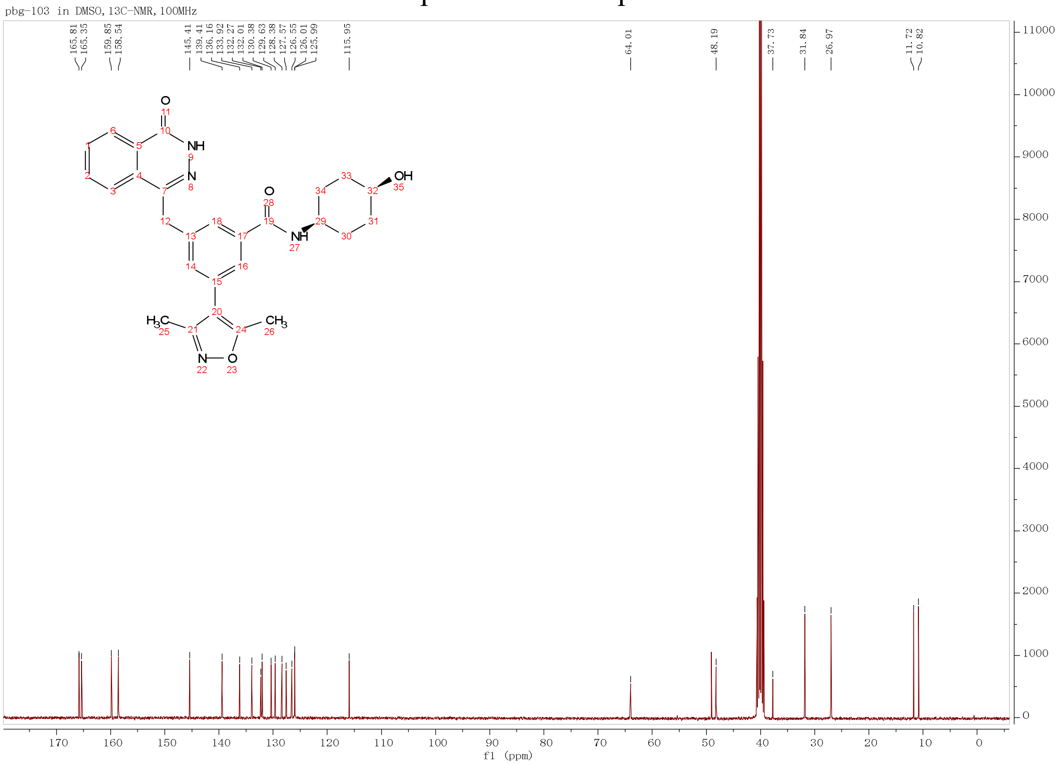

^{13}C-NMR spectrum of compound DDT11

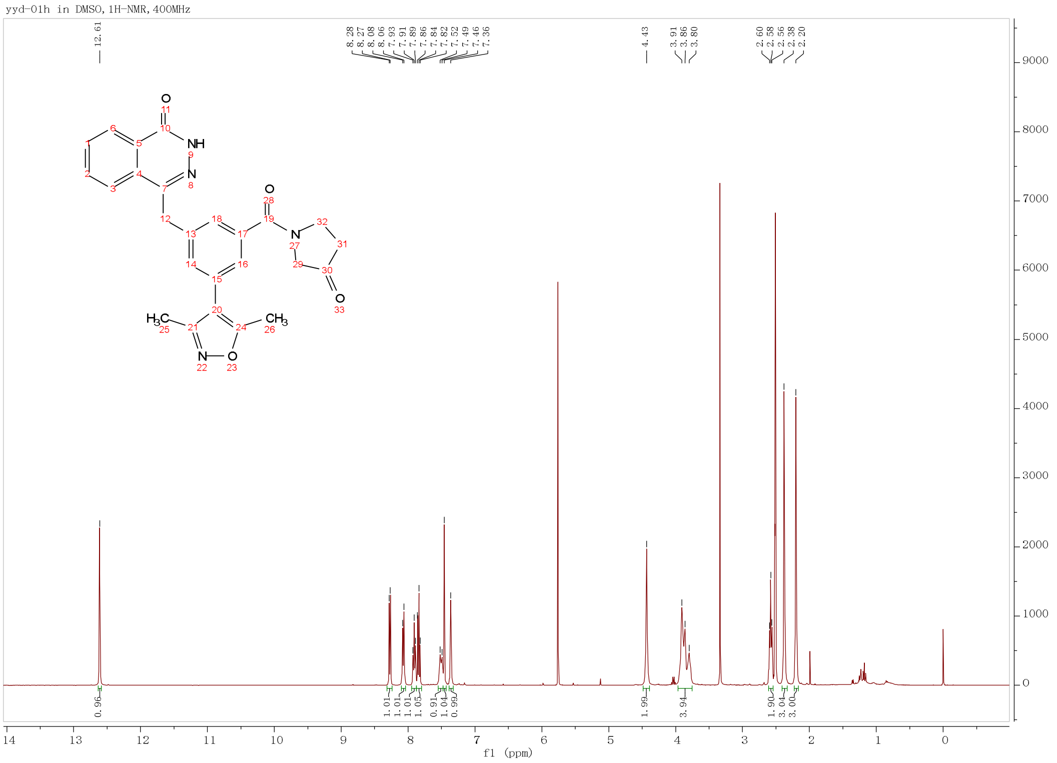

^{1}H-NMR spectrum of compound DDT12

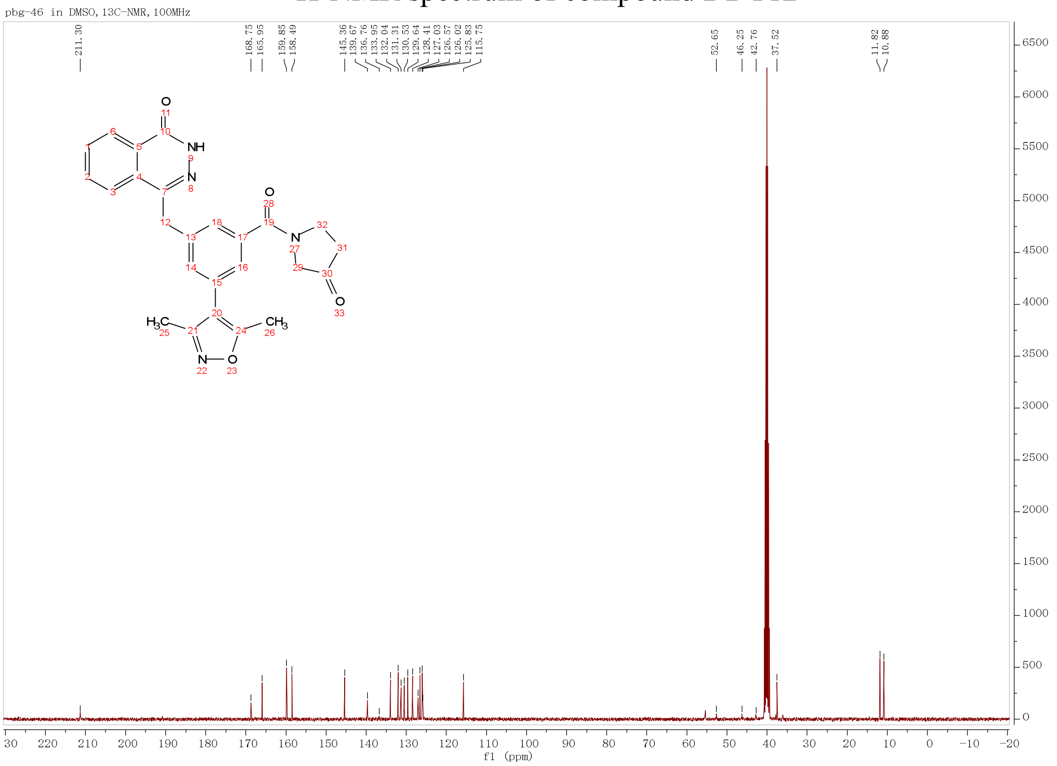

^{13}C-NMR spectrum of compound DDT12

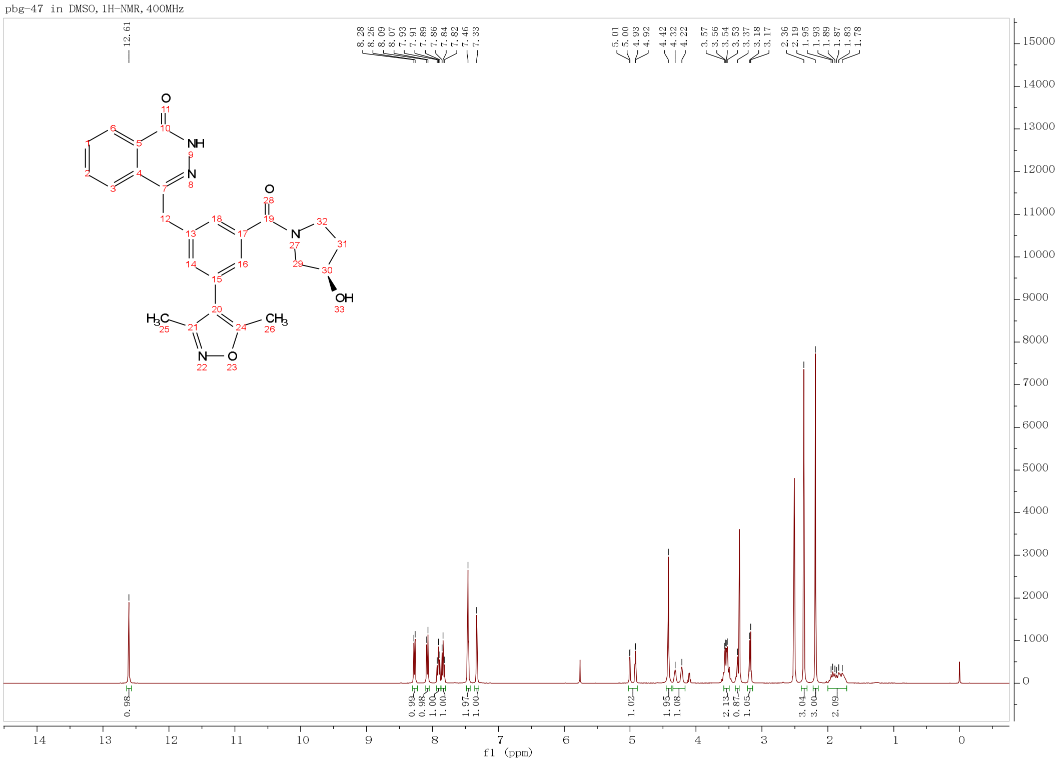

^{1}H-NMR spectrum of compound DDT13

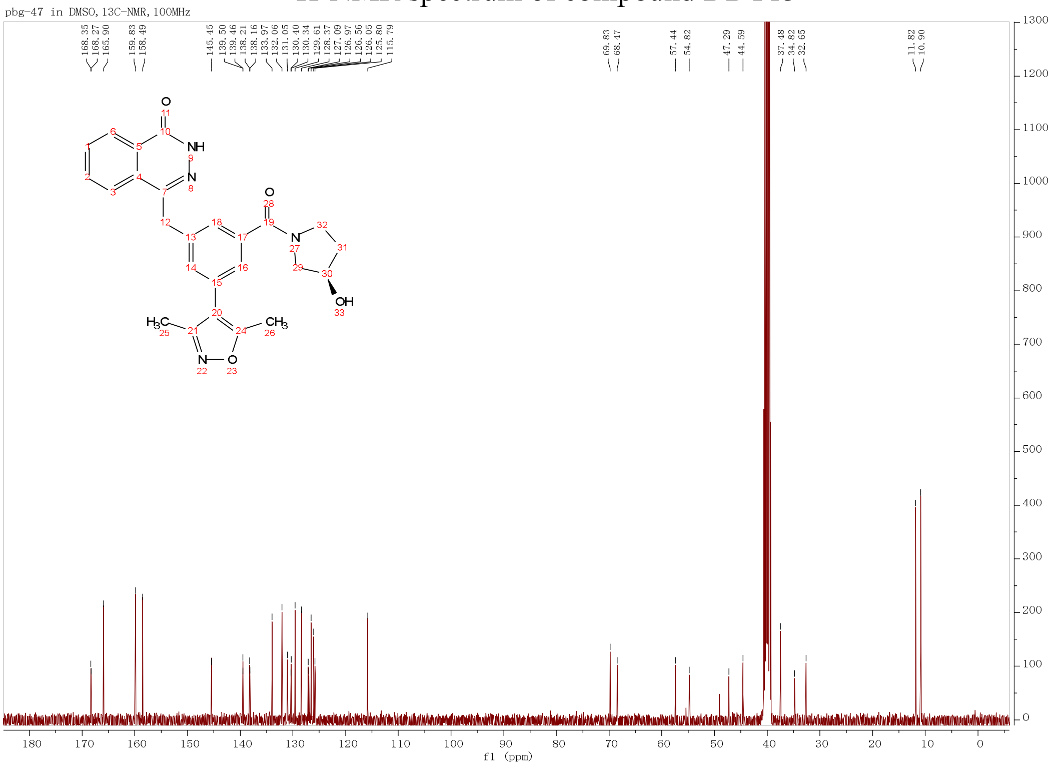

^{13}C-NMR spectrum of compound DDT13

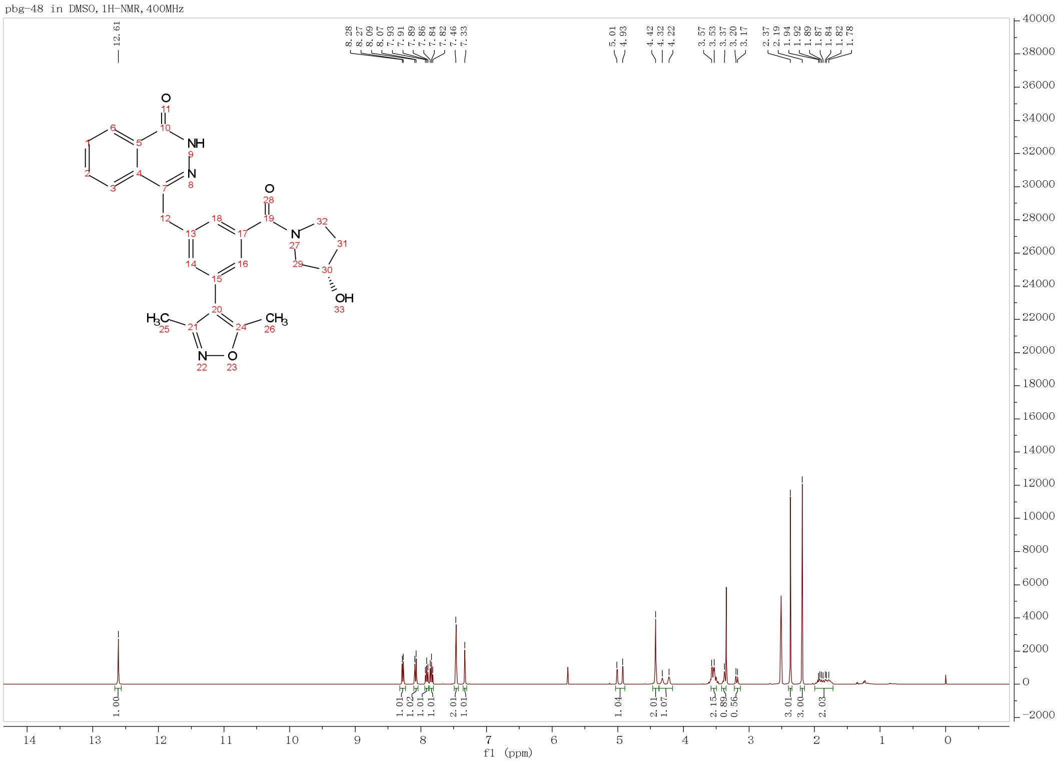

^{1}H-NMR spectrum of compound DDT14

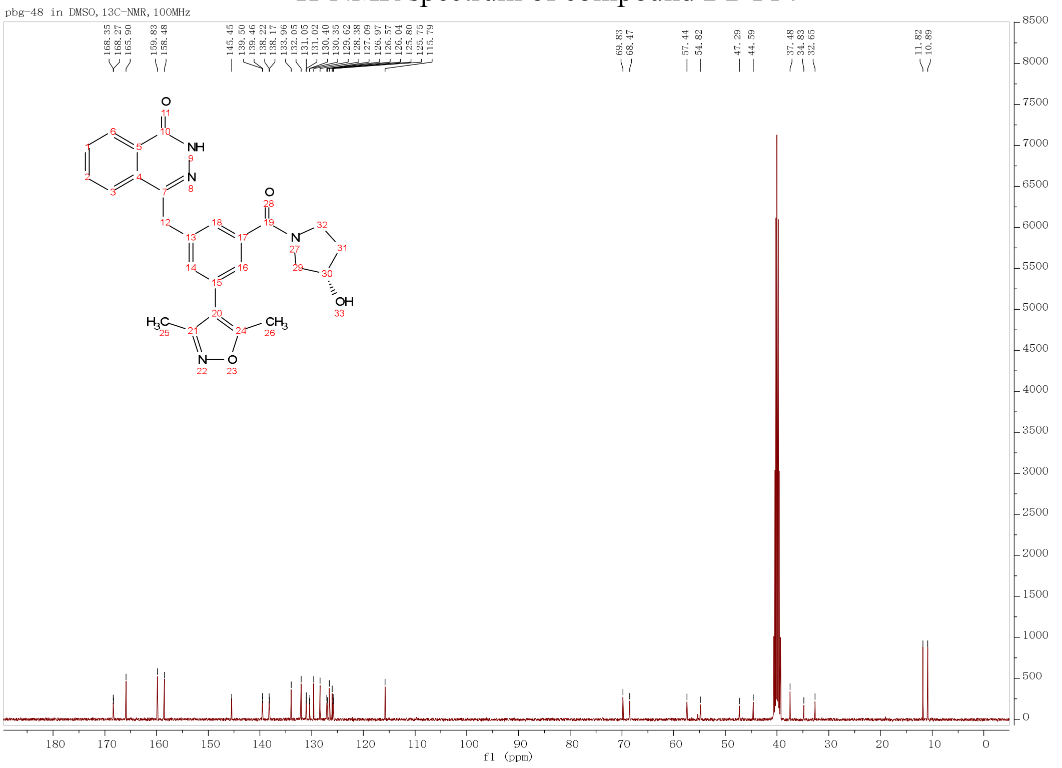

^{13}C-NMR spectrum of compound DDT14

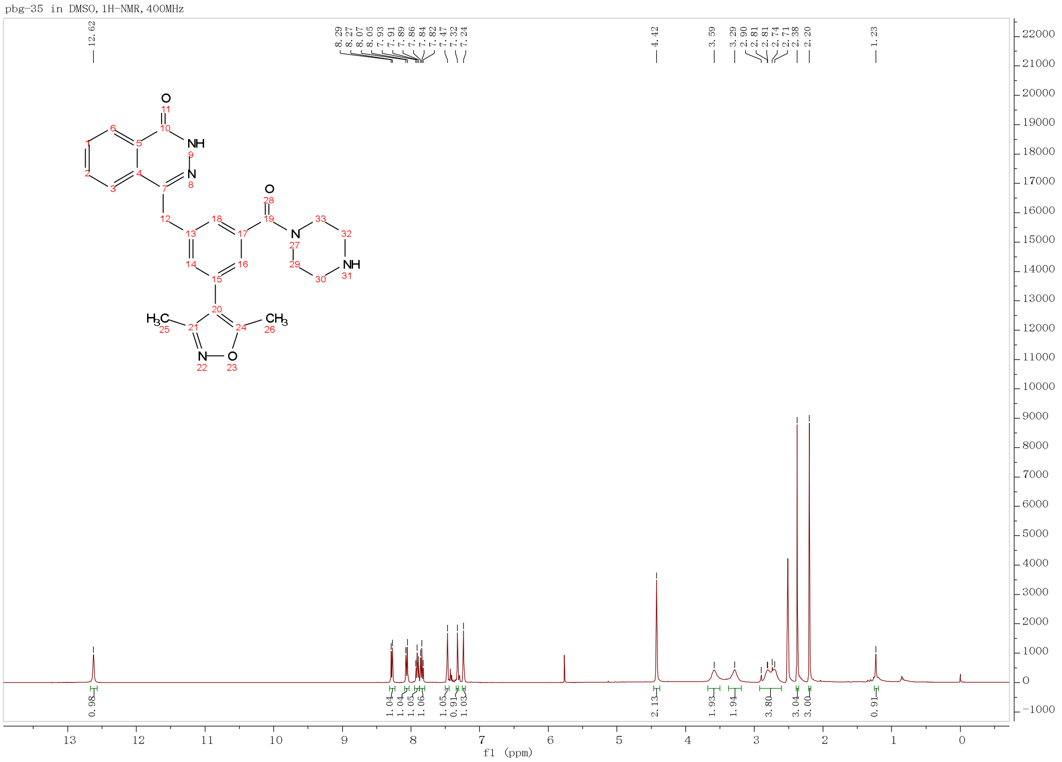

^{1}H-NMR spectrum of compound DDT15

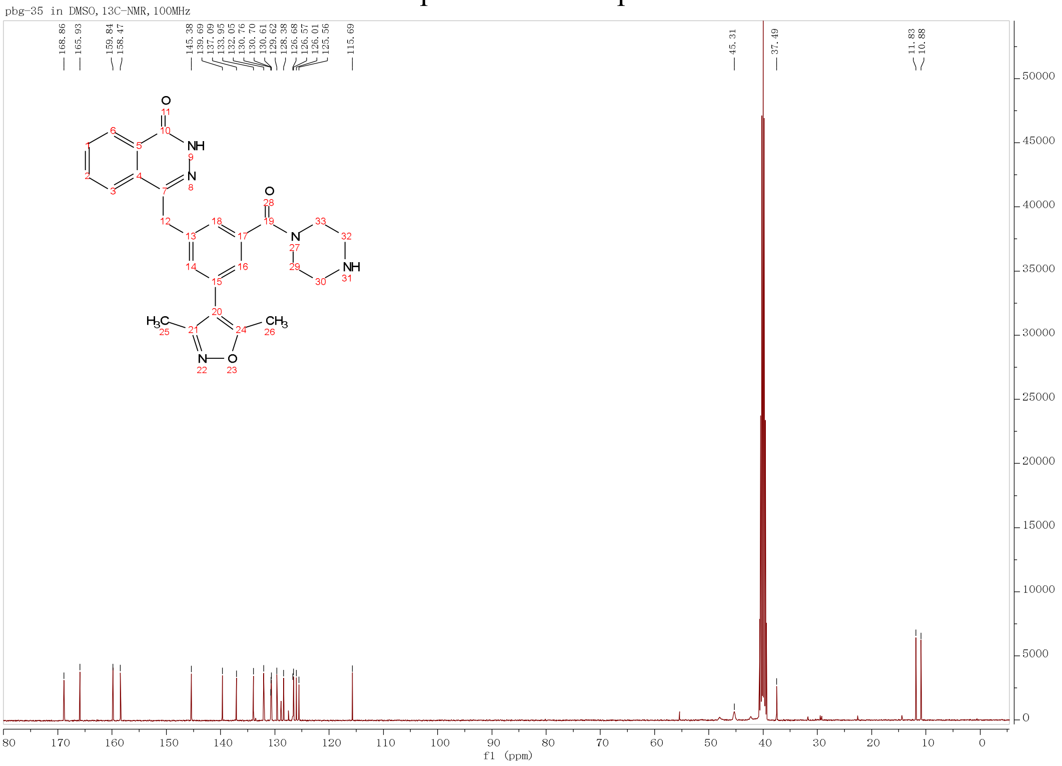

^{13}C-NMR spectrum of compound DDT15

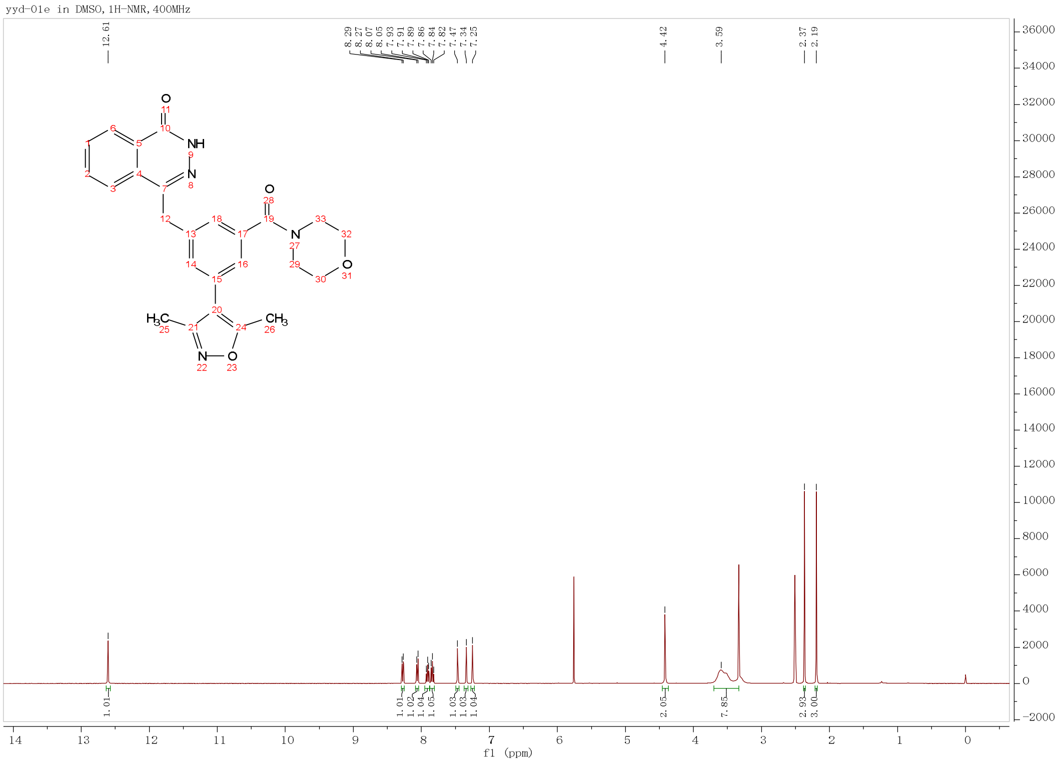

^{1}H-NMR spectrum of compound DDT16

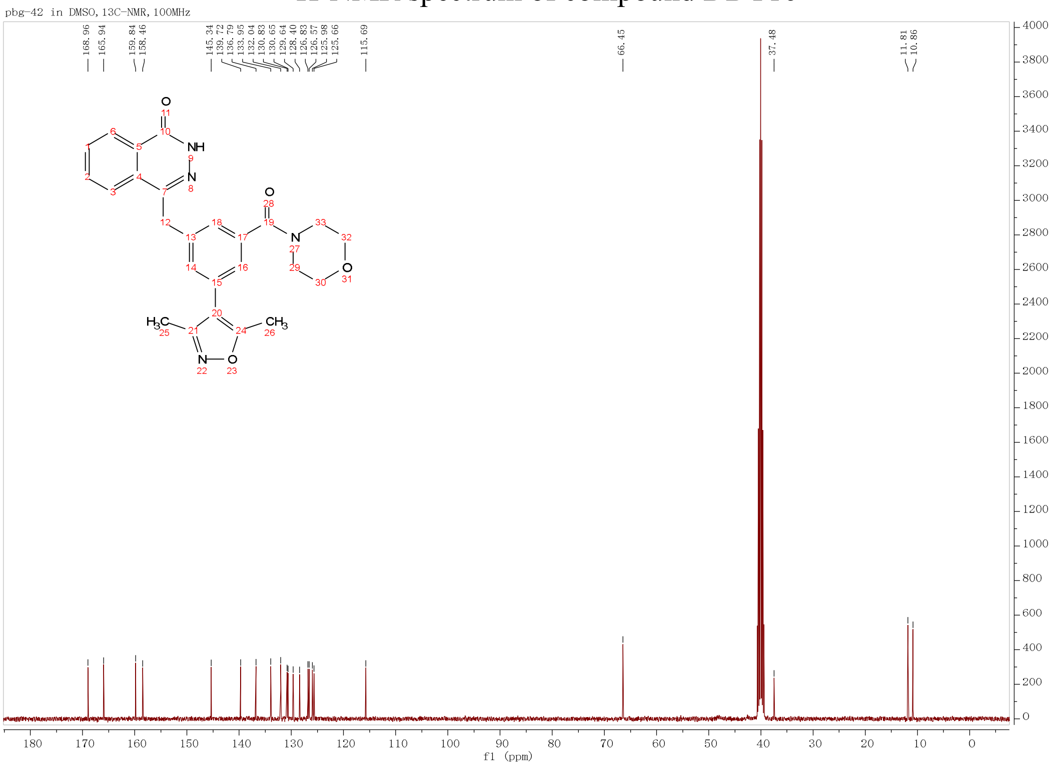

^{13}C-NMR spectrum of compound DDT16

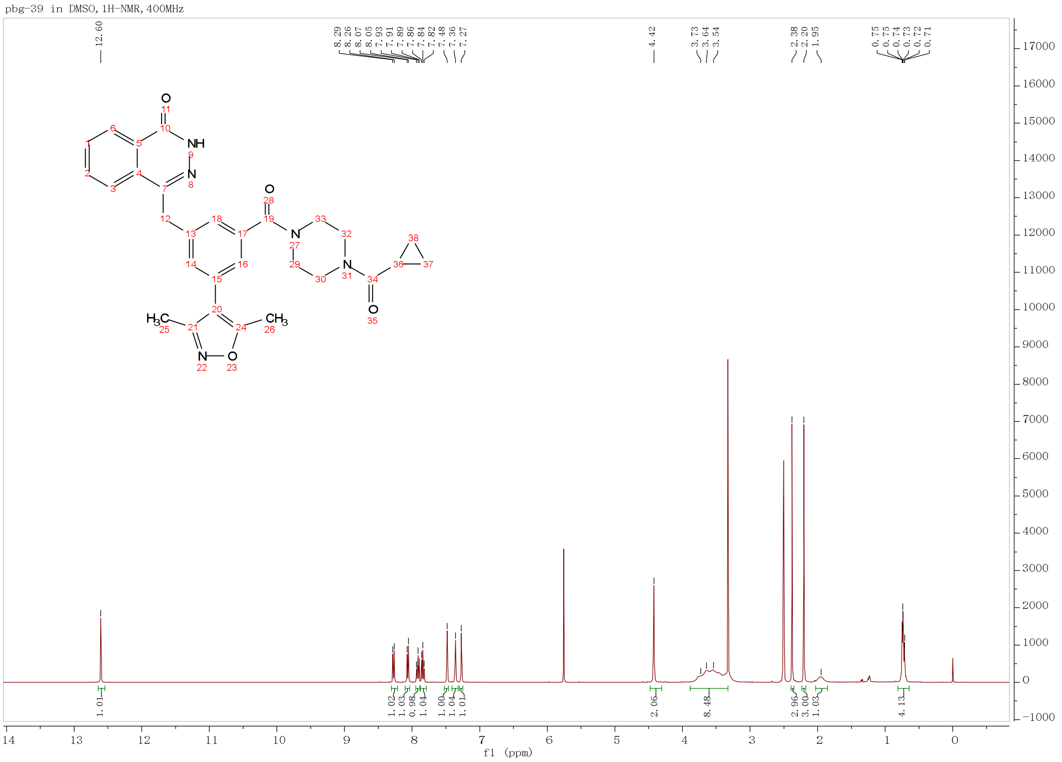

^{1}H-NMR spectrum of compound DDT17

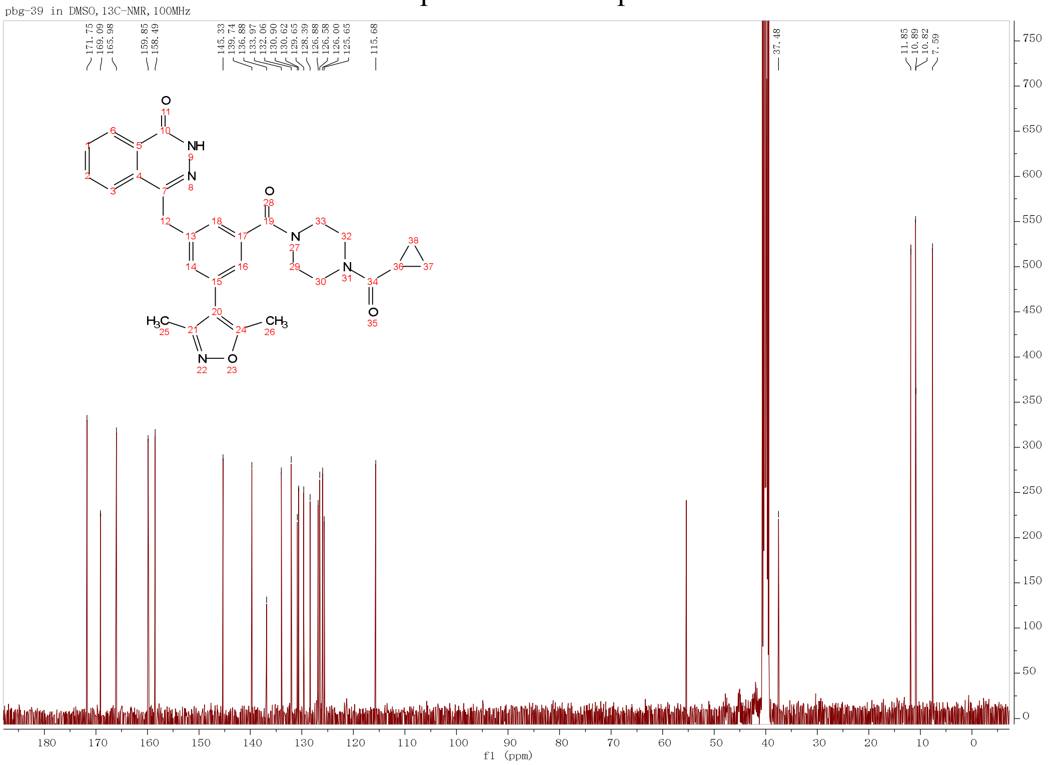

^{13}C-NMR spectrum of compound DDT17

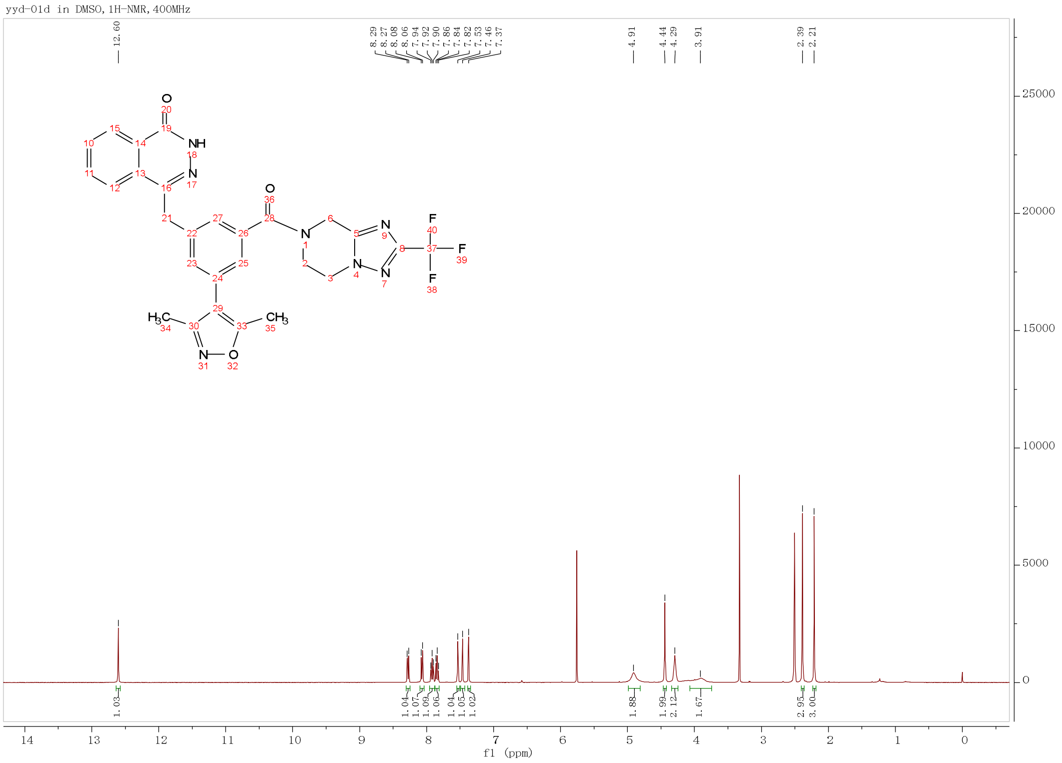

^{1}H-NMR spectrum of compound DDT18

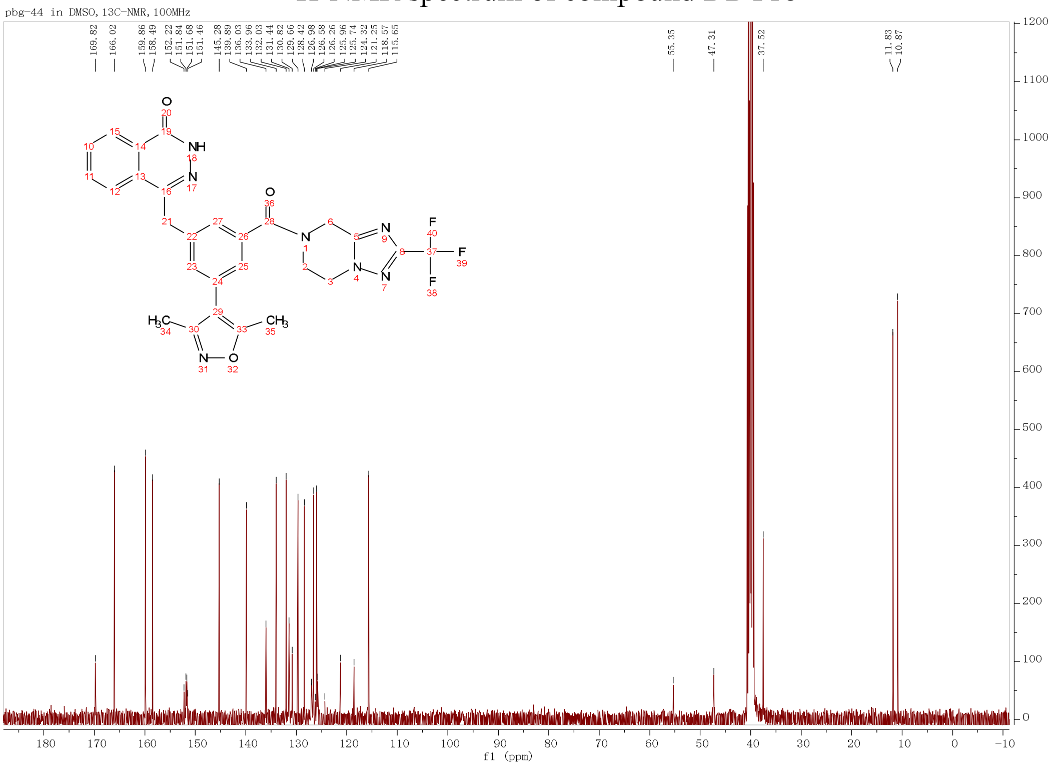

^{13}C-NMR spectrum of compound DDT18

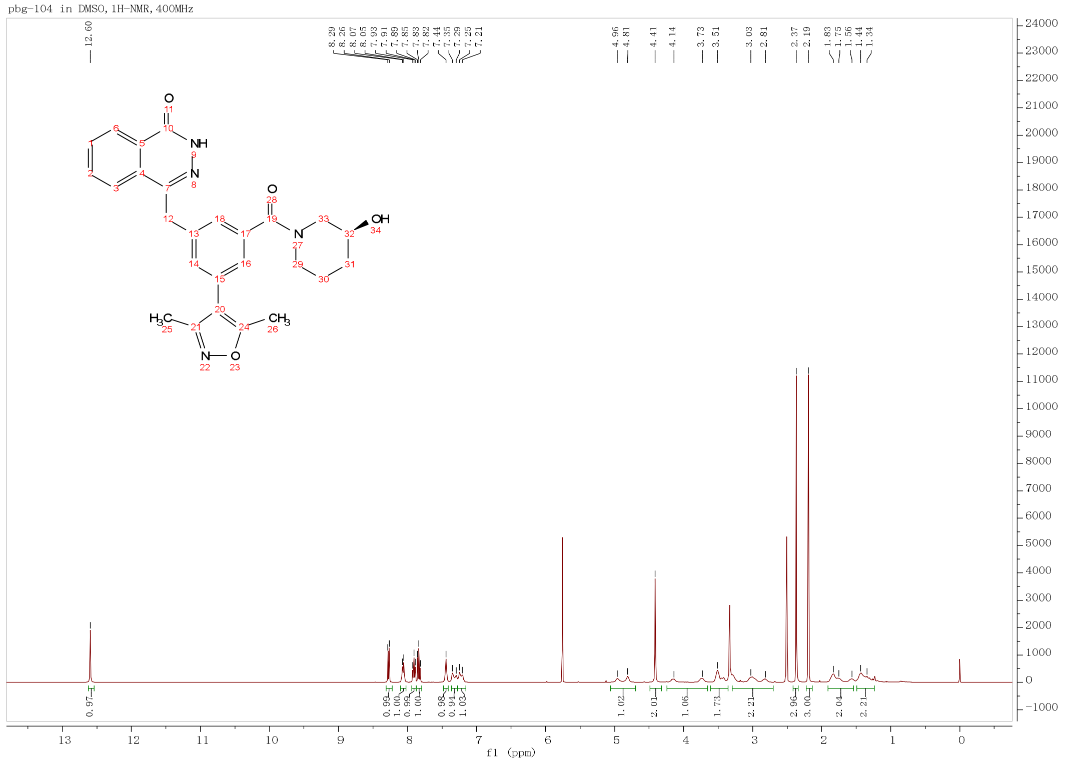

^{1}H-NMR spectrum of compound DDT19

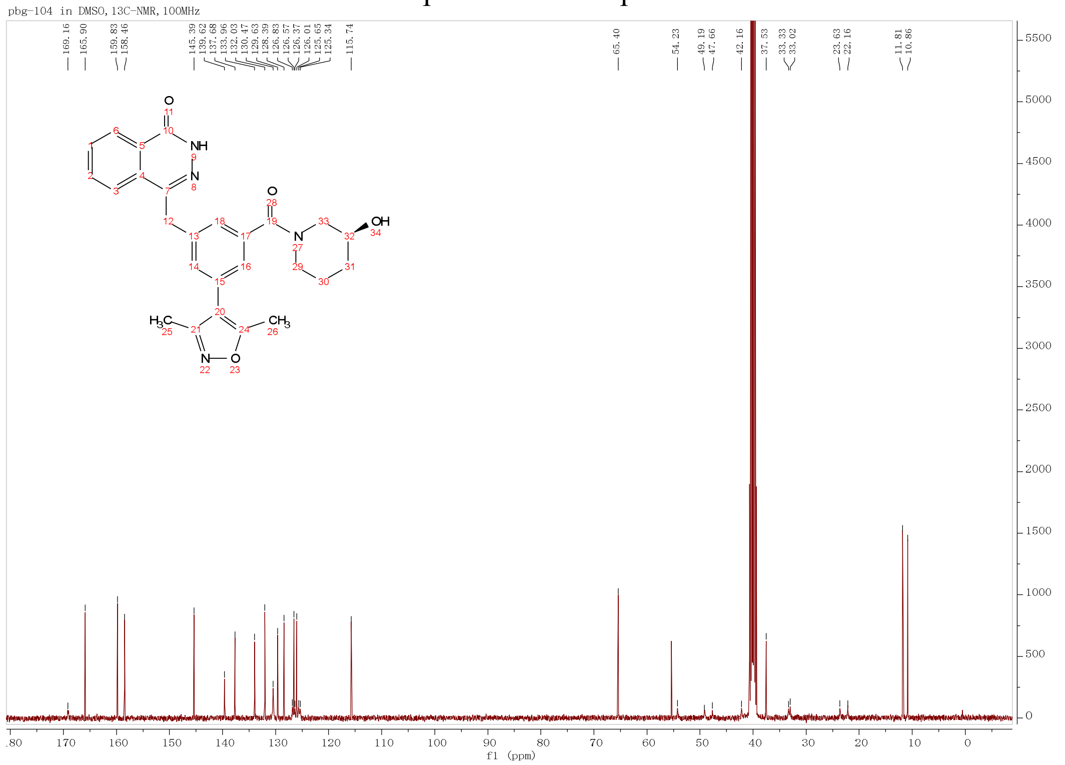

^{13}C-NMR spectrum of compound DDT19

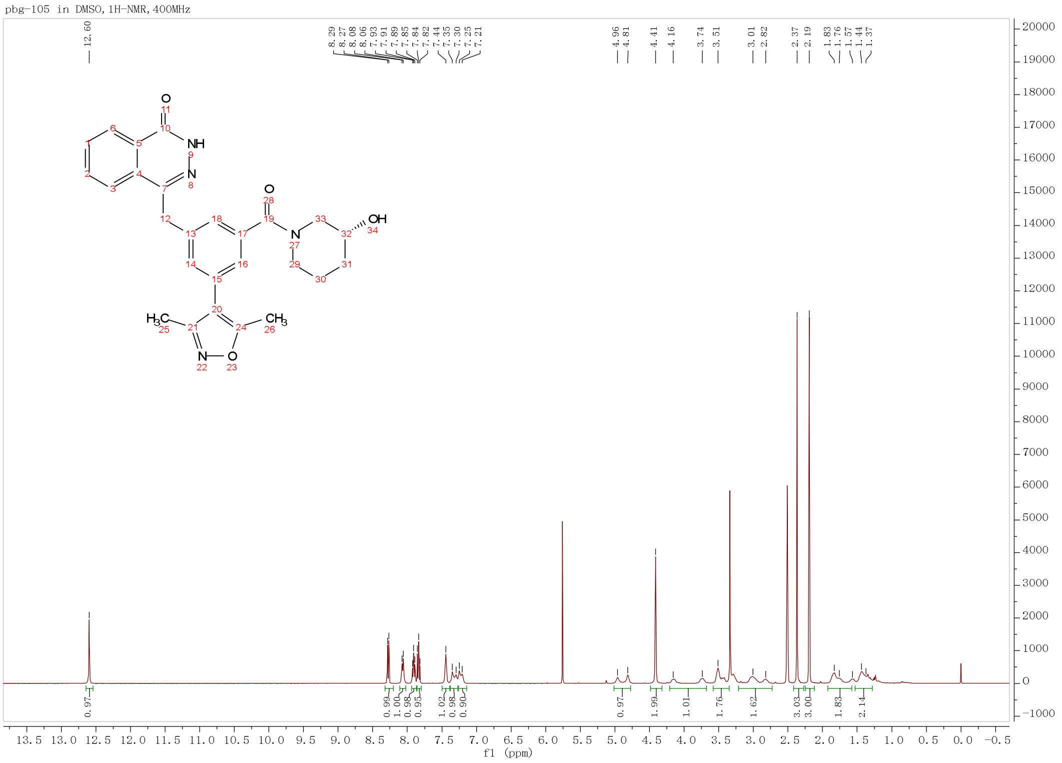

^{1}H-NMR spectrum of compound DDT20

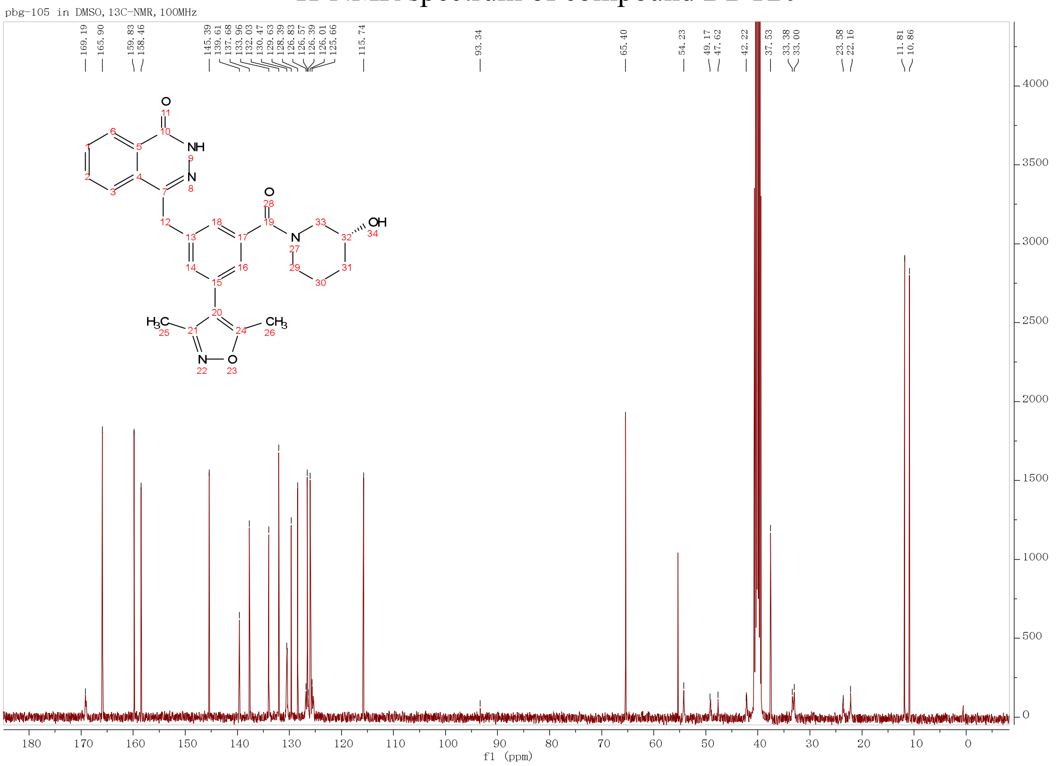

^{13}C-NMR spectrum of compound DDT20

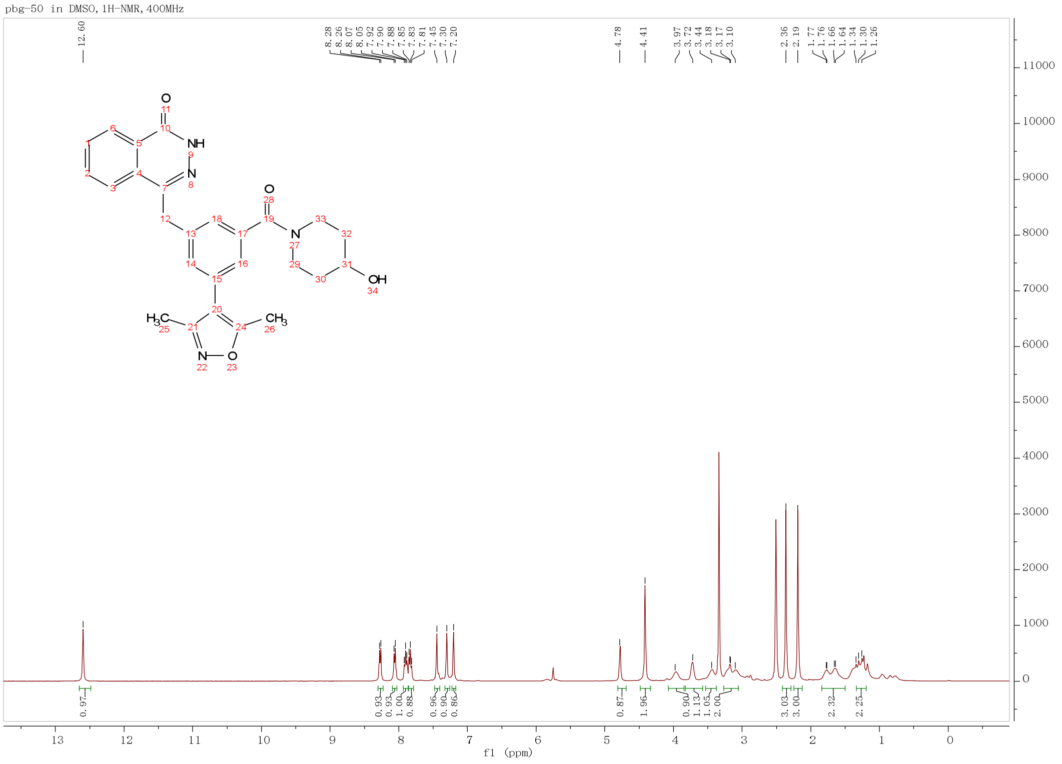

^{1}H-NMR spectrum of compound DDT21

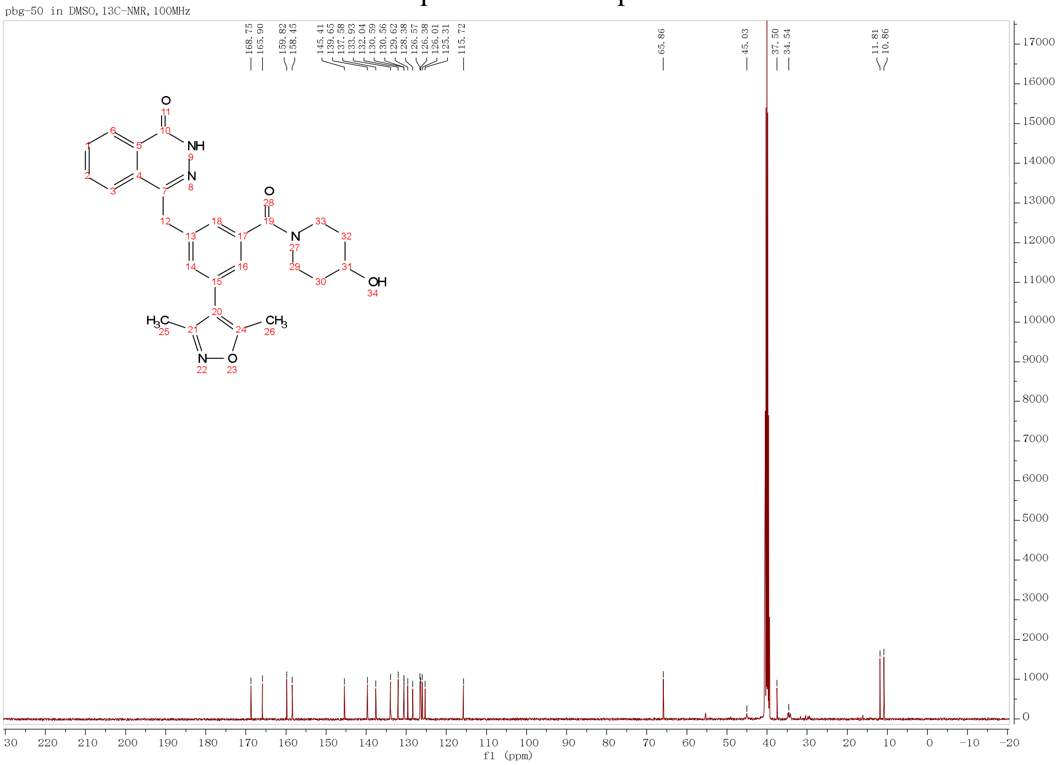

^{13}C-NMR spectrum of compound DDT21

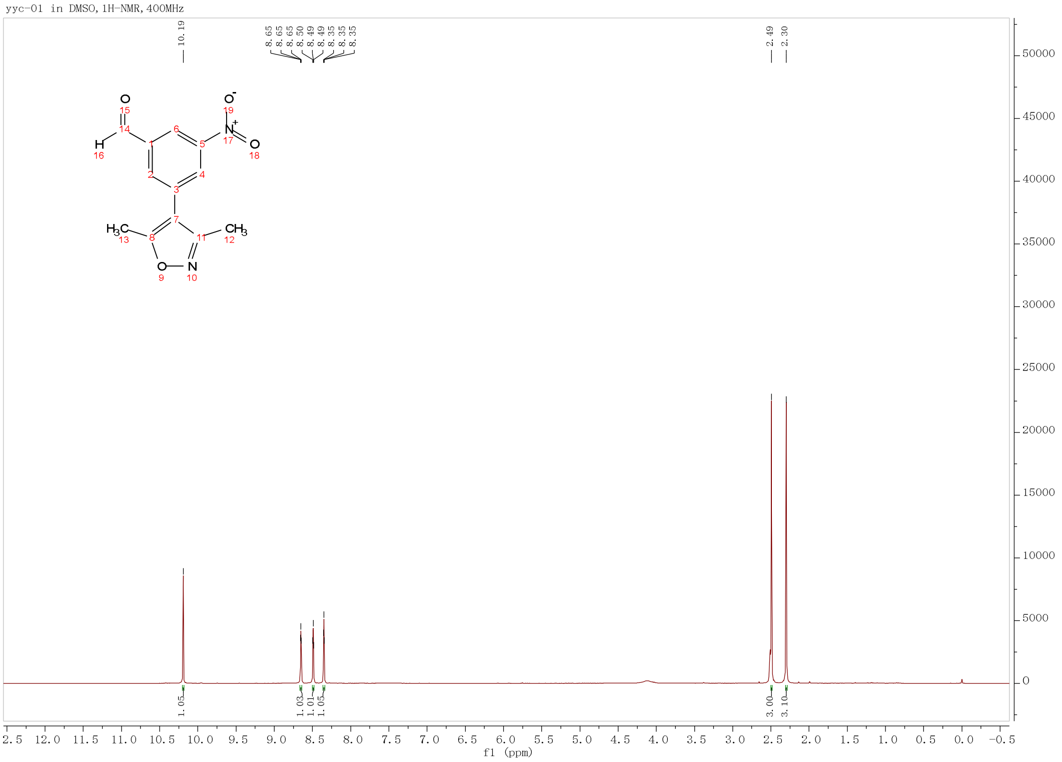

^{1}H-NMR spectrum of compound 16

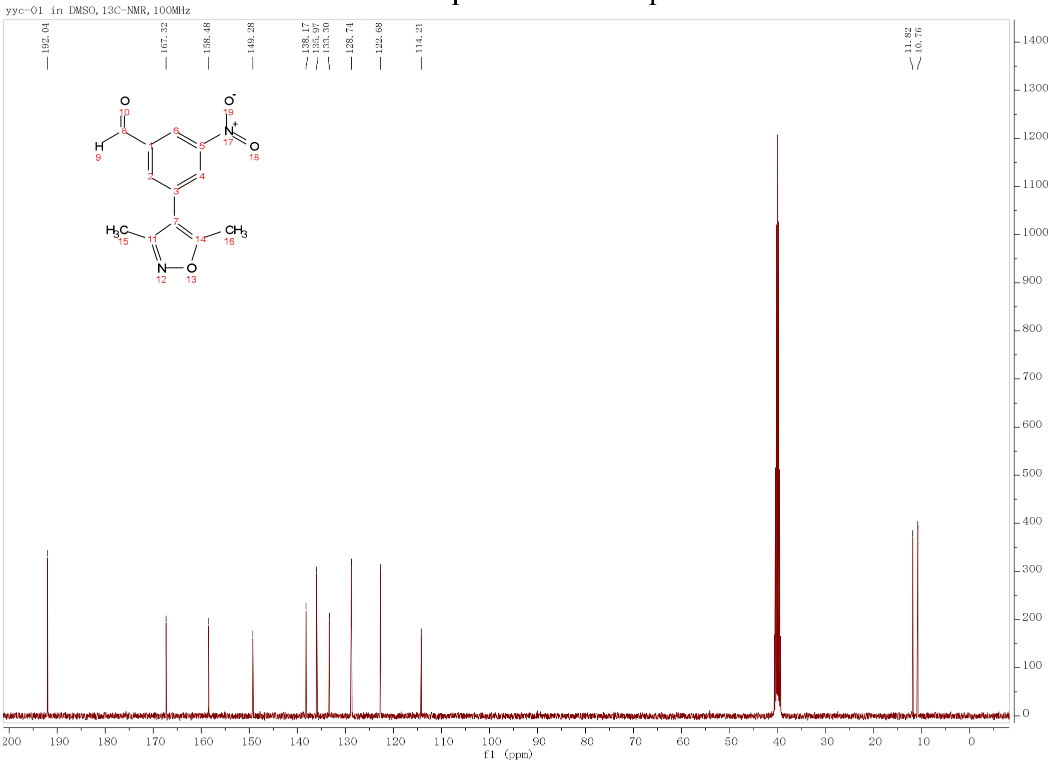

^{13}C-NMR spectrum of compound 16

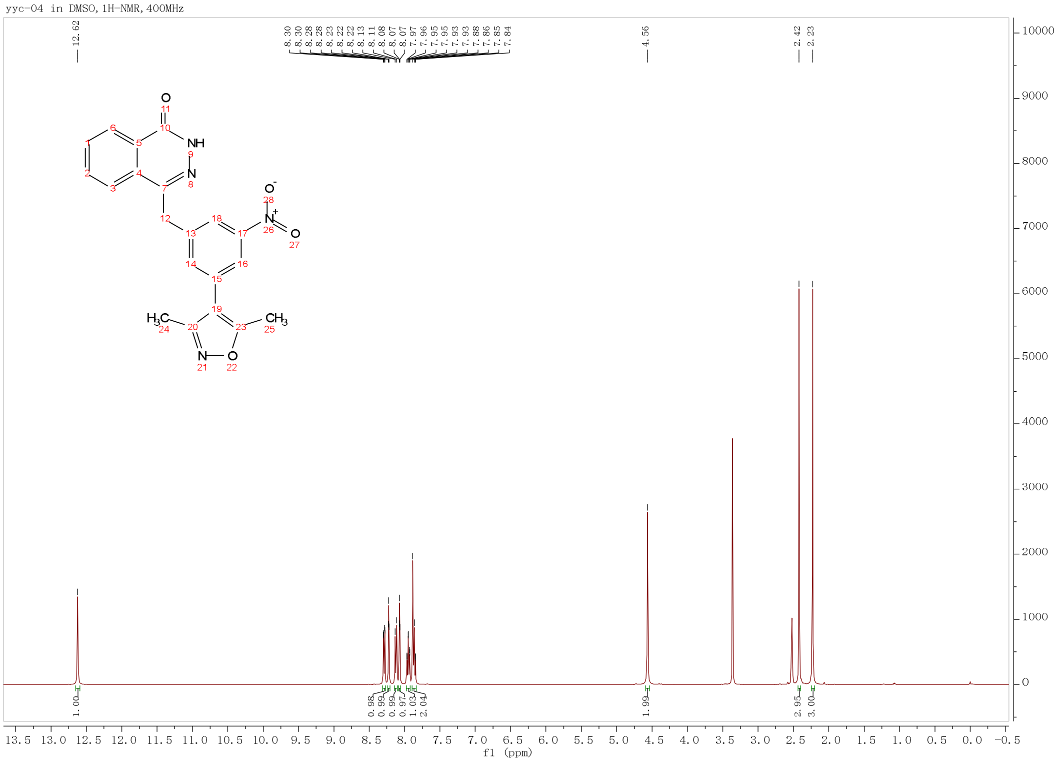

^{1}H-NMR spectrum of compound 18

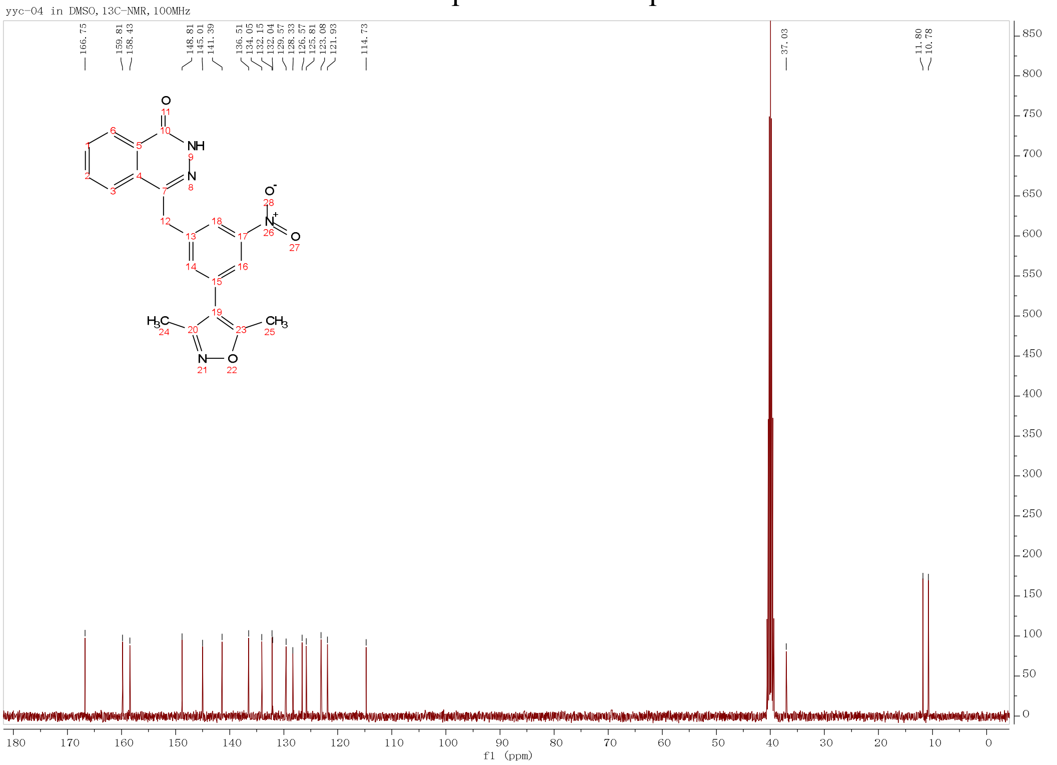

^{13}C-NMR spectrum of compound 18

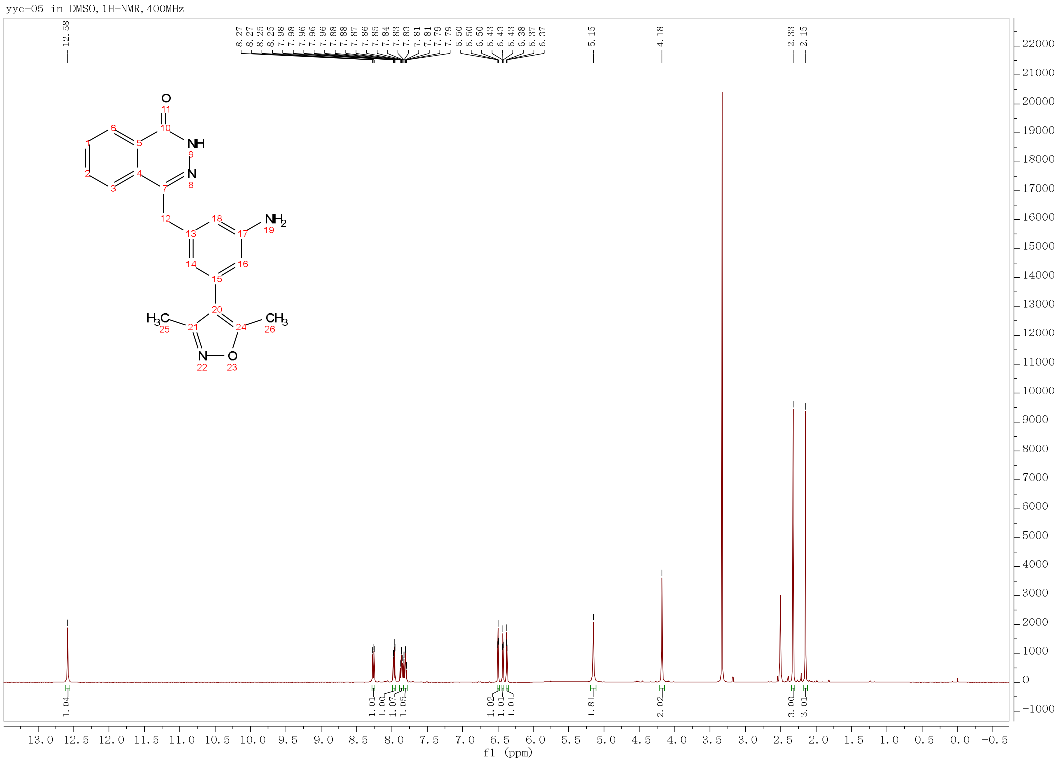

^{1}H-NMR spectrum of compound 19

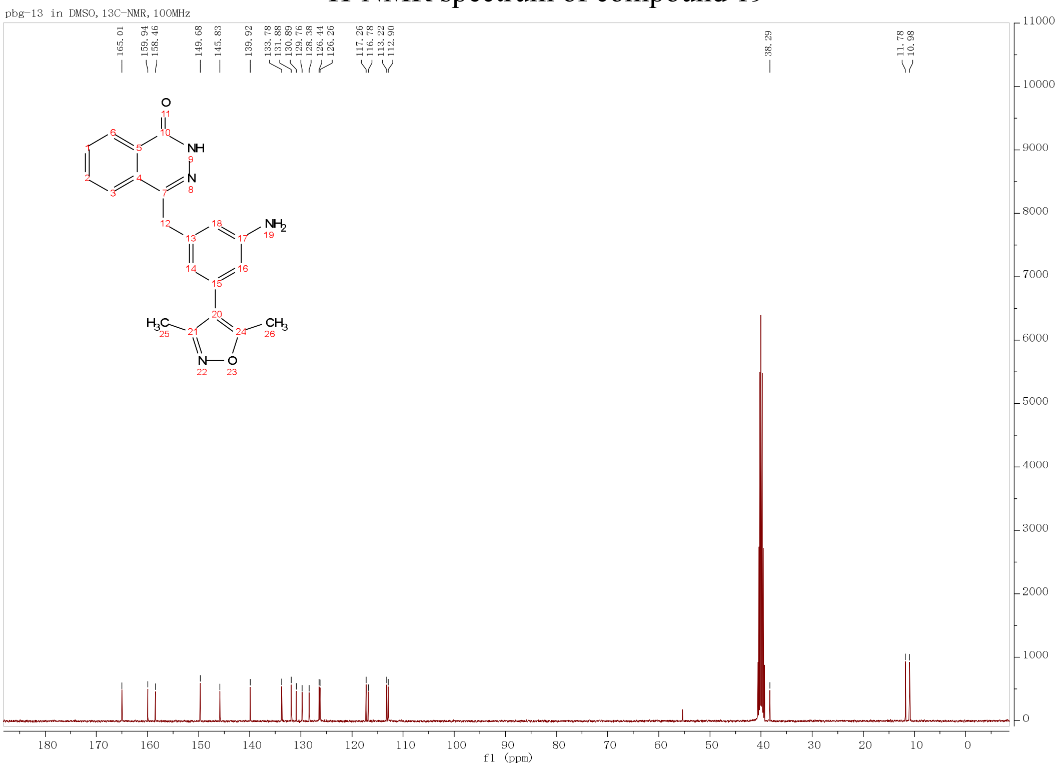

^{13}C-NMR spectrum of compound 19

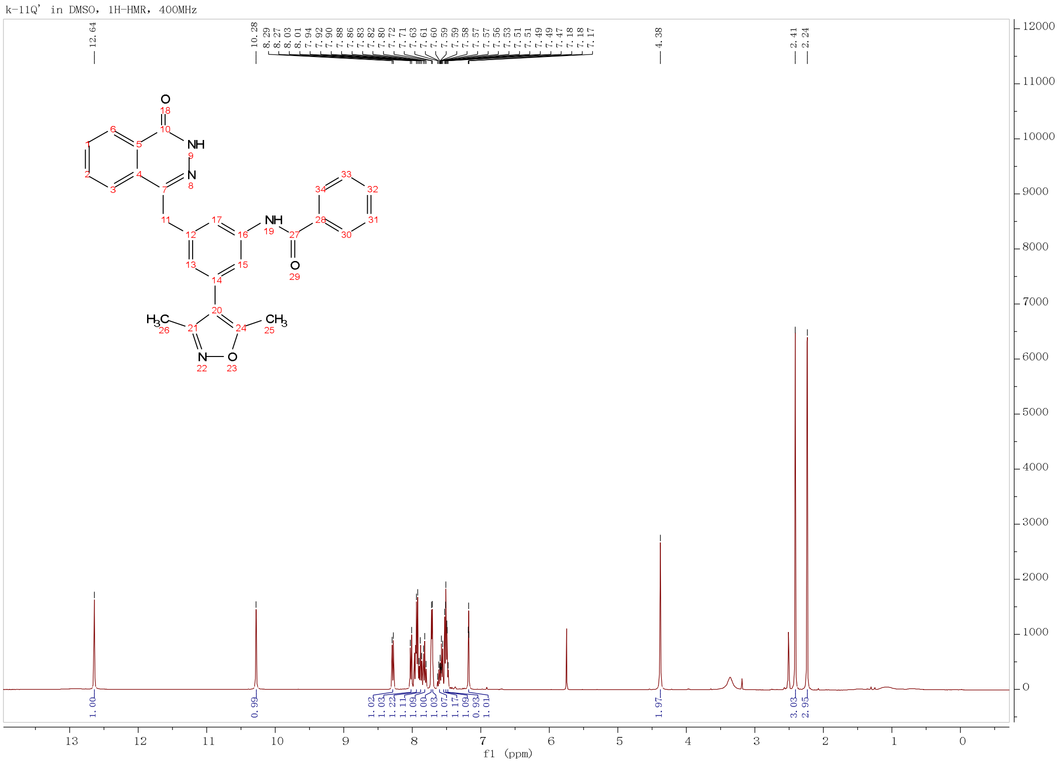

^{1}H-NMR spectrum of compound DDT22

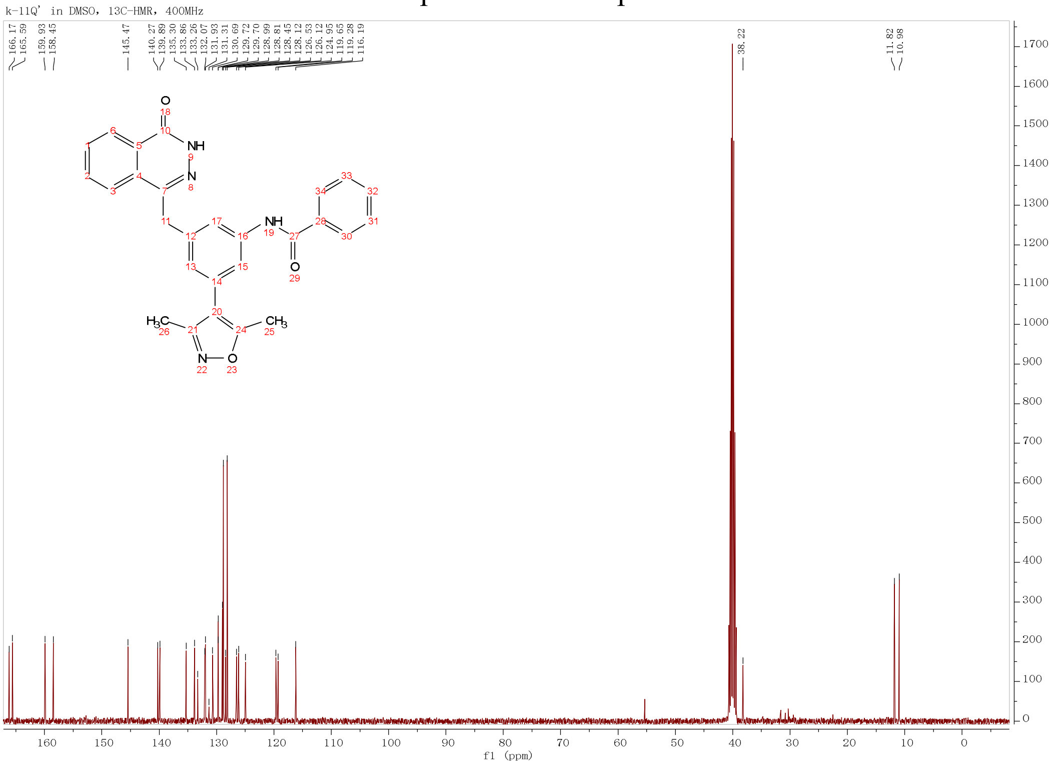

^{13}C-NMR spectrum of compound DDT22

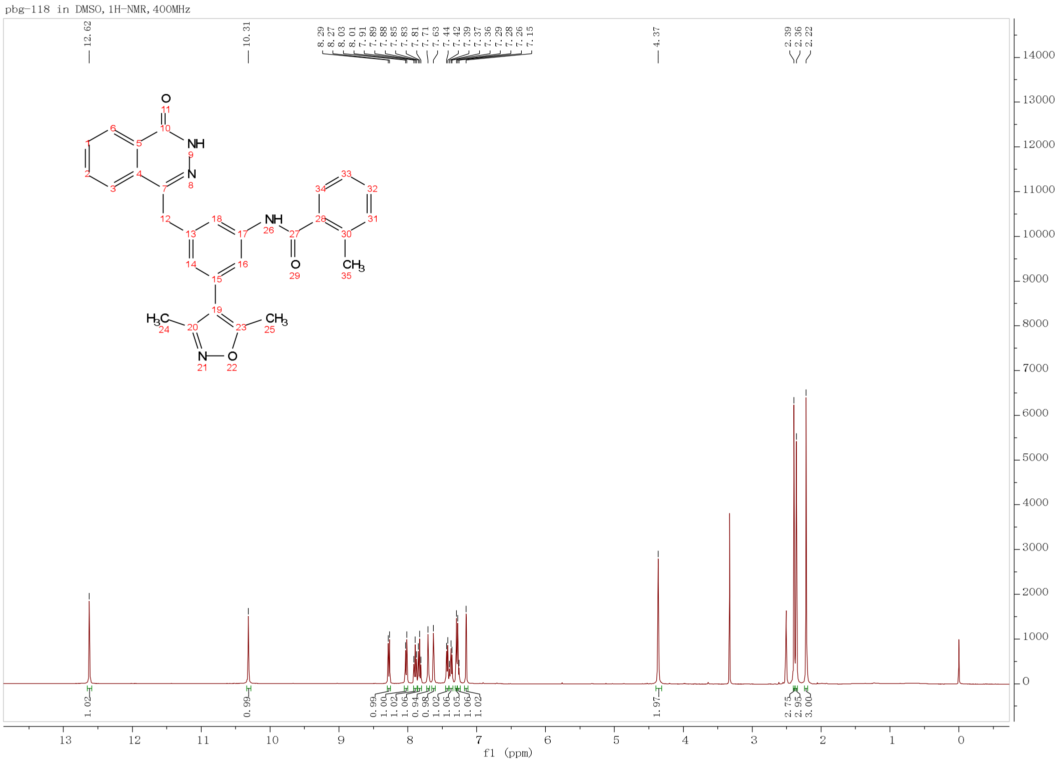

^{1}H-NMR spectrum of compound DDT23

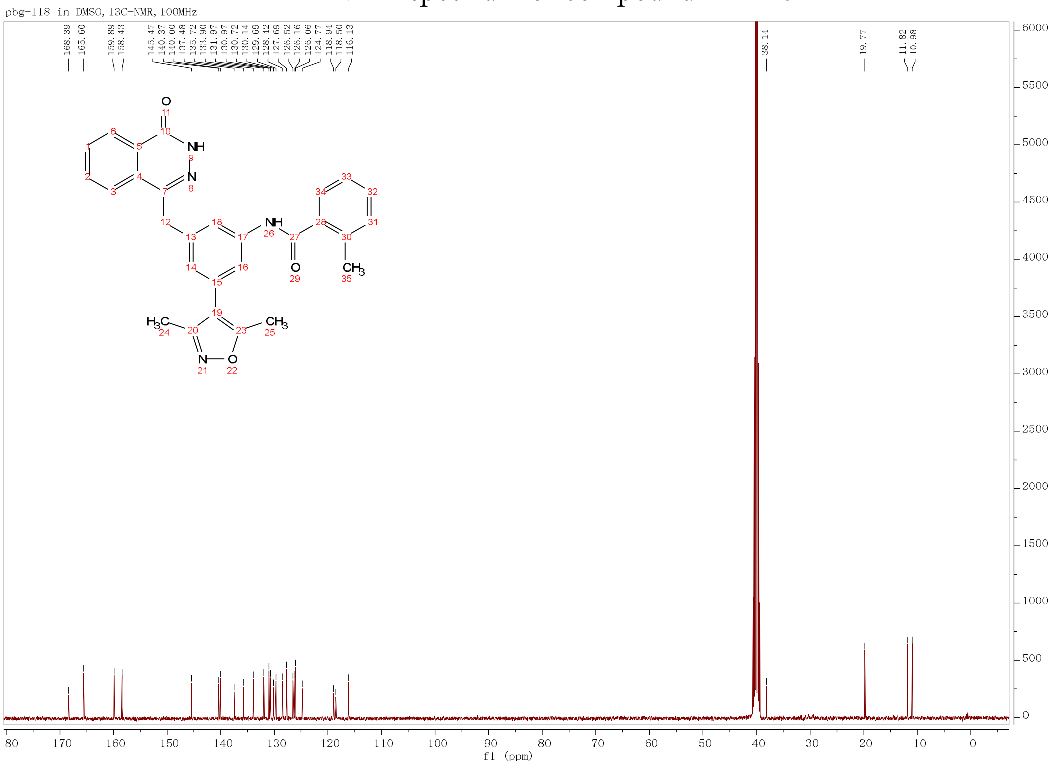

^{13}C-NMR spectrum of compound DDT23

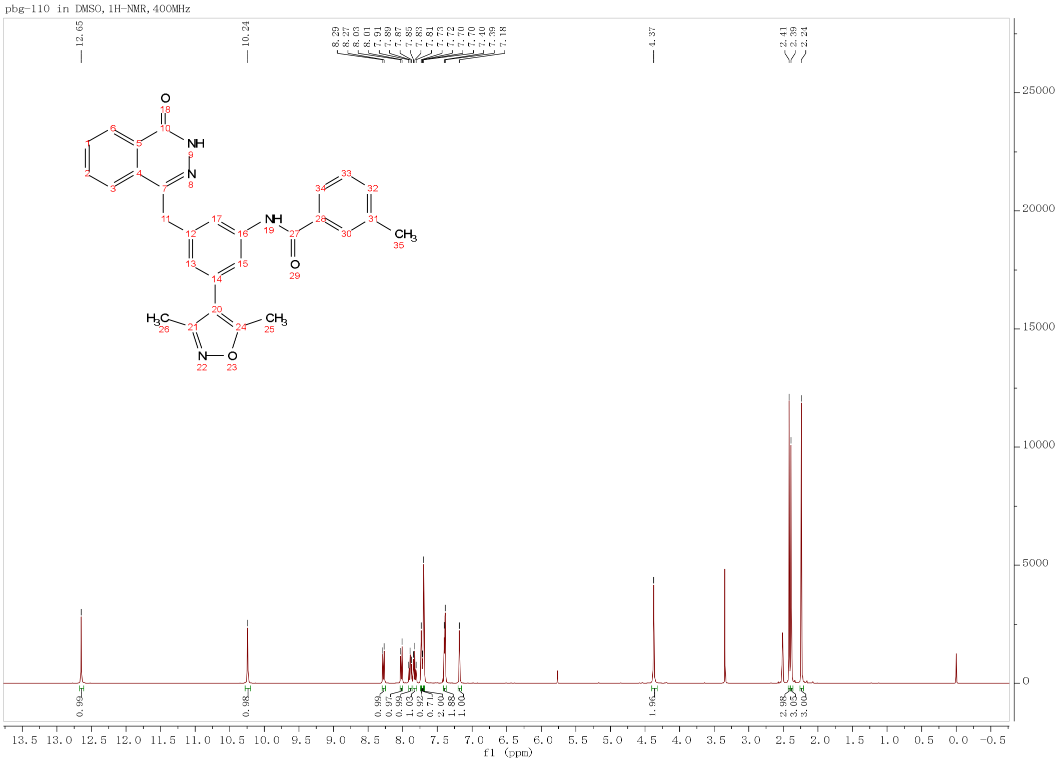

^{1}H-NMR spectrum of compound DDT24

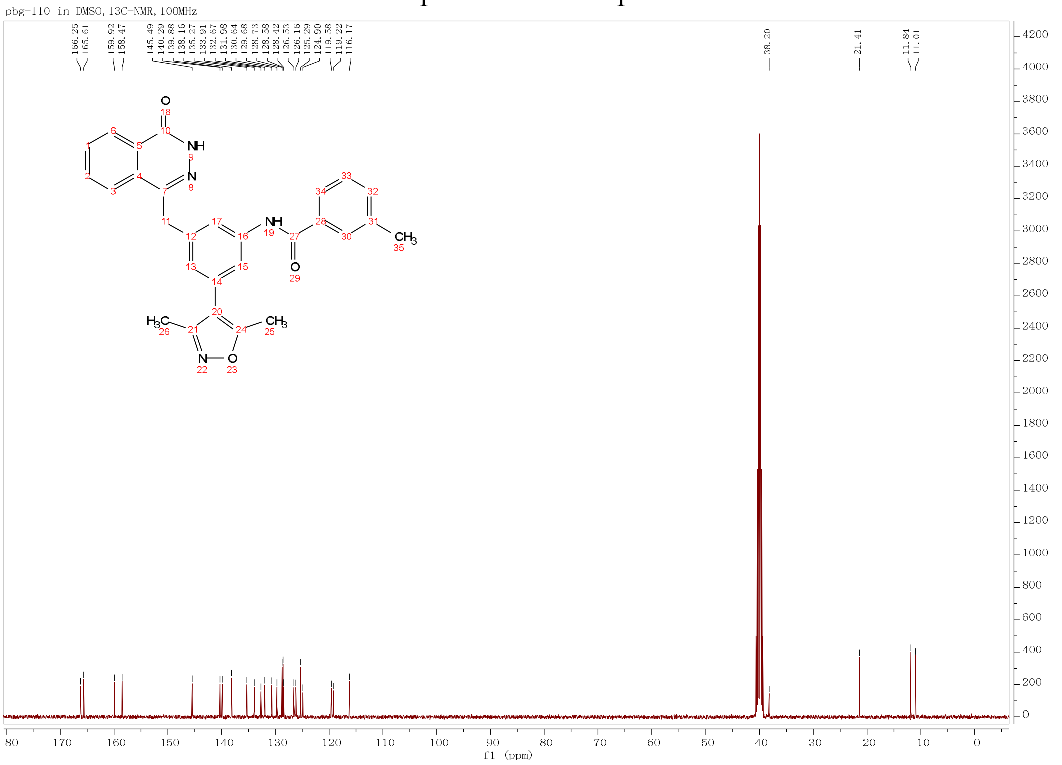

^{13}C-NMR spectrum of compound DDT24

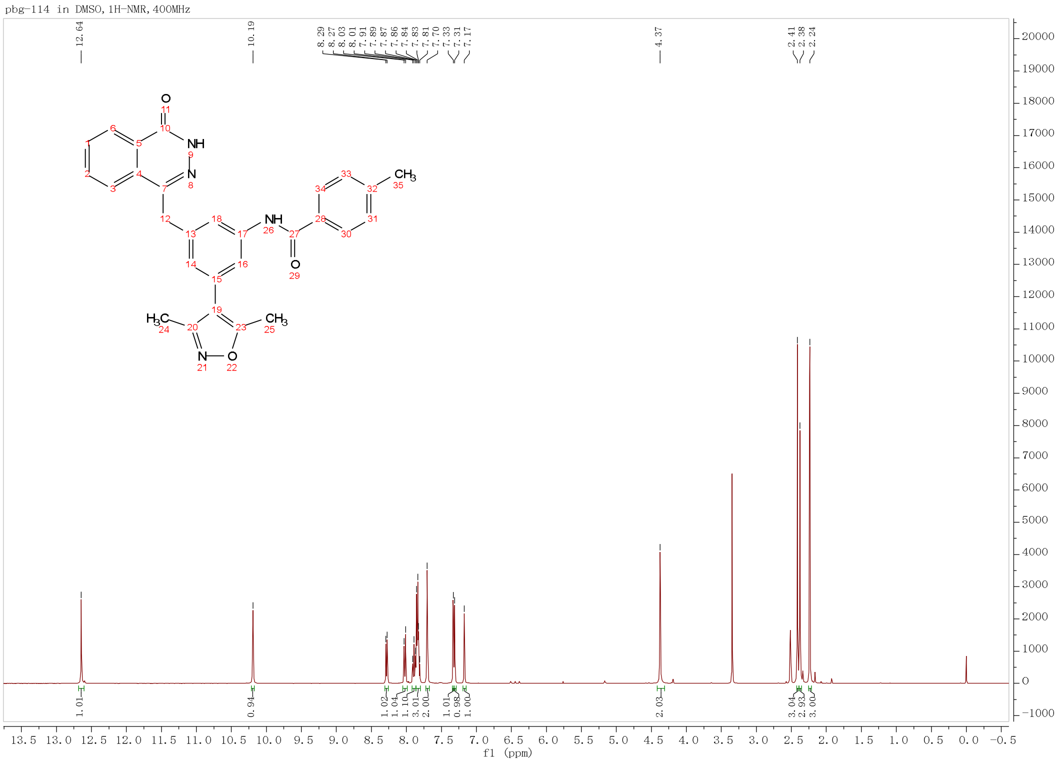

^{1}H-NMR spectrum of compound DDT25

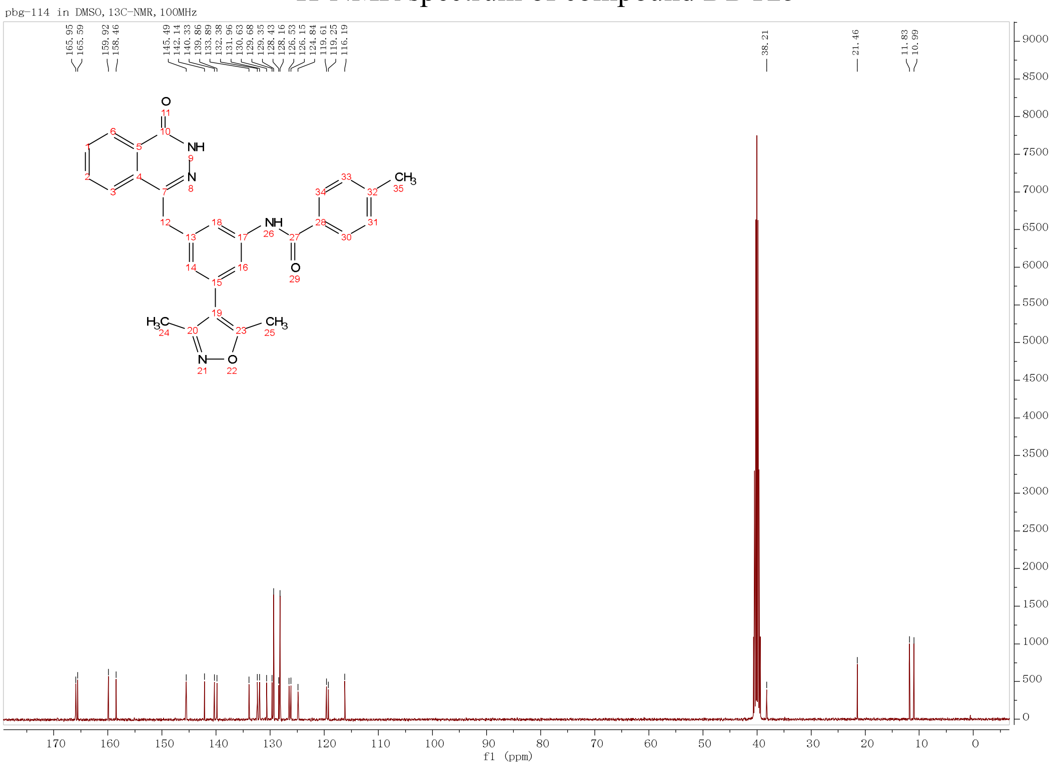

^{13}C-NMR spectrum of compound DDT25

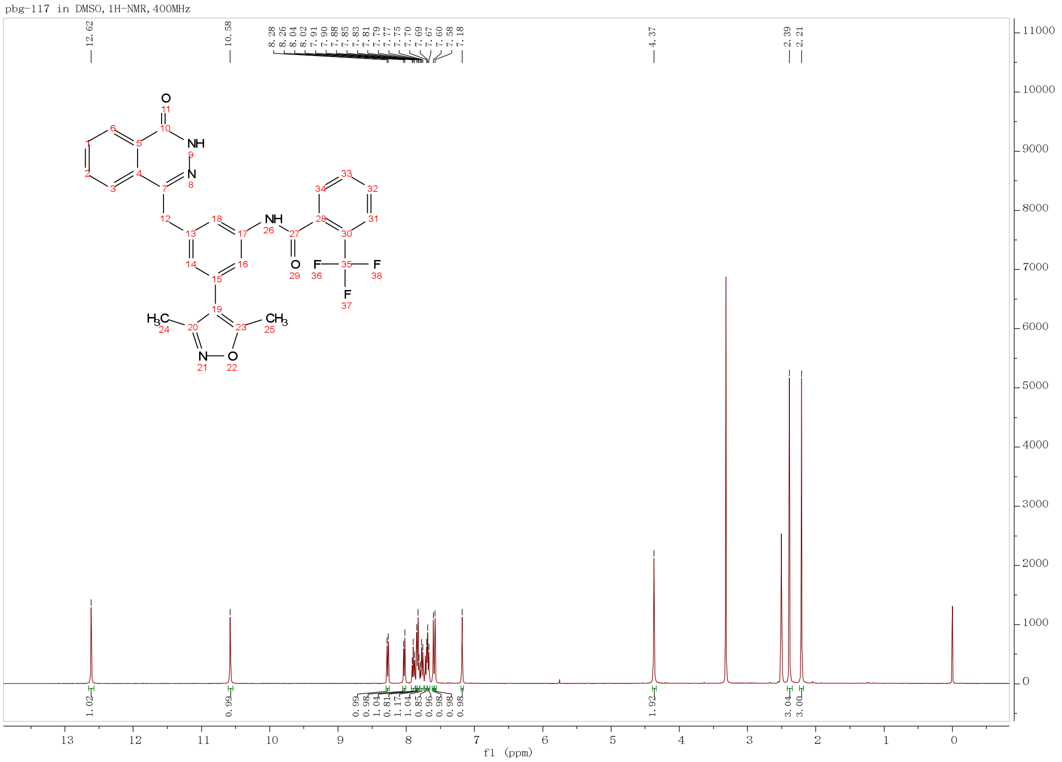

^{1}H-NMR spectrum of compound DDT26

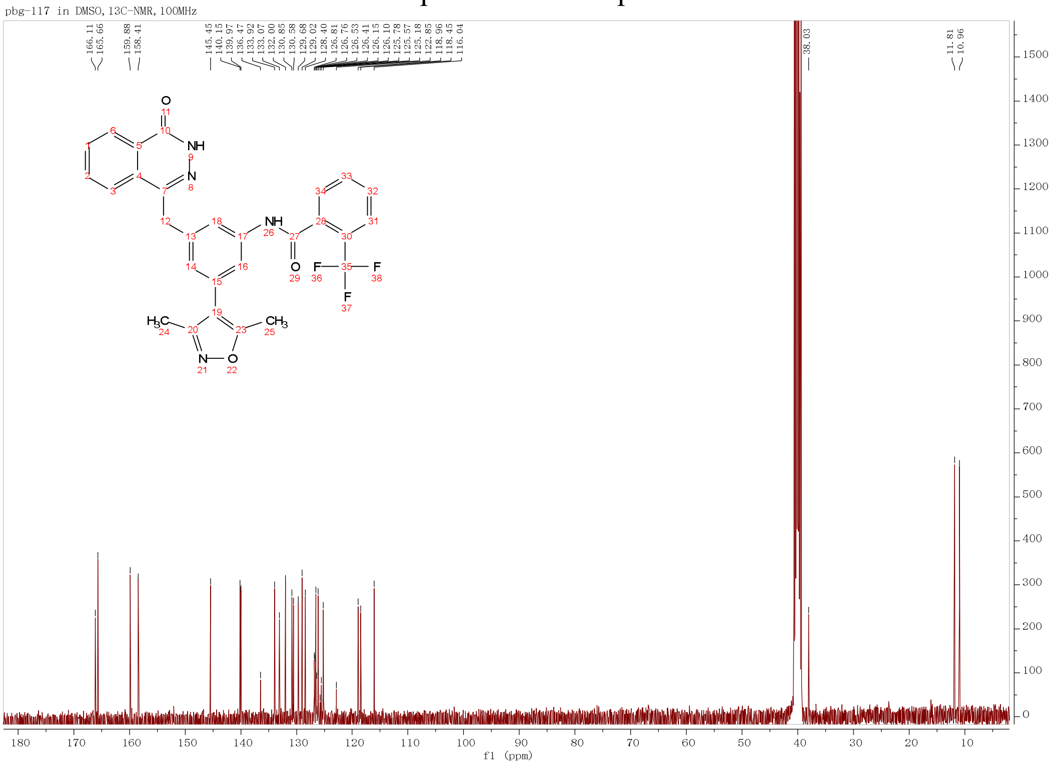

^{13}C-NMR spectrum of compound DDT26

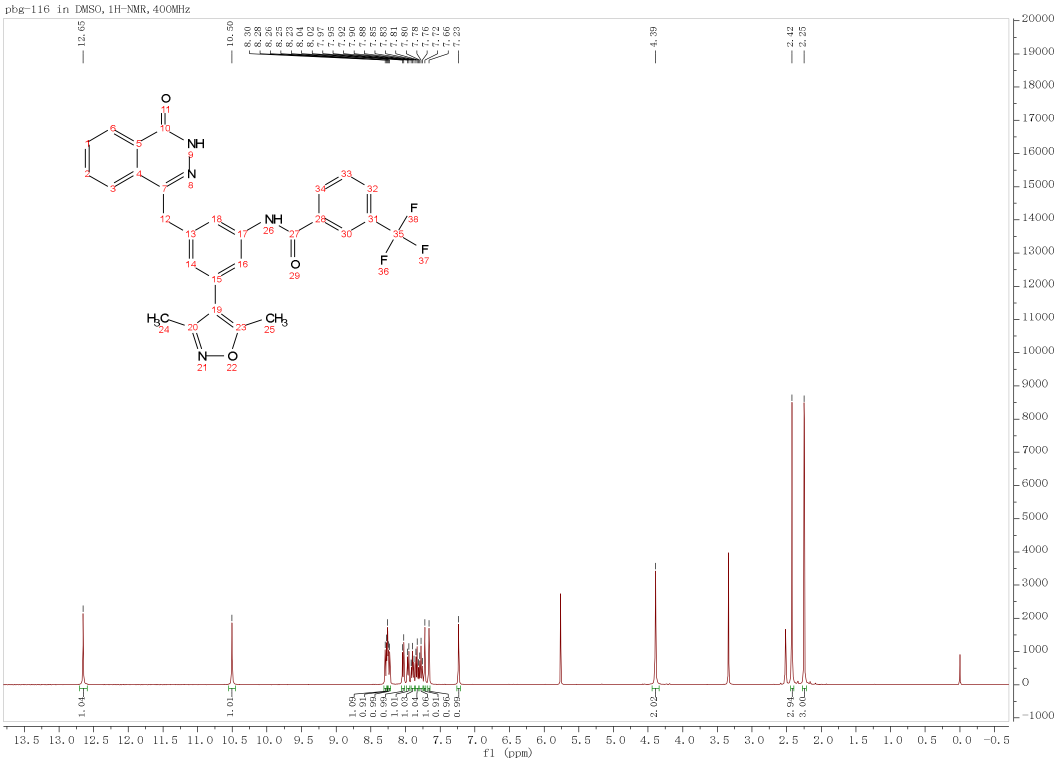

^{1}H-NMR spectrum of compound DDT27

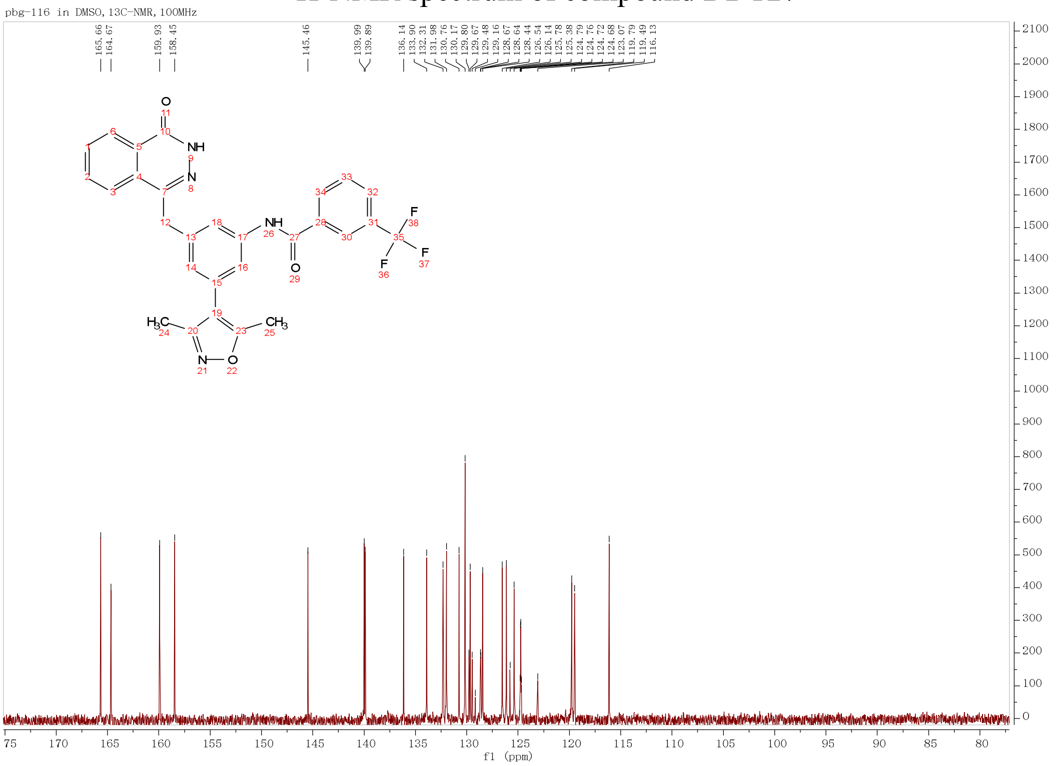

^{13}C-NMR spectrum of compound DDT27

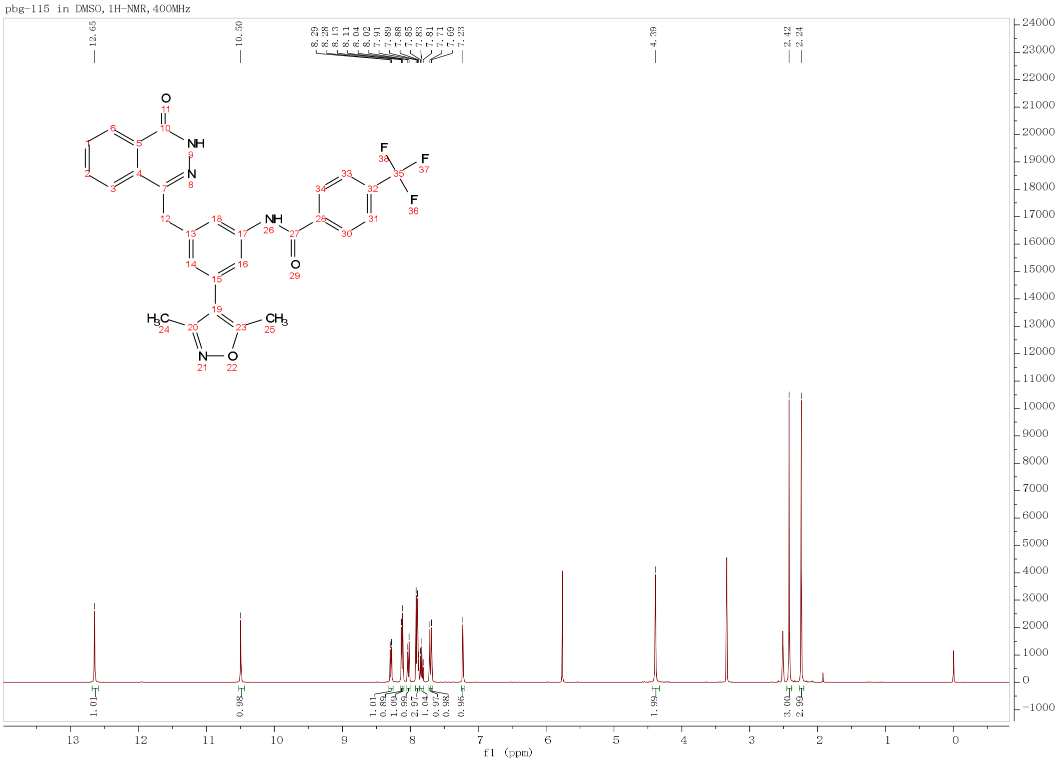

^{1}H-NMR spectrum of compound DDT28

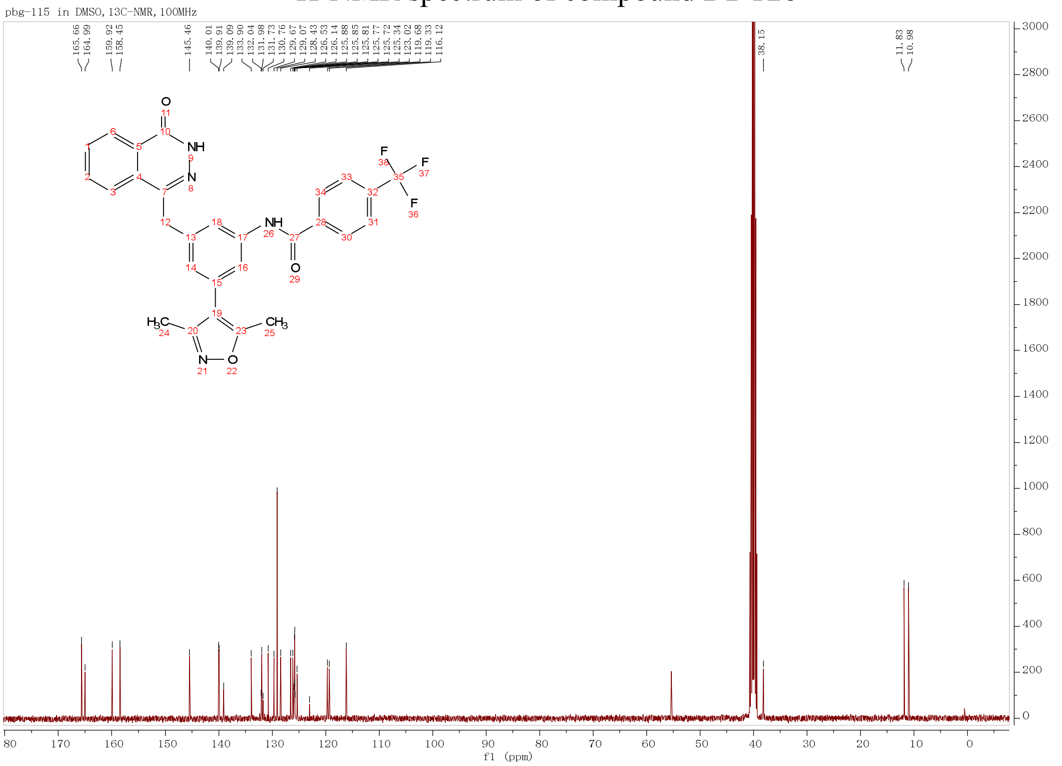

^{13}C-NMR spectrum of compound DDT28

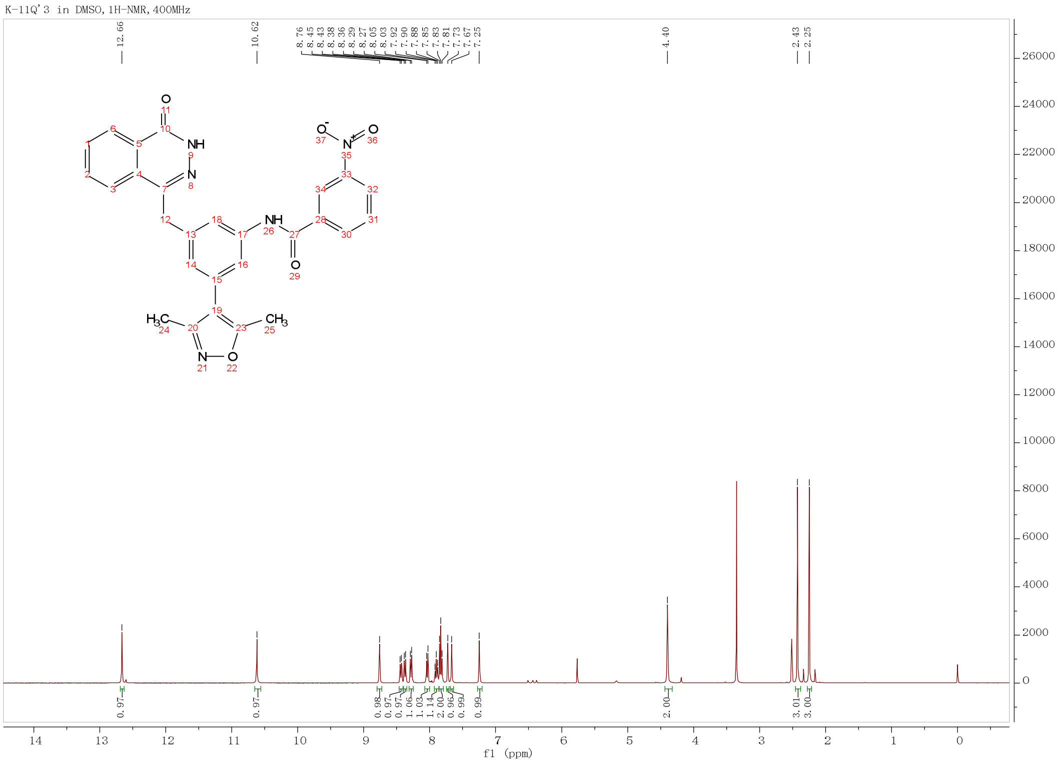

^{1}H-NMR spectrum of compound DDT29

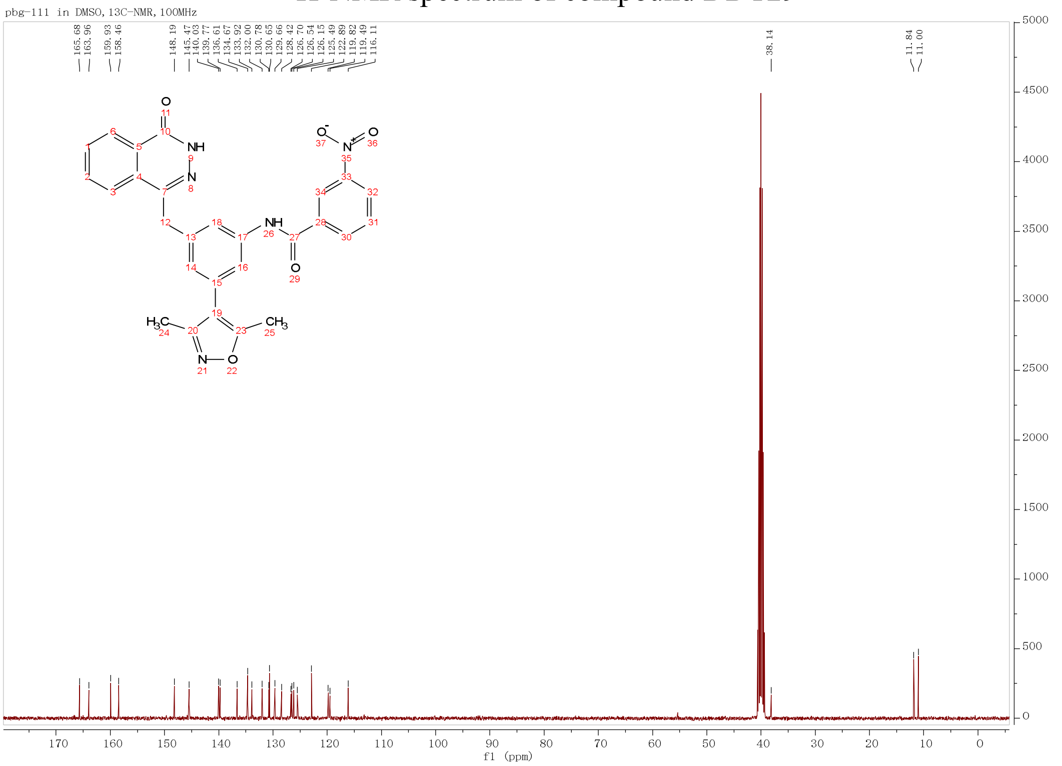

^{13}C-NMR spectrum of compound DDT29

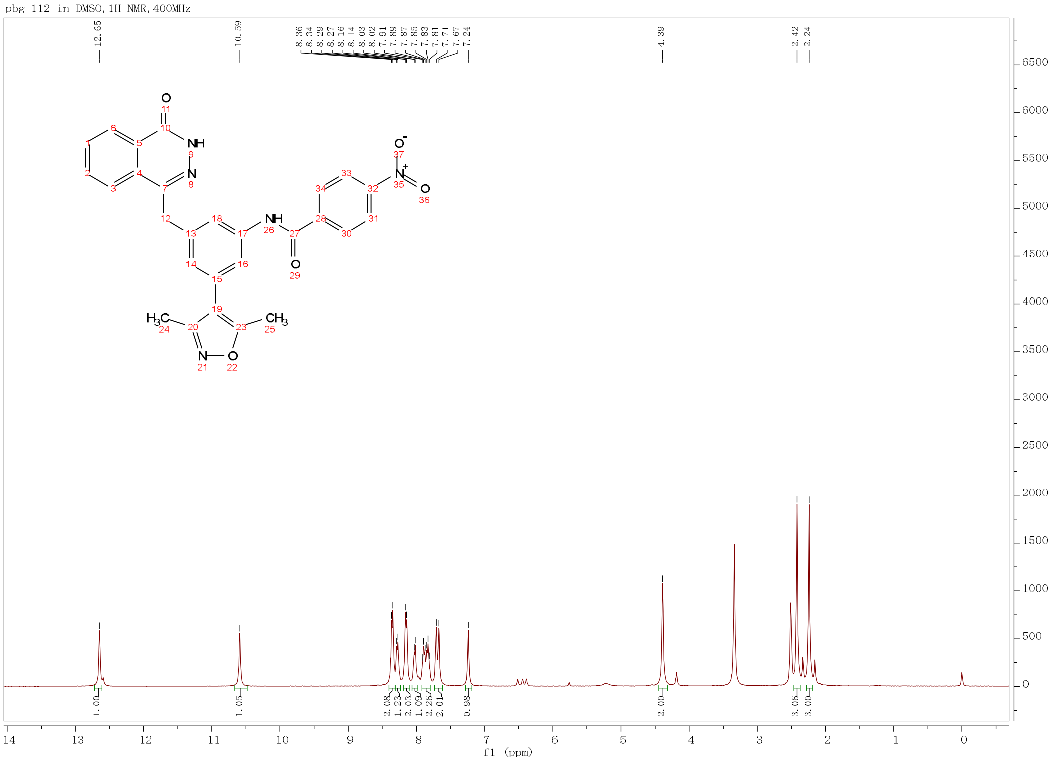

^{1}H-NMR spectrum of compound DDT30

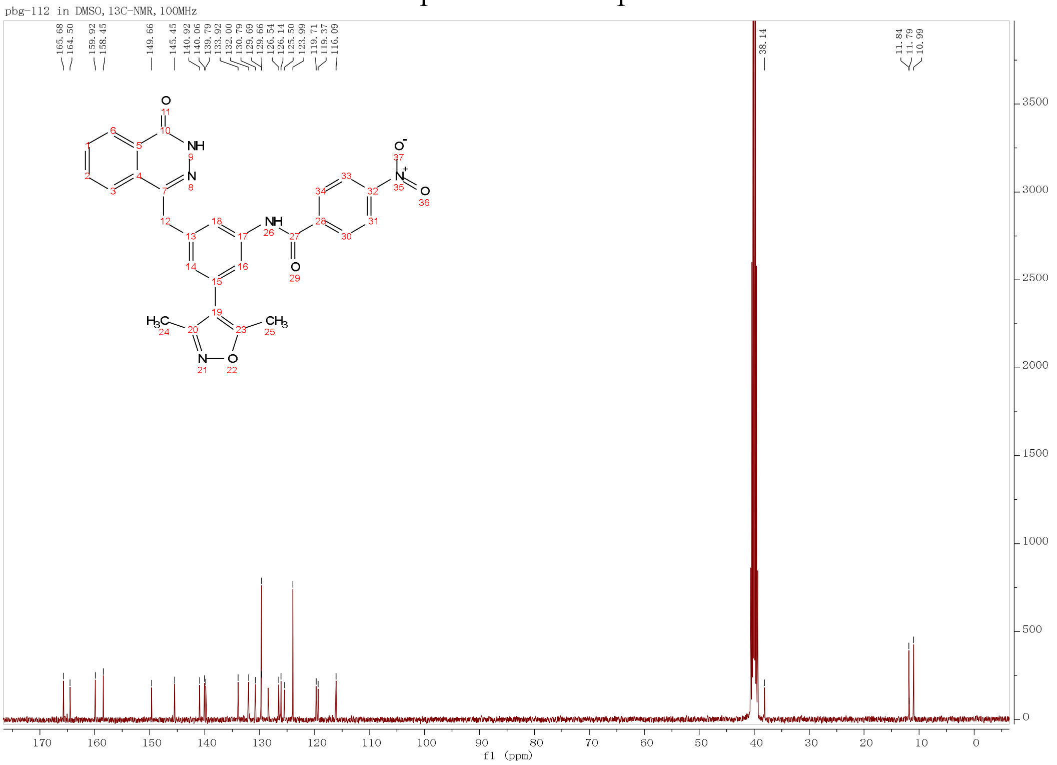

^{13}C-NMR spectrum of compound DDT30

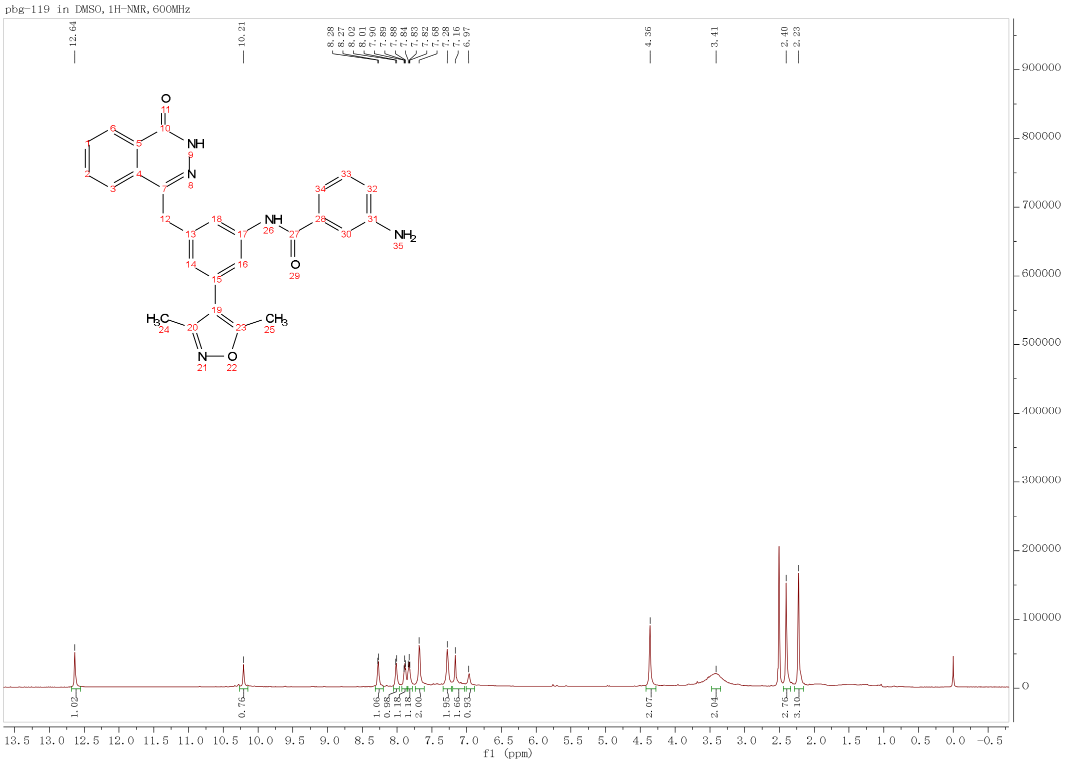

^{1}H-NMR spectrum of compound DDT31

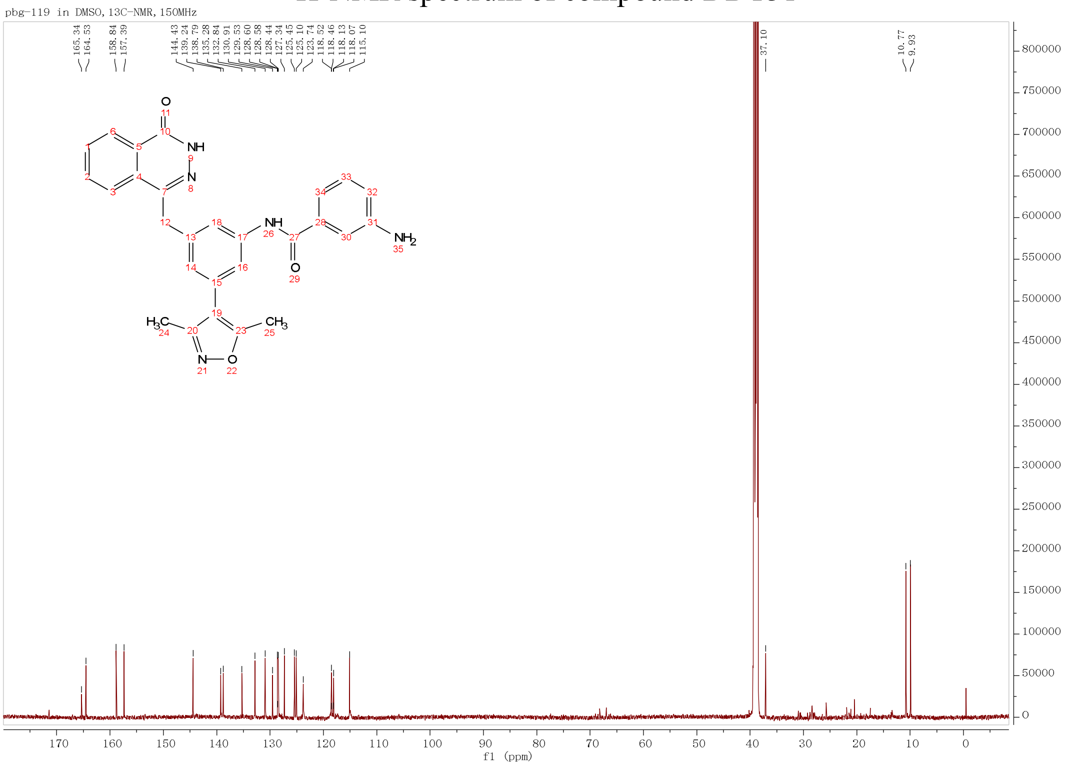

^{13}C-NMR spectrum of compound DDT31

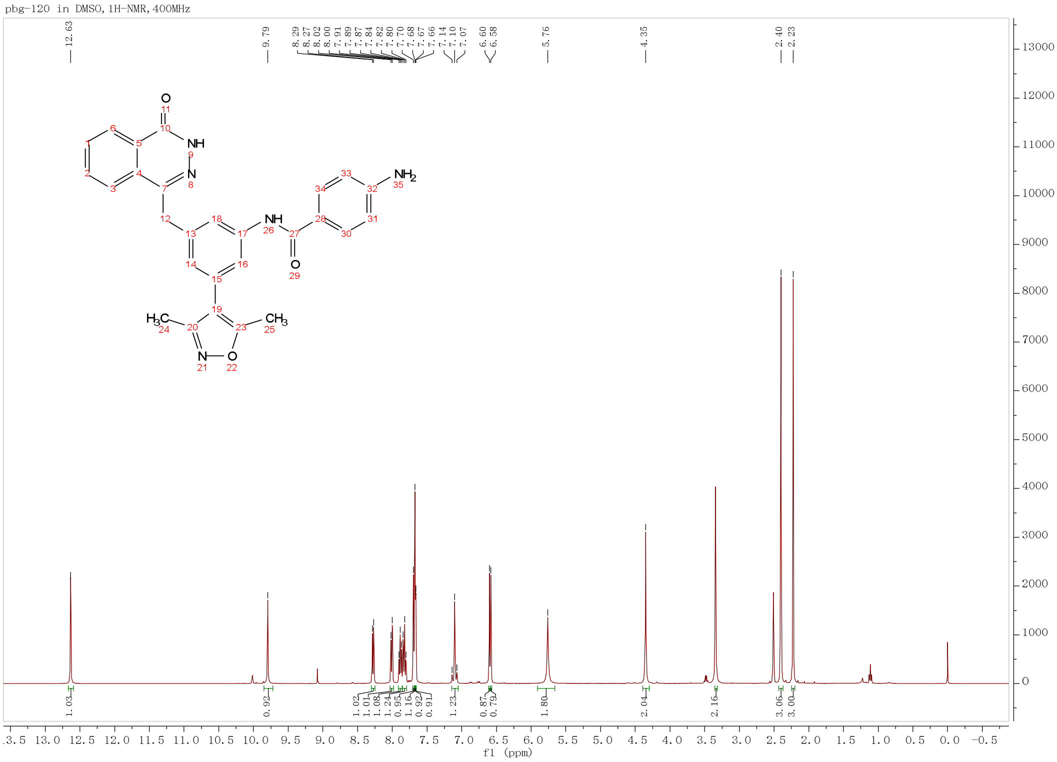

^{1}H-NMR spectrum of compound DDT32

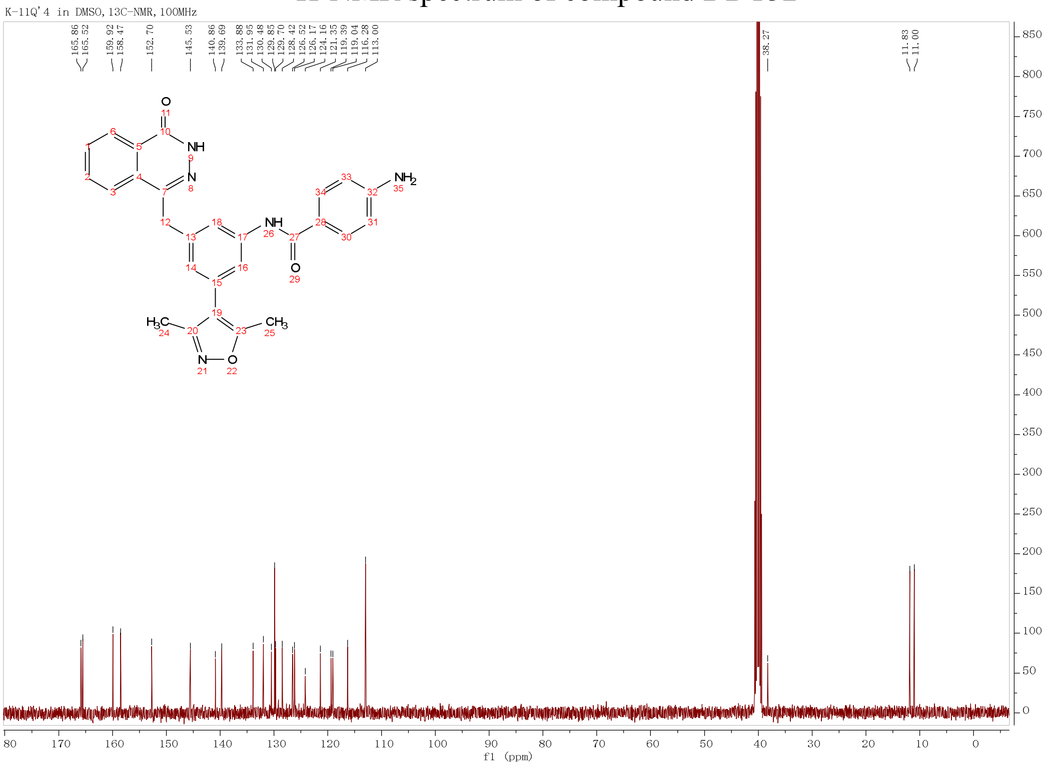

^{13}C-NMR spectrum of compound DDT32

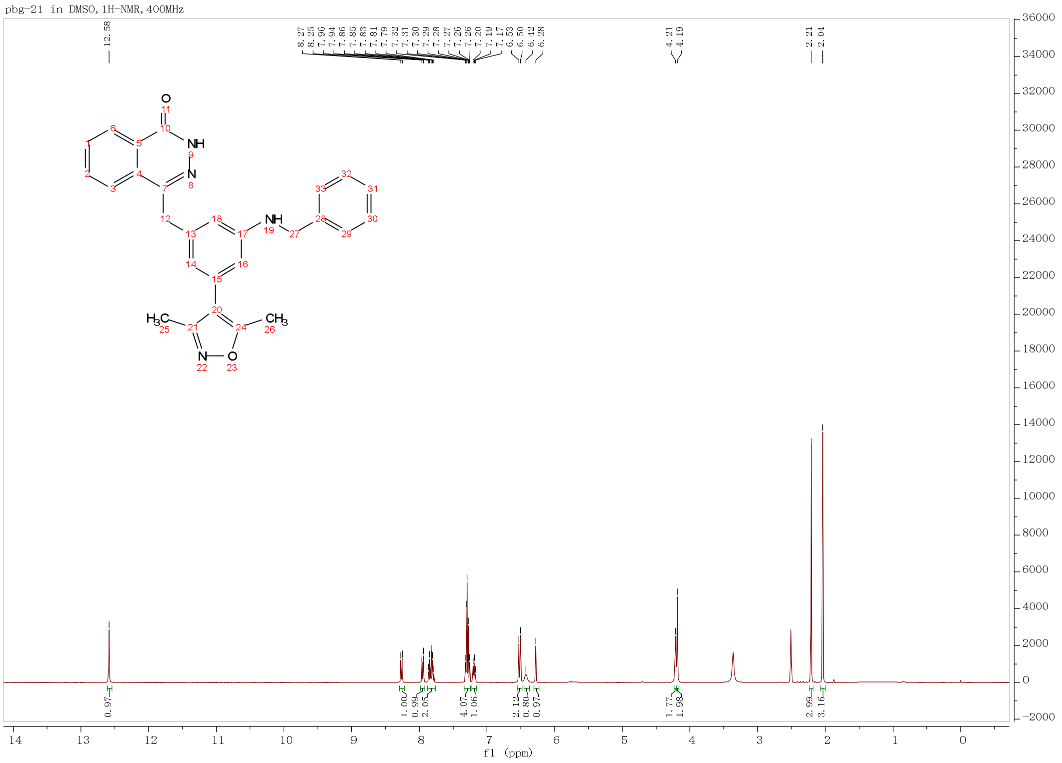

^{1}H-NMR spectrum of compound DDT33

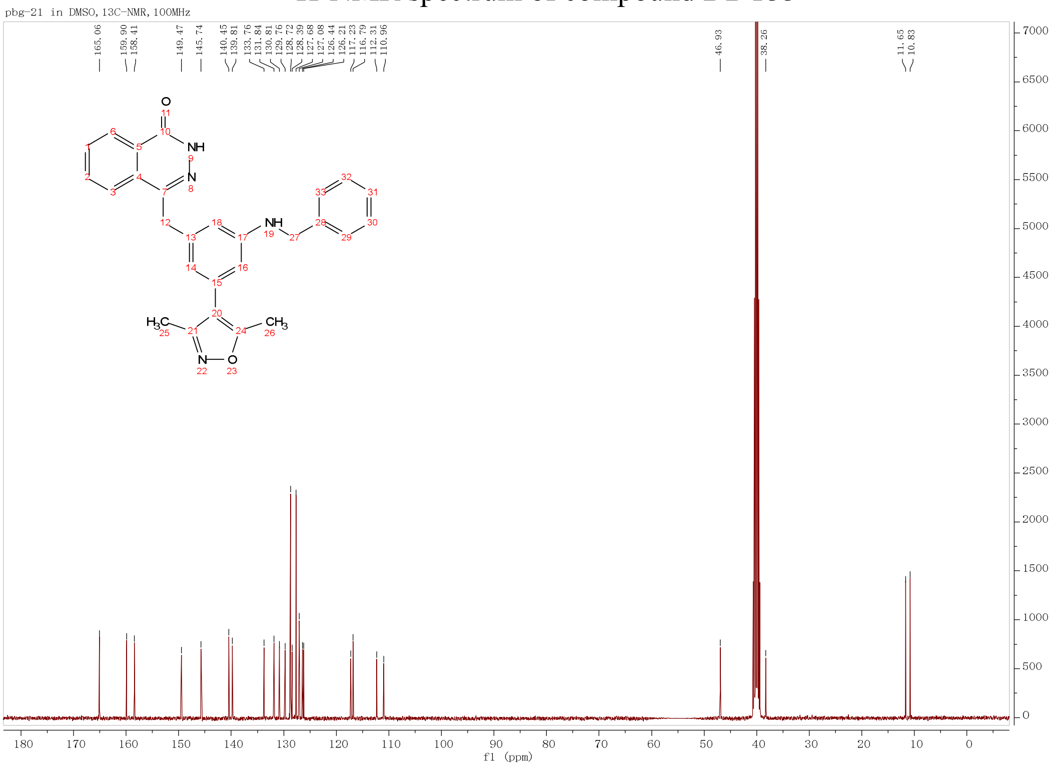

^{13}C-NMR spectrum of compound DDT33

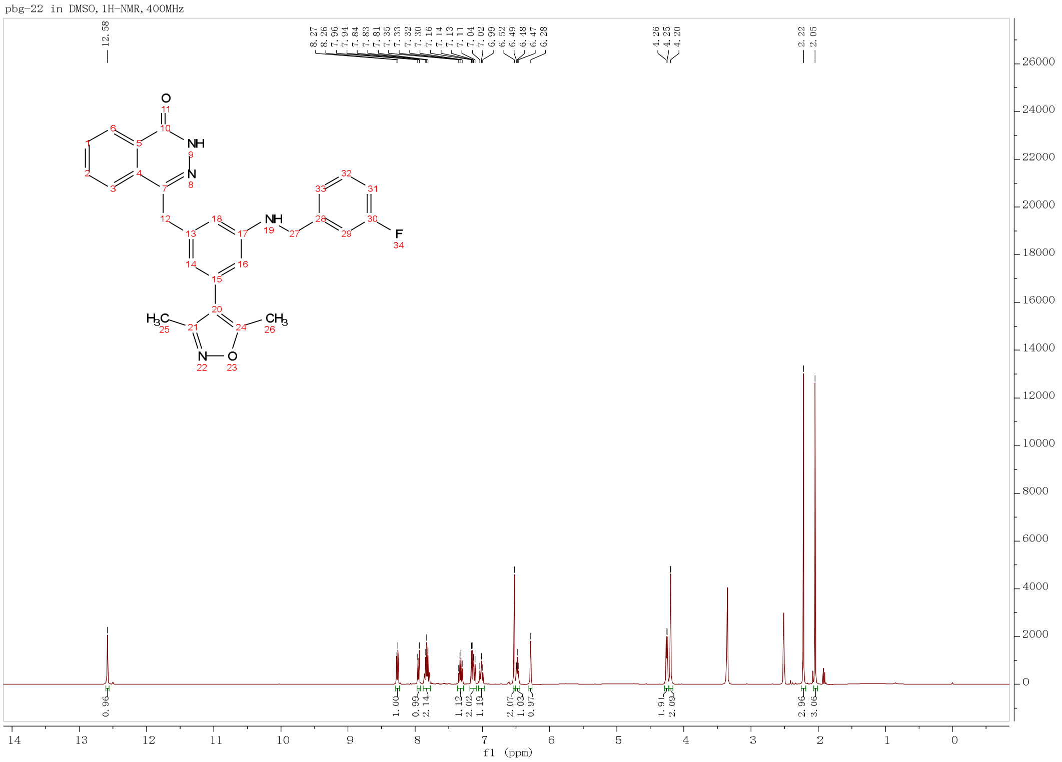

^{1}H-NMR spectrum of compound DDT34

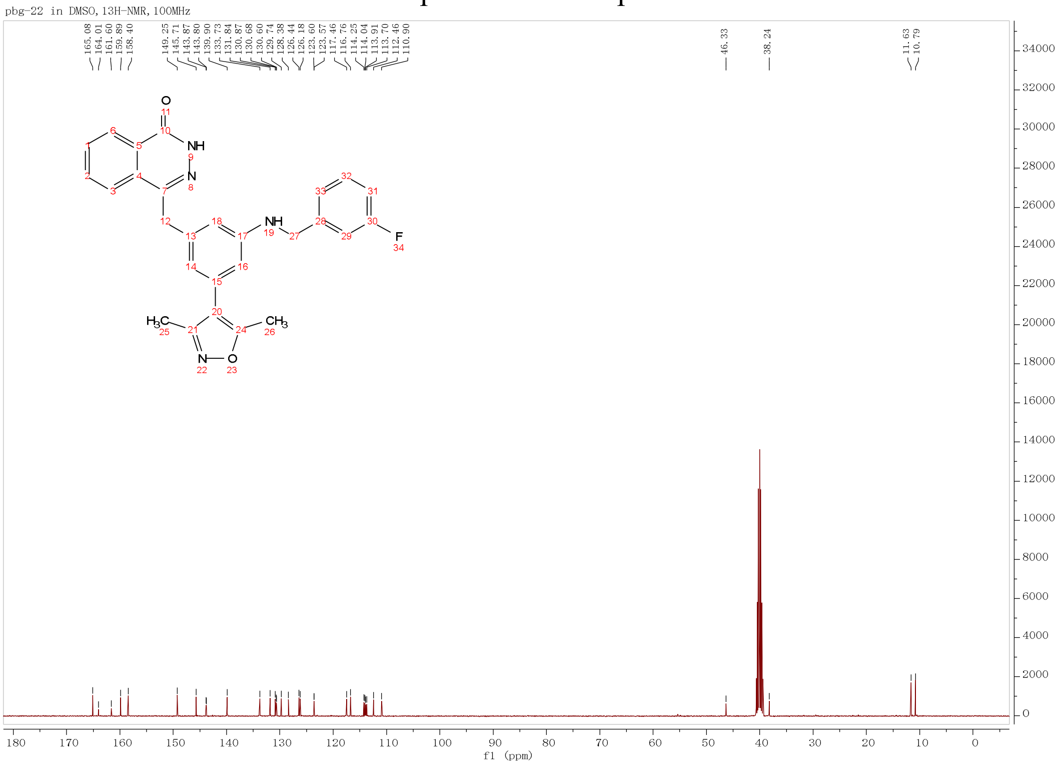

^{13}C-NMR spectrum of compound DDT34

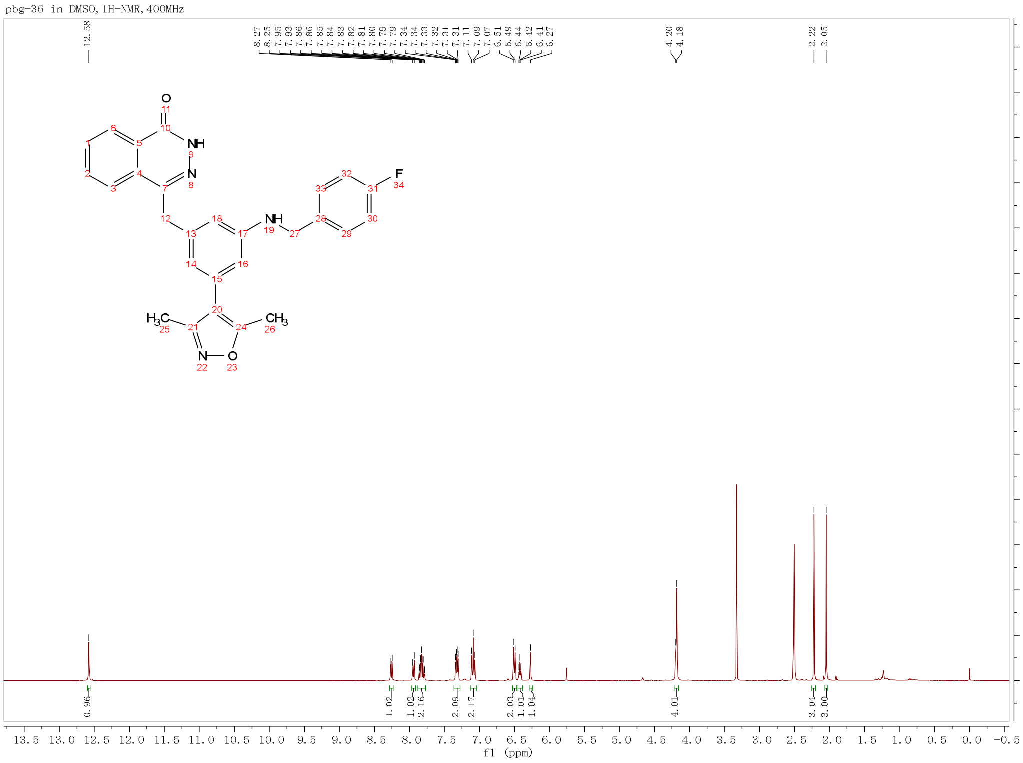

^{1}H-NMR spectrum of compound DDT35

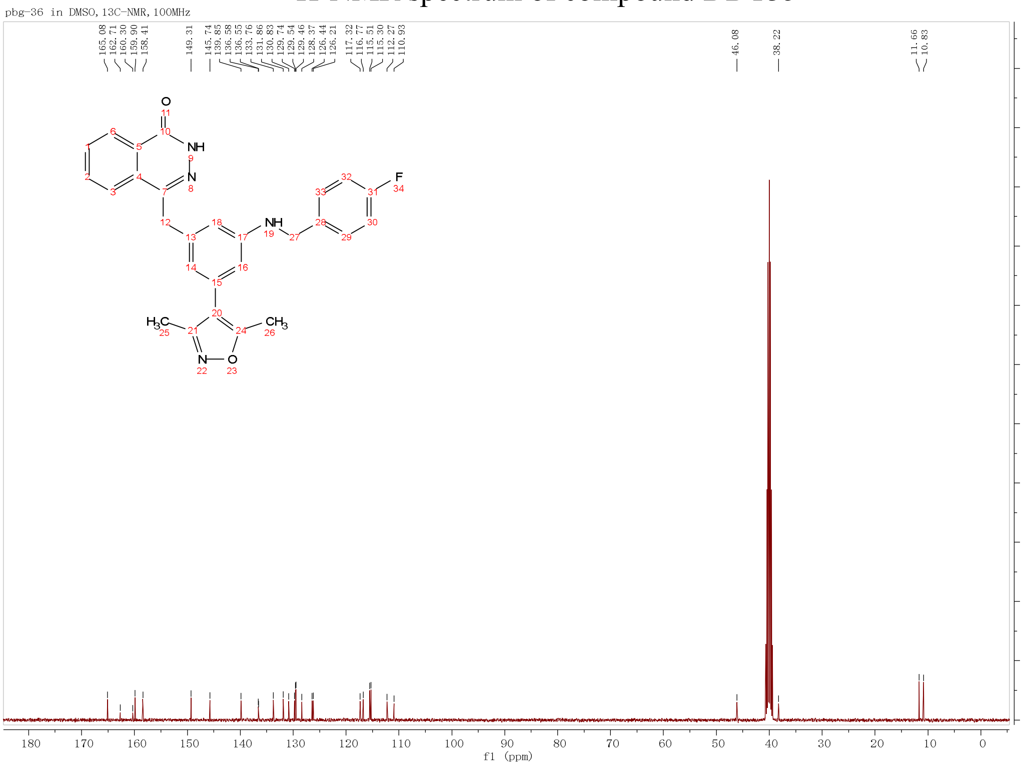

^{13}C-NMR spectrum of compound DDT35

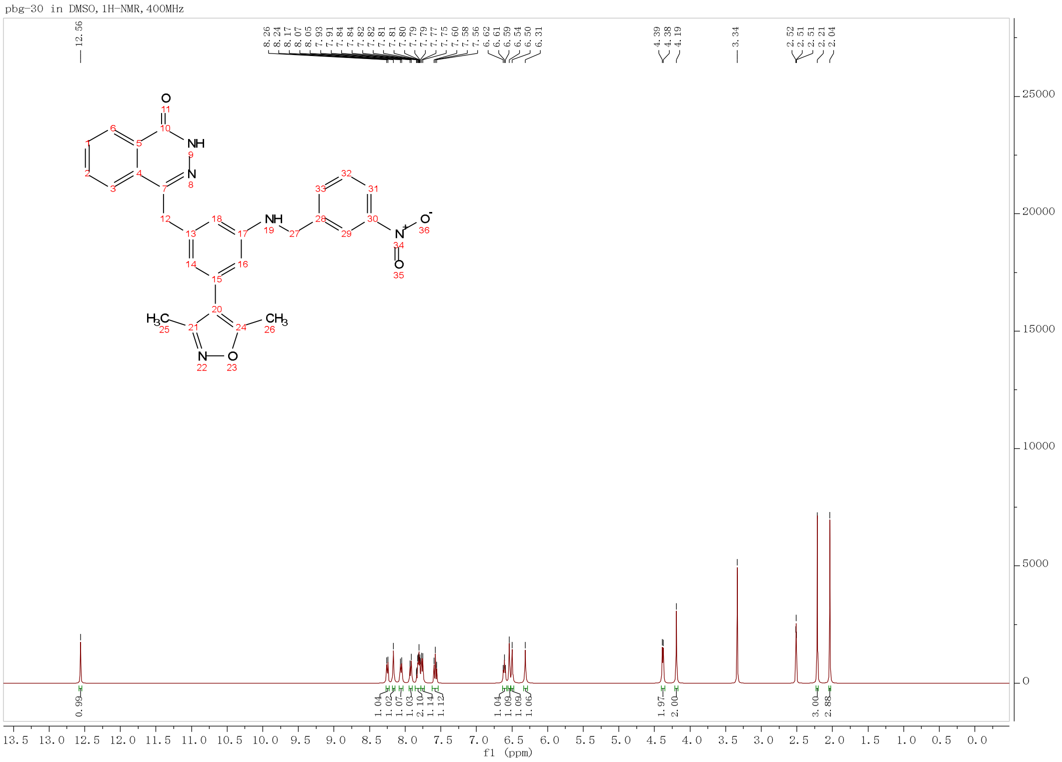

^{1}H-NMR spectrum of compound DDT36

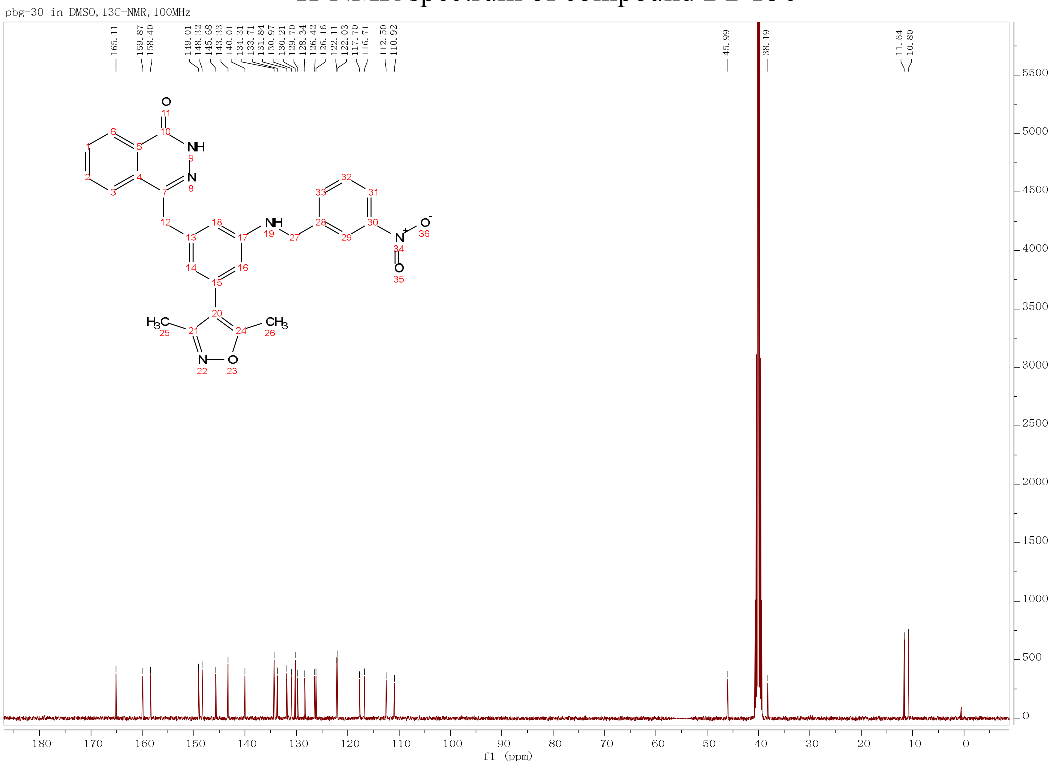

^{13}C-NMR spectrum of compound DDT36

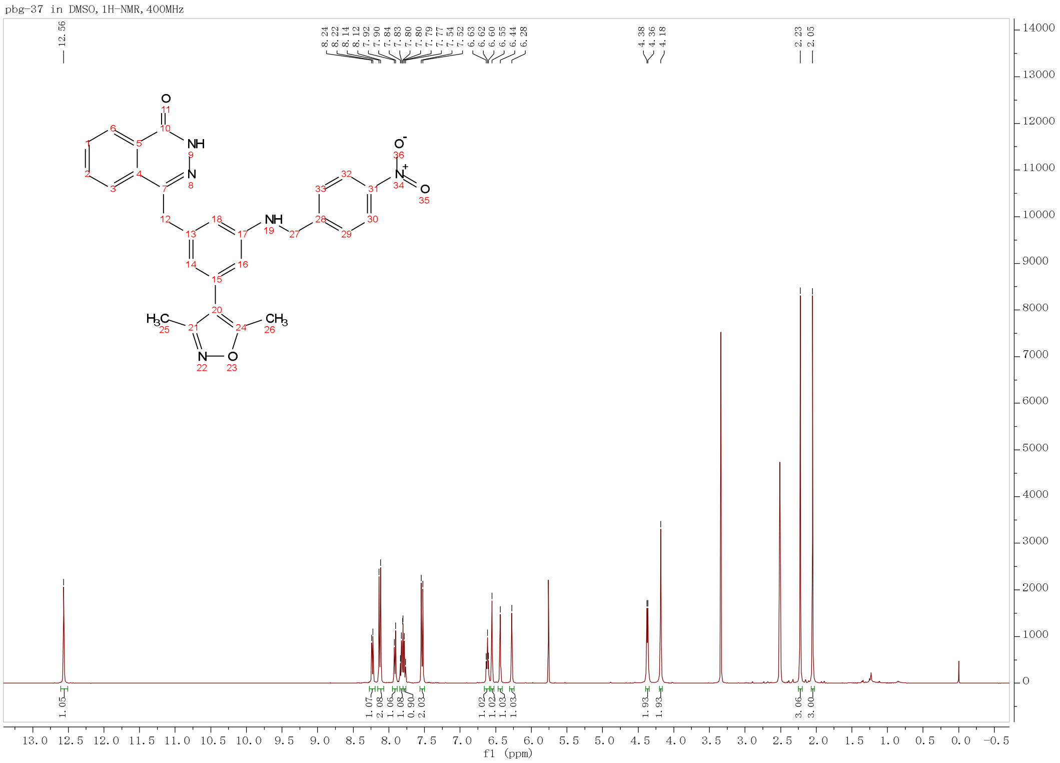

^{1}H-NMR spectrum of compound DDT37

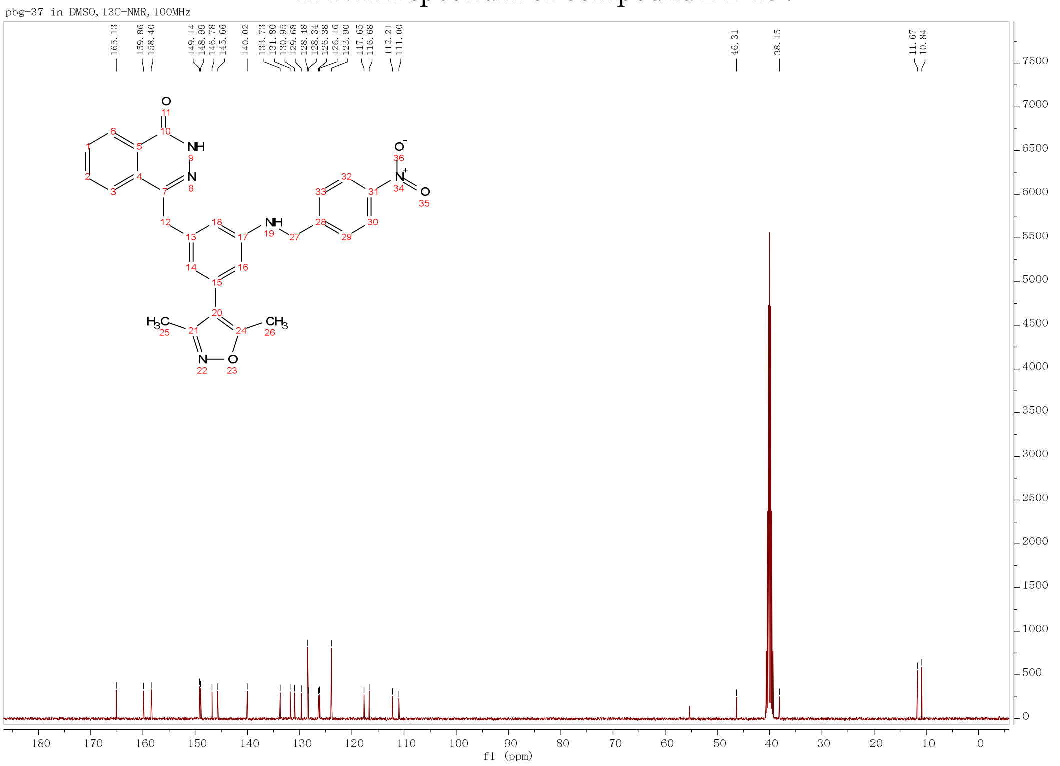

^{13}C-NMR spectrum of compound DDT37

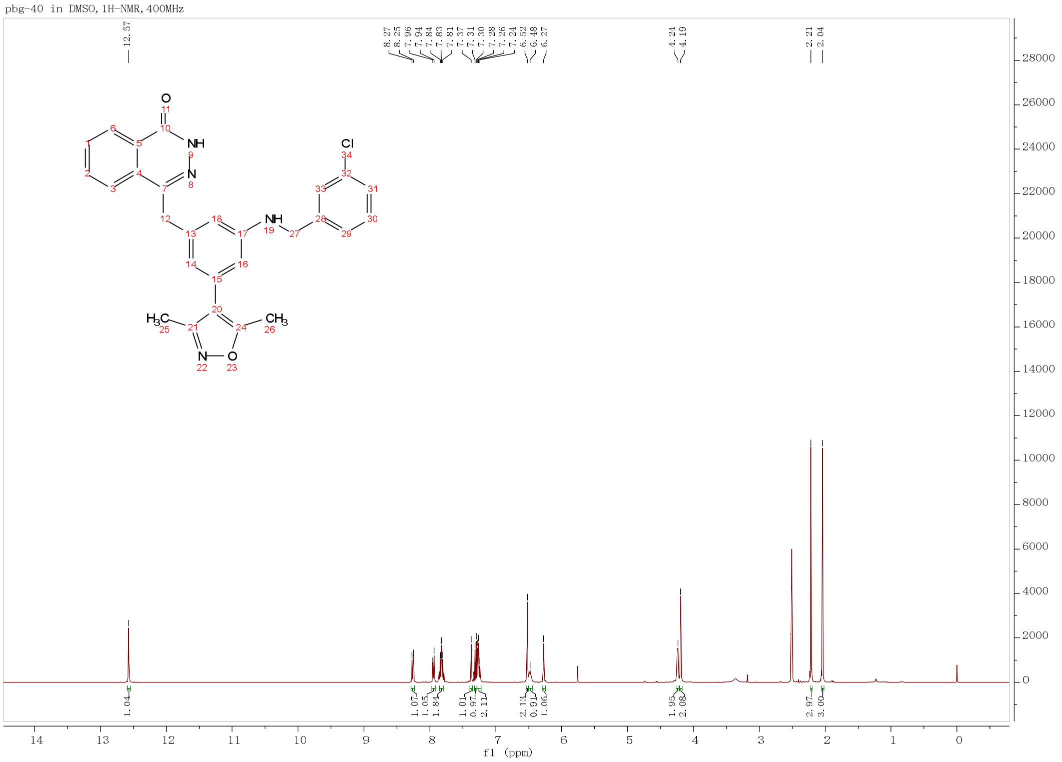

^{1}H-NMR spectrum of compound DDT38

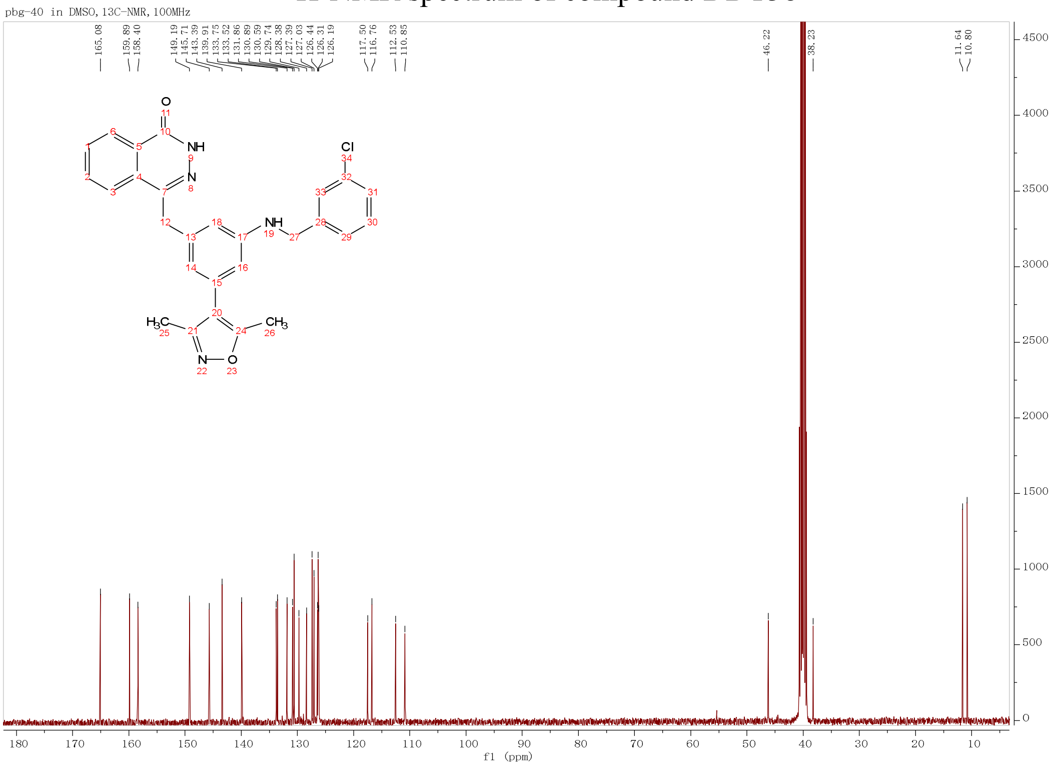

^{13}C-NMR spectrum of compound DDT38

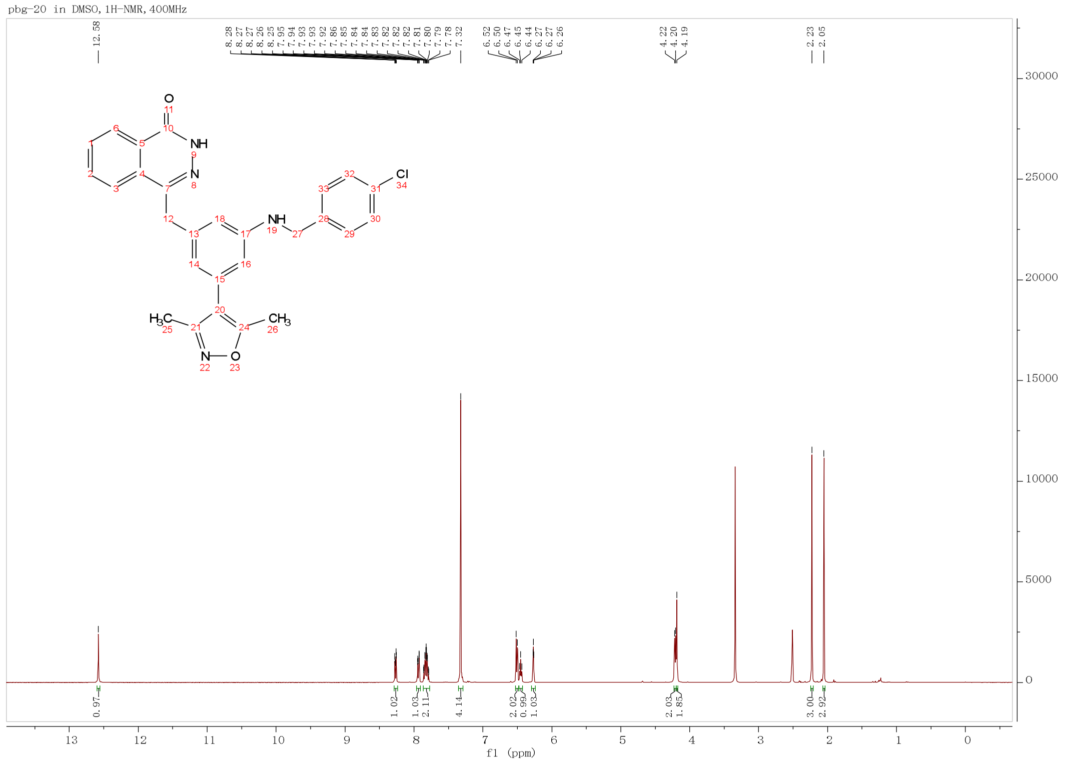

^{1}H-NMR spectrum of compound DDT39

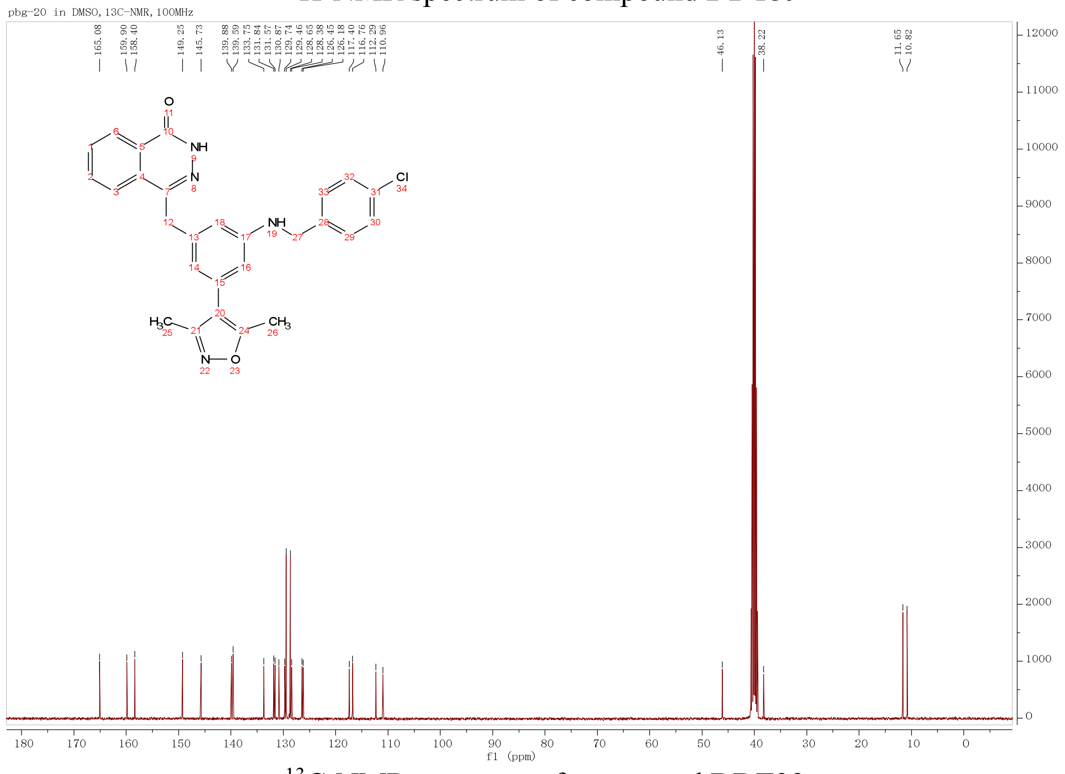

^{13}C-NMR spectrum of compound DDT39

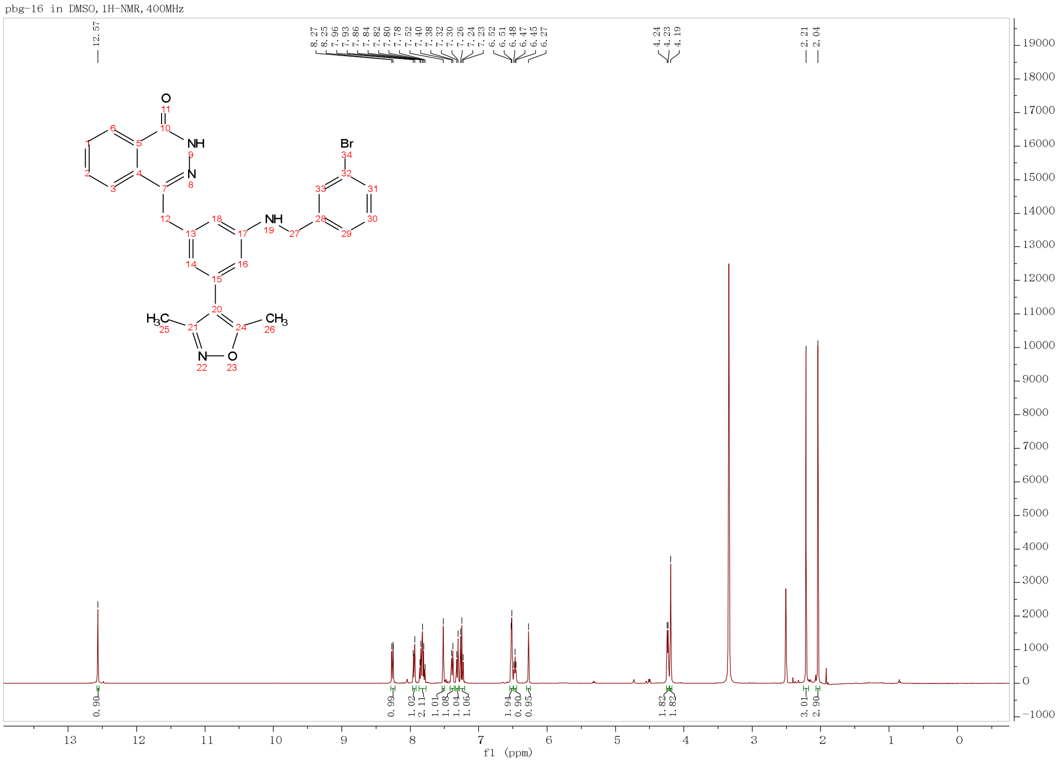

^{1}H-NMR spectrum of compound DDT40

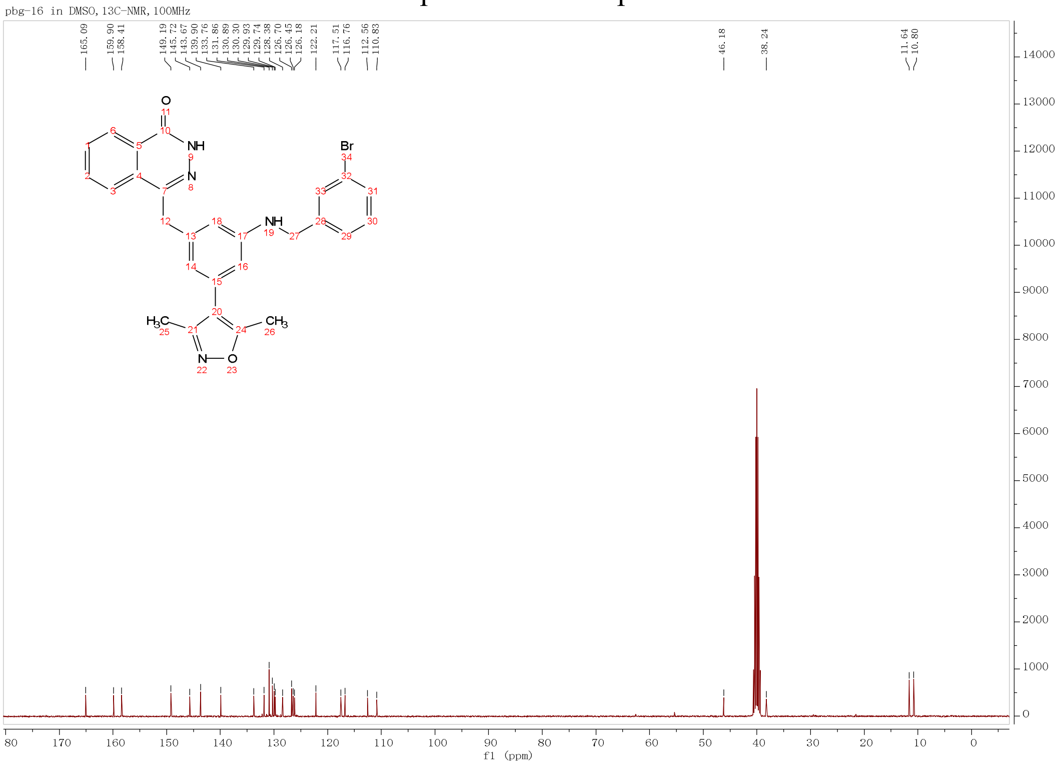

^{13}C-NMR spectrum of compound DDT40

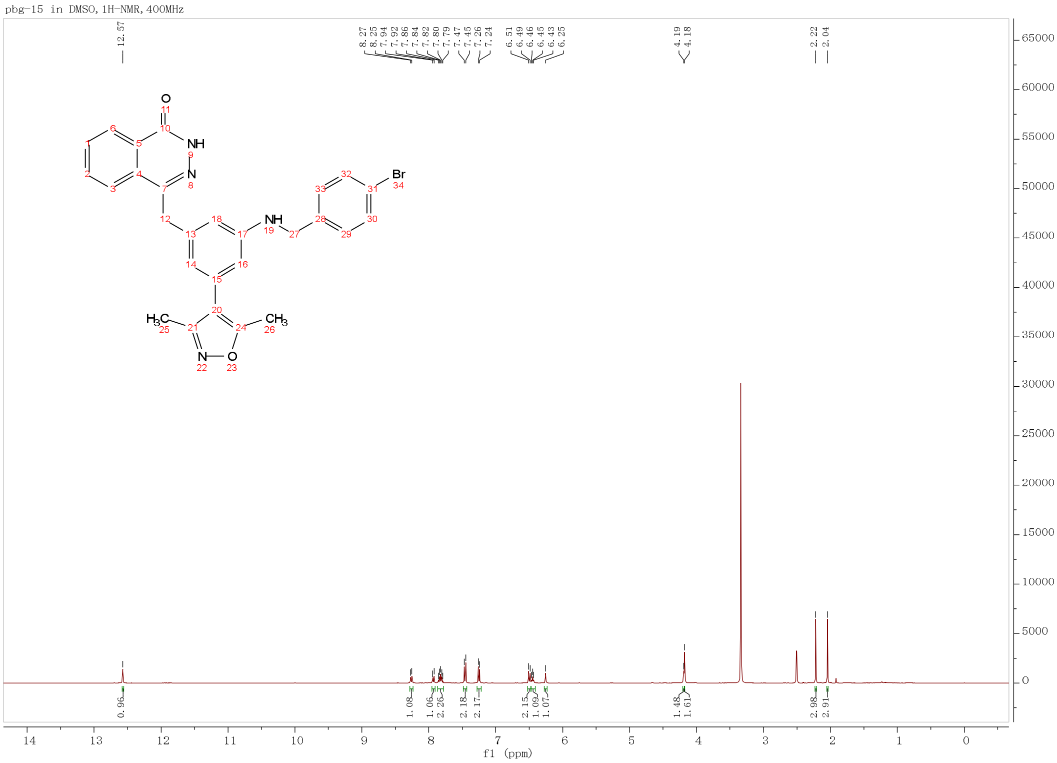

^{1}H-NMR spectrum of compound DDT41

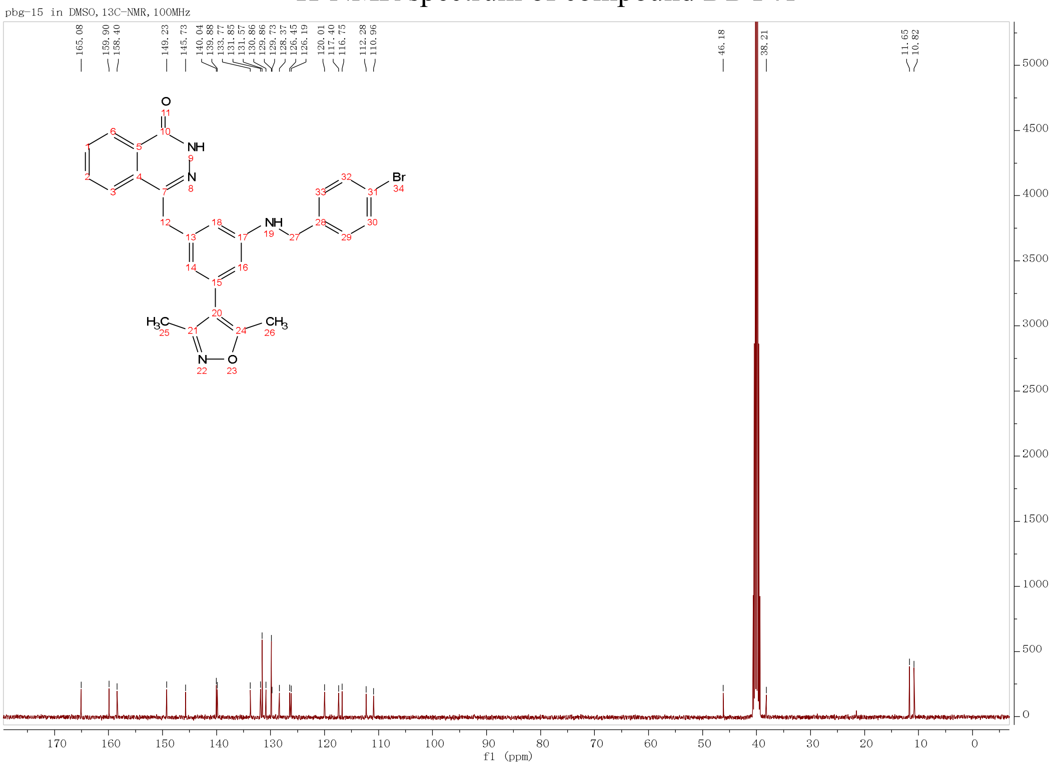

^{13}C-NMR spectrum of compound DDT41

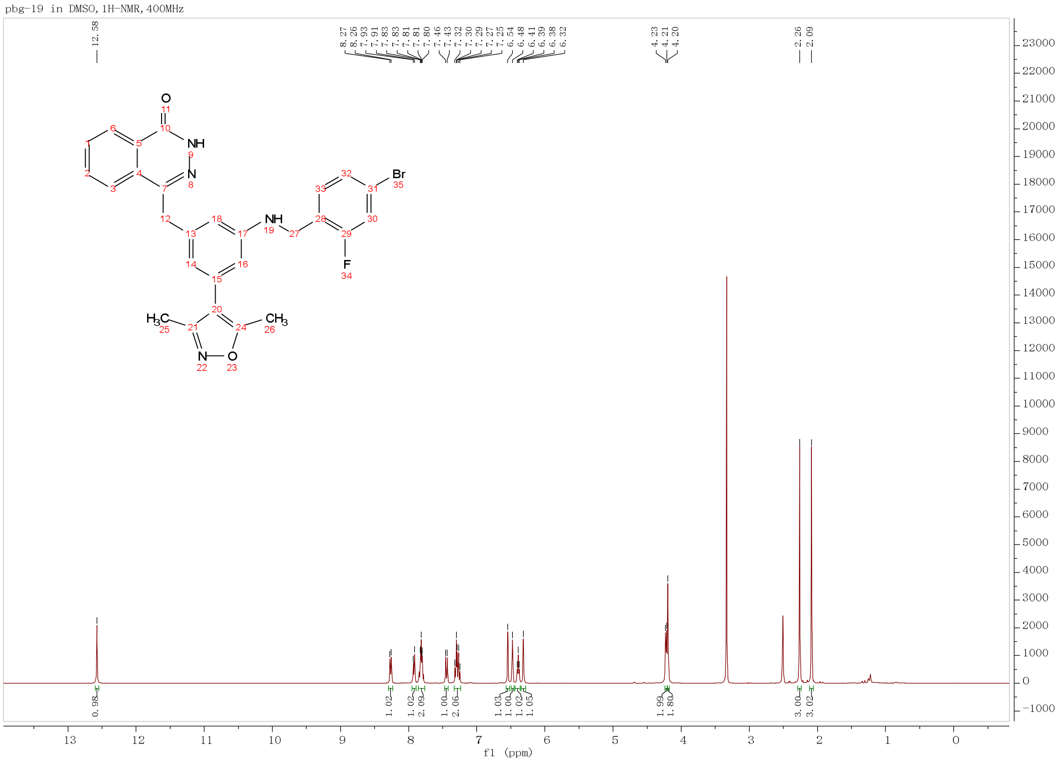

^{1}H-NMR spectrum of compound DDT42

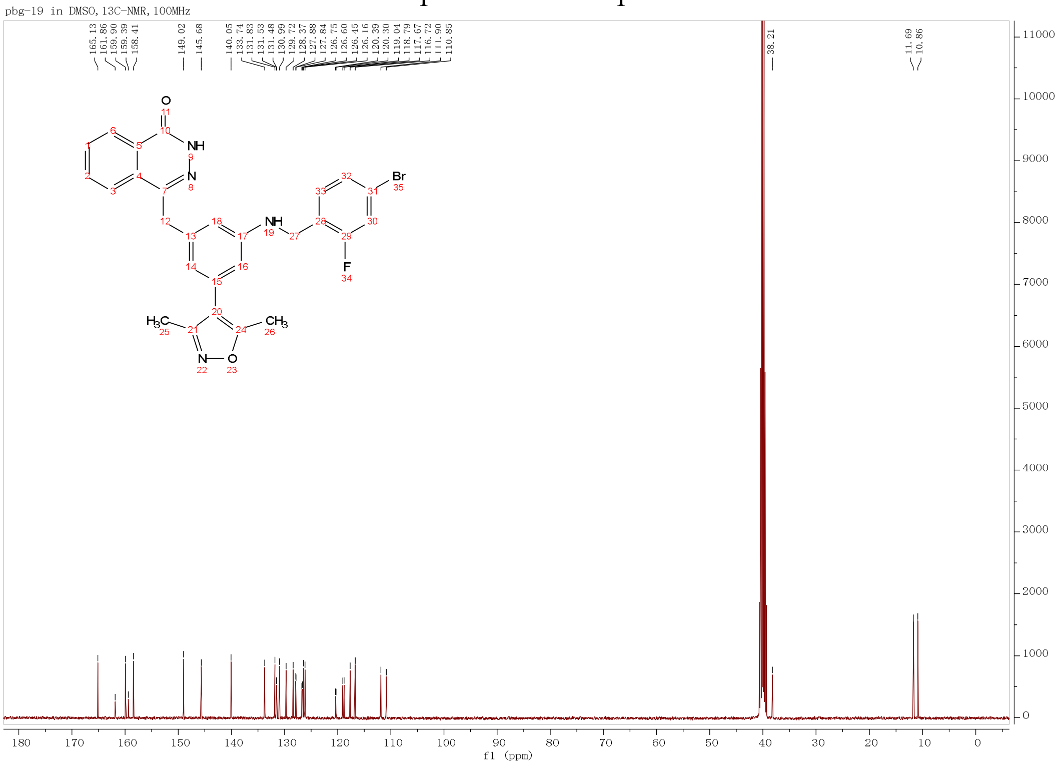

^{13}C-NMR spectrum of compound DDT42

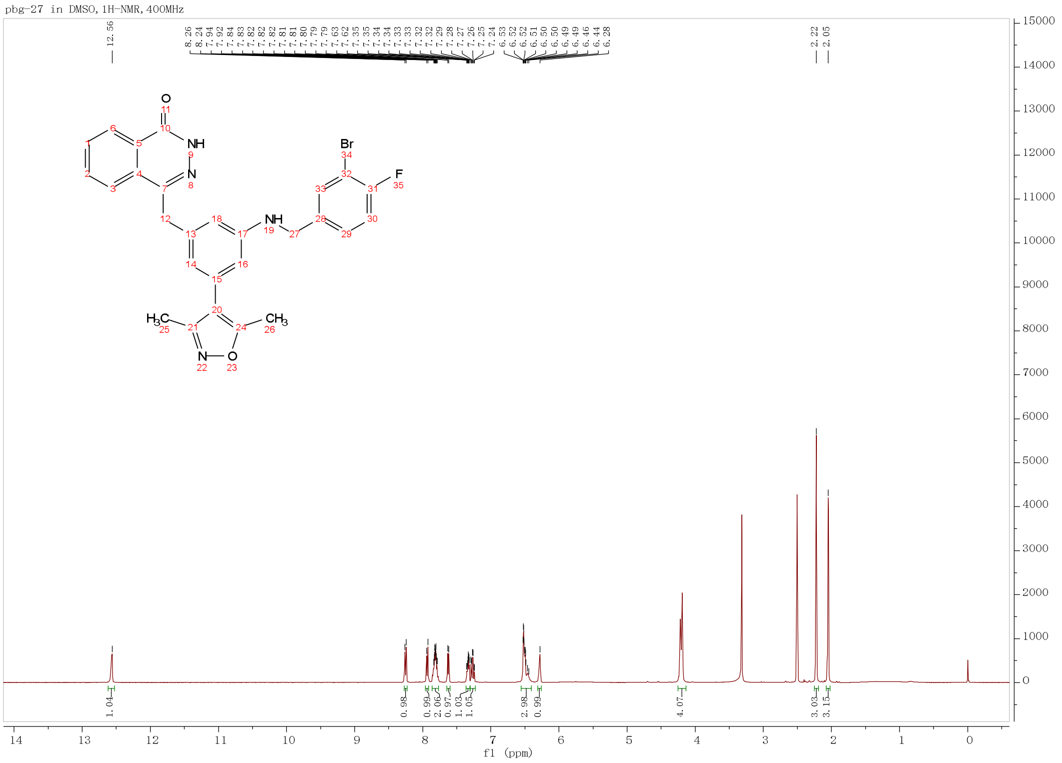

^{1}H-NMR spectrum of compound DDT43

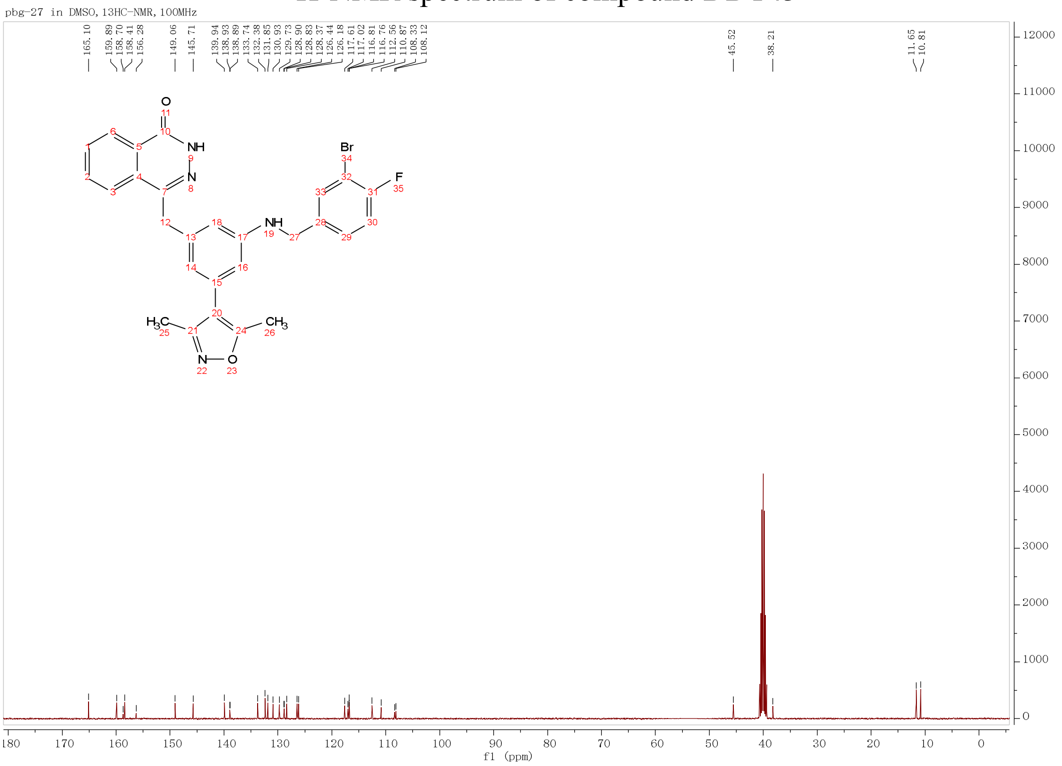

^{13}C-NMR spectrum of compound DDT43

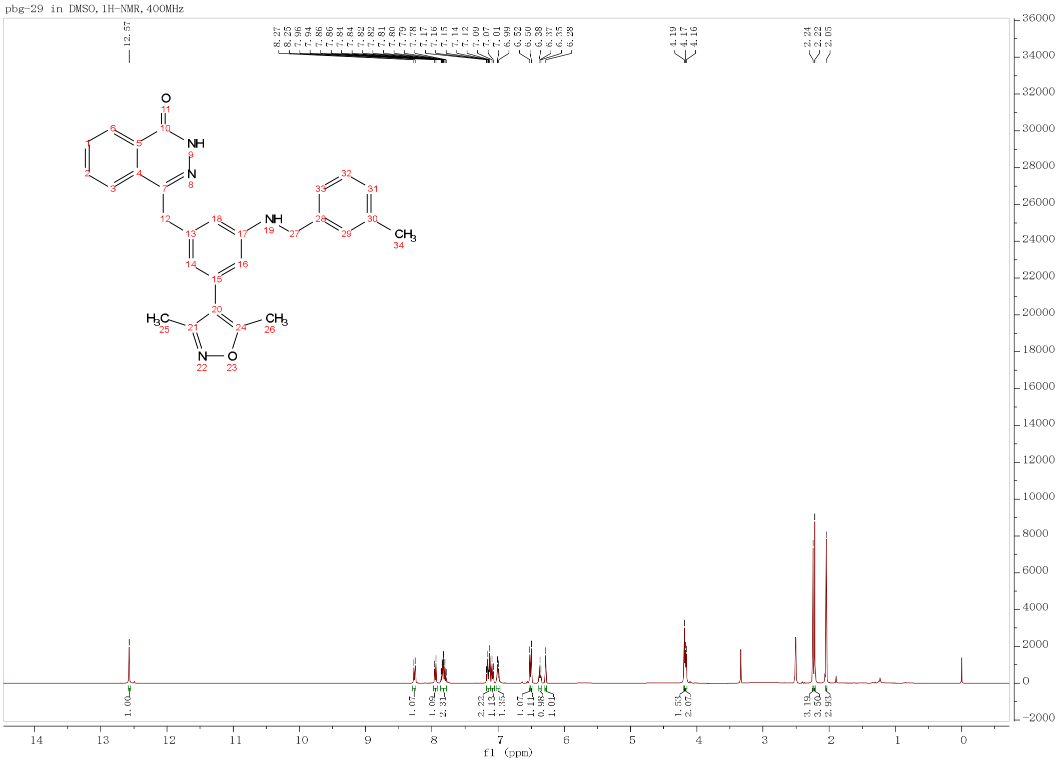

^{1}H-NMR spectrum of compound DDT44

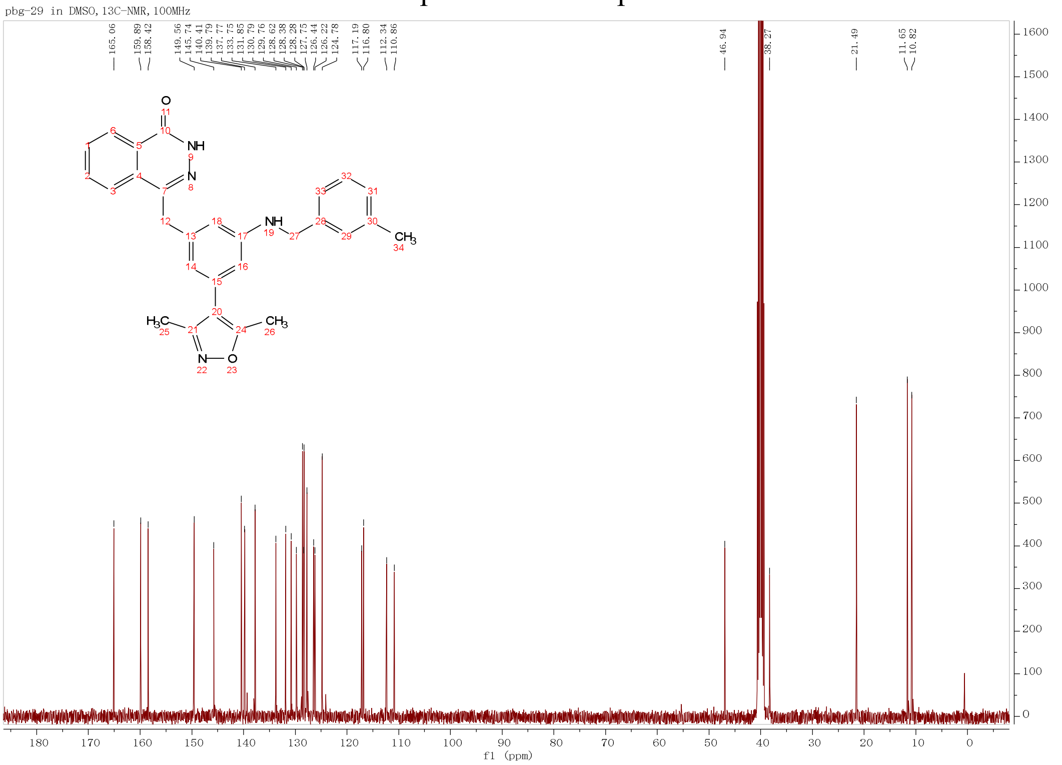

^{13}C-NMR spectrum of compound DDT44

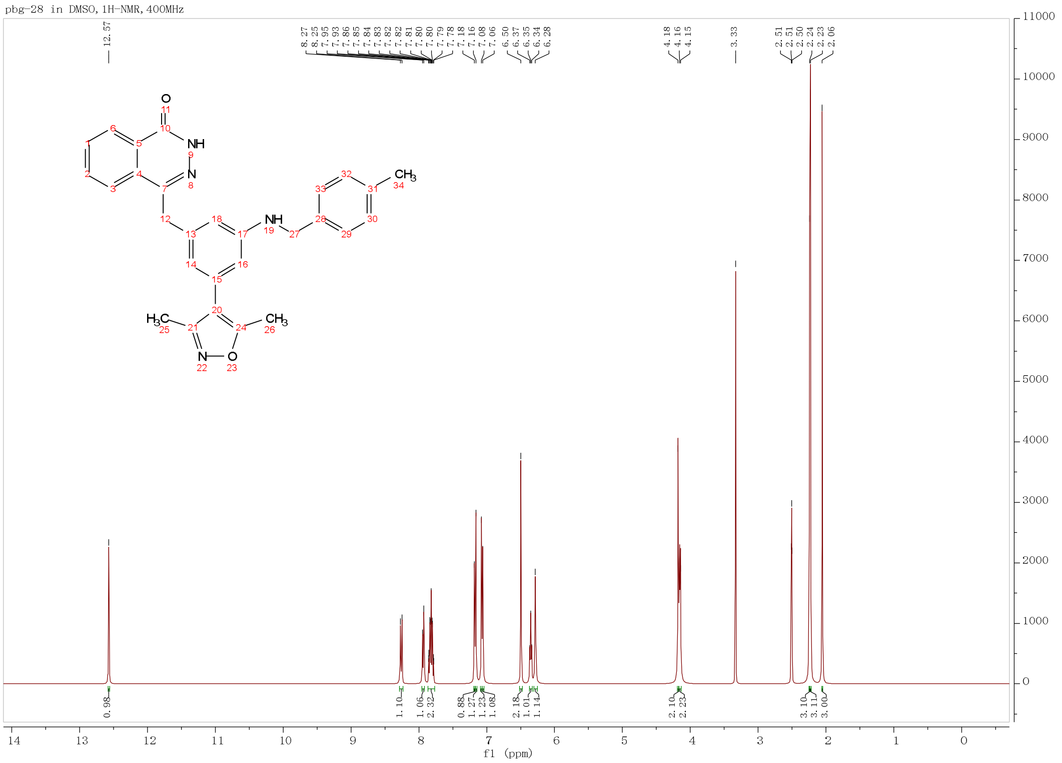

^{1}H-NMR spectrum of compound DDT45

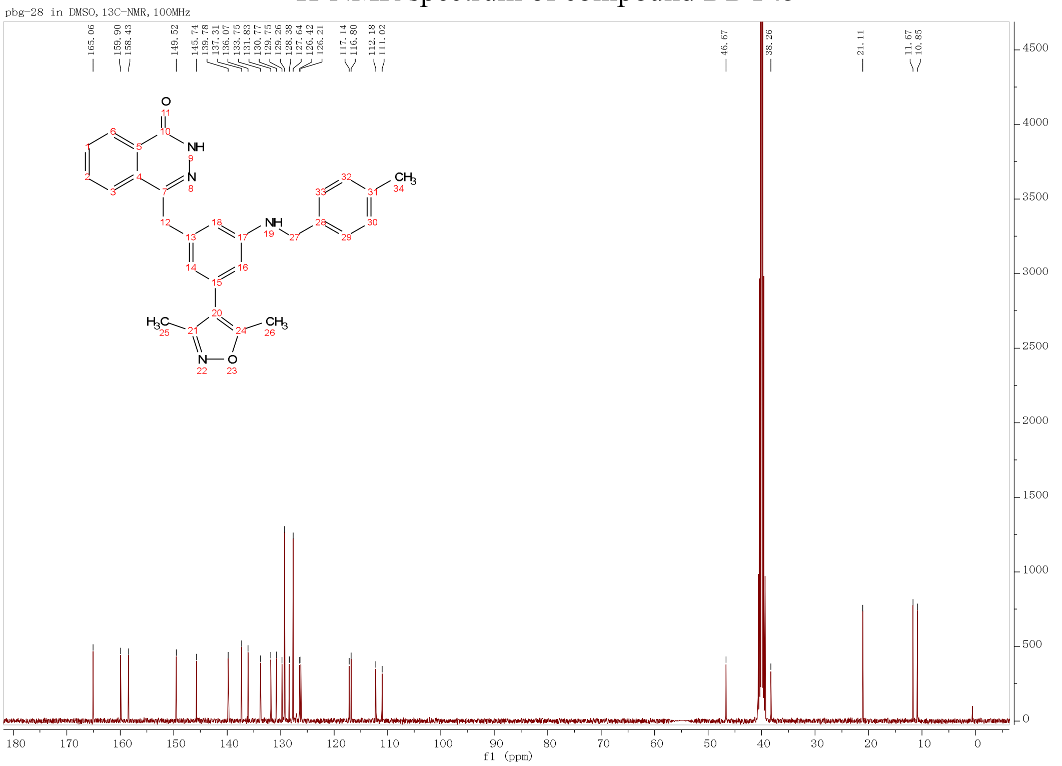

^{13}C-NMR spectrum of compound DDT45

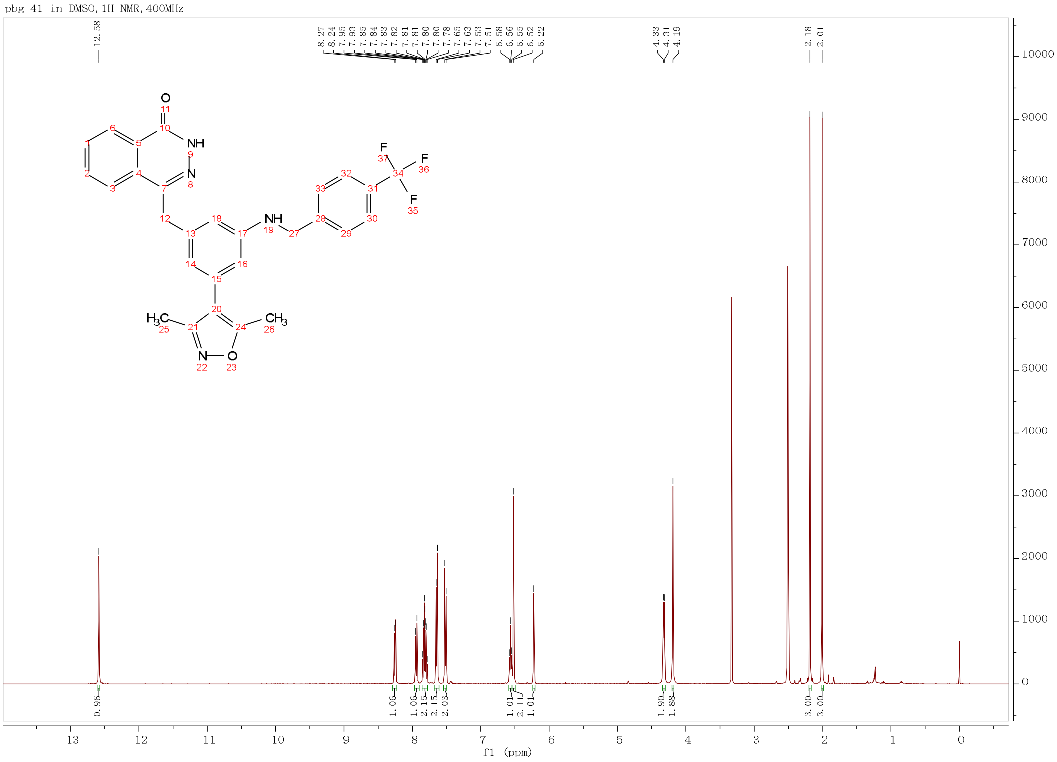

^{1}H-NMR spectrum of compound DDT46

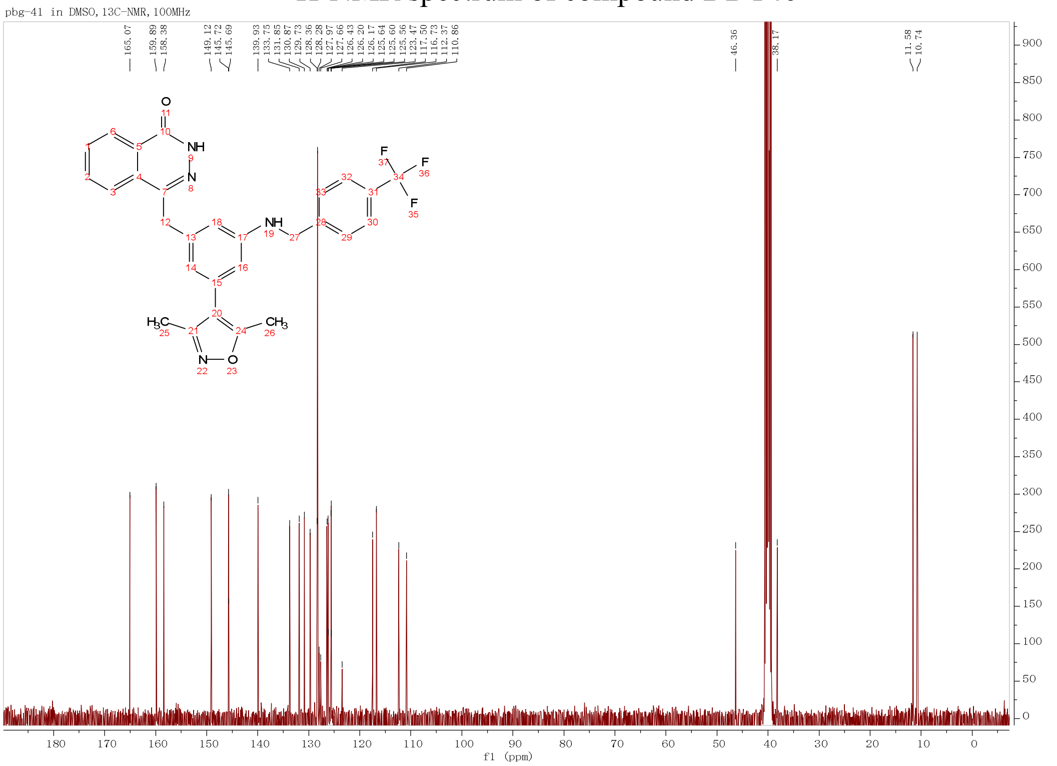

^{13}C-NMR spectrum of compound DDT46

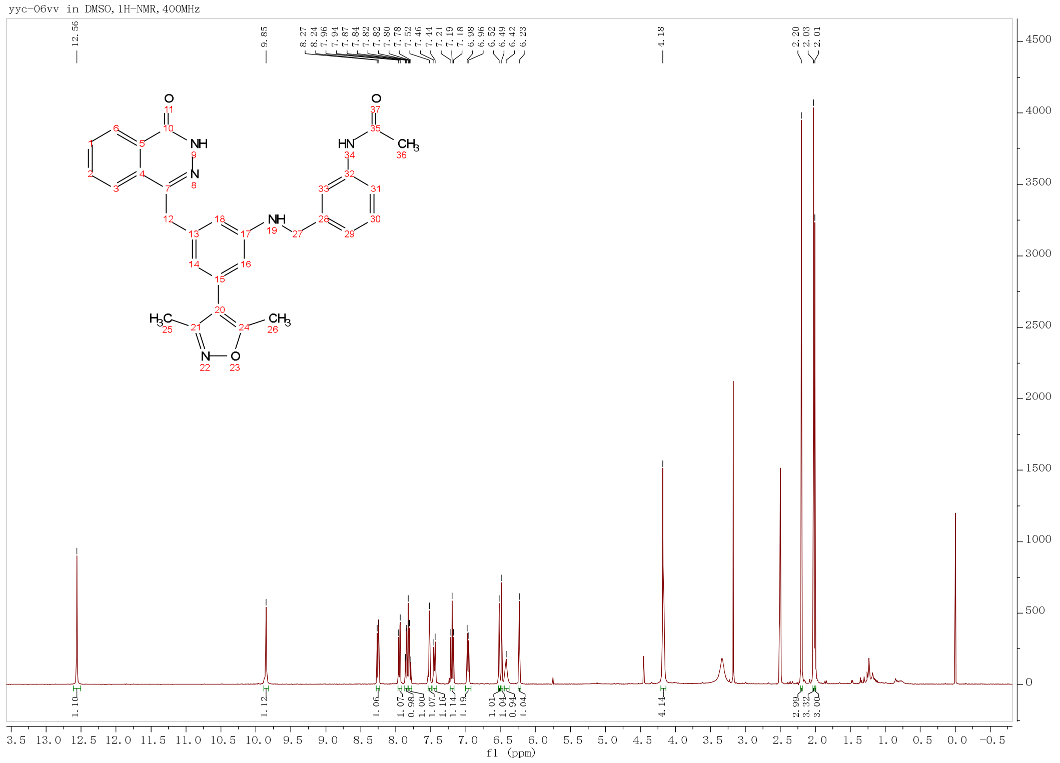

^{1}H-NMR spectrum of compound DDT47

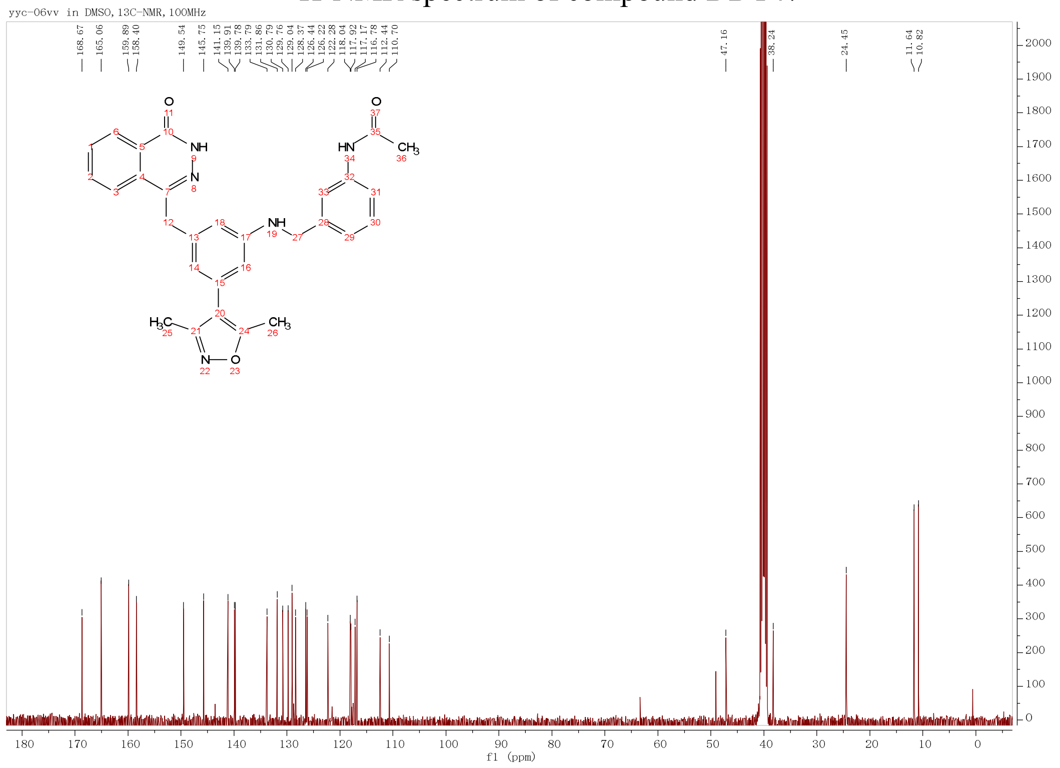

^{13}C-NMR spectrum of compound DDT47

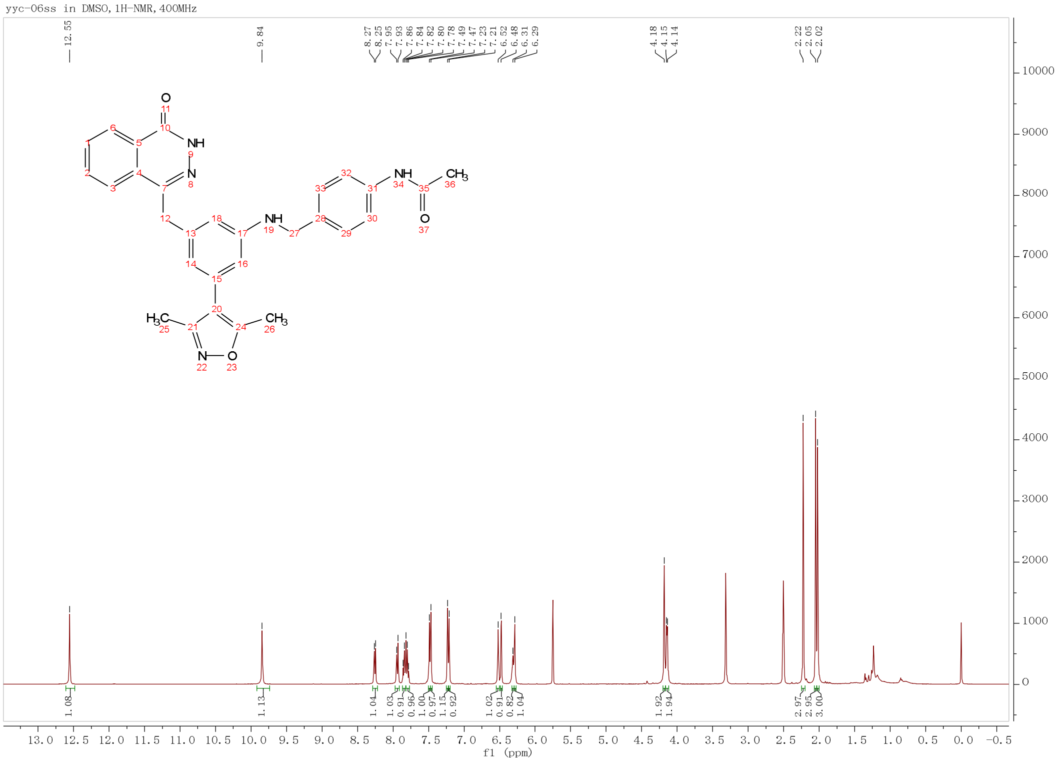

^{1}H-NMR spectrum of compound DDT48

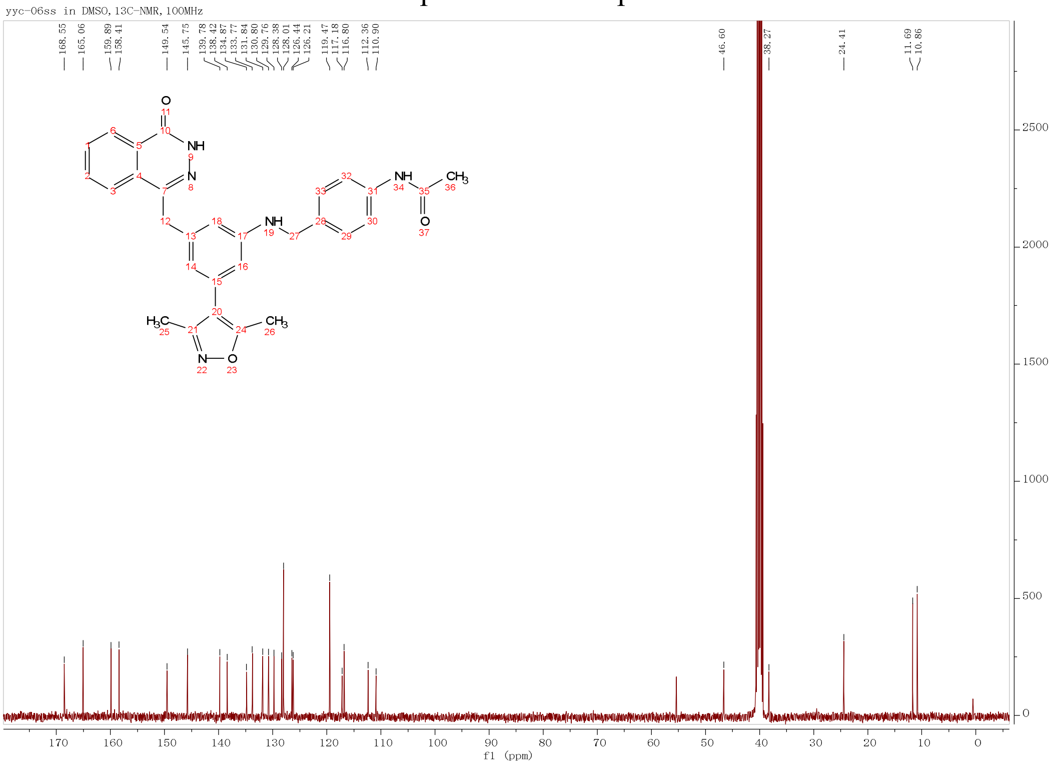

^{13}C-NMR spectrum of compound DDT48

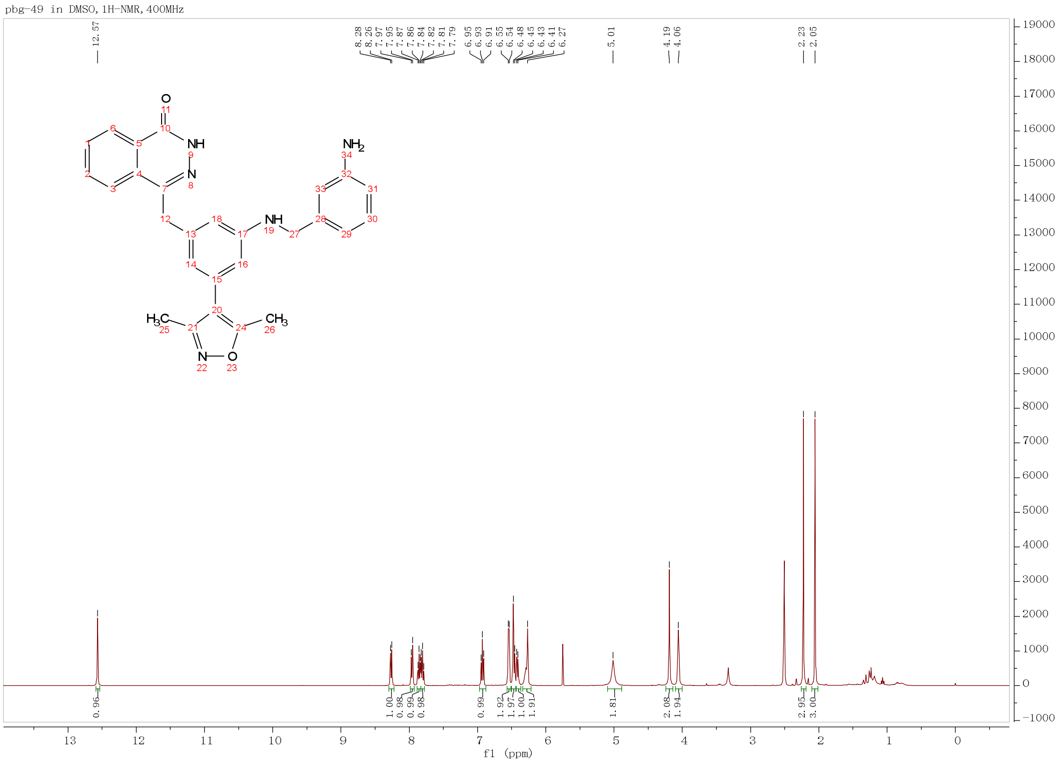

^{1}H-NMR spectrum of compound DDT49

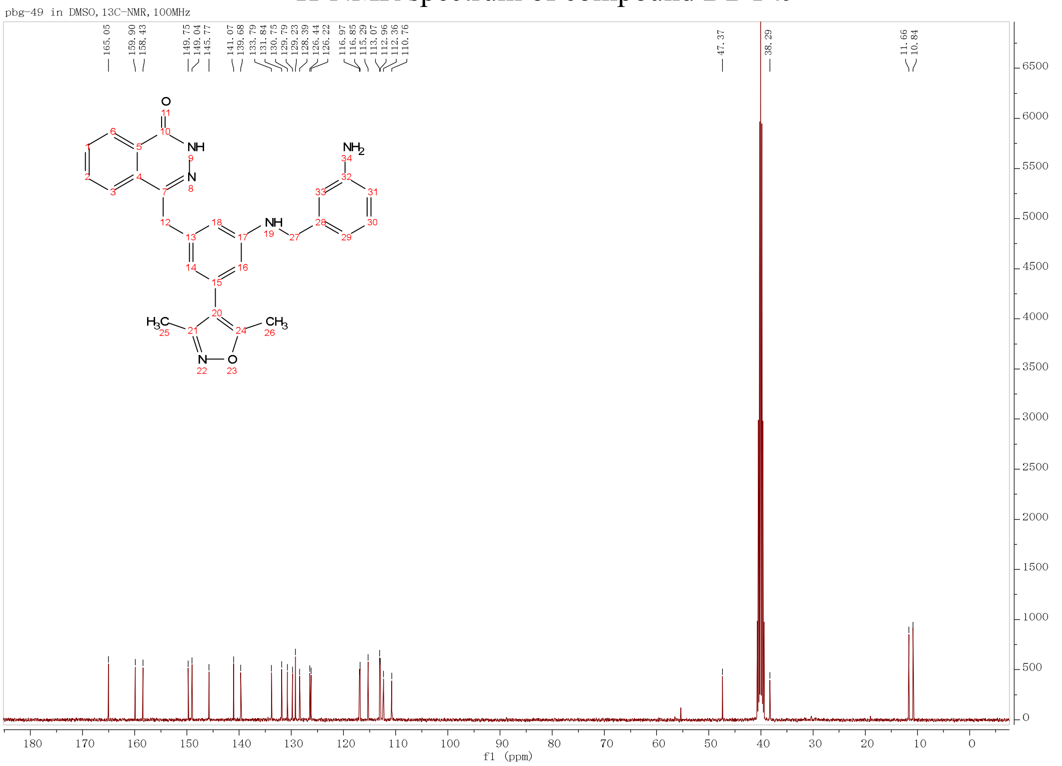

^{13}C-NMR spectrum of compound DDT49

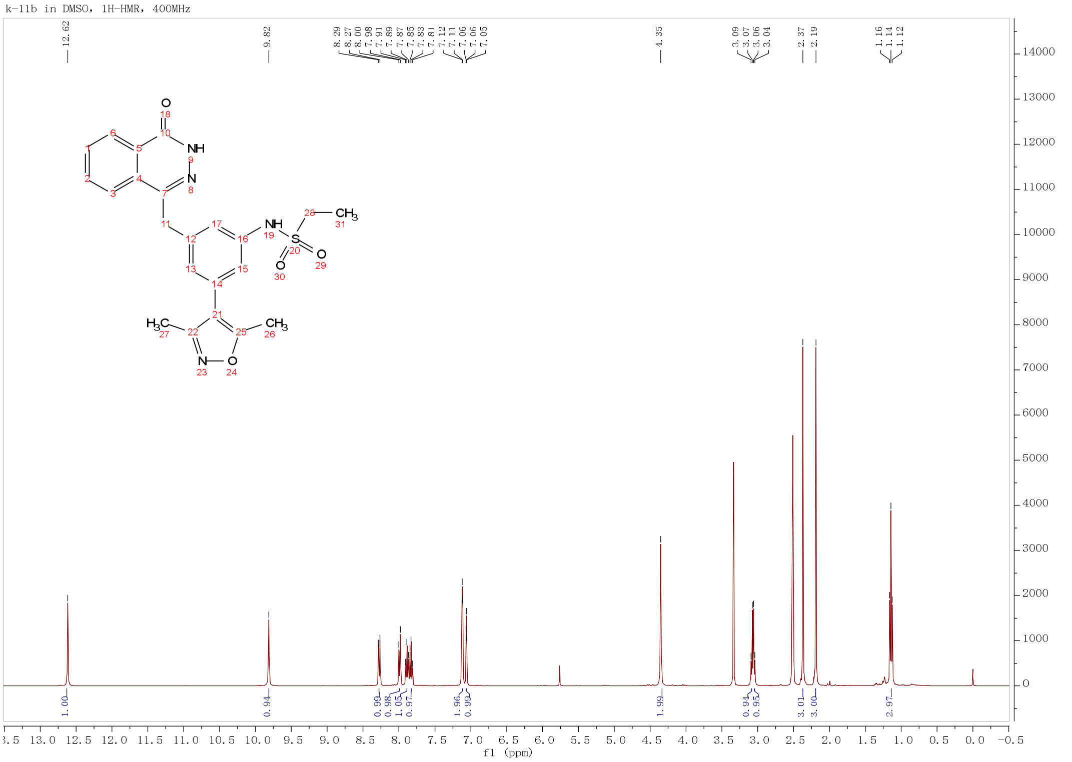

^{1}H-NMR spectrum of compound DDT50

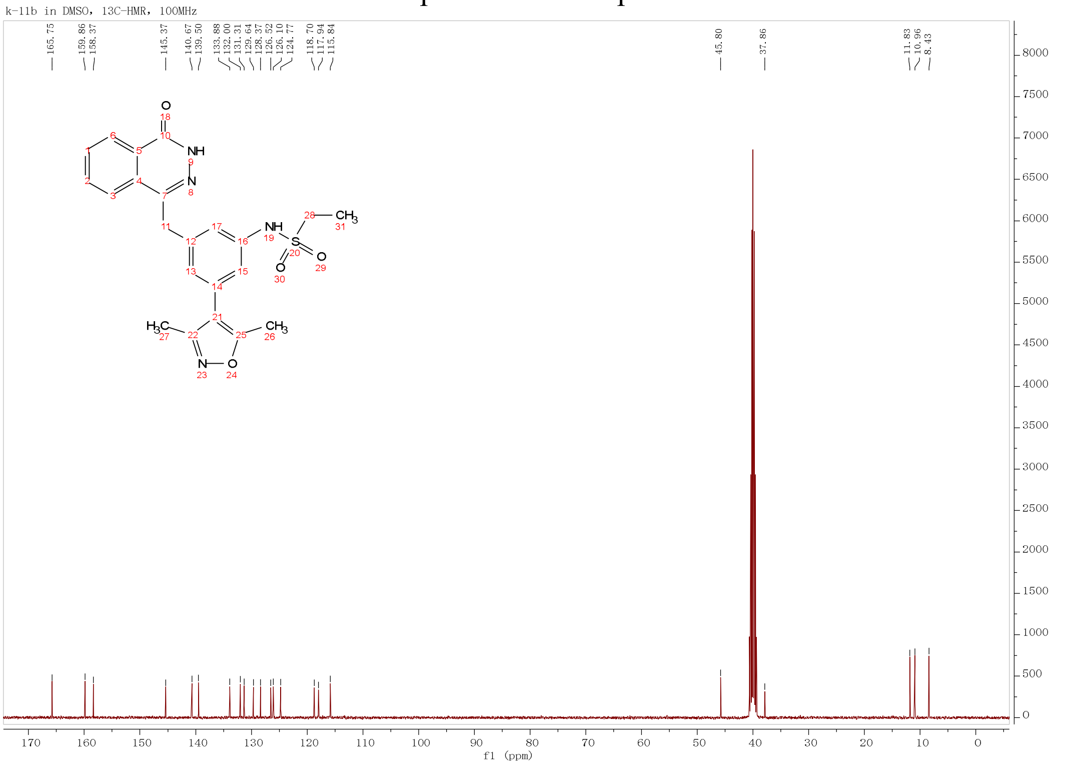

^{13}C-NMR spectrum of compound DDT50

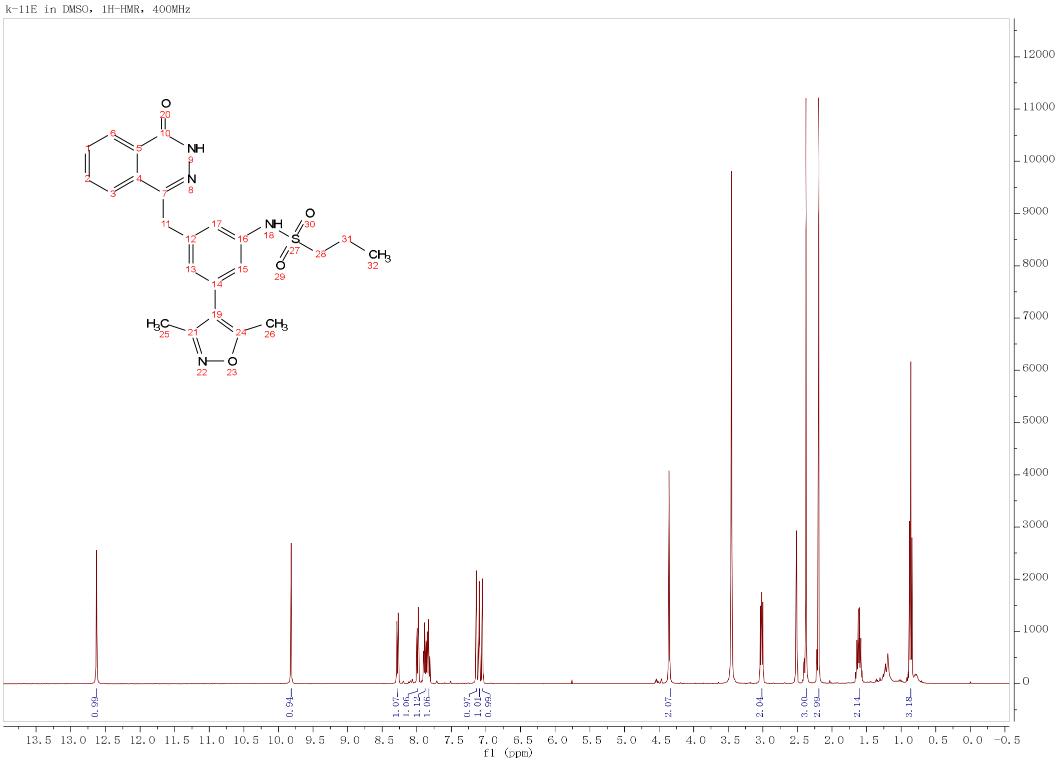

^{1}H-NMR spectrum of compound DDT51

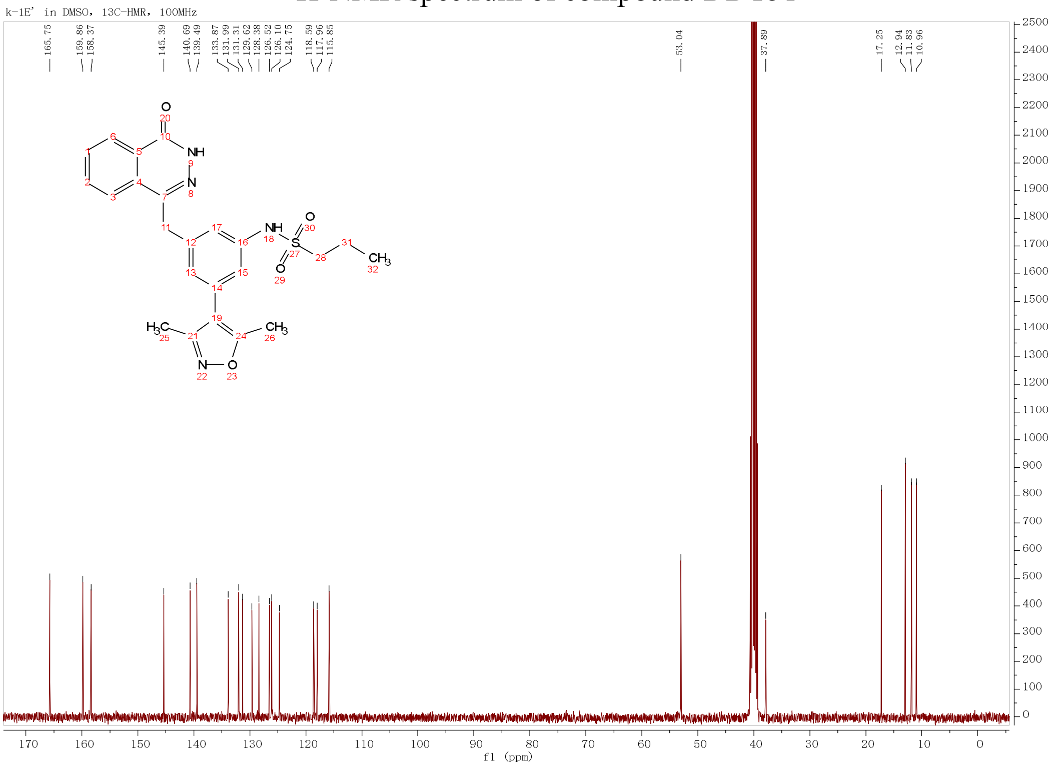

^{13}C-NMR spectrum of compound DDT51

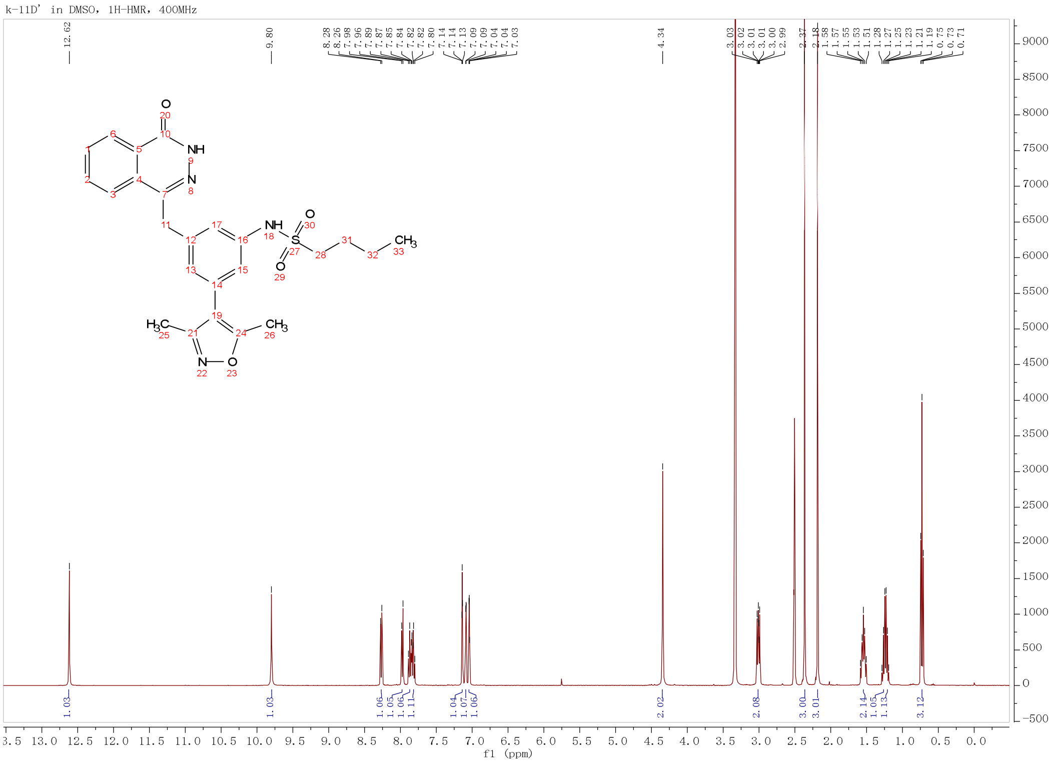

^{1}H-NMR spectrum of compound DDT52

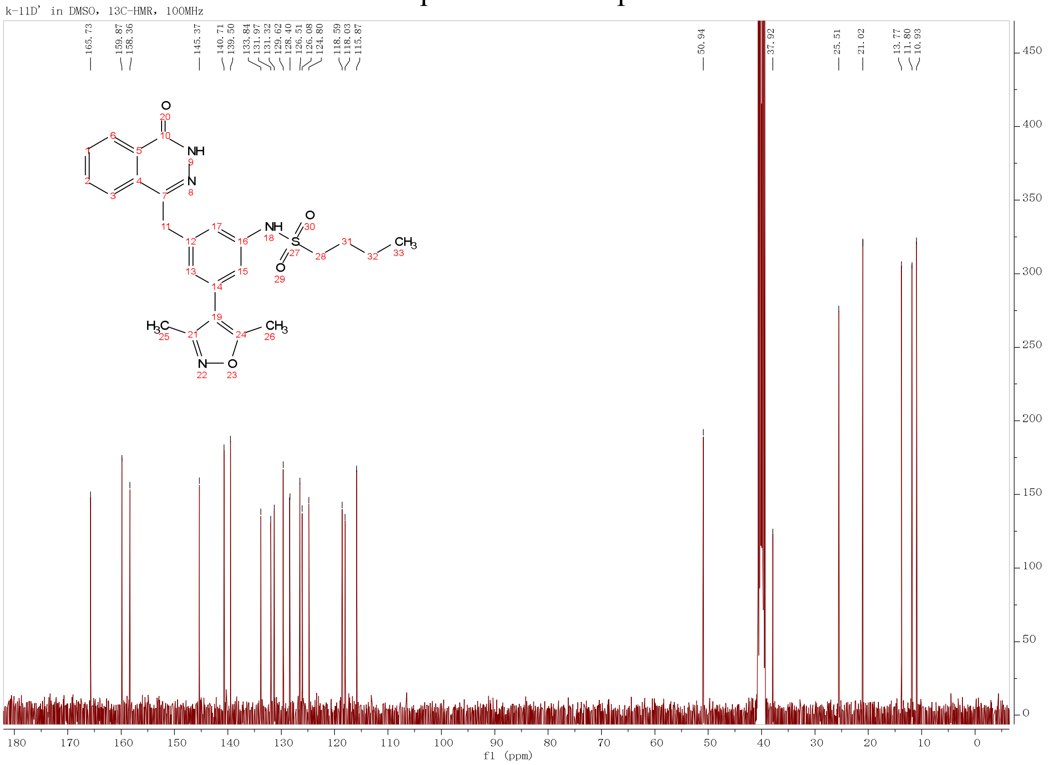

^{13}C-NMR spectrum of compound DDT52

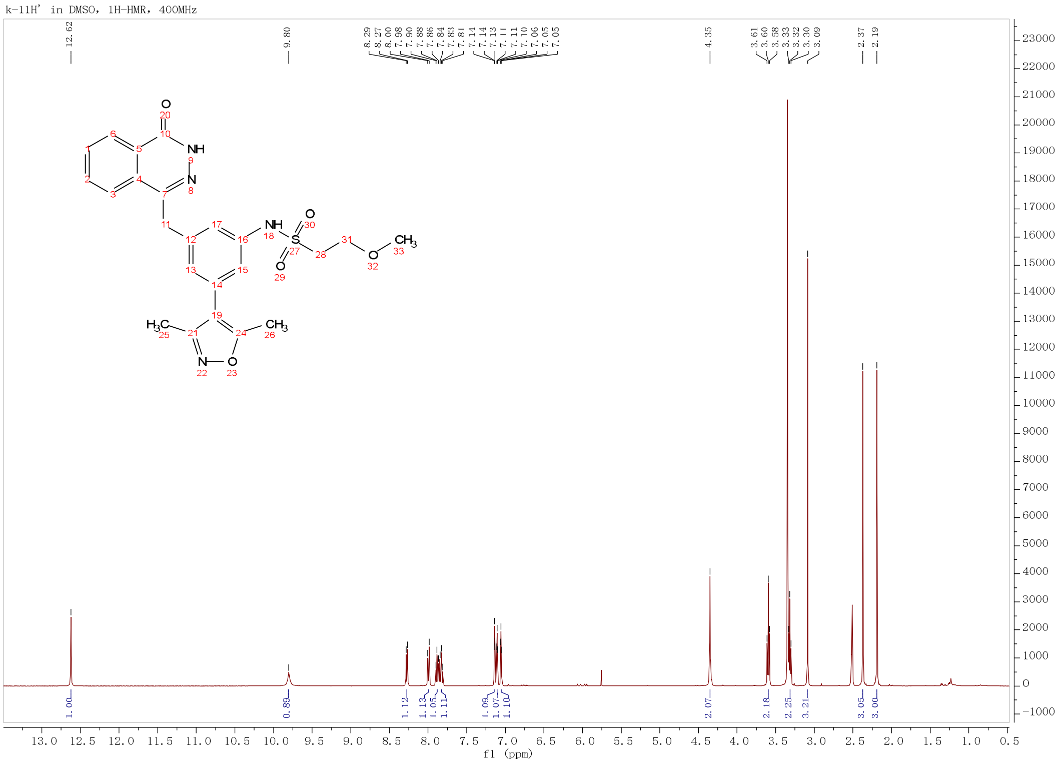

^{1}H-NMR spectrum of compound DDT53

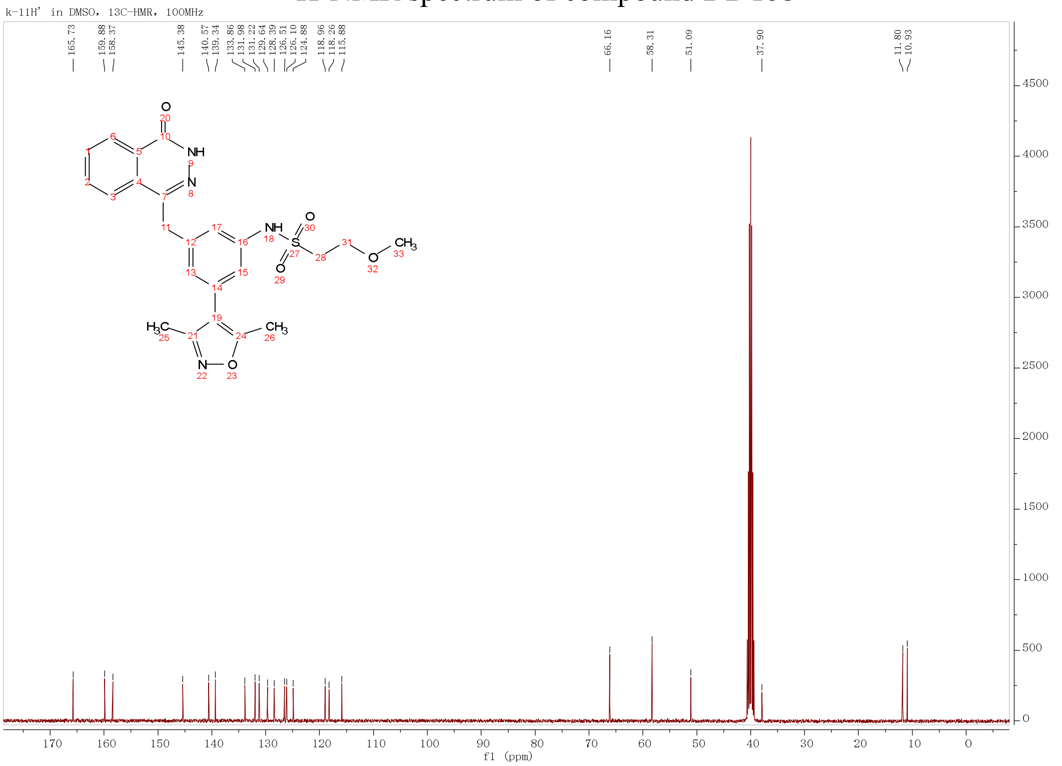

^{13}C-NMR spectrum of compound DDT53

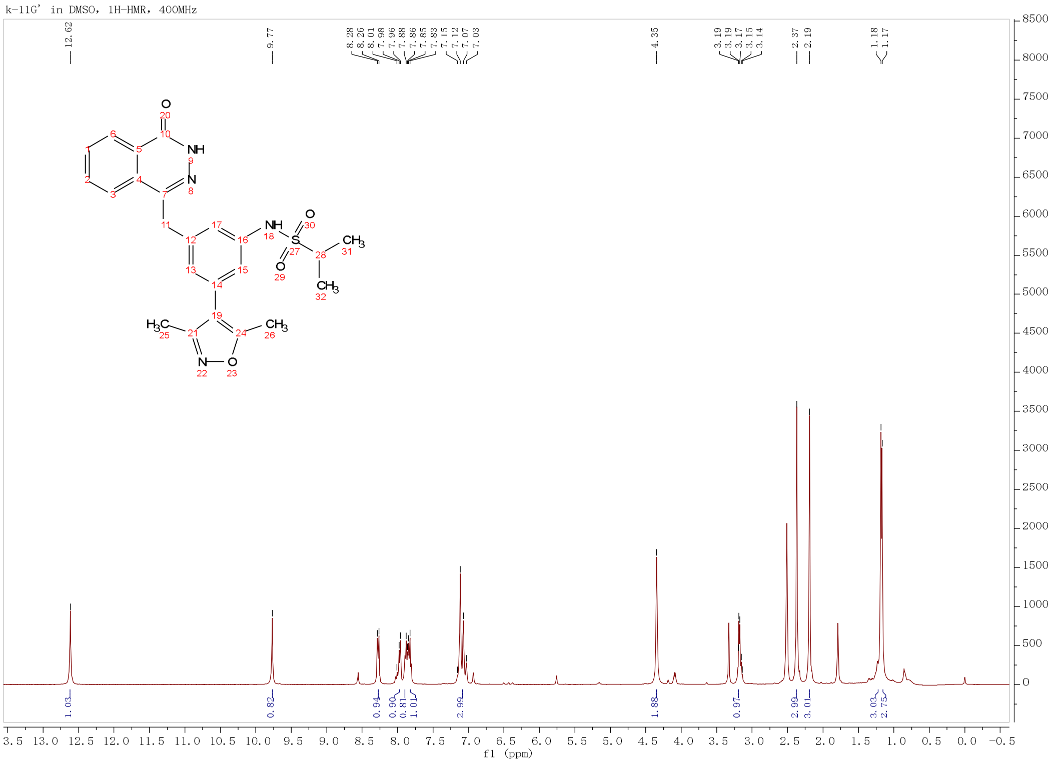

^{1}H-NMR spectrum of compound DDT54

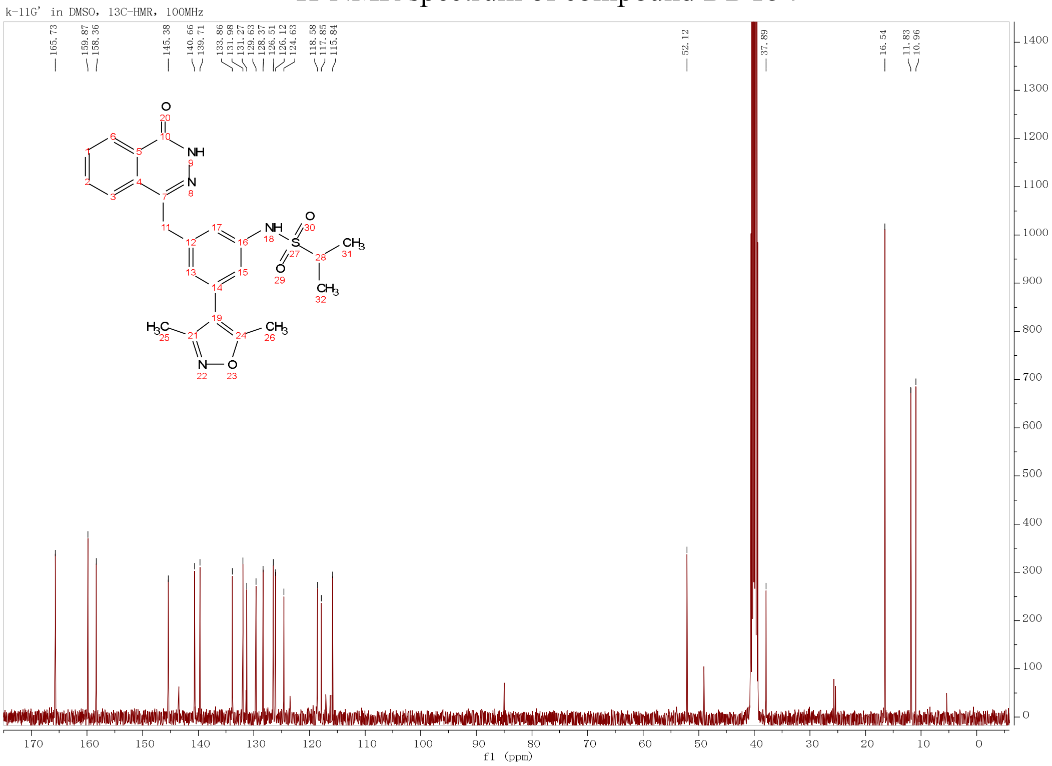

^{13}C-NMR spectrum of compound DDT54

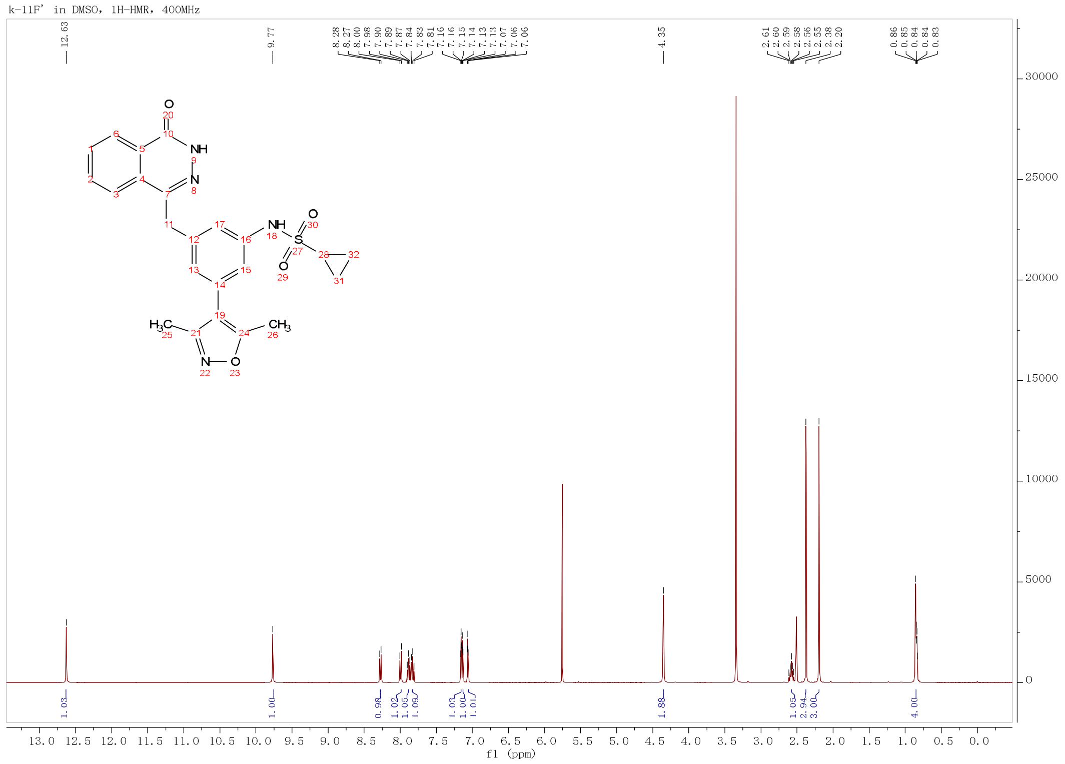

^{1}H-NMR spectrum of compound DDT55

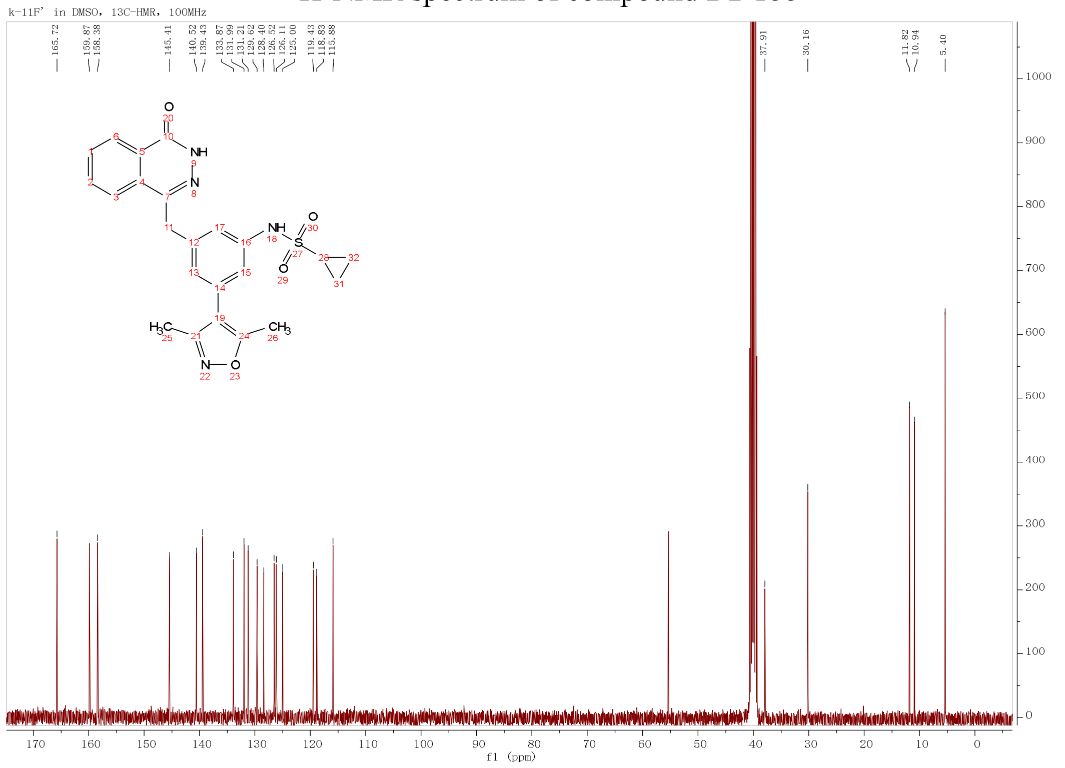

^{13}C-NMR spectrum of compound DDT55

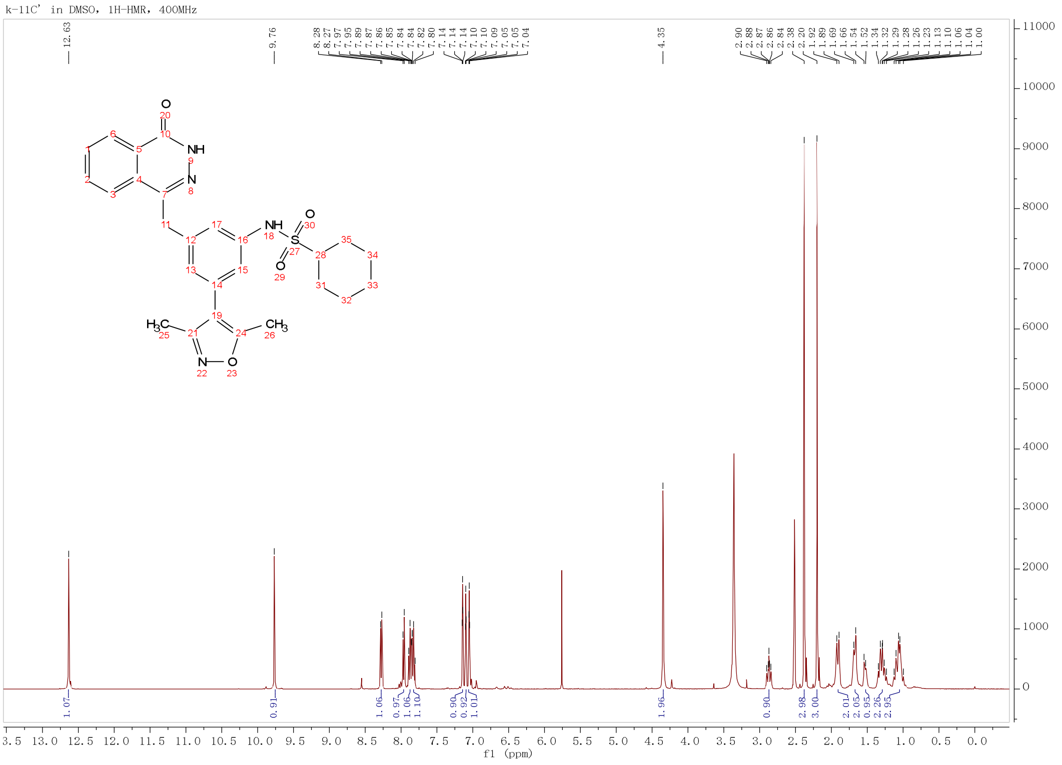

^{1}H-NMR spectrum of compound DDT56

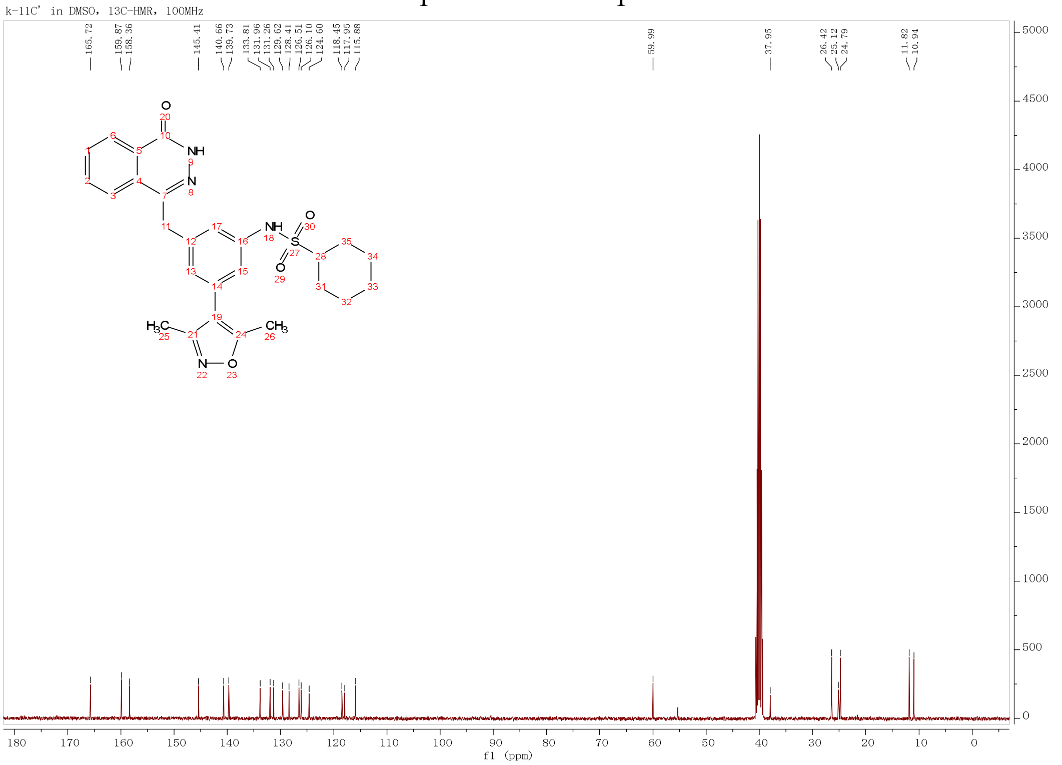

^{13}C-NMR spectrum of compound DDT56

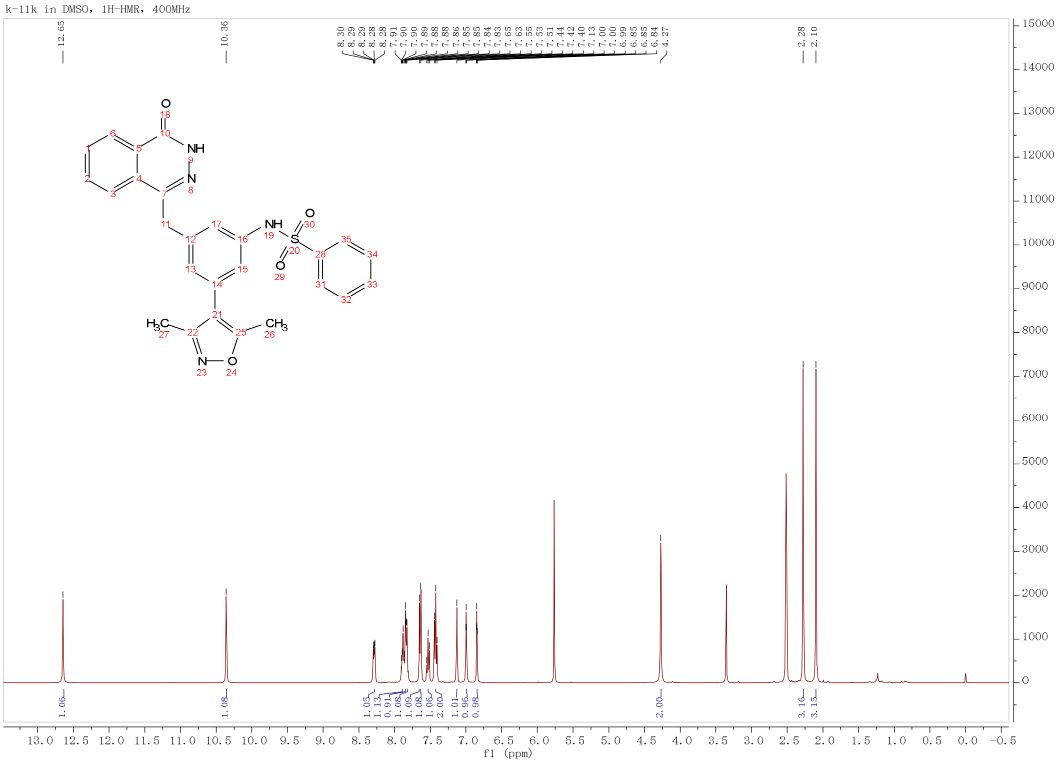

^{1}H-NMR spectrum of compound DDT57

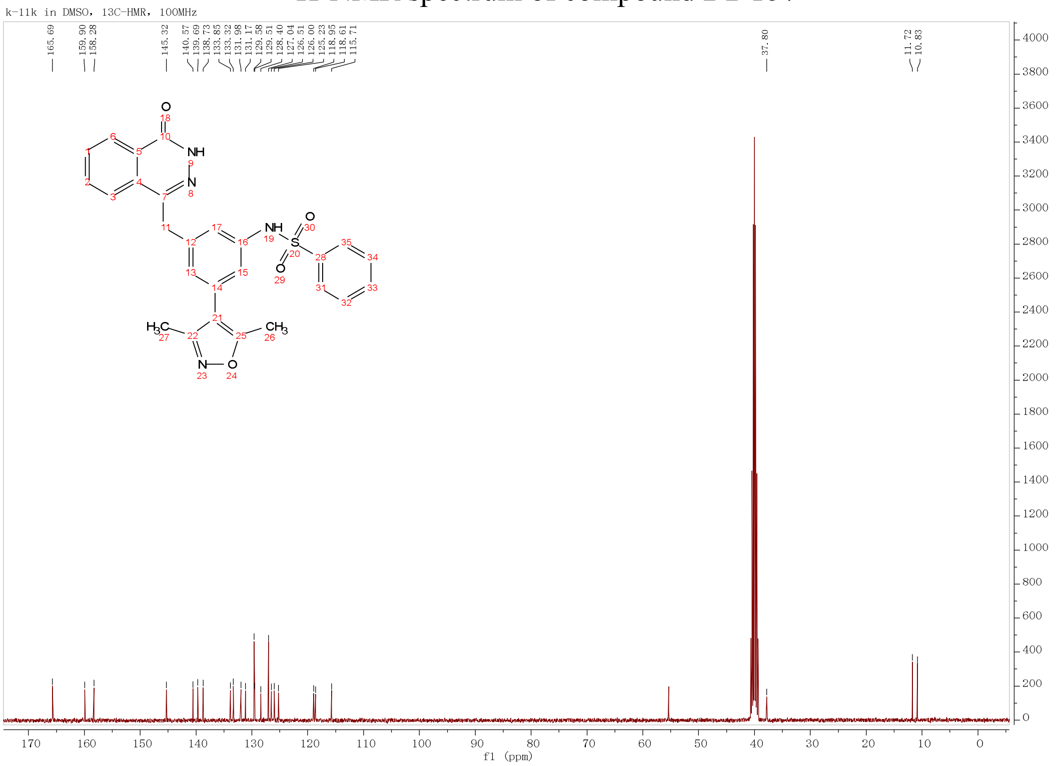

^{13}C-NMR spectrum of compound DDT57

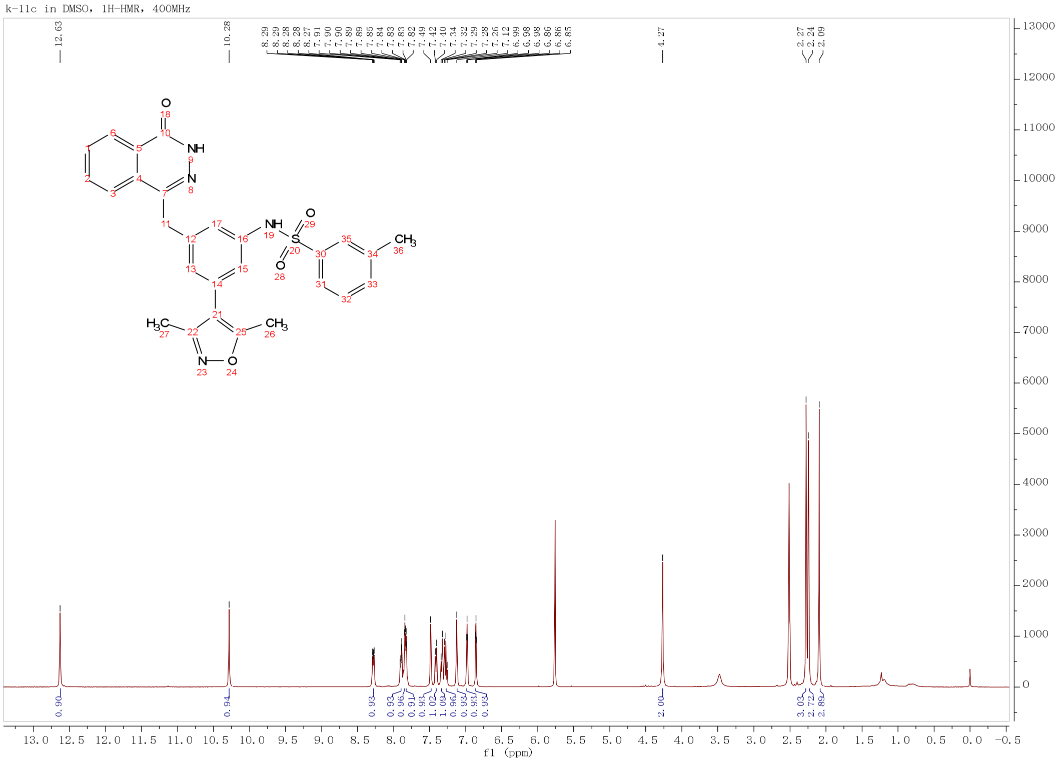

^{1}H-NMR spectrum of compound DDT58

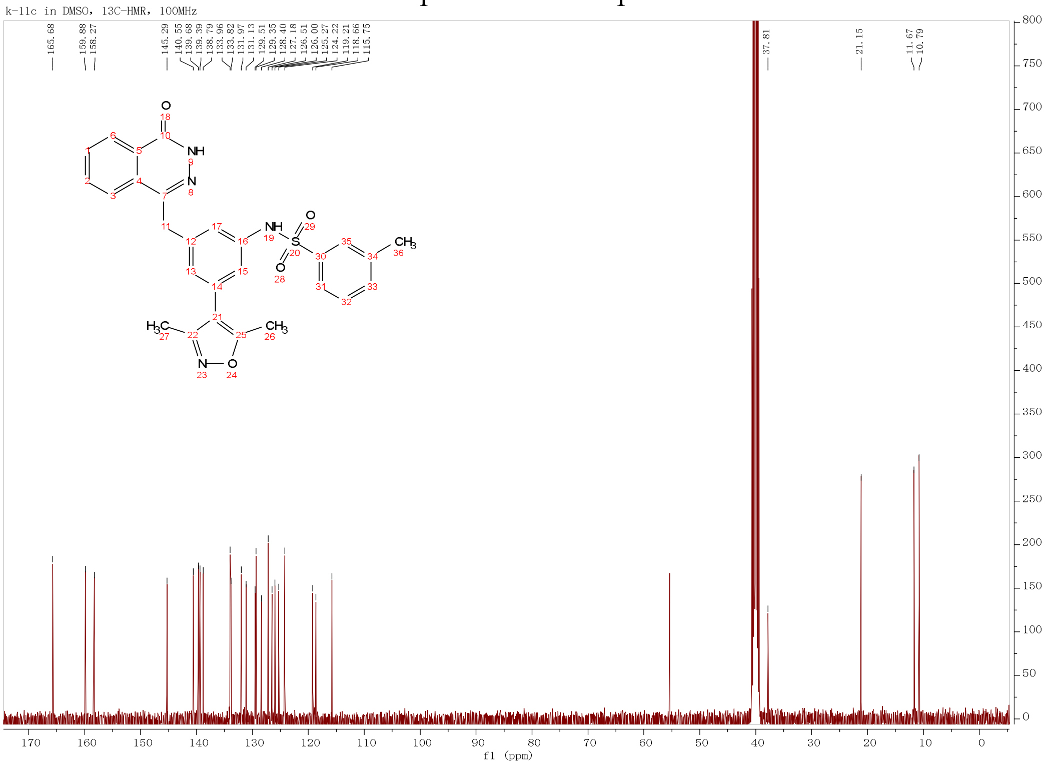

^{13}C-NMR spectrum of compound DDT58

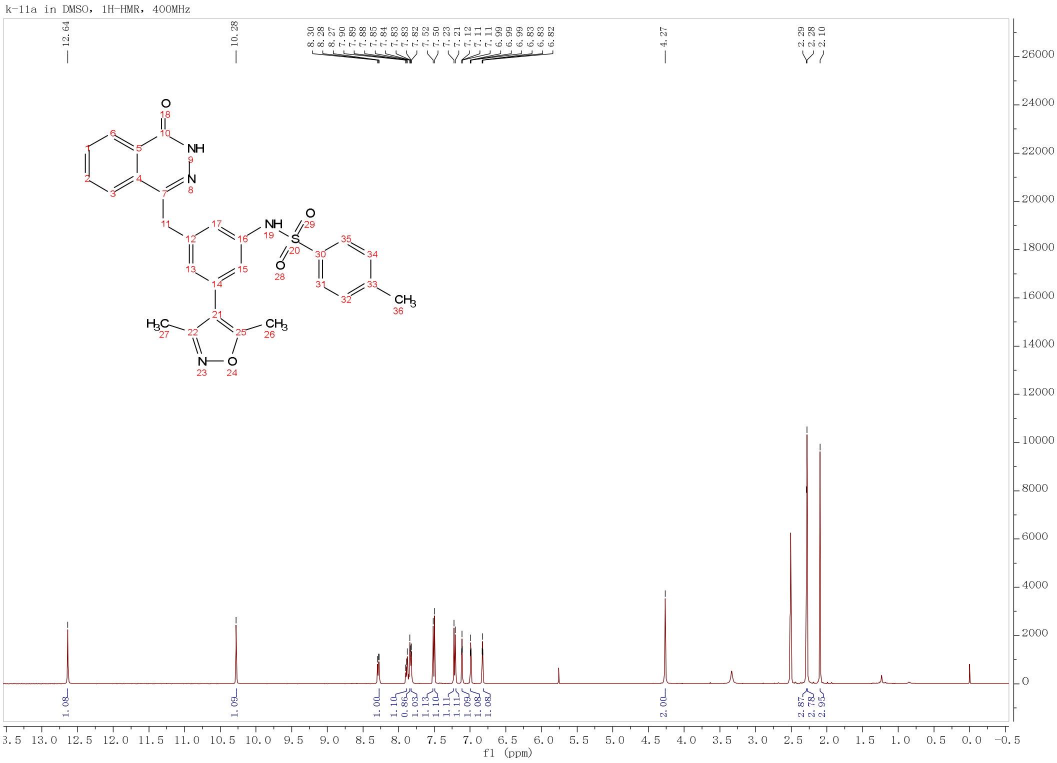

^{1}H-NMR spectrum of compound DDT59

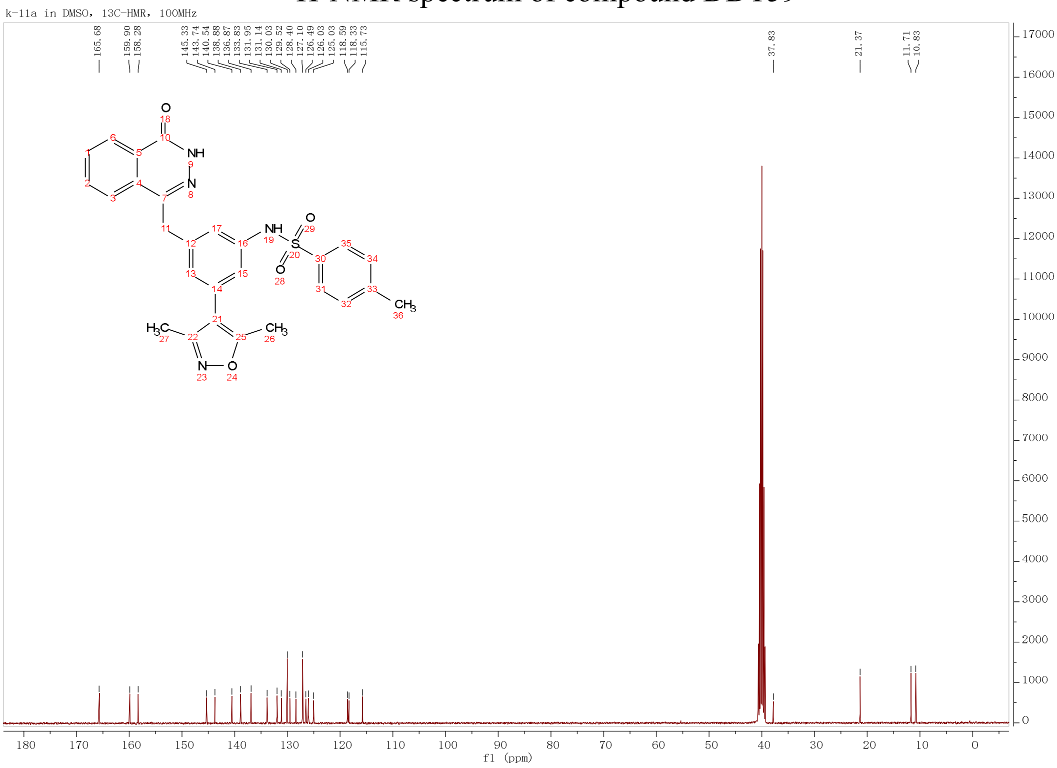

^{13}C-NMR spectrum of compound DDT59

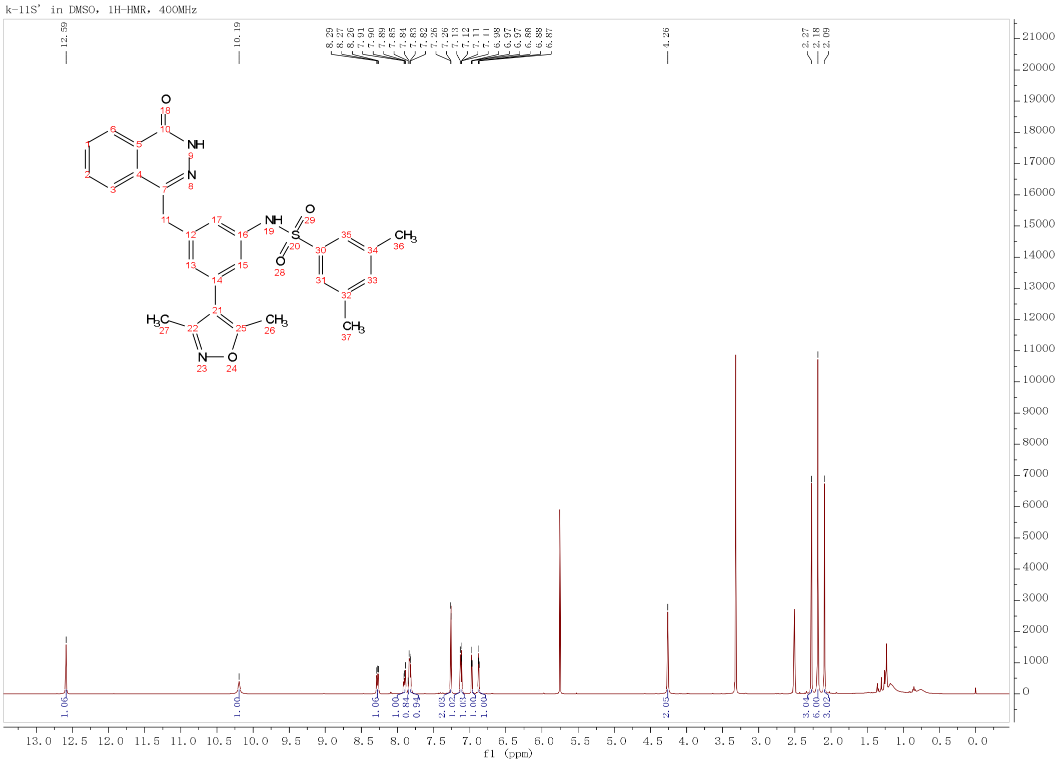

^{1}H-NMR spectrum of compound DDT60

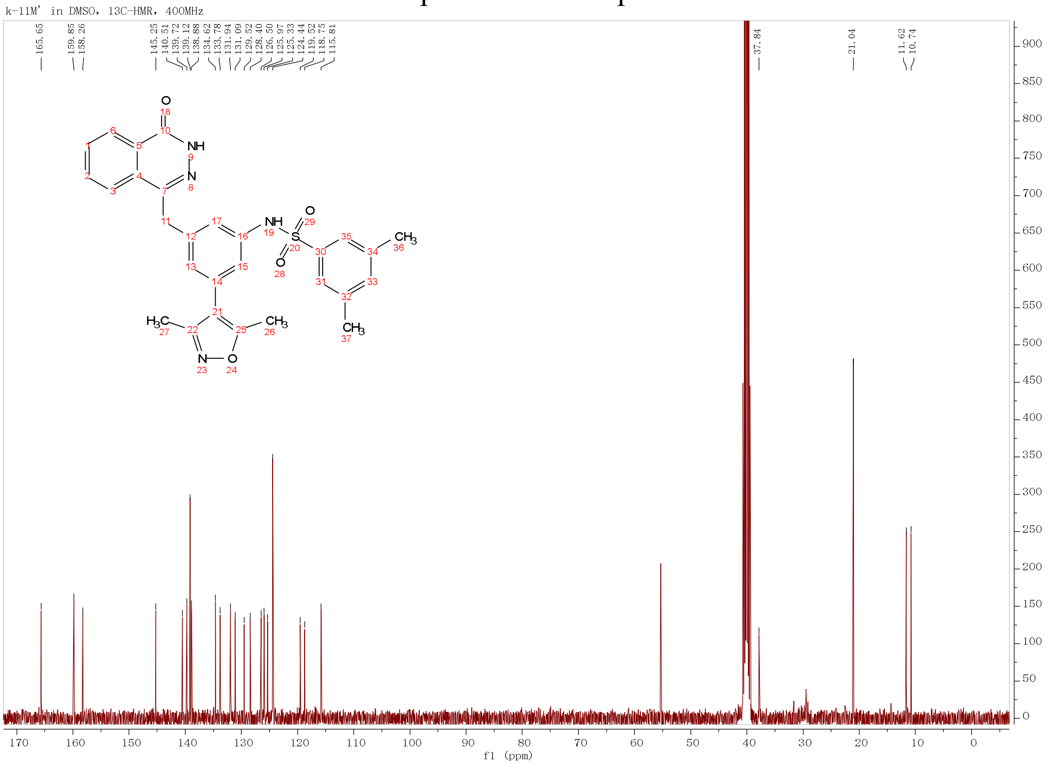

^{13}C-NMR spectrum of compound DDT60

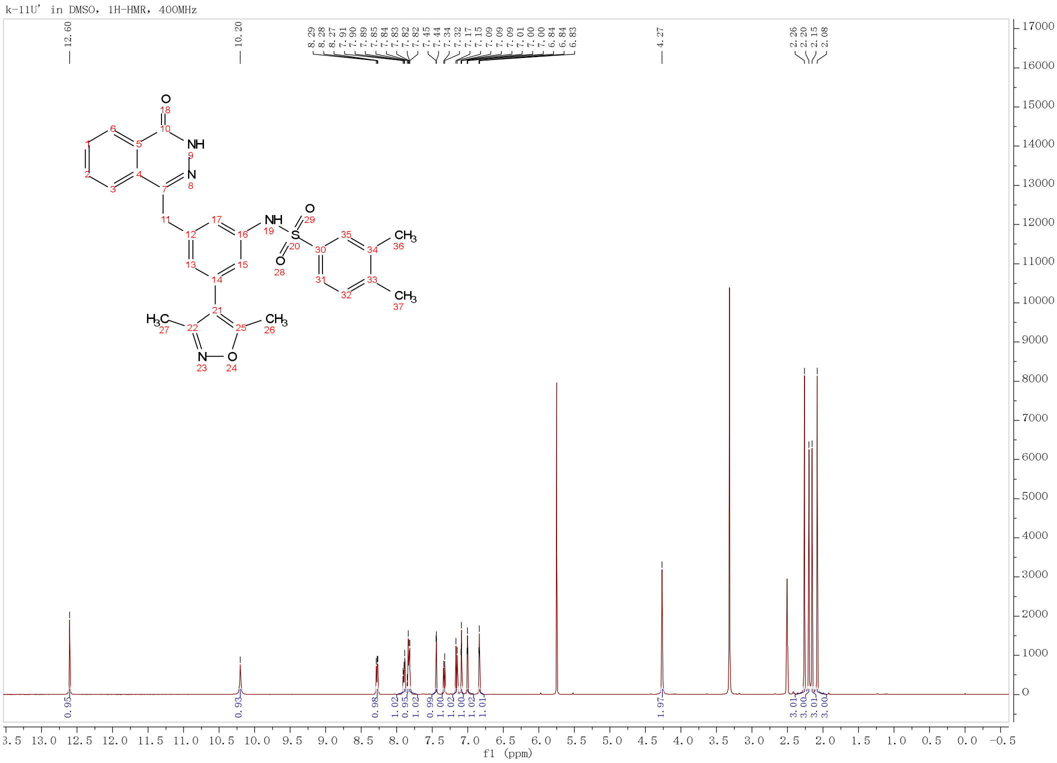

^{1}H-NMR spectrum of compound DDT61

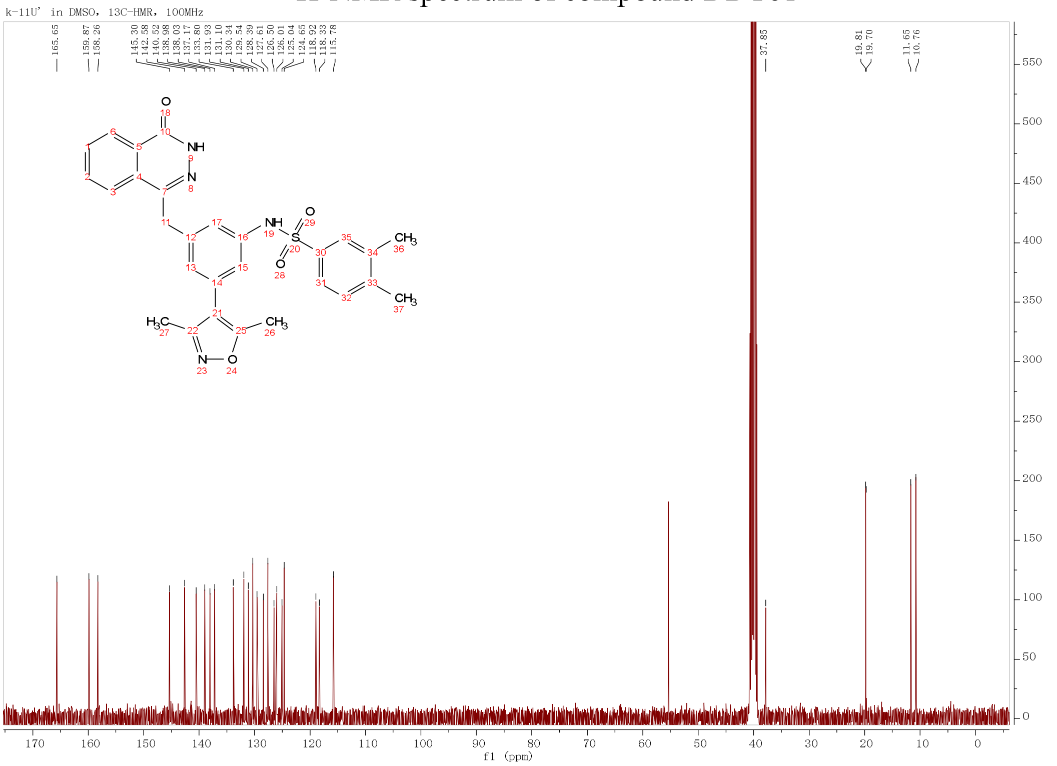

^{13}C-NMR spectrum of compound DDT61

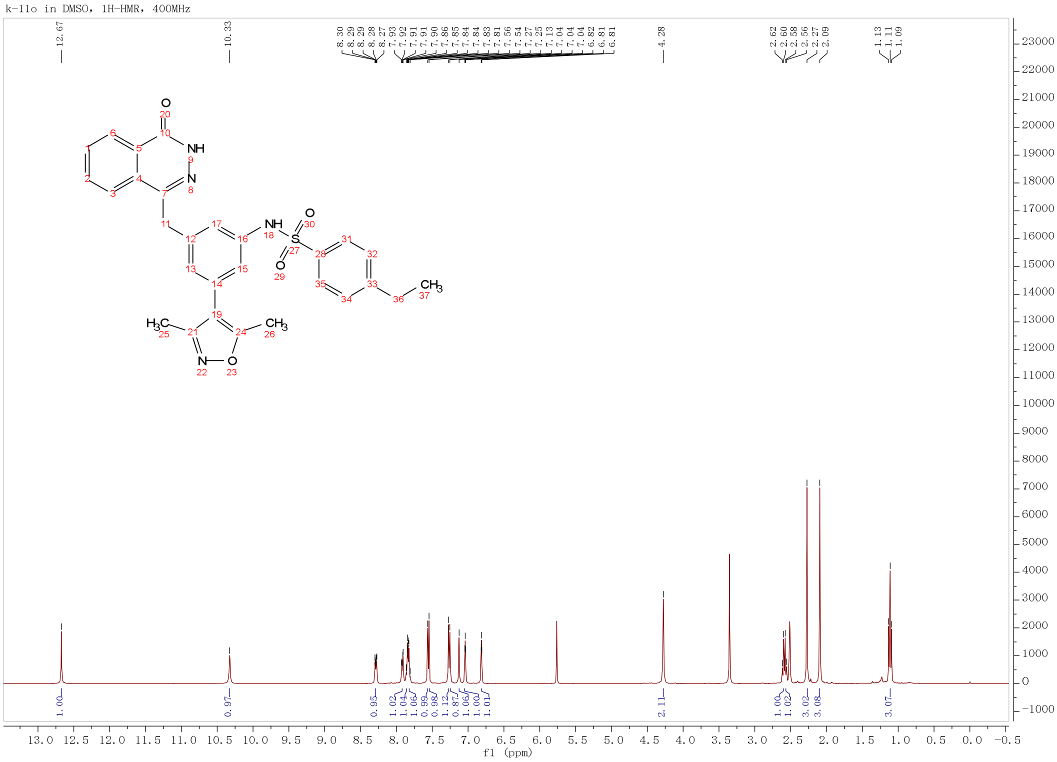

^{1}H-NMR spectrum of compound DDT62

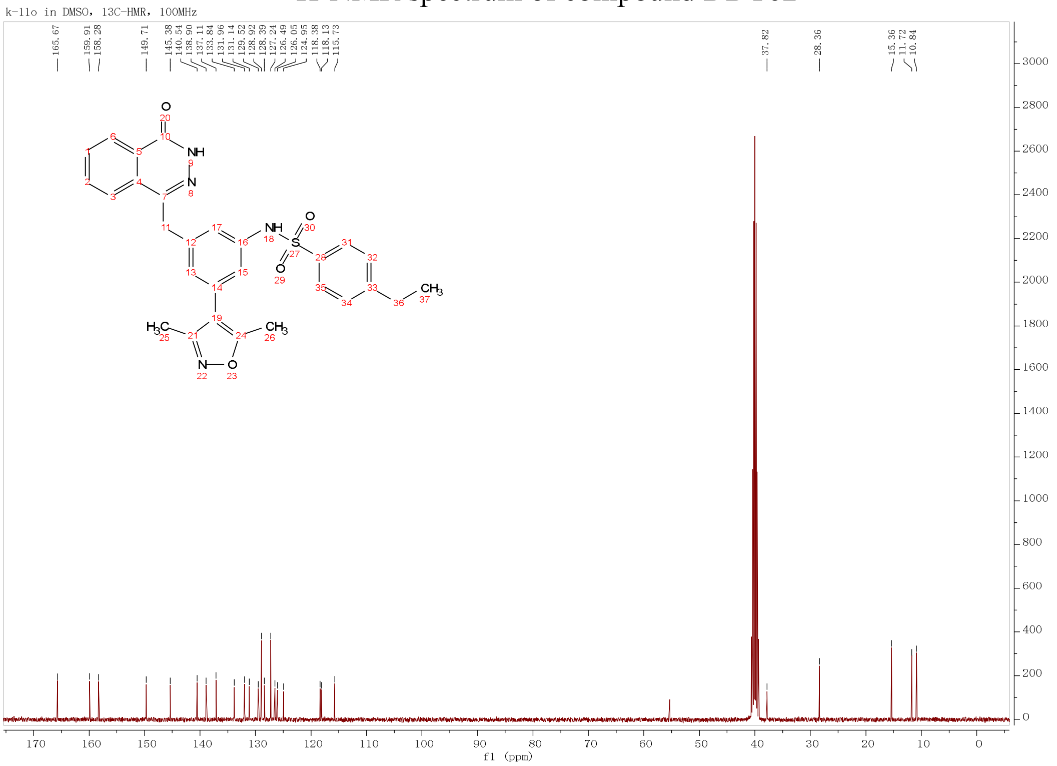

^{13}C-NMR spectrum of compound DDT62

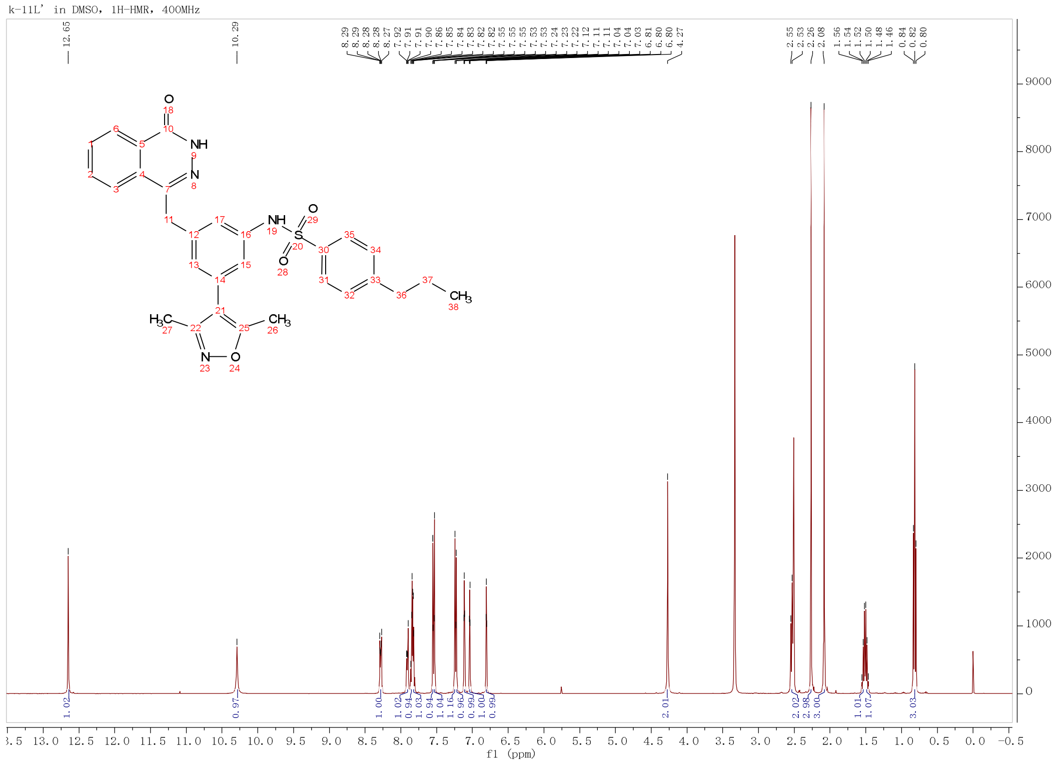

^{1}H-NMR spectrum of compound DDT63

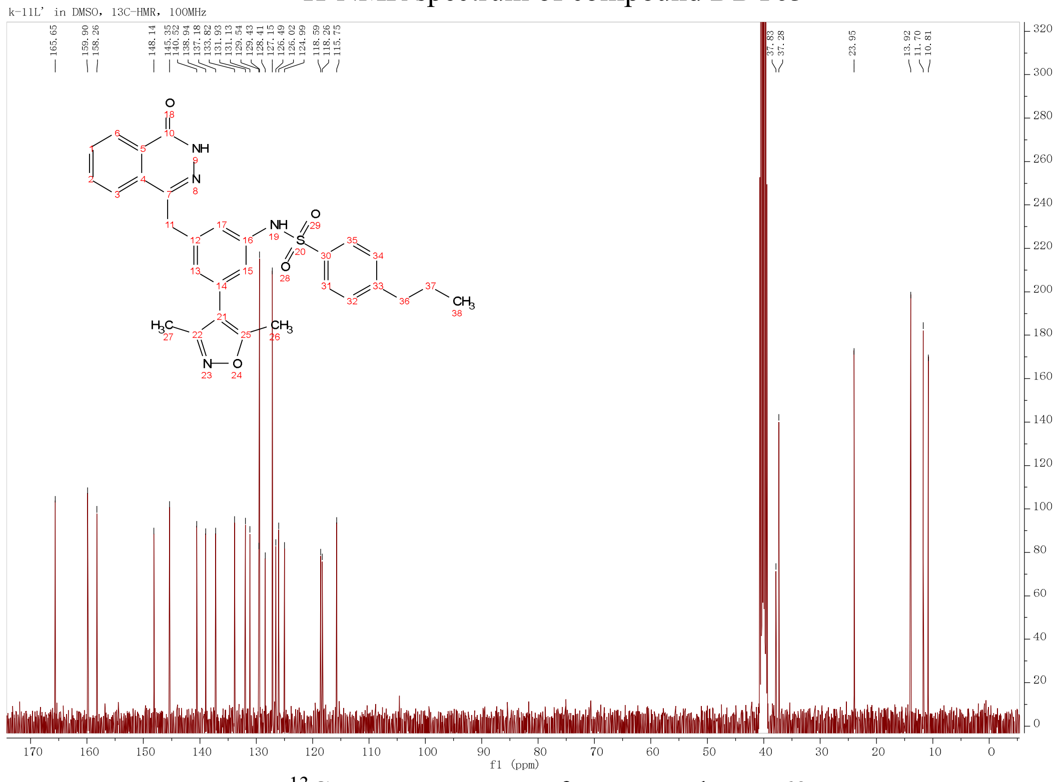

^{13}C-NMR spectrum of compound DDT63

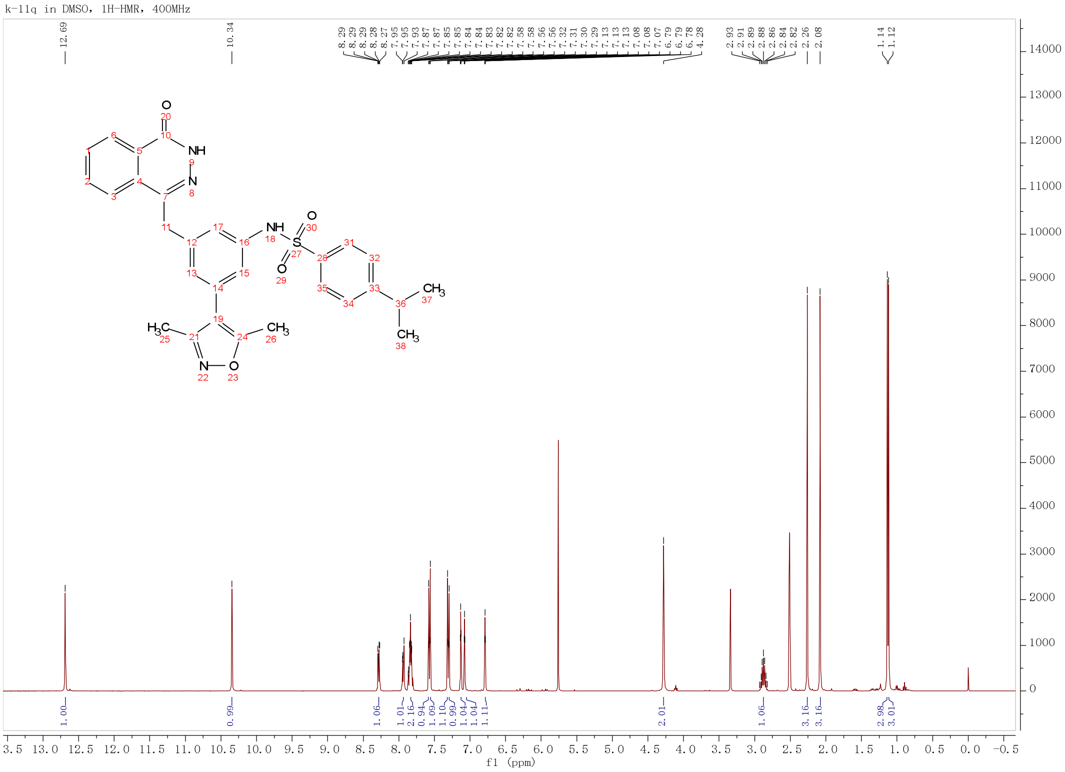

^{1}H-NMR spectrum of compound DDT64

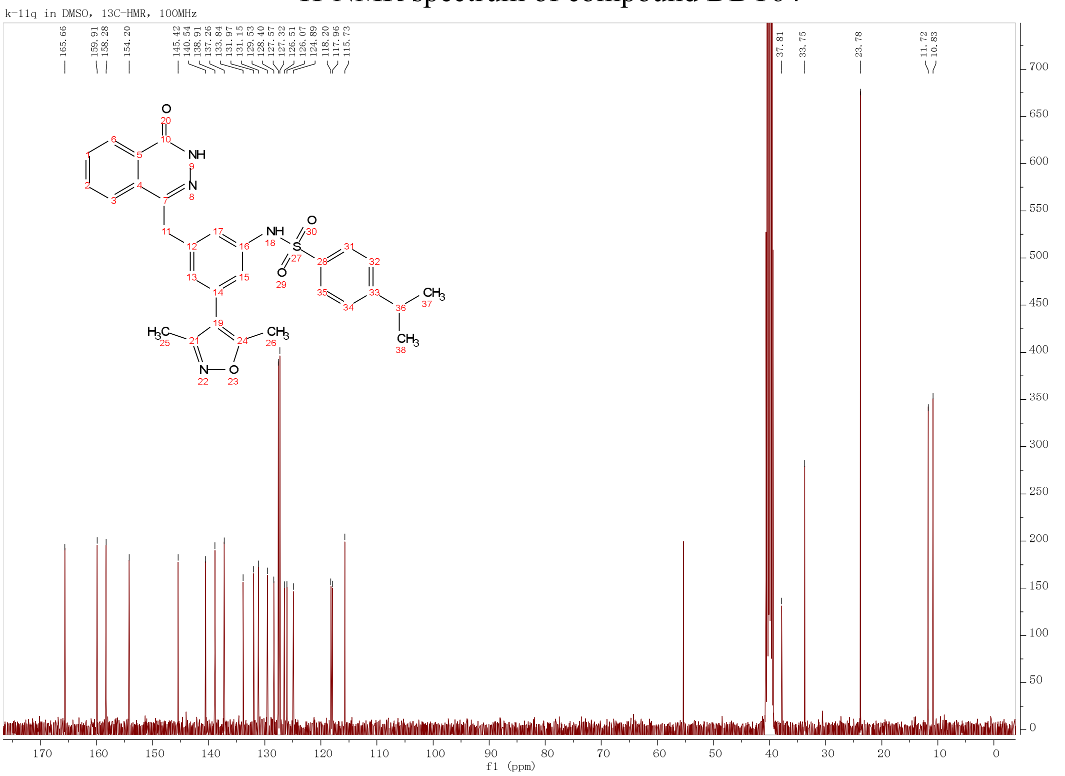

^{13}C-NMR spectrum of compound DDT64

k-11K' in DMSO, 1H-HMR, 400MHz

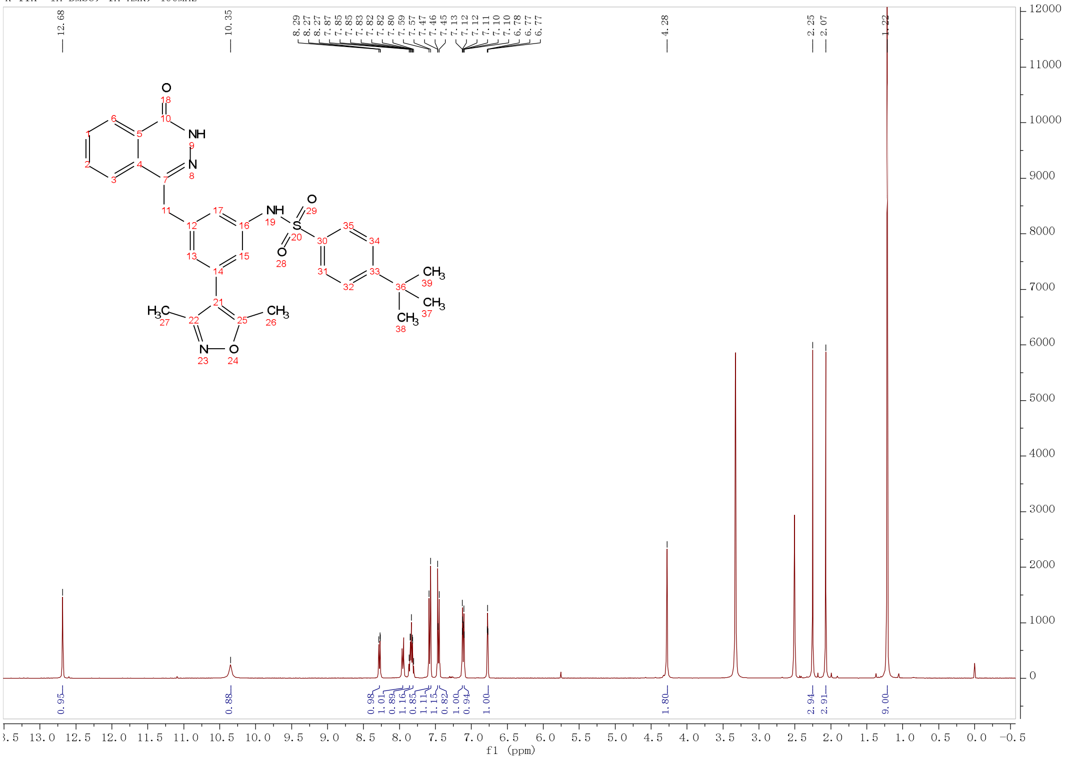

^{1}H-NMR spectrum of compound DDT65

k-11K' in DMSO, 13C-HMR, 100MHz

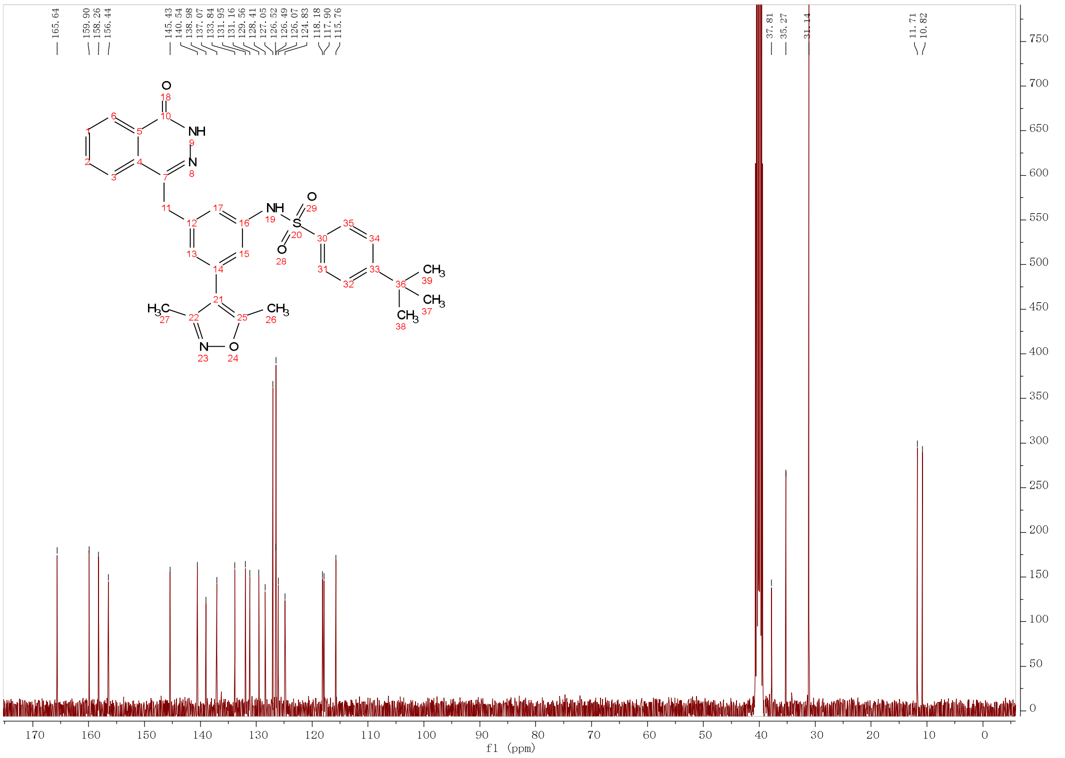

^{13}C-NMR spectrum of compound DDT65

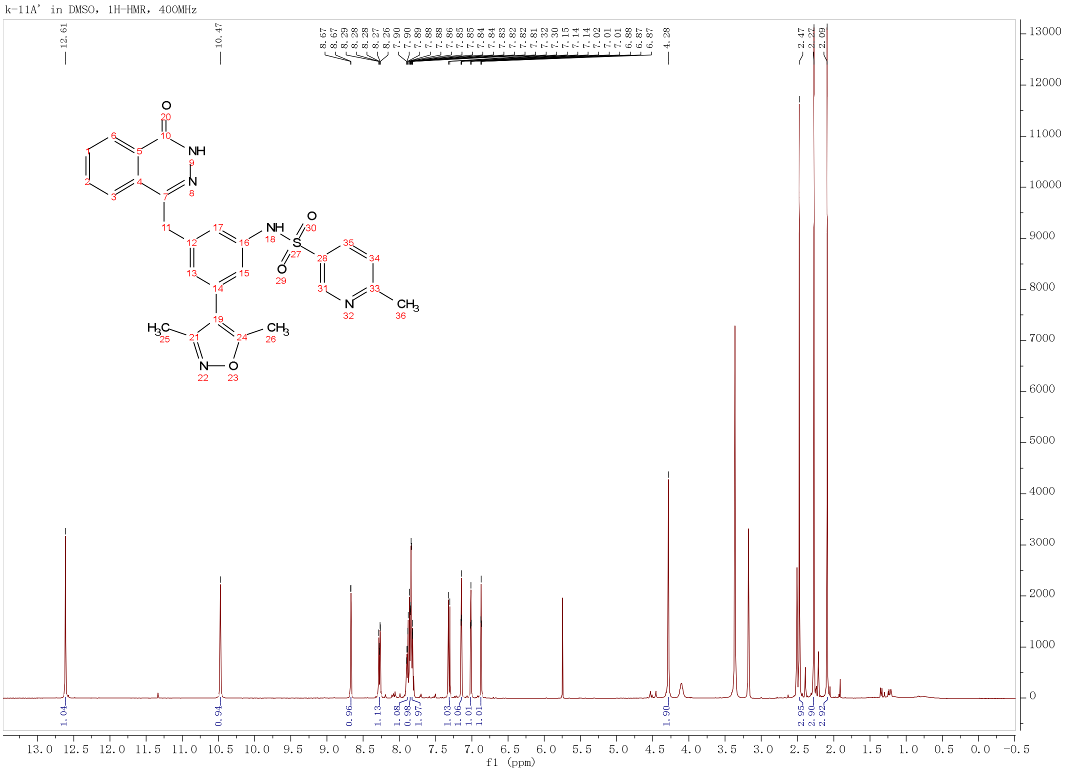

^{1}H-NMR spectrum of compound DDT66

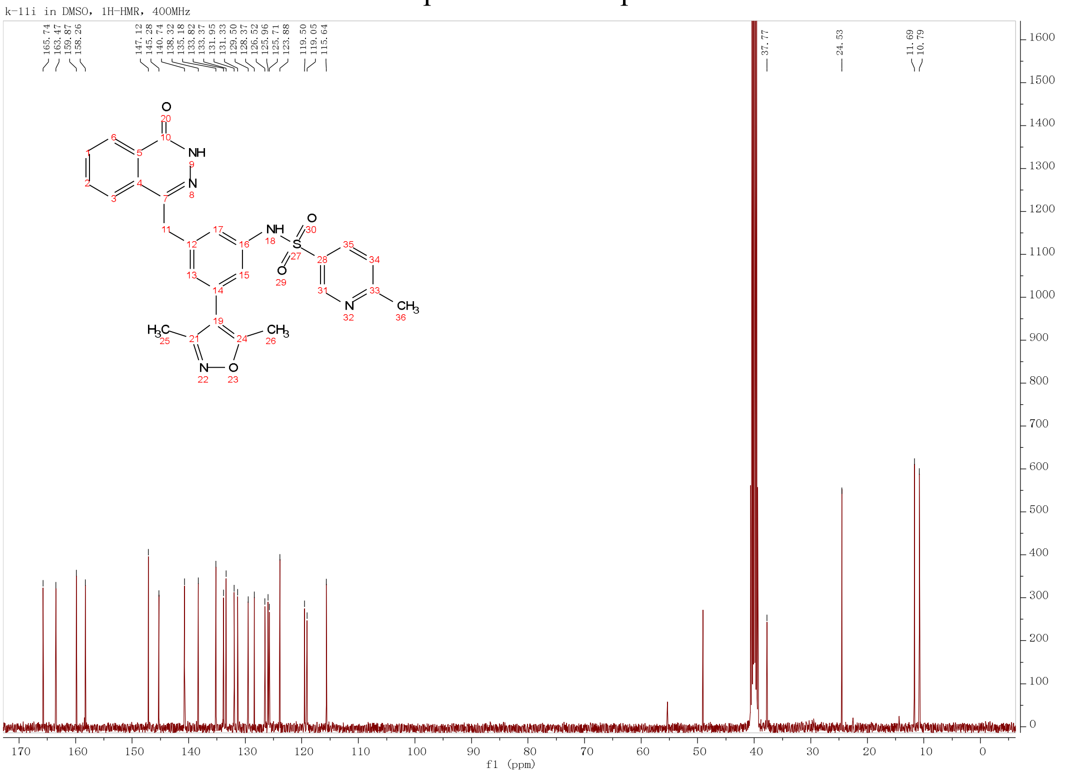

^{13}C-NMR spectrum of compound DDT66

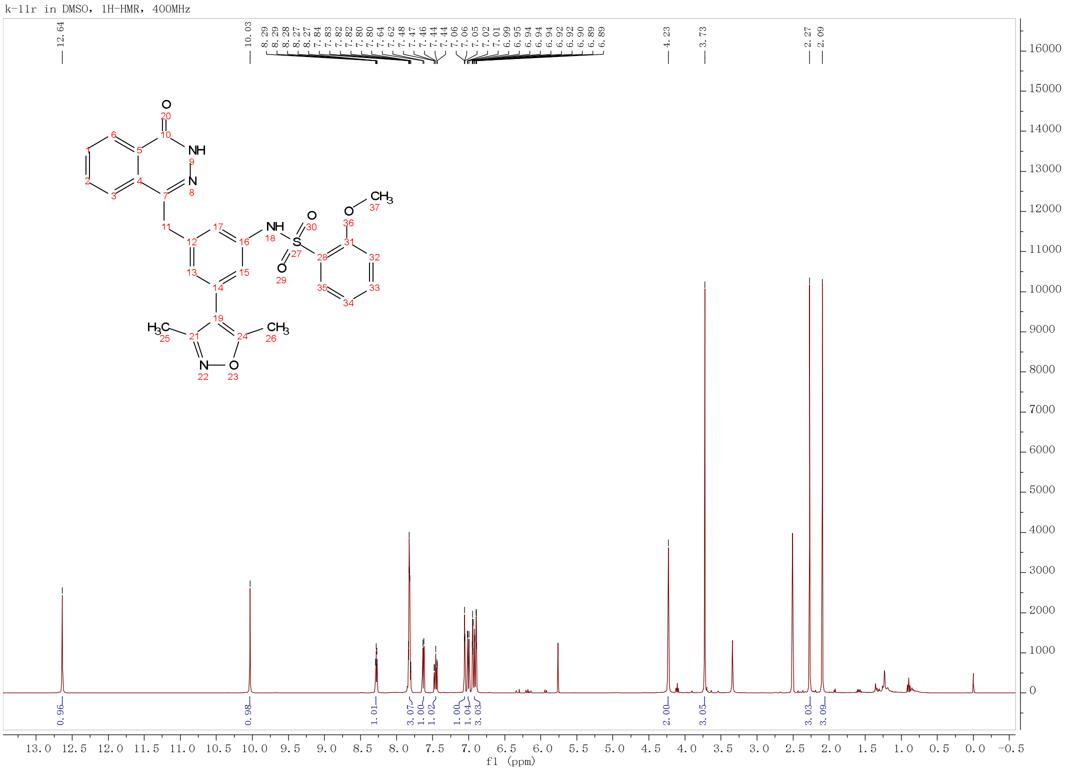

^{1}H-NMR spectrum of compound DDT67

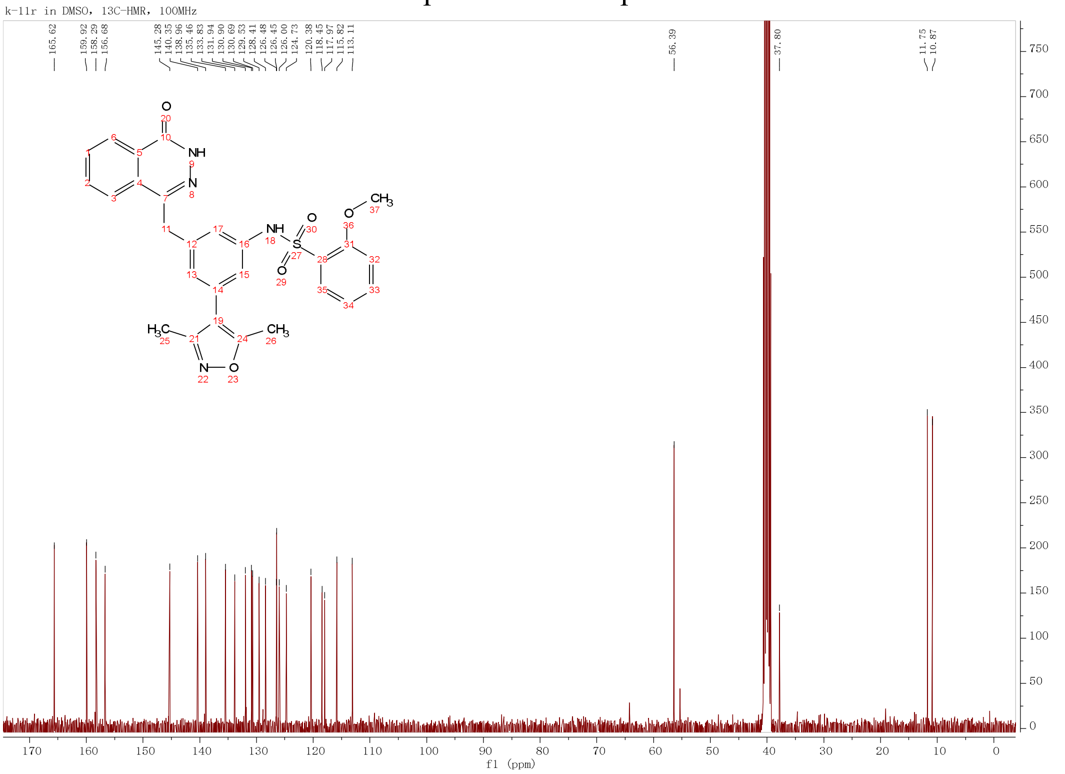

^{13}C-NMR spectrum of compound DDT67

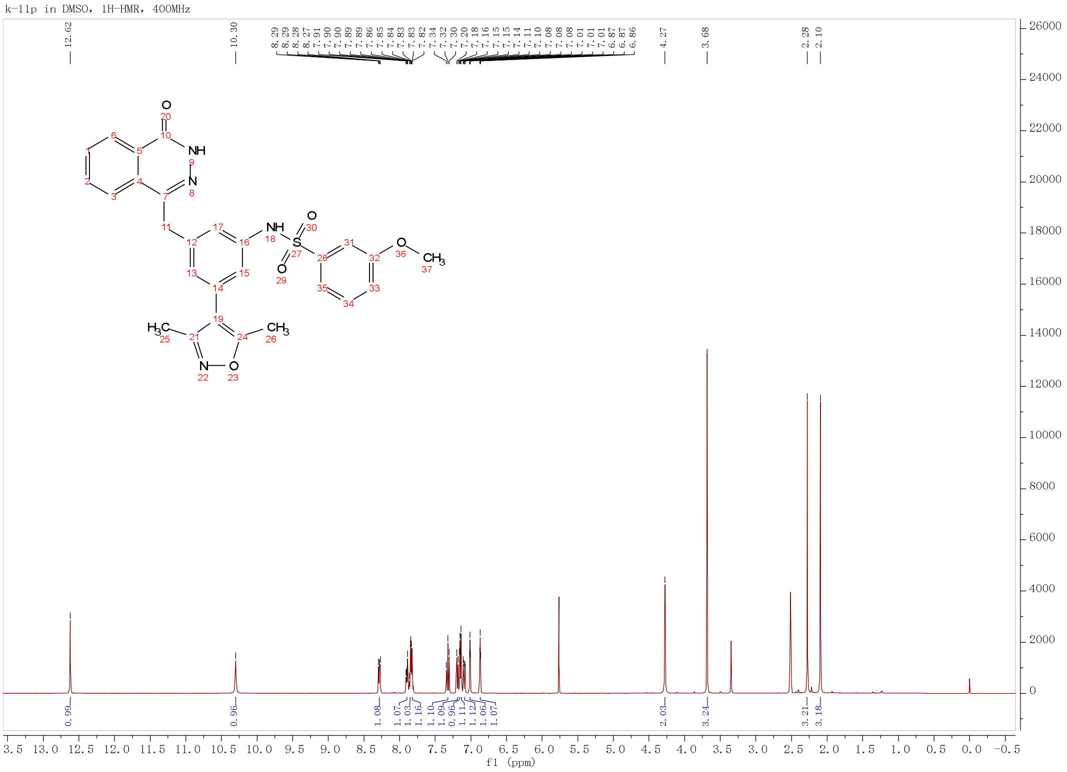

^{1}H-NMR spectrum of compound DDT68

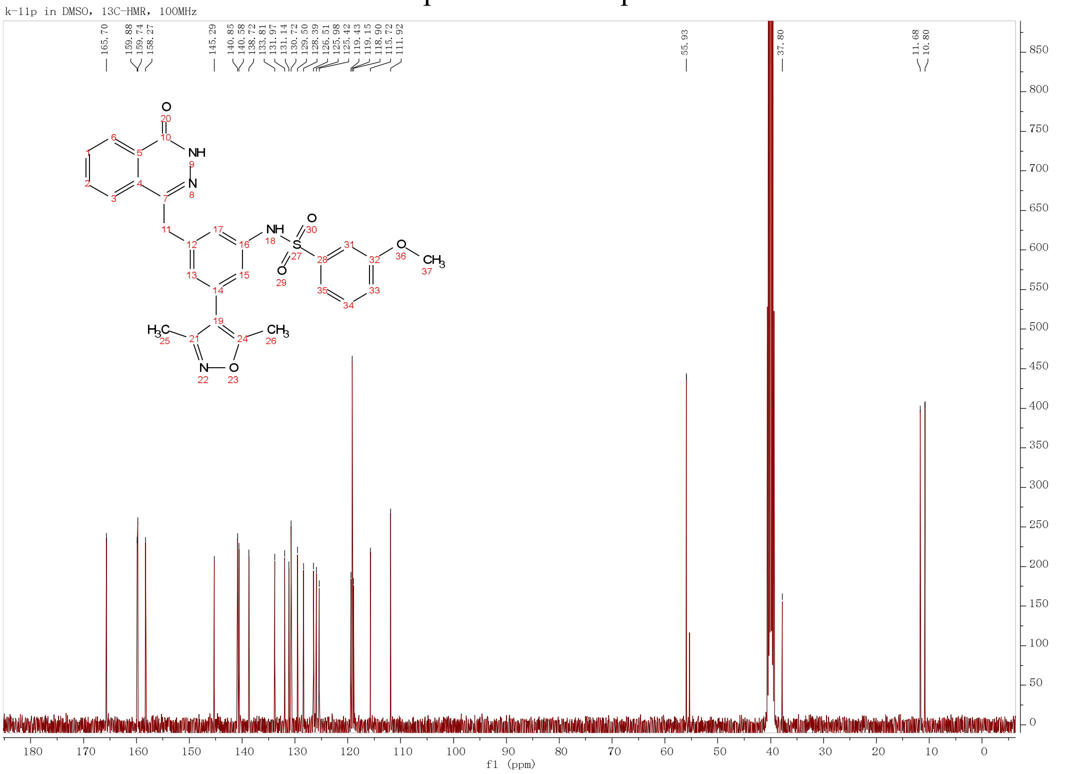

^{13}C-NMR spectrum of compound DDT68

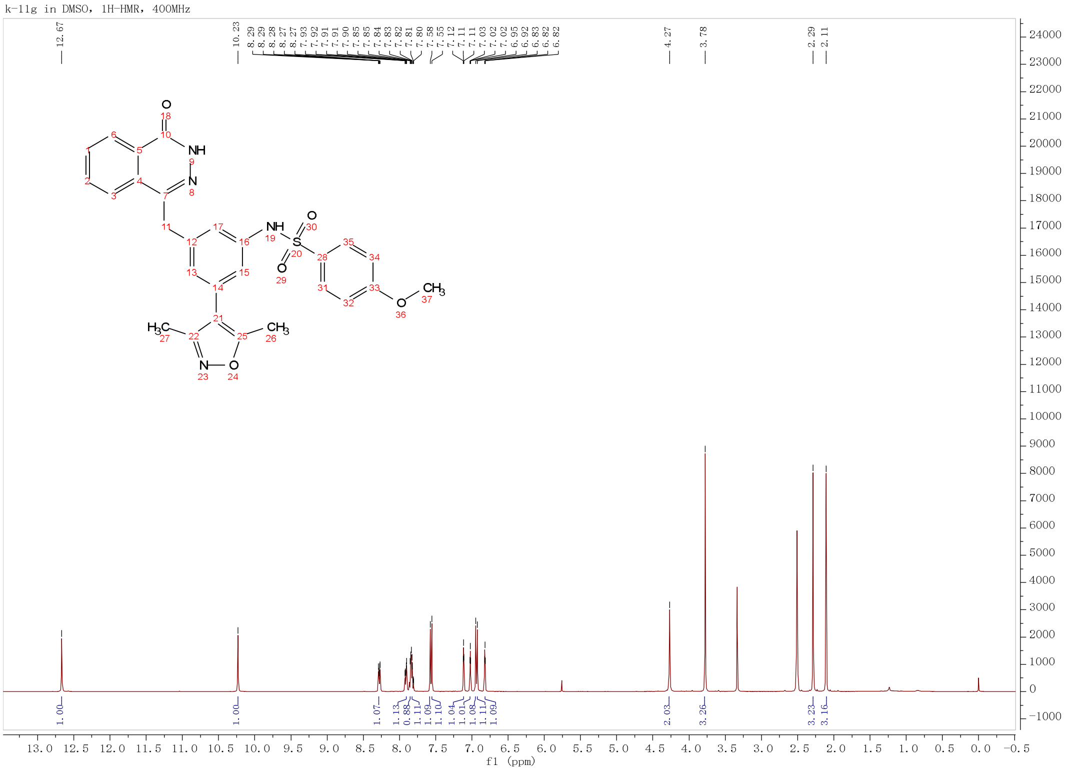

^{1}H-NMR spectrum of compound DDT69

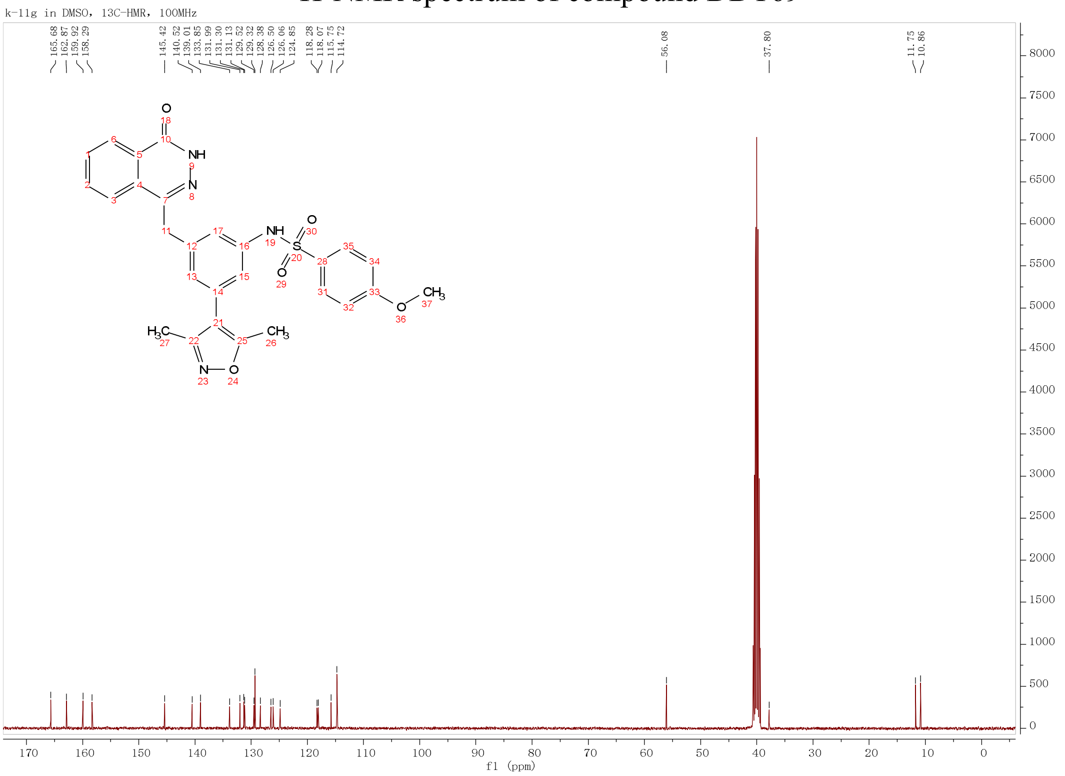

^{13}C-NMR spectrum of compound DDT69

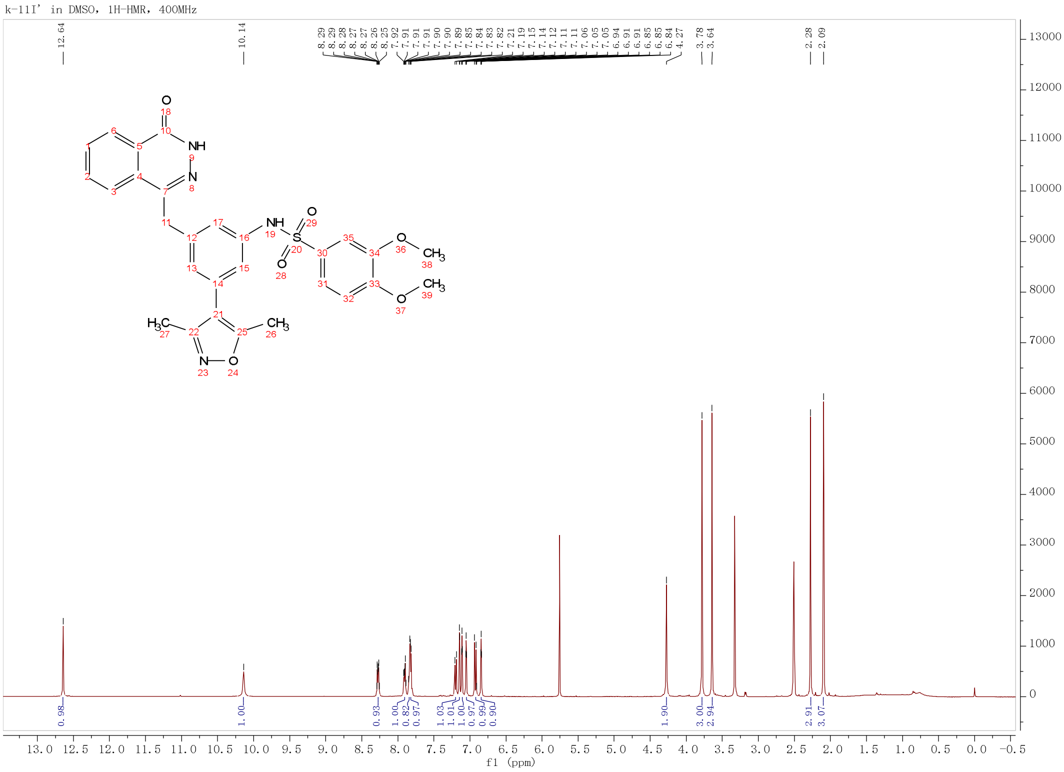

^{1}H-NMR spectrum of compound DDT70

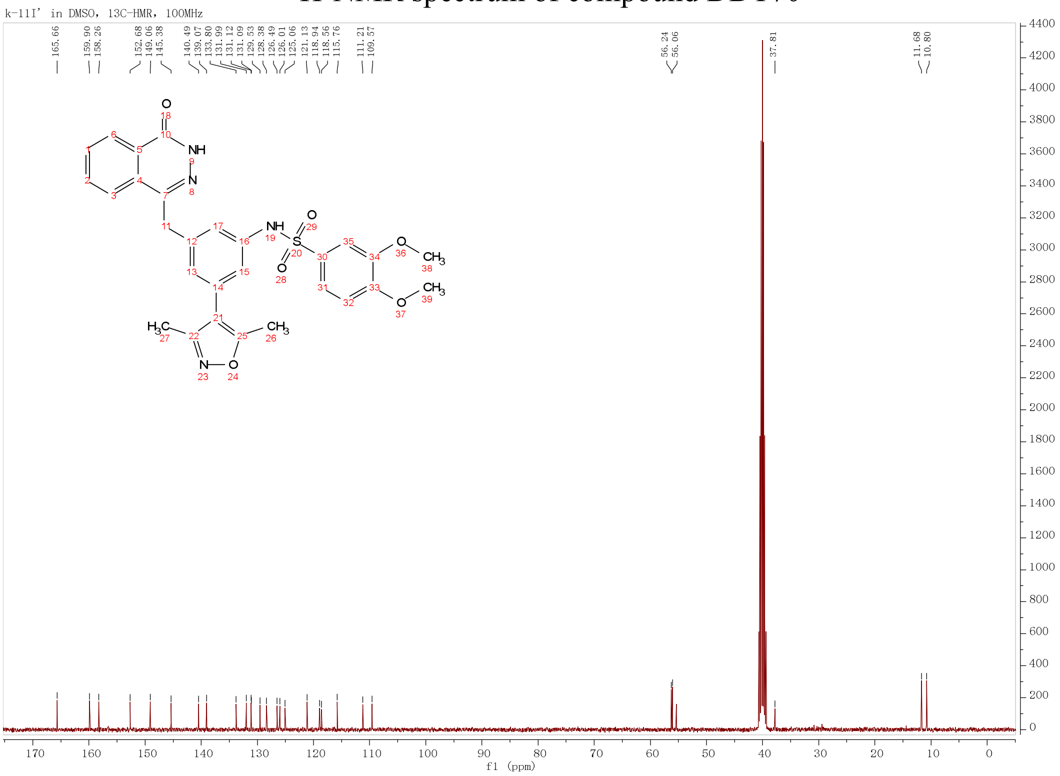

^{13}C-NMR spectrum of compound DDT70

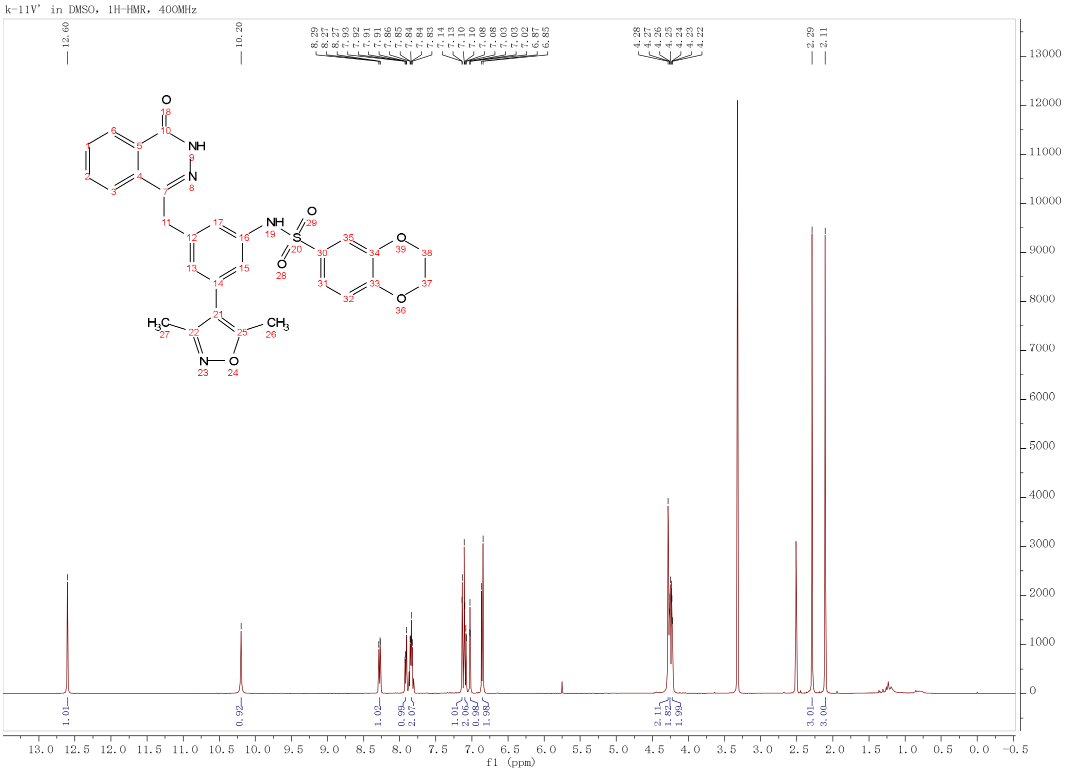

^{1}H-NMR spectrum of compound DDT71

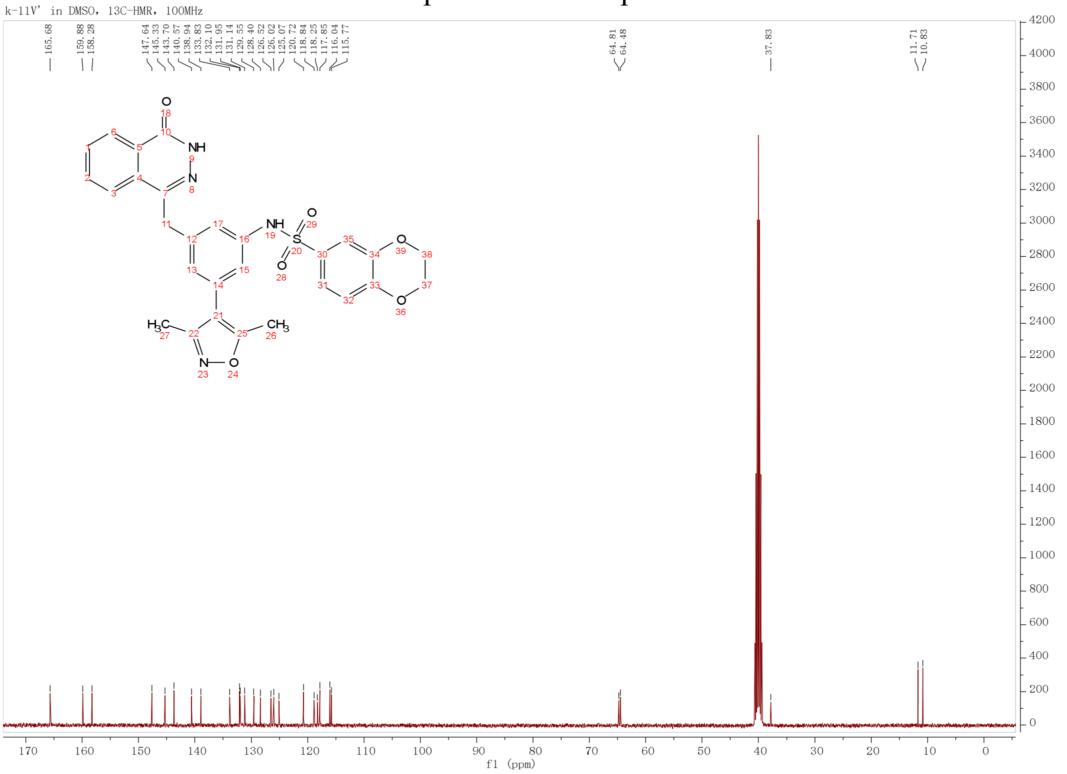

^{13}C-NMR spectrum of compound DDT71

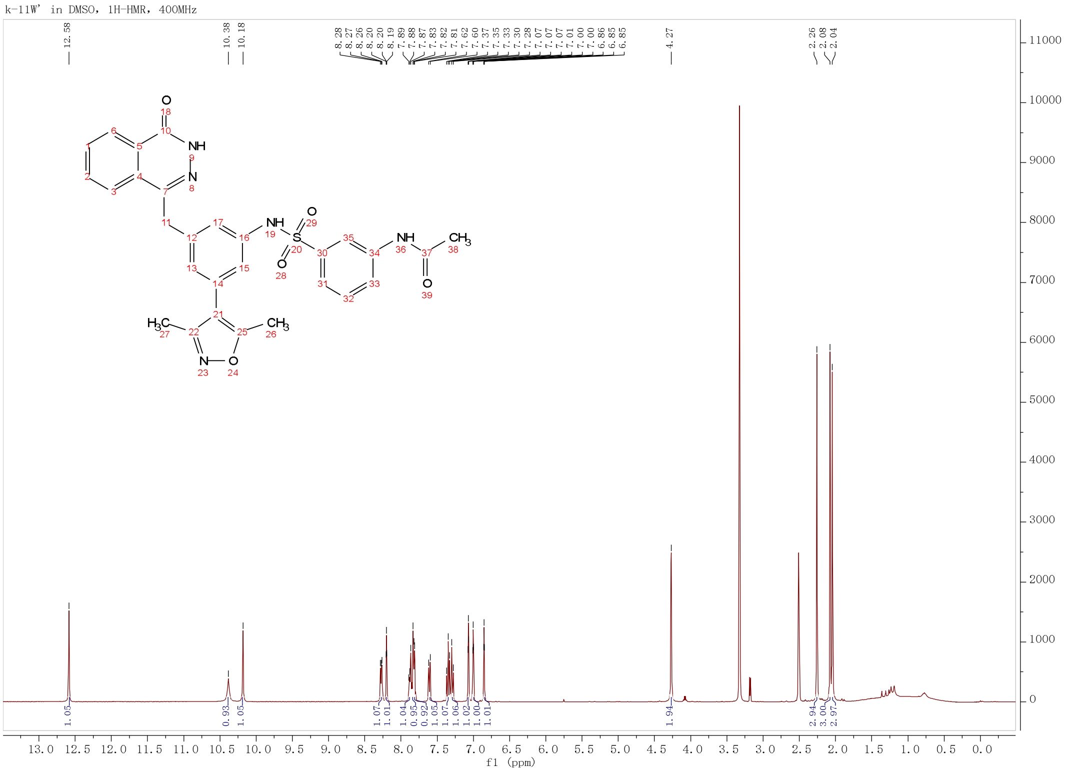

^{1}H-NMR spectrum of compound DDT72

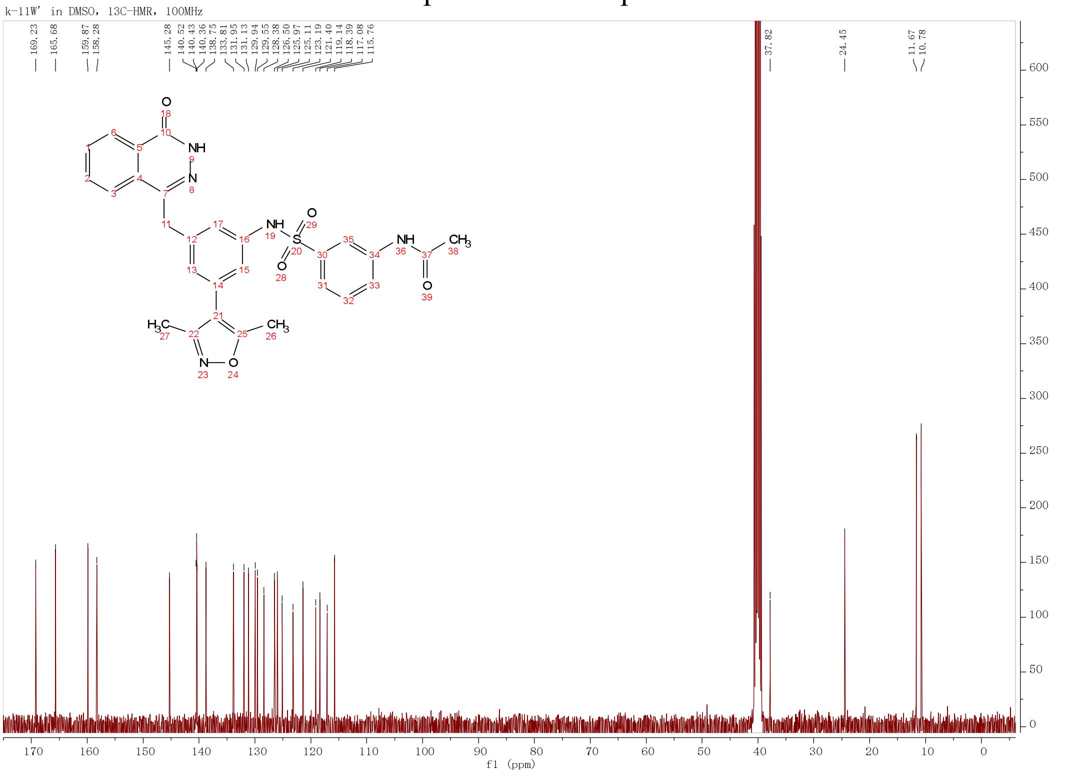

^{13}C-NMR spectrum of compound DDT72

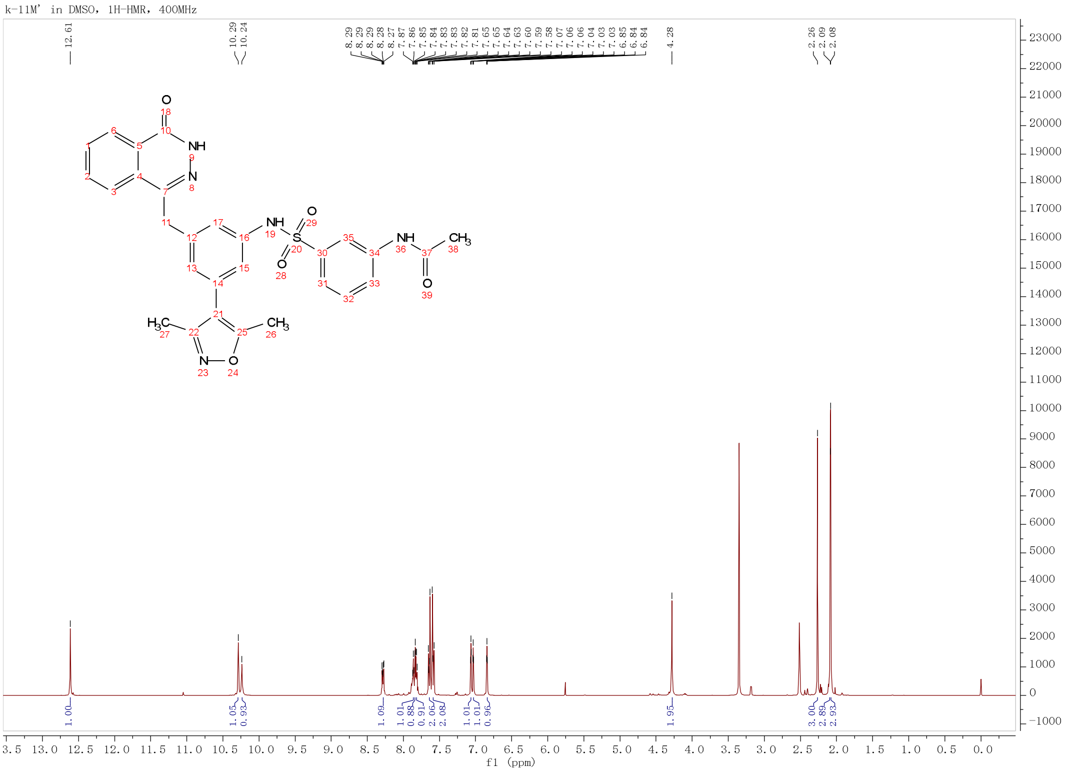

^{1}H-NMR spectrum of compound DDT73

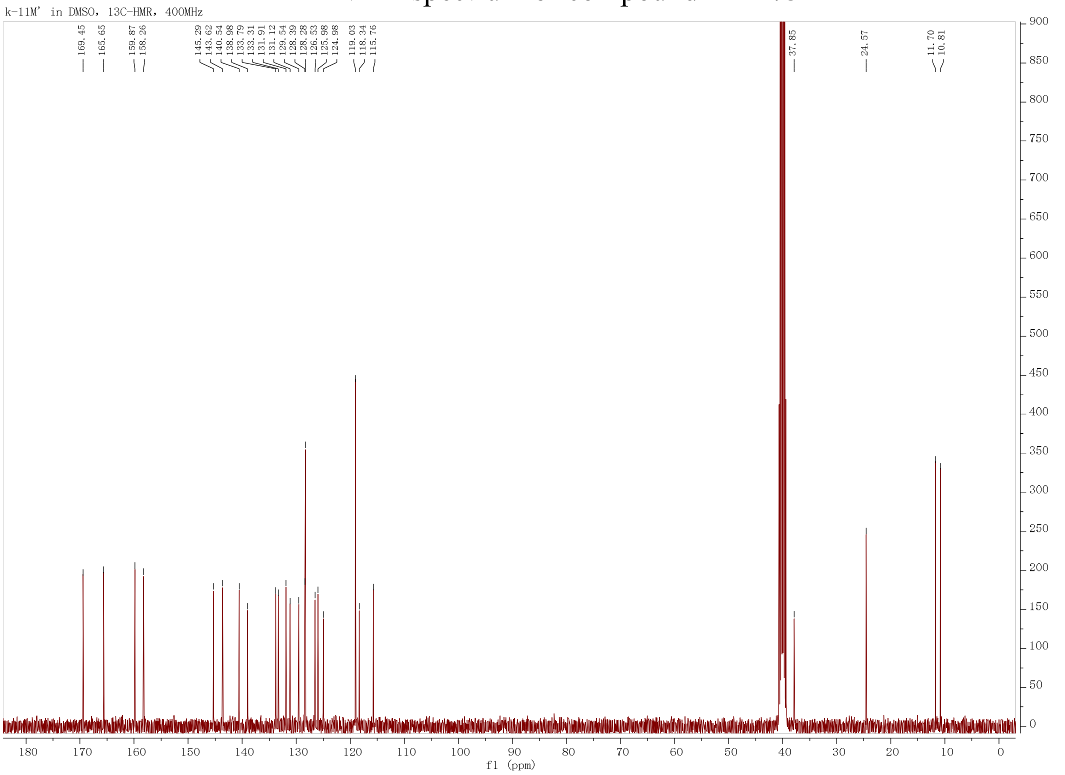

^{13}C-NMR spectrum of compound DDT73

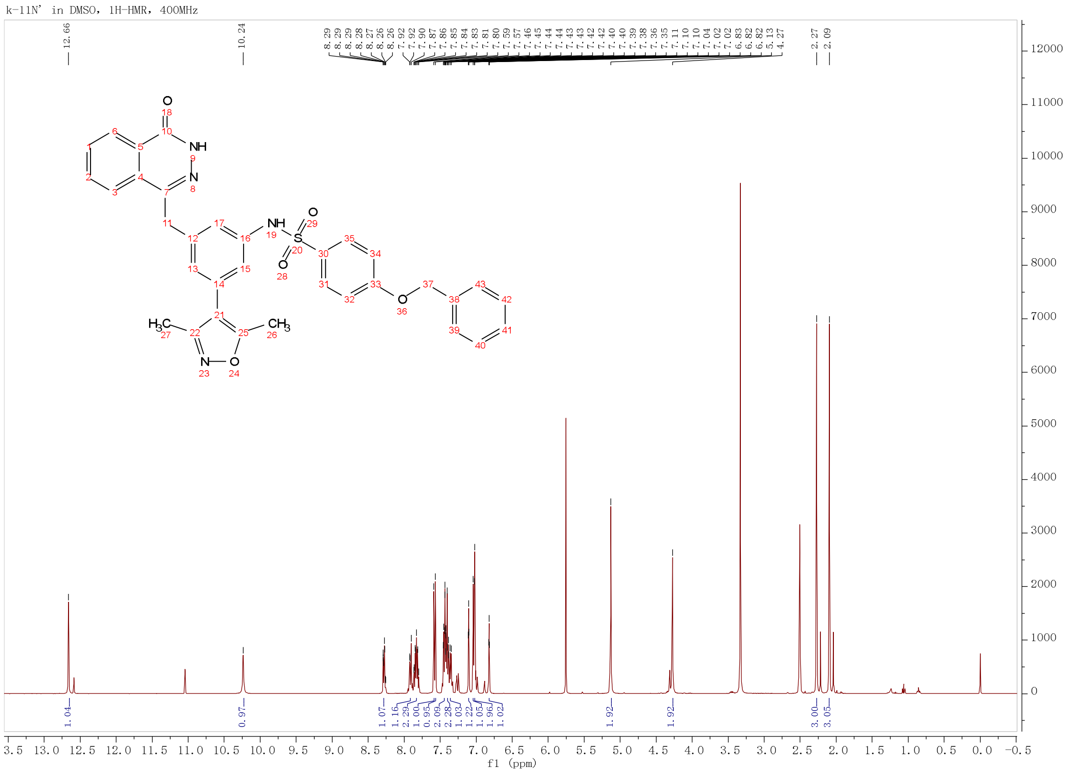

^{1}H-NMR spectrum of compound DDT74

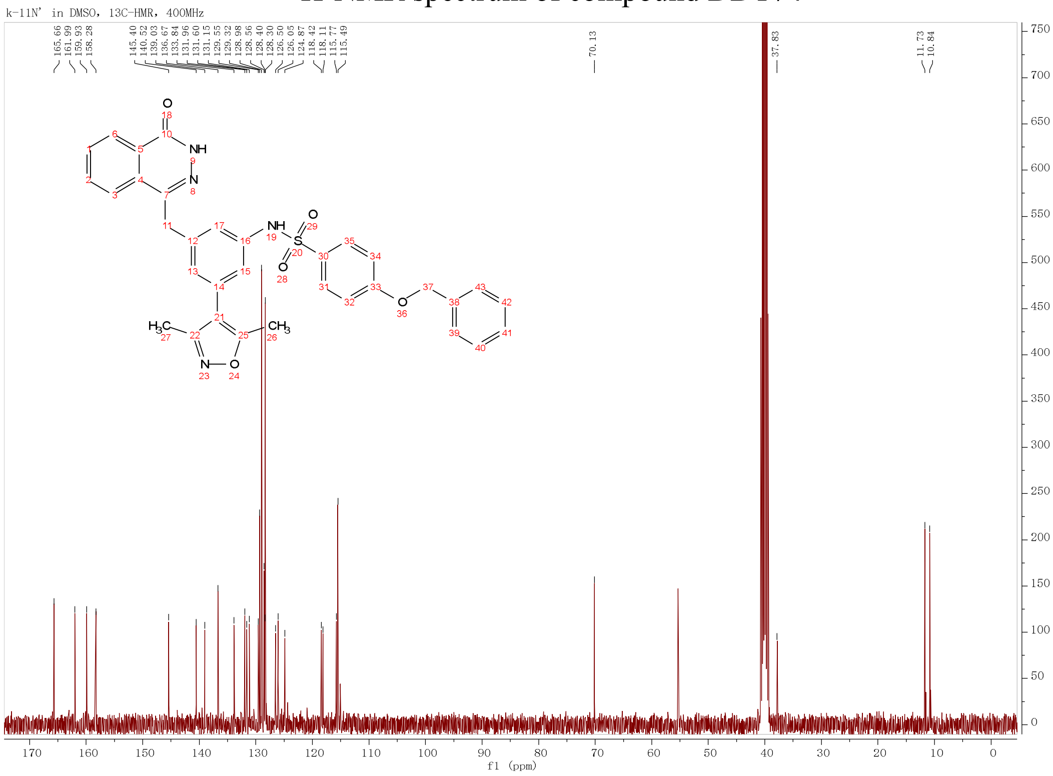

^{13}C-NMR spectrum of compound DDT74

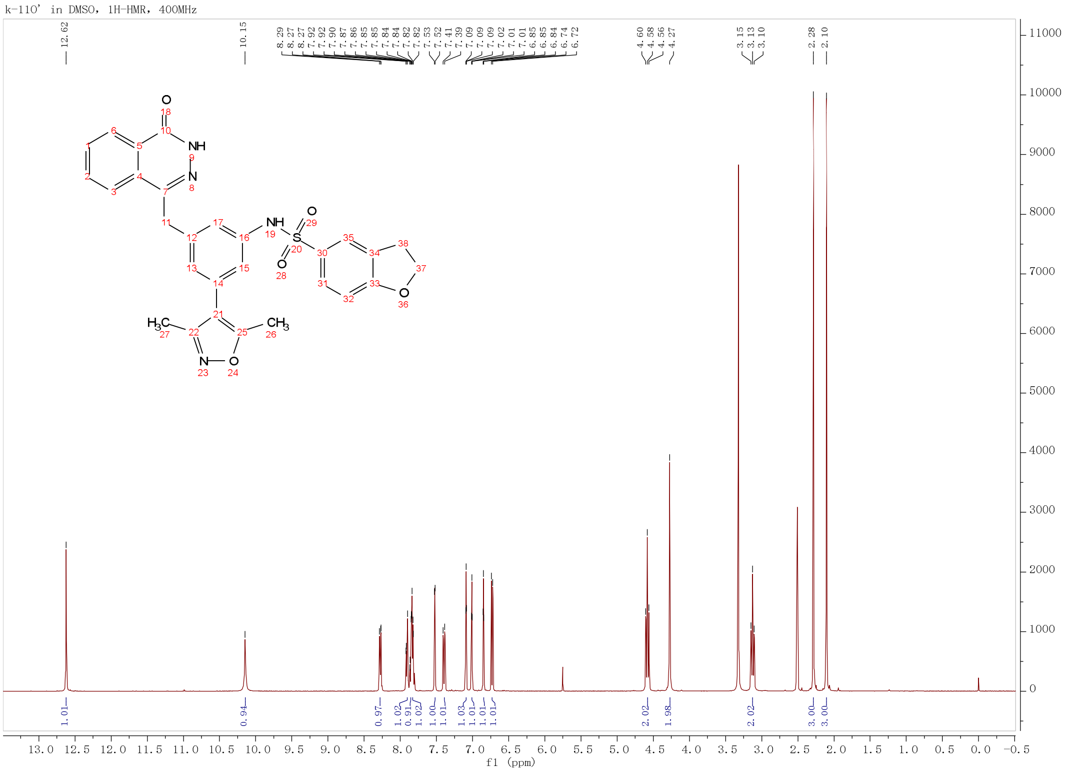

^{1}H-NMR spectrum of compound DDT75

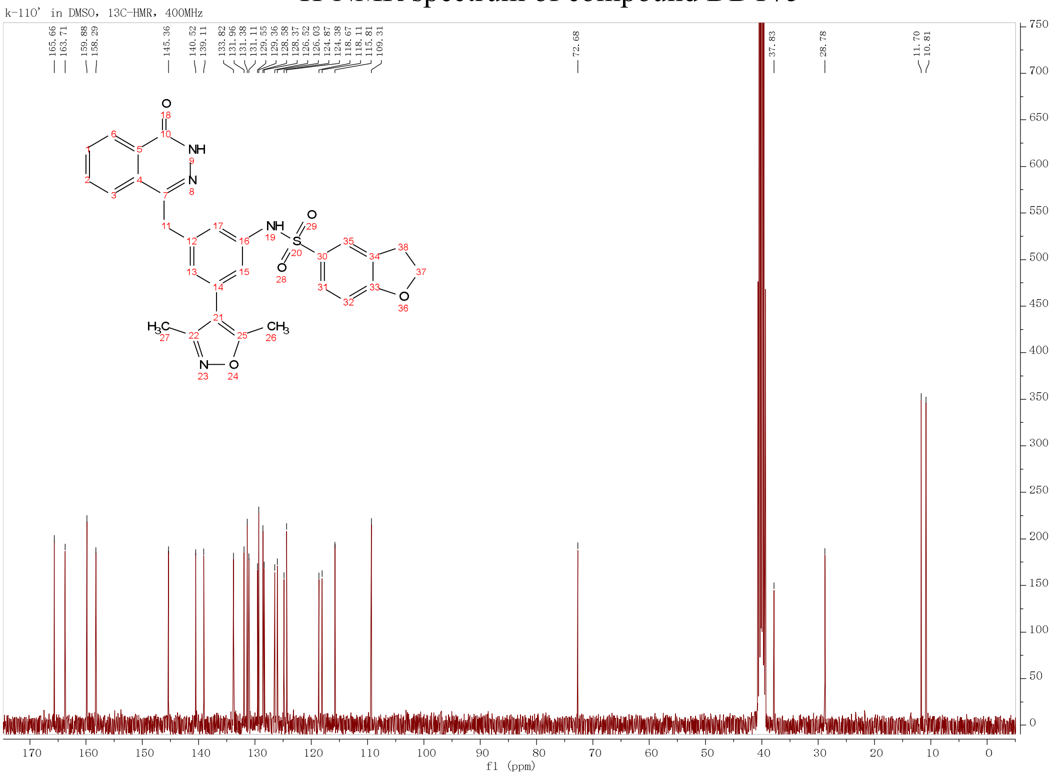

^{13}C-NMR spectrum of compound DDT75

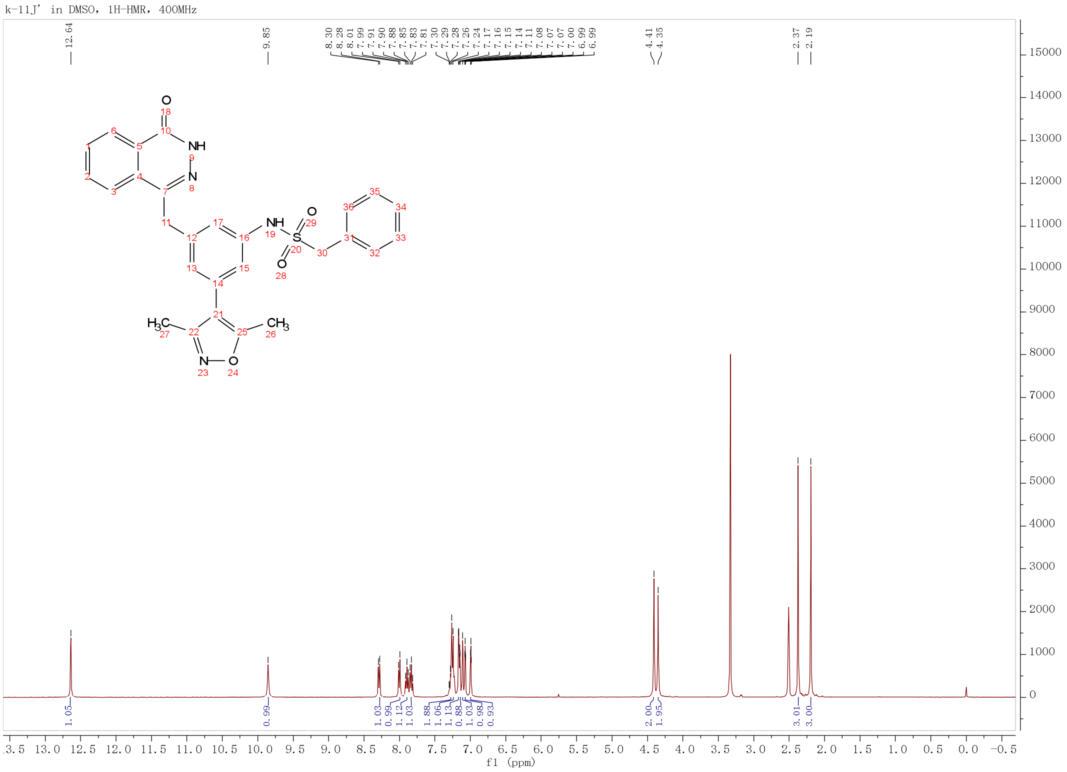

^{1}H-NMR spectrum of compound DDT76

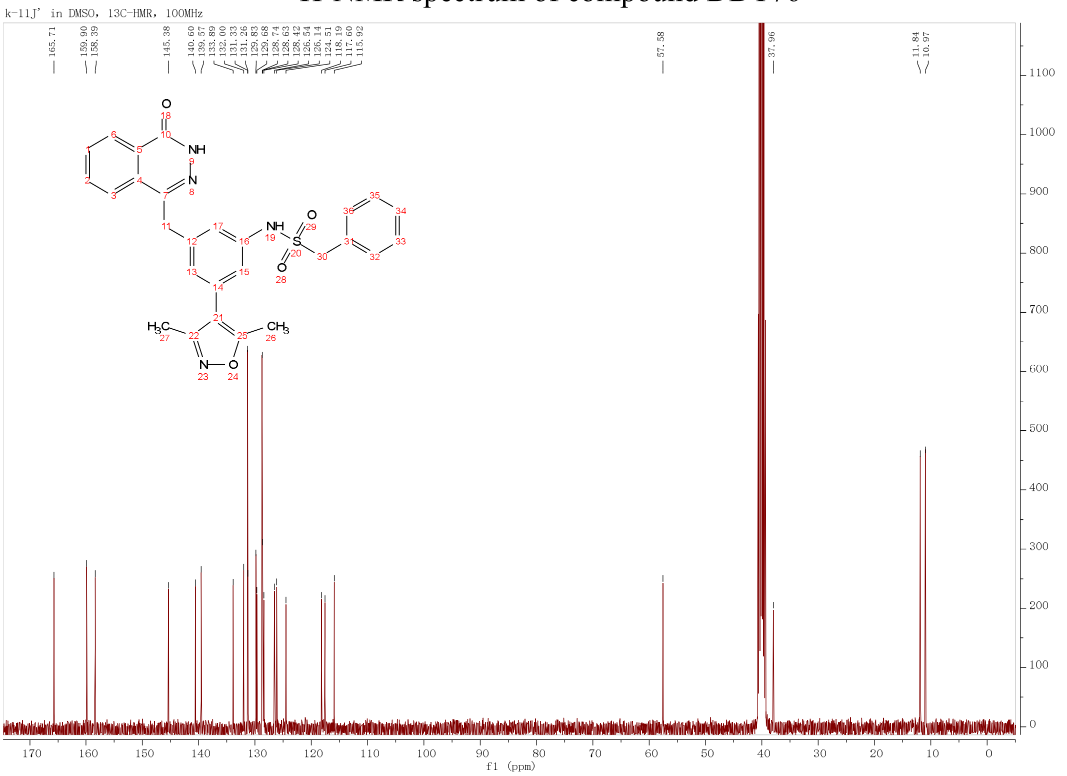

^{13}C-NMR spectrum of compound DDT76

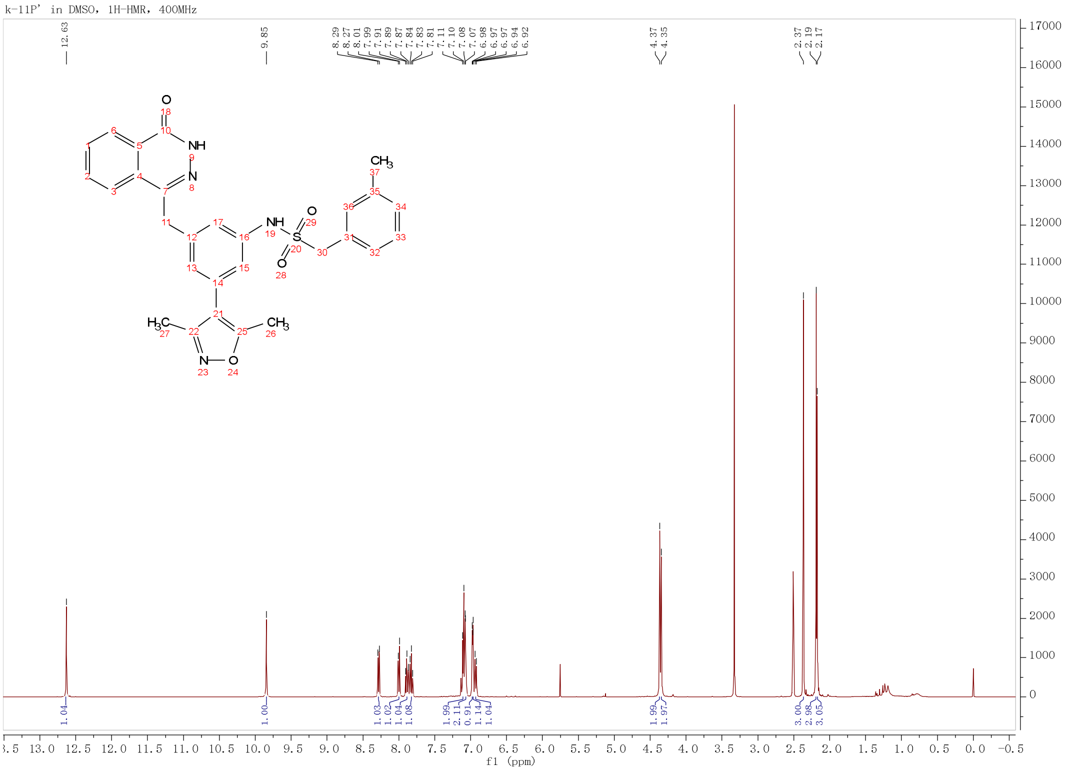

^{1}H-NMR spectrum of compound DDT77

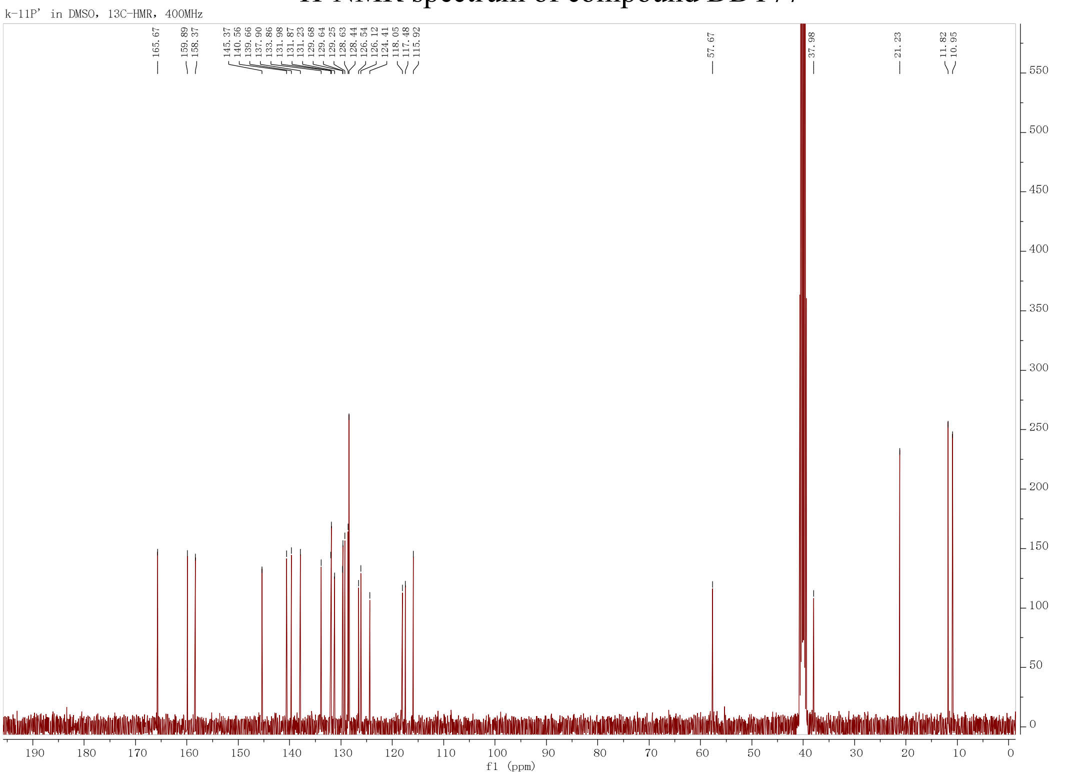

^{13}C-NMR spectrum of compound DDT77

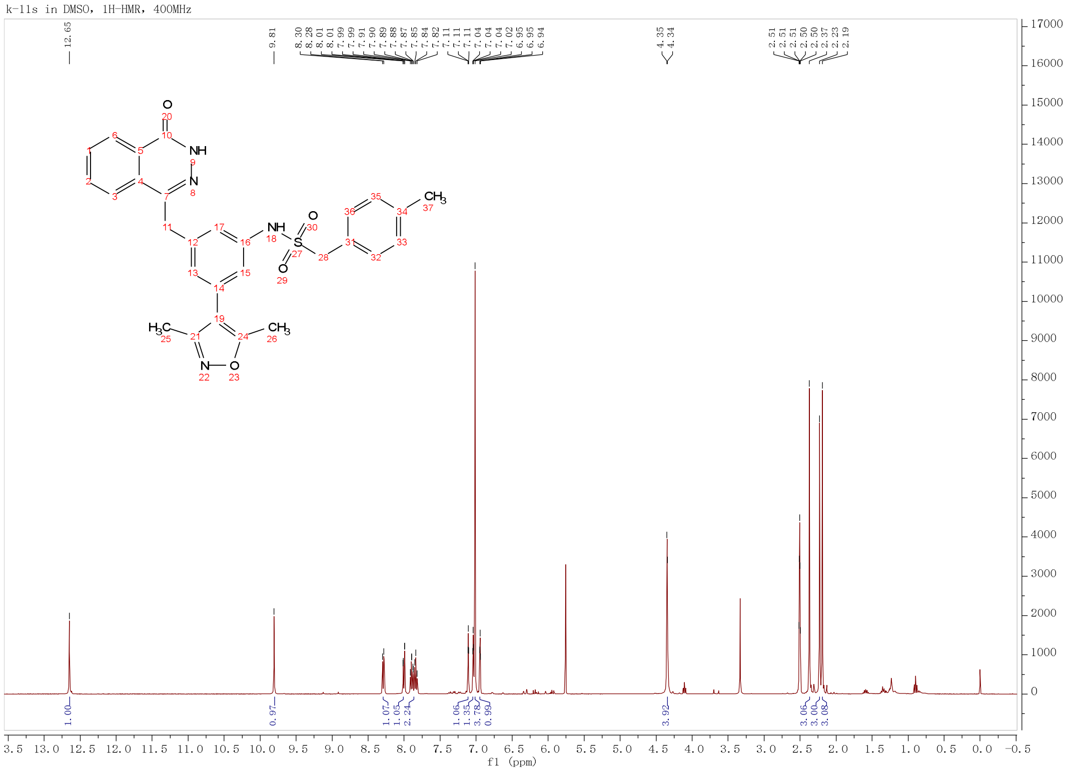

^{1}H-NMR spectrum of compound DDT78

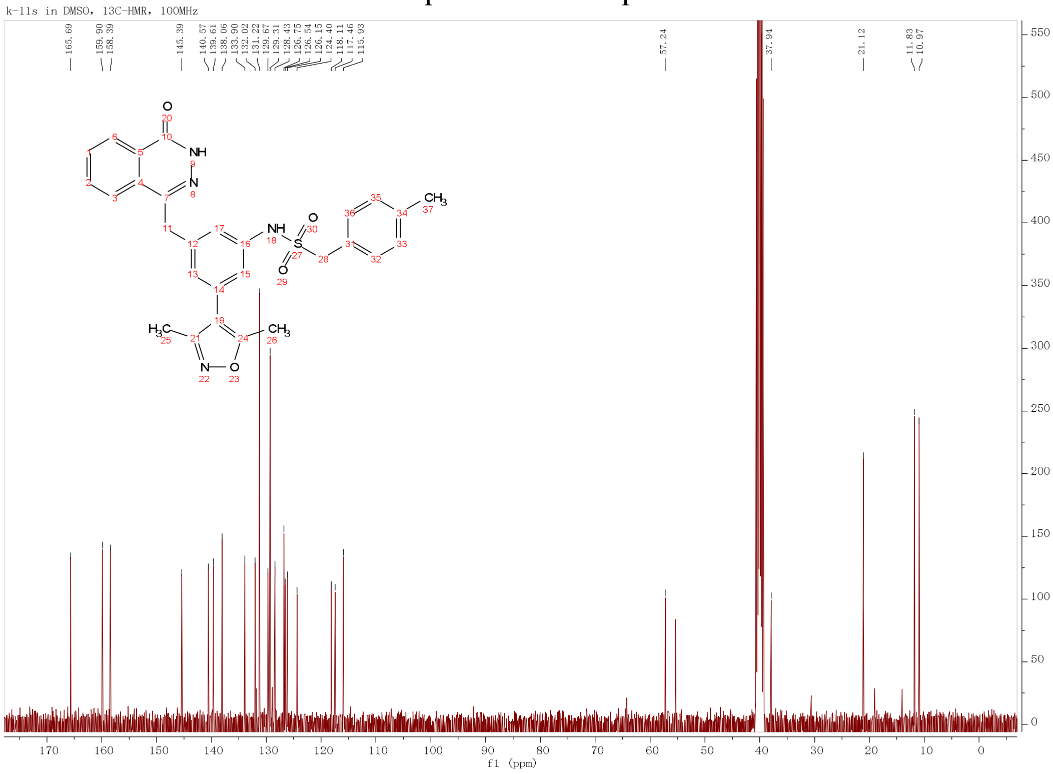

^{13}C-NMR spectrum of compound DDT78

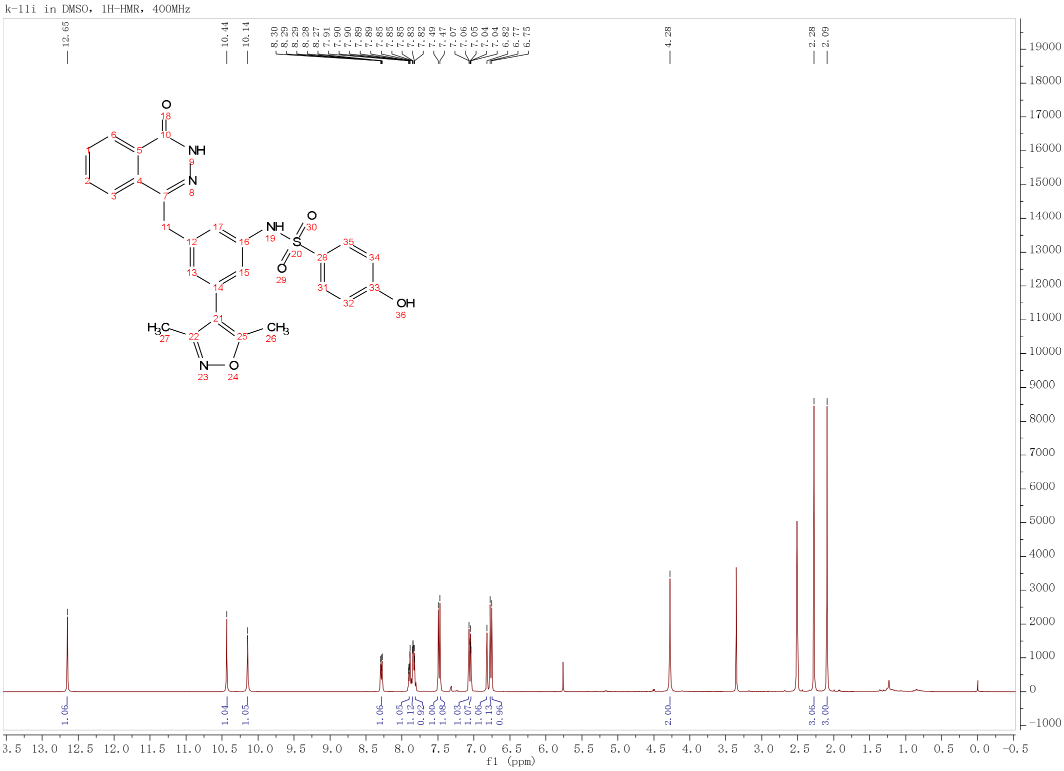

^{1}H-NMR spectrum of compound DDT79

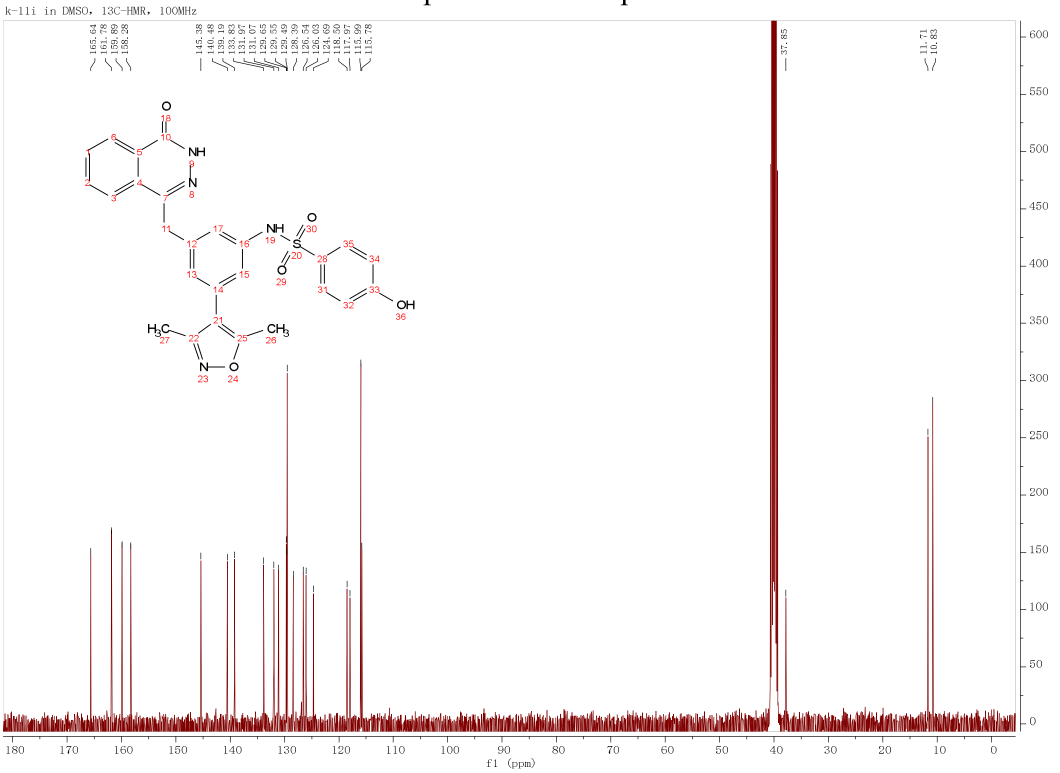

^{13}C-NMR spectrum of compound DDT79

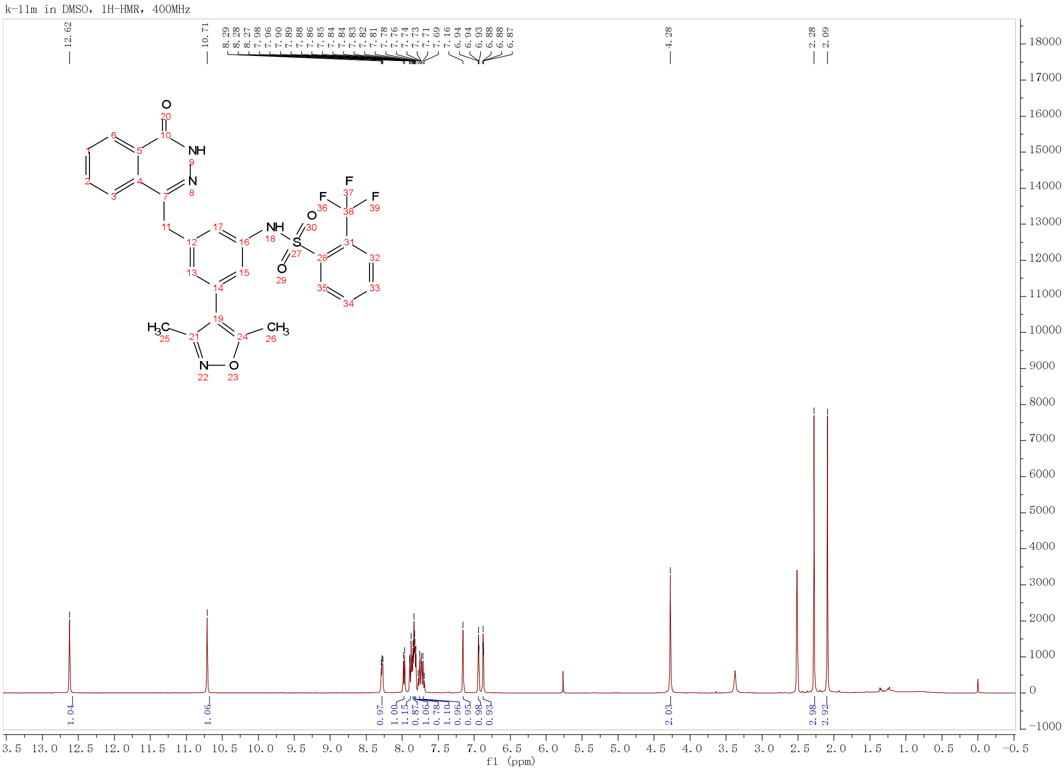

^{1}H-NMR spectrum of compound DDT80

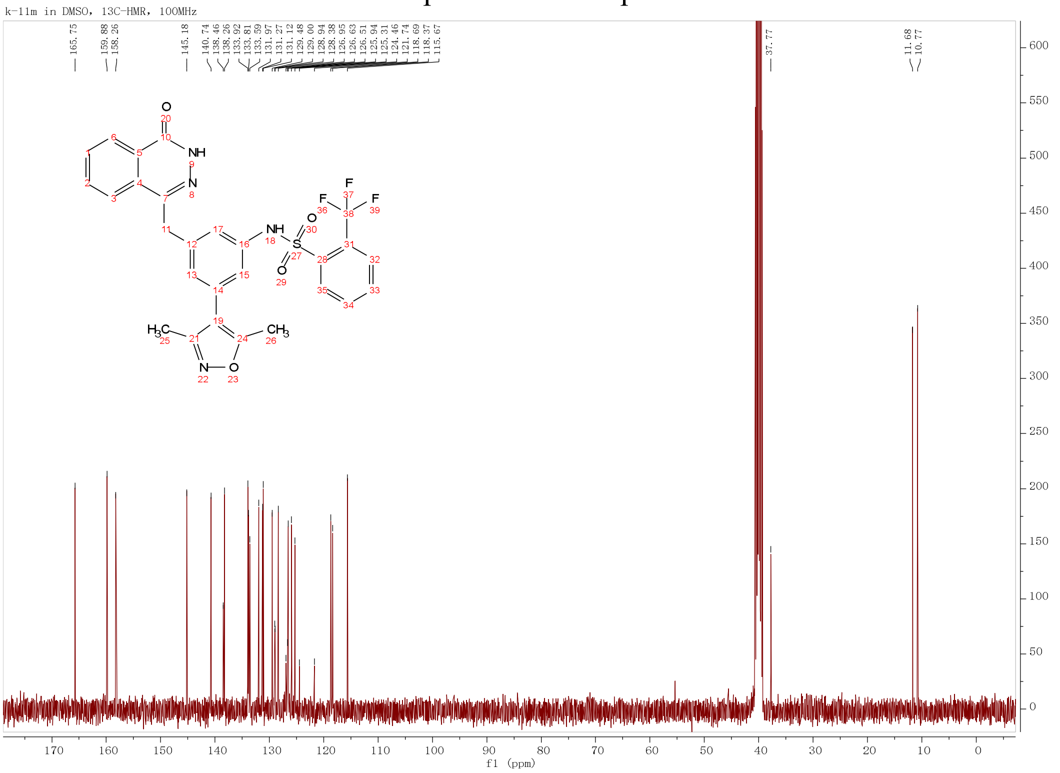

^{13}C-NMR spectrum of compound DDT80

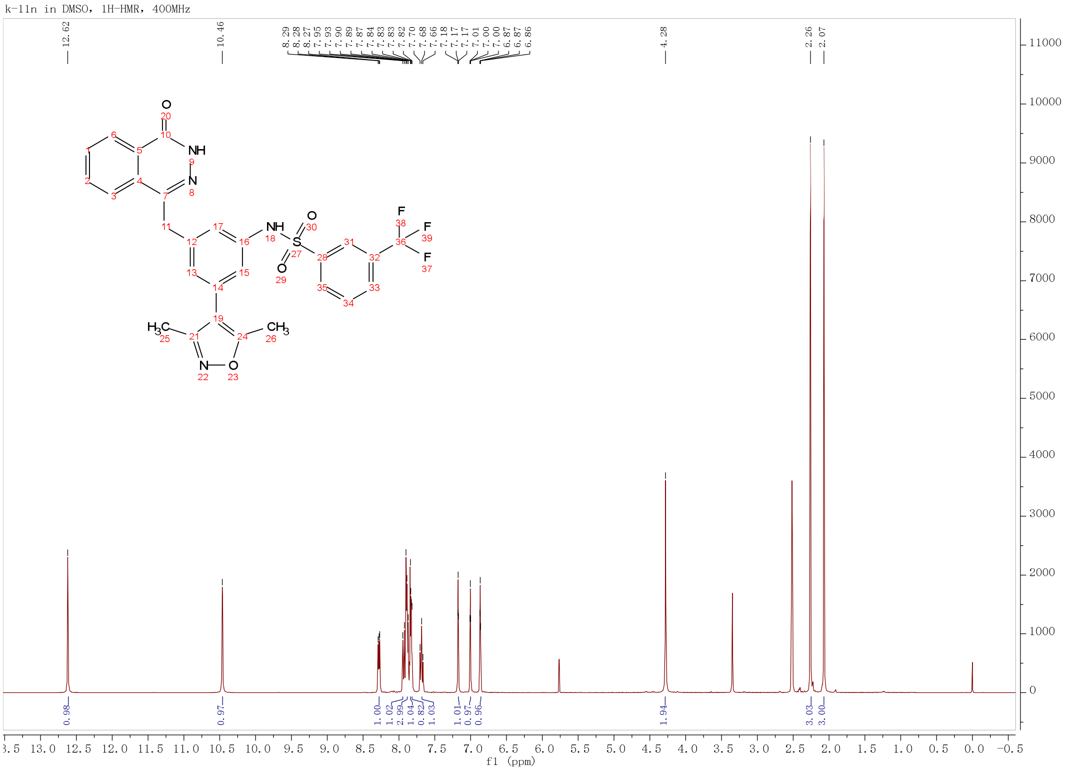

^{1}H-NMR spectrum of compound DDT81

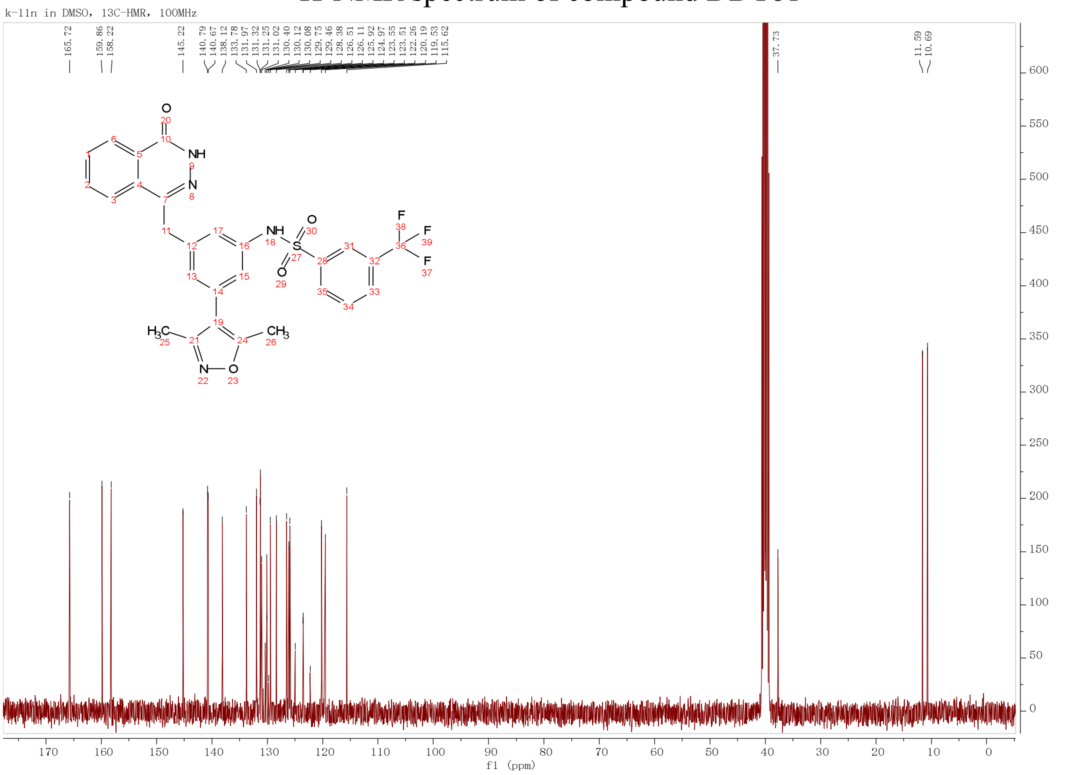

^{13}C-NMR spectrum of compound DDT81

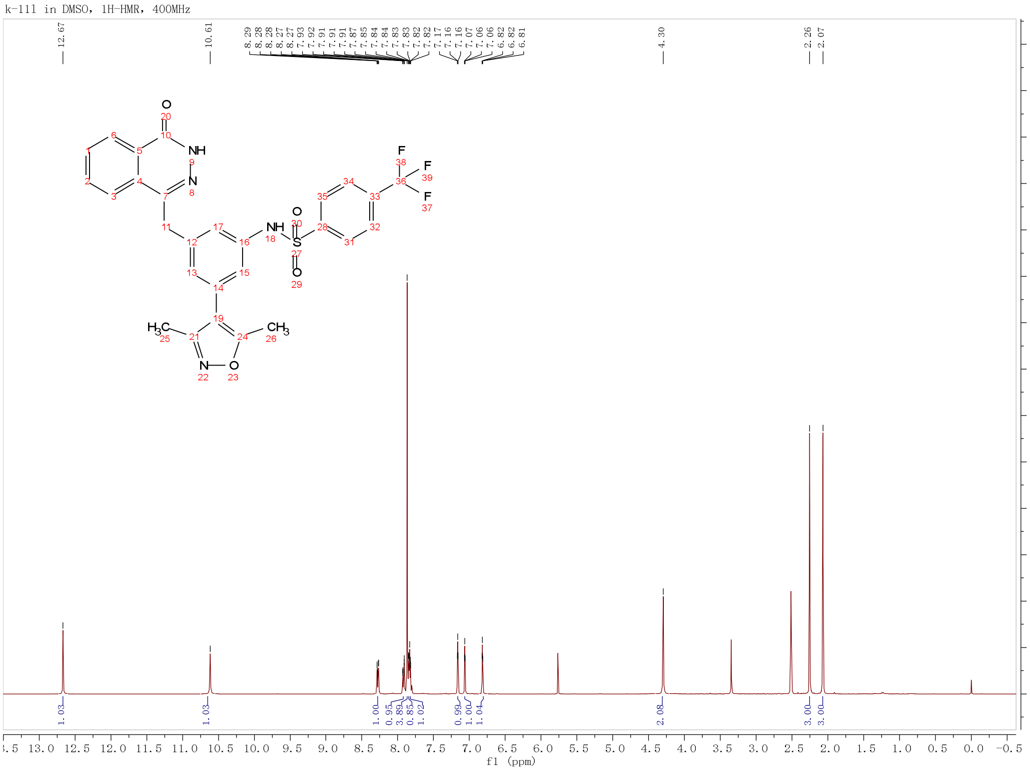

^{1}H-NMR spectrum of compound DDT82

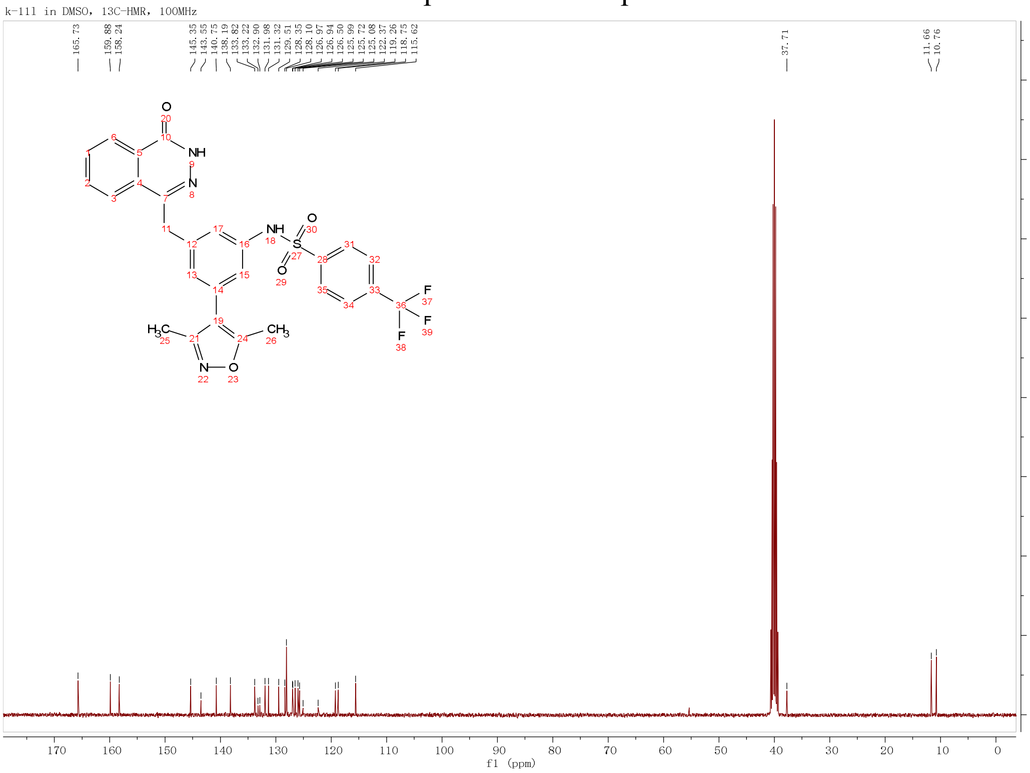

^{13}C-NMR spectrum of compound DDT82

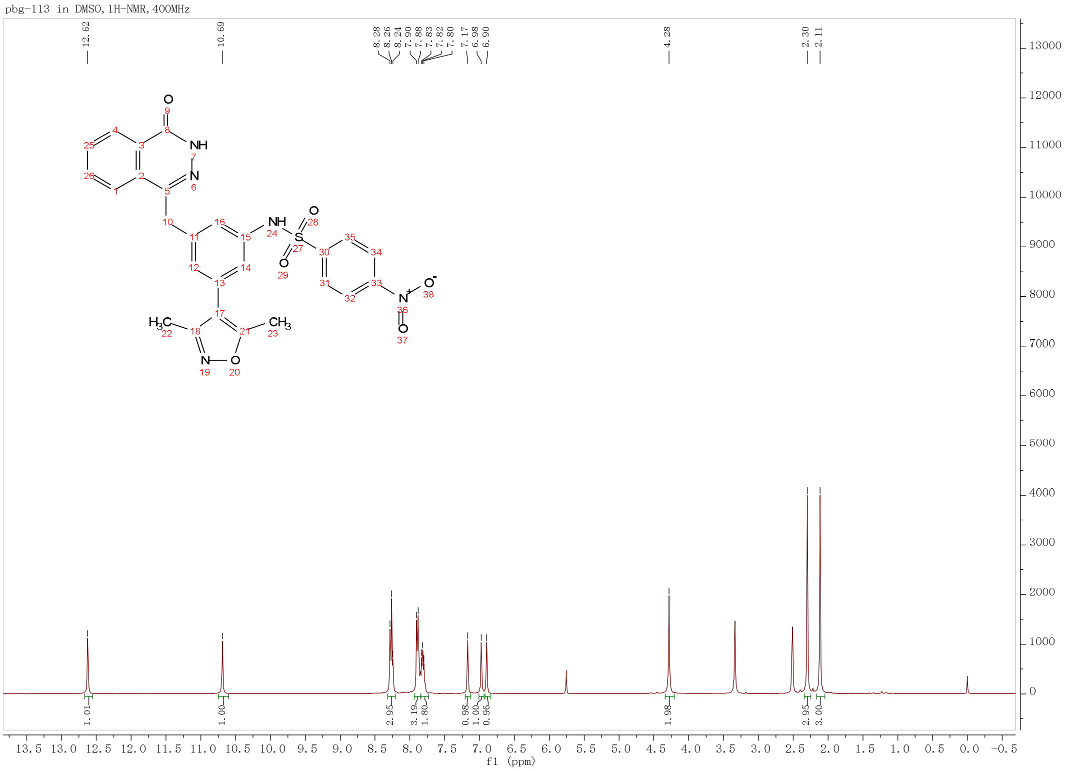

^{1}H-NMR spectrum of compound DDT83

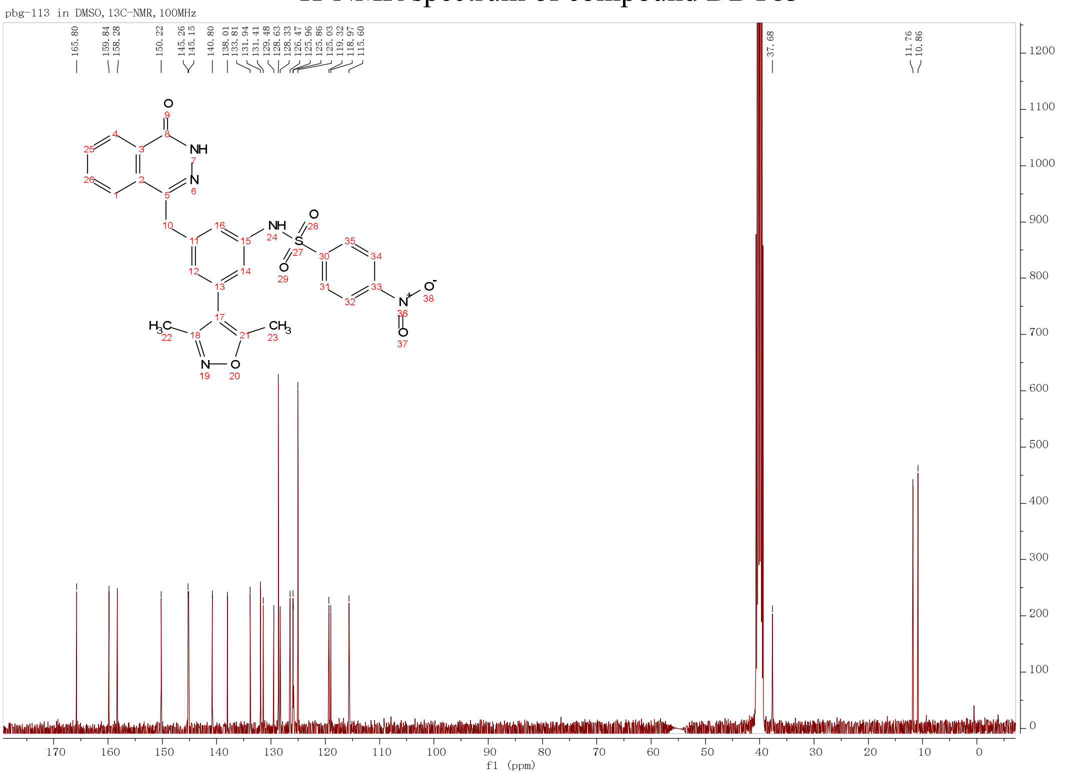

^{13}C-NMR spectrum of compound DDT83

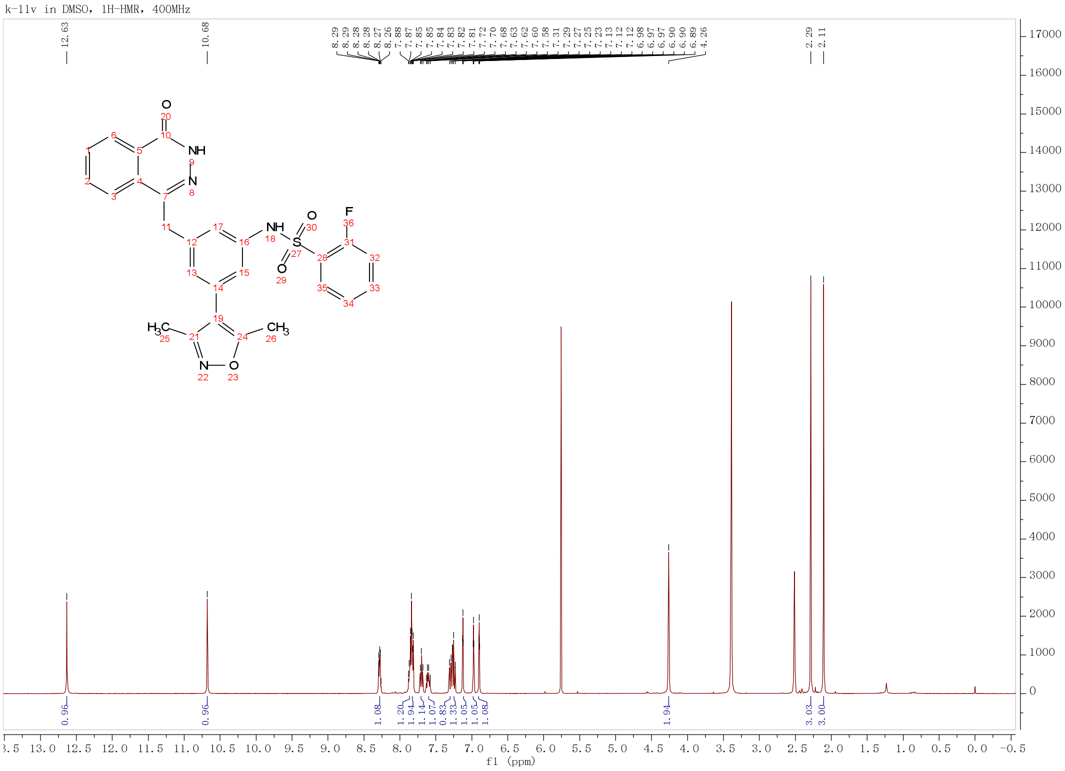

^{1}H-NMR spectrum of compound DDT84

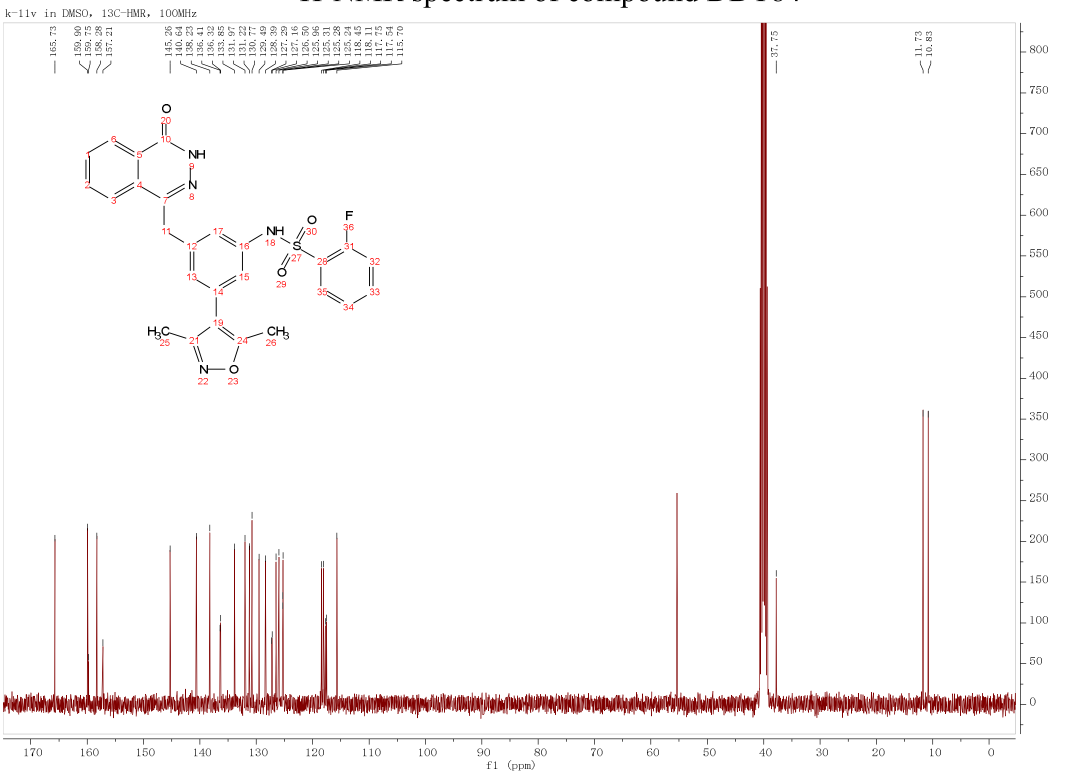

^{13}C-NMR spectrum of compound DDT84

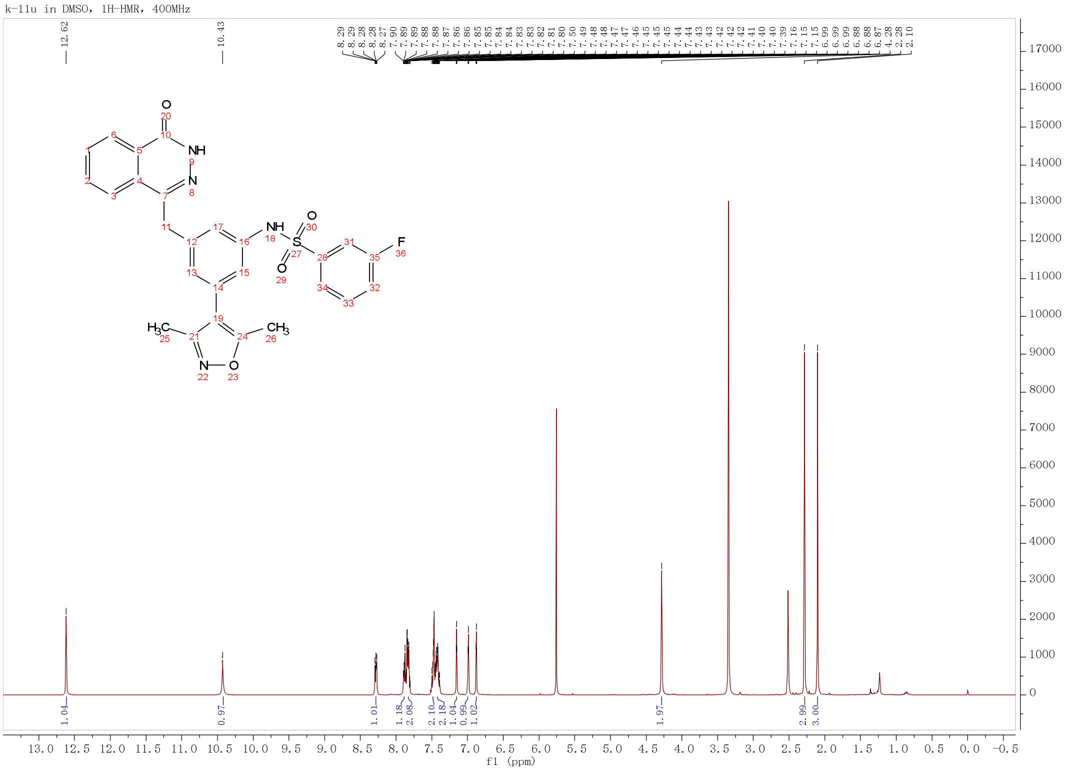

^{1}H-NMR spectrum of compound DDT85

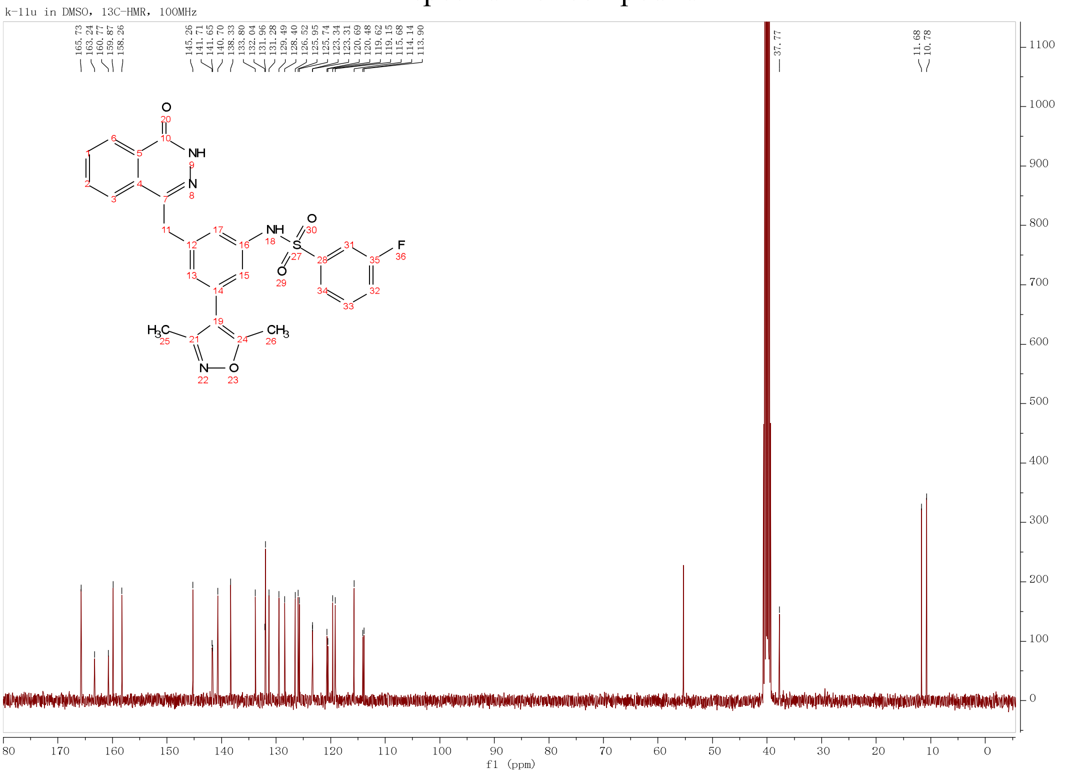

^{13}C-NMR spectrum of compound DDT85

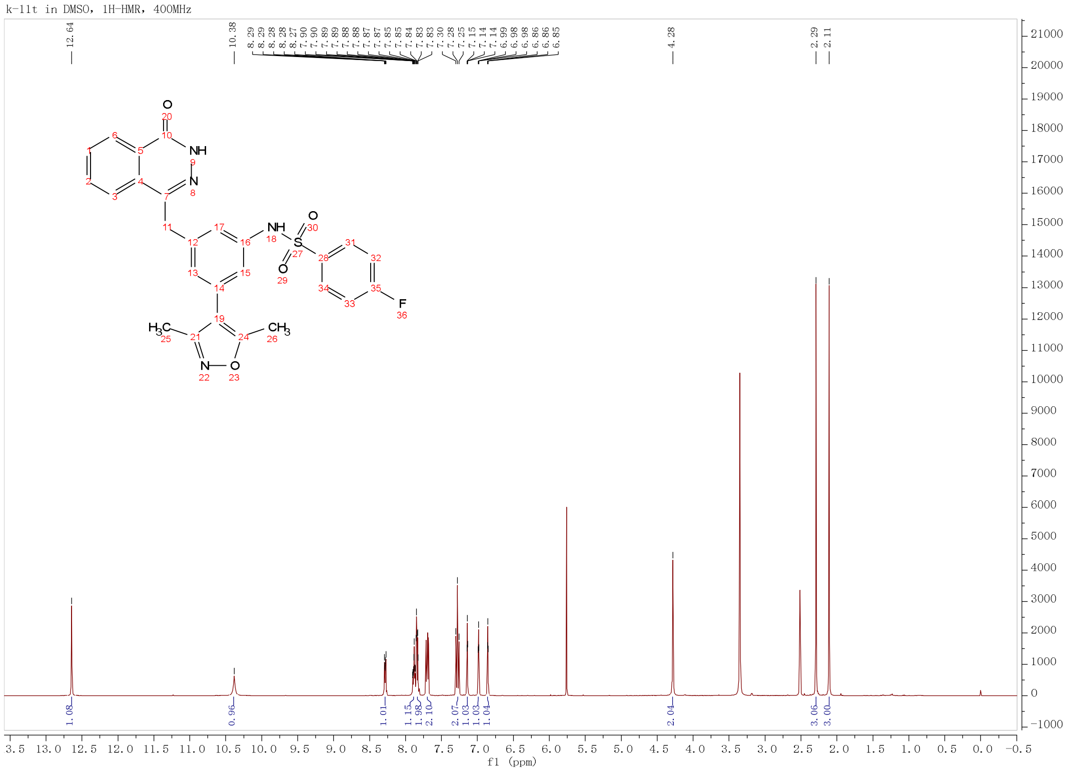

^{1}H-NMR spectrum of compound DDT86

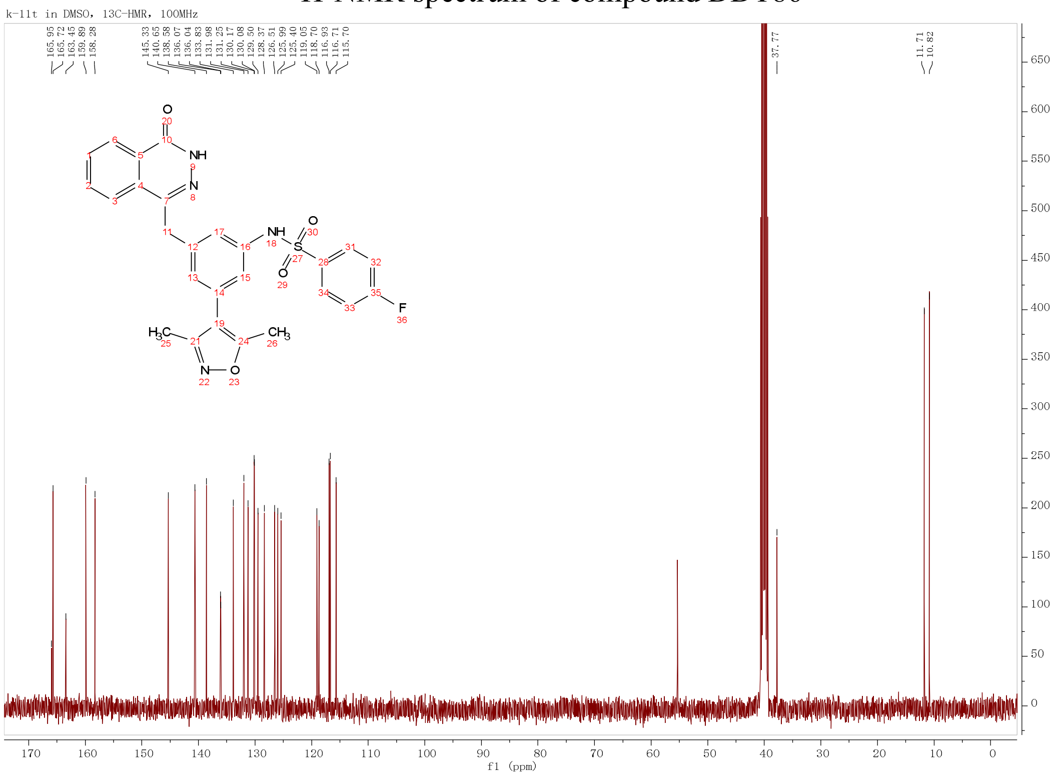

^{13}C-NMR spectrum of compound DDT86

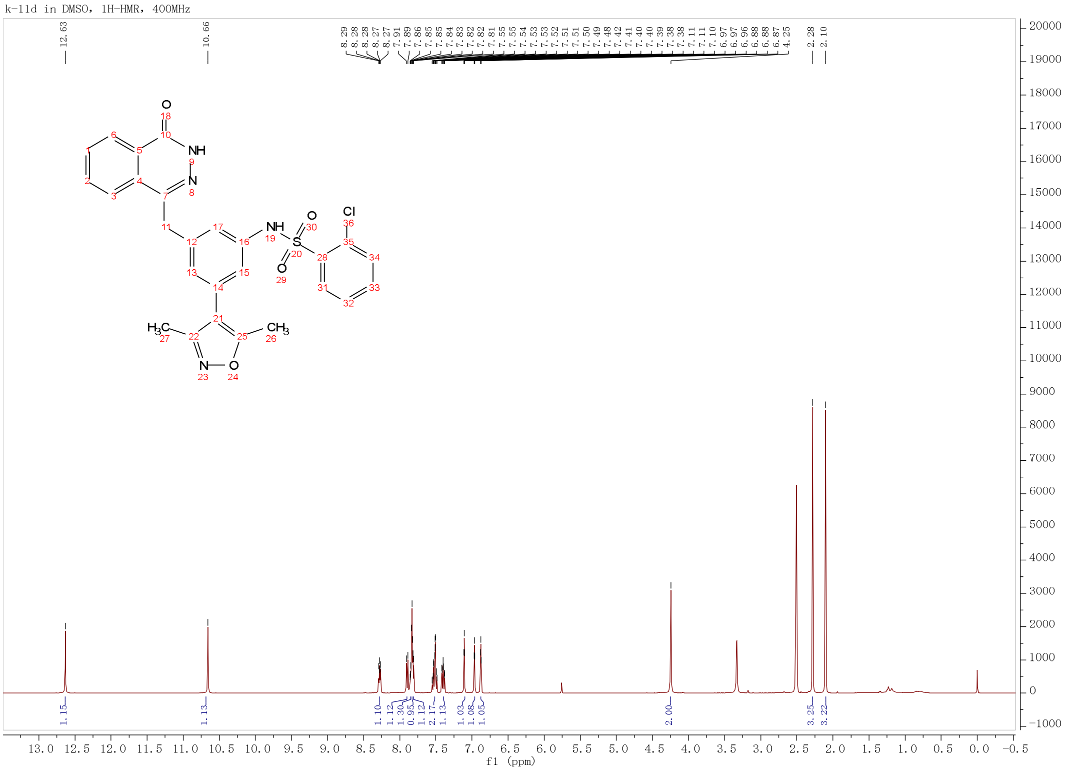

^{1}H-NMR spectrum of compound DDT87

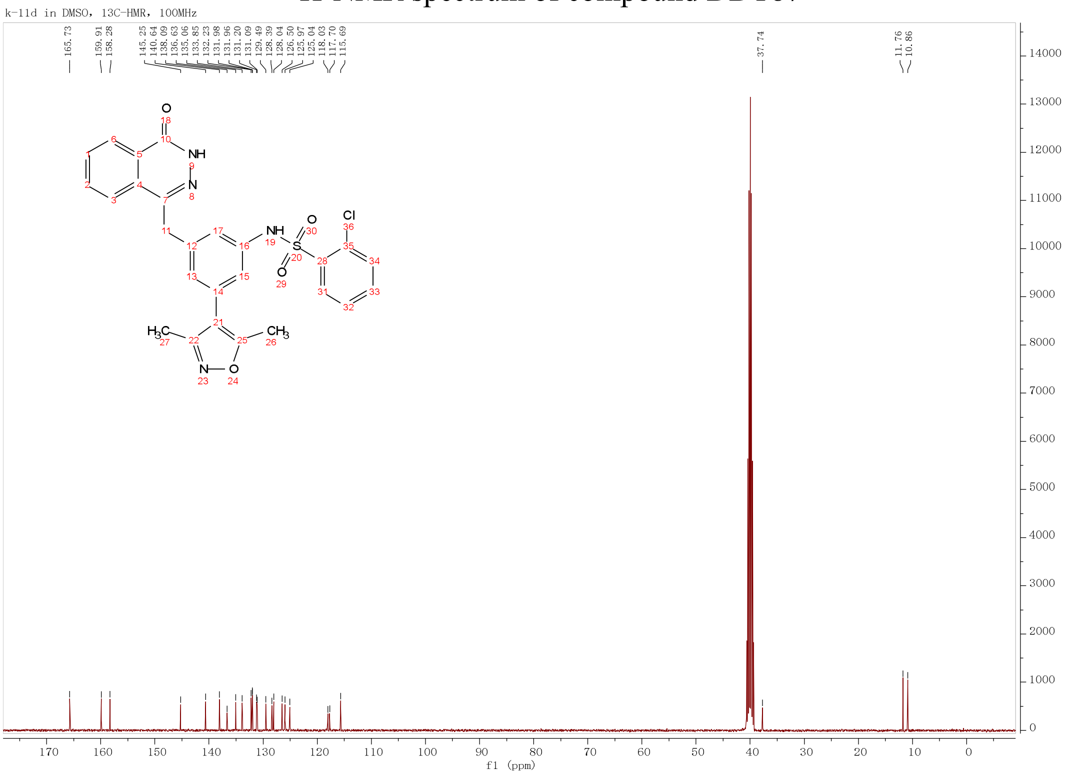

^{13}C-NMR spectrum of compound DDT87

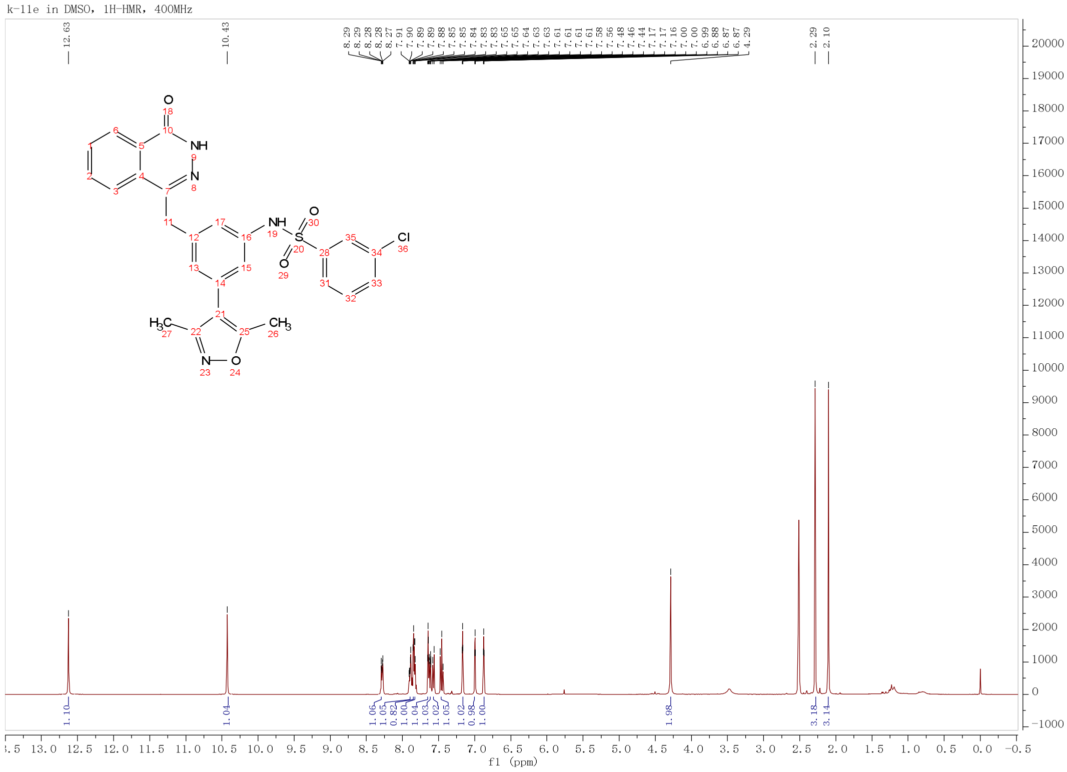

^{1}H-NMR spectrum of compound DDT88

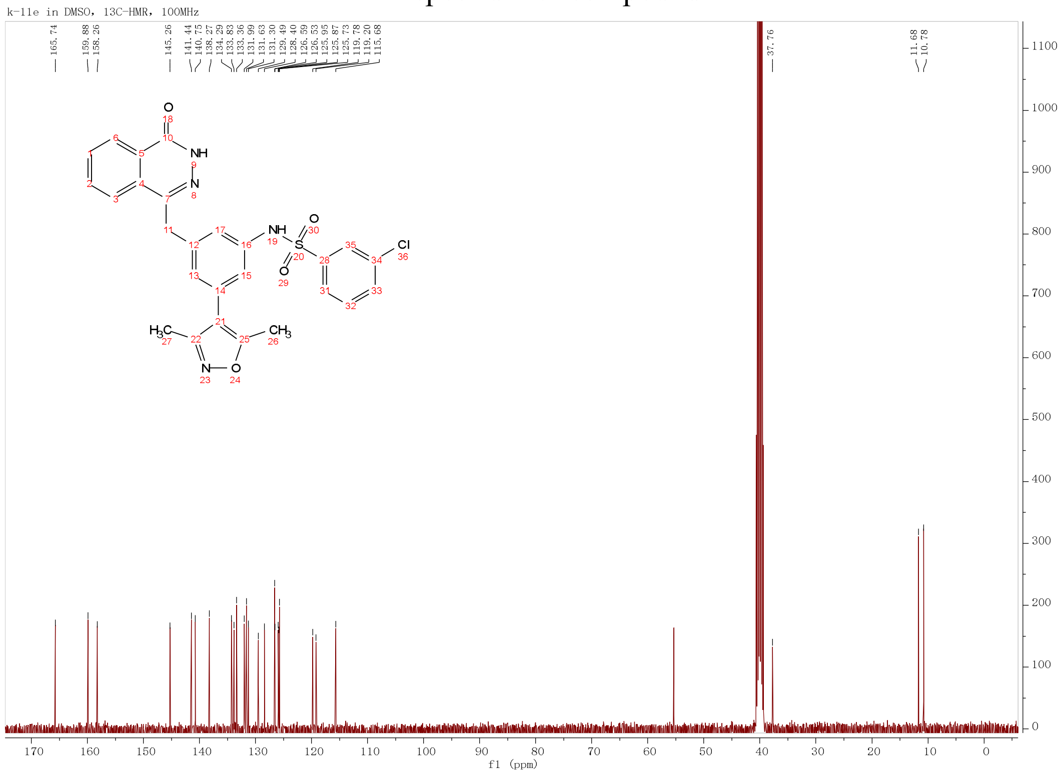

^{13}C-NMR spectrum of compound DDT88

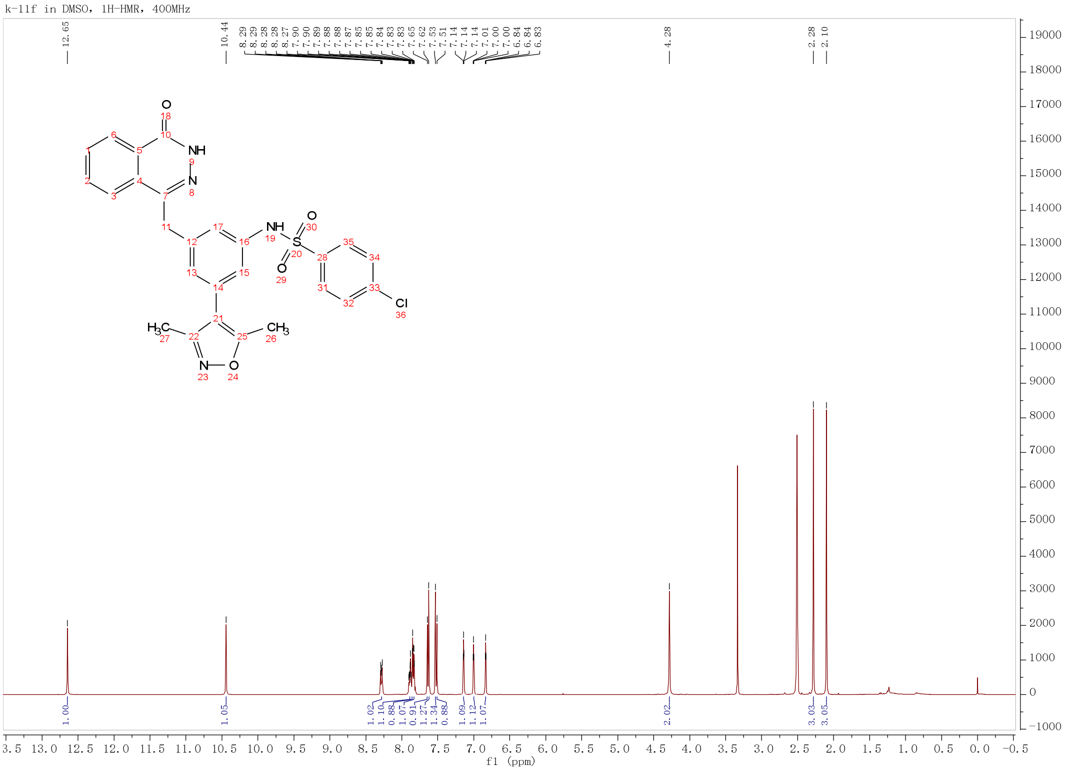

^{1}H-NMR spectrum of compound DDT89

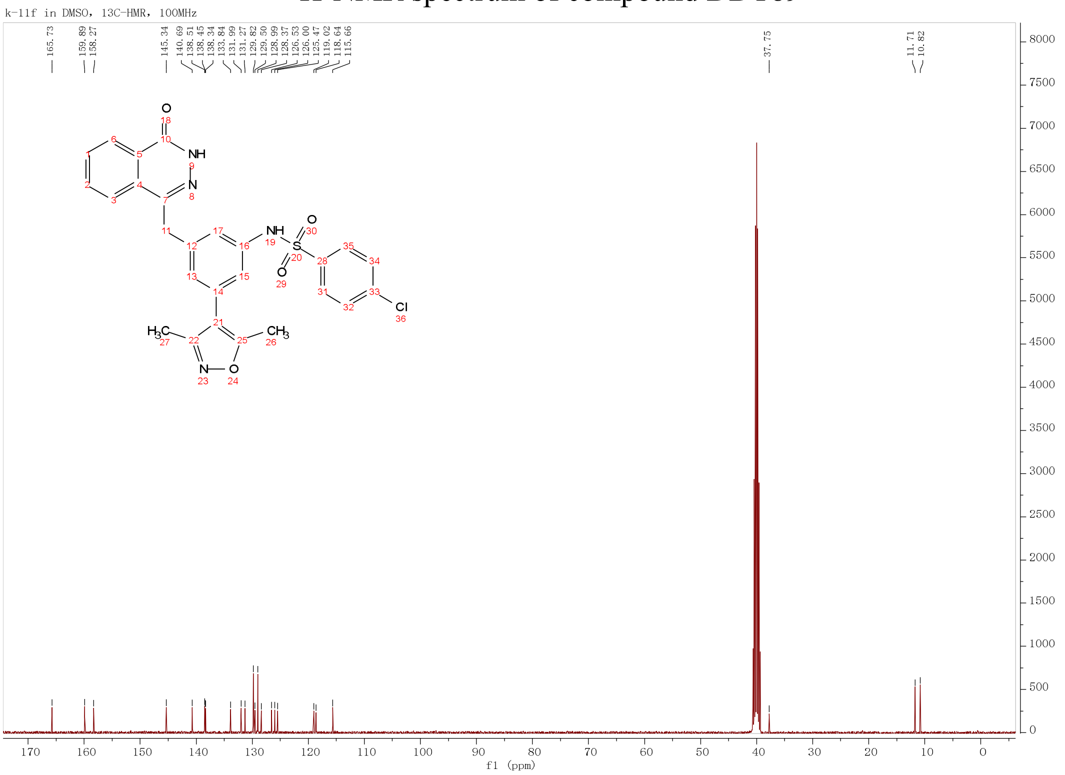

^{13}C-NMR spectrum of compound DDT89

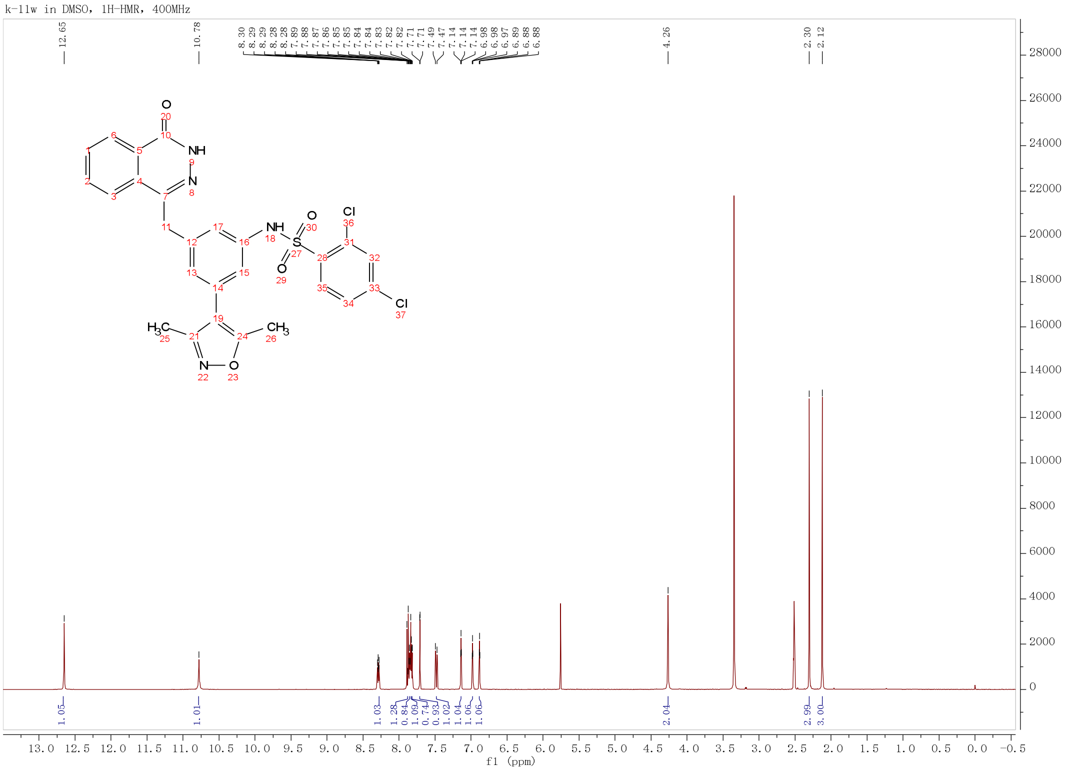

^{1}H-NMR spectrum of compound DDT90

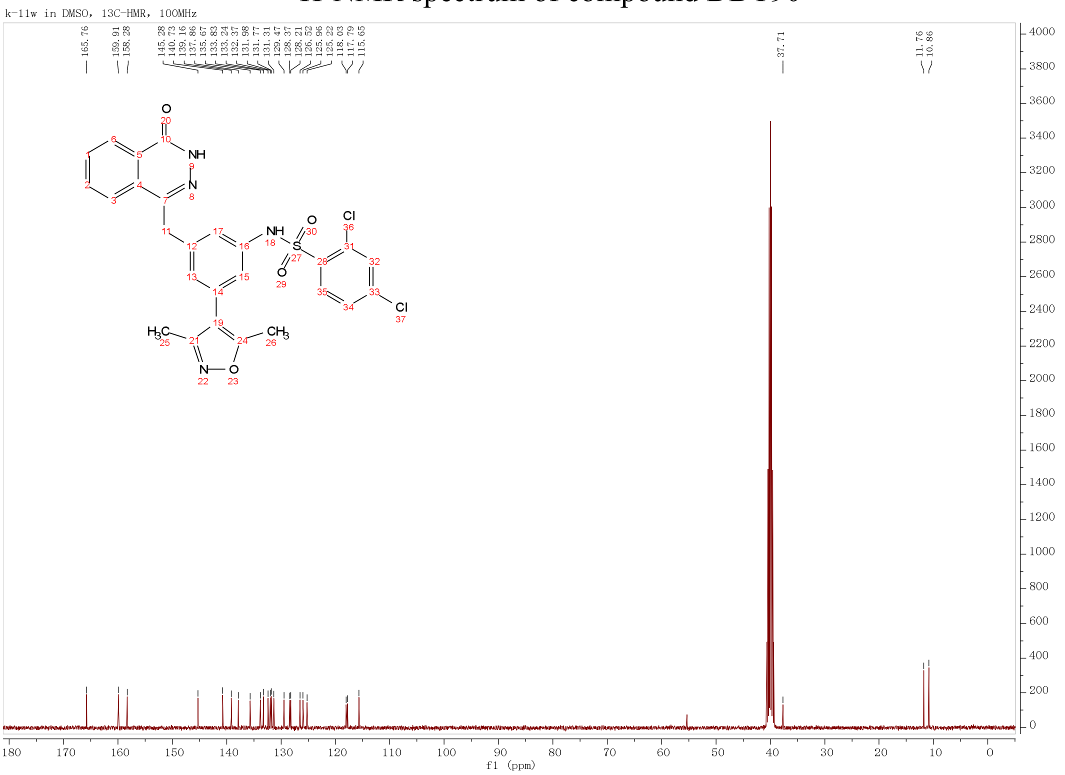

^{13}C-NMR spectrum of compound DDT90

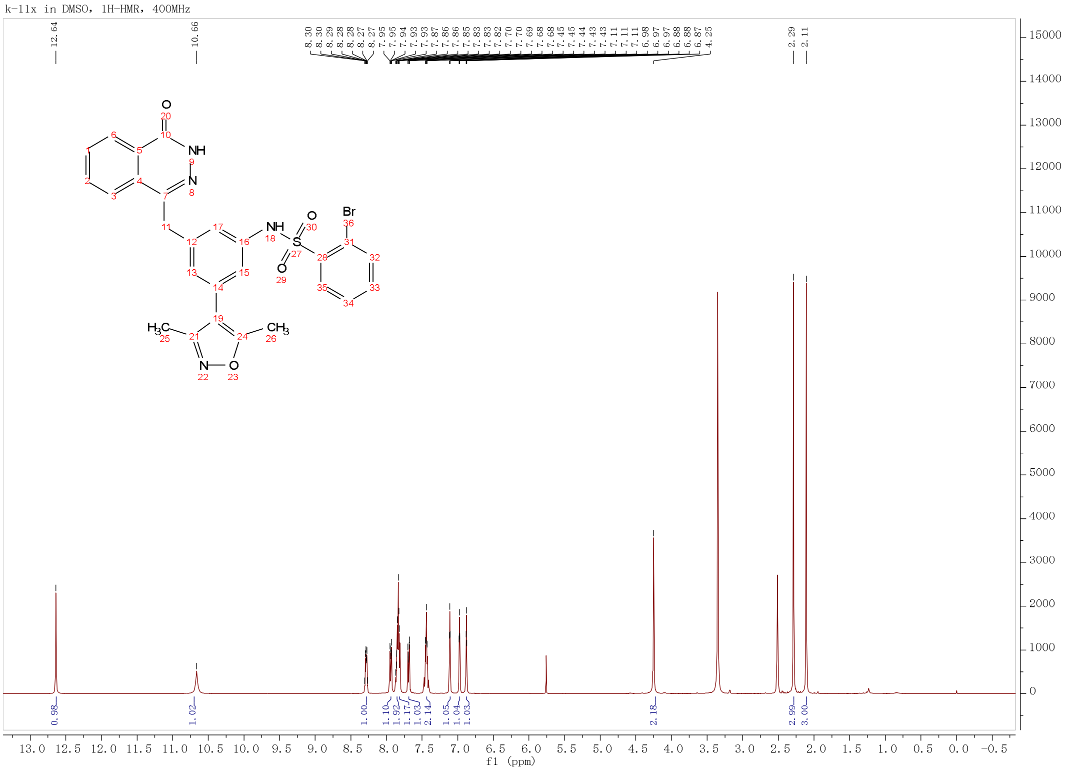

^{1}H-NMR spectrum of compound DDT91

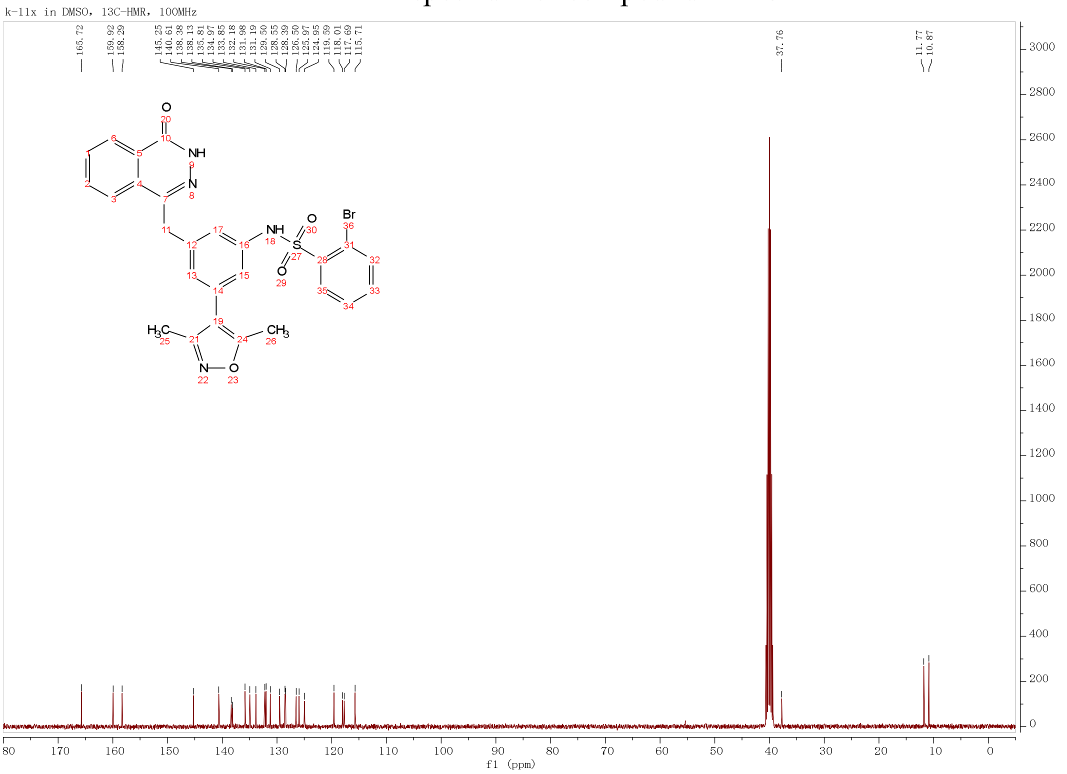

^{13}C-NMR spectrum of compound DDT91

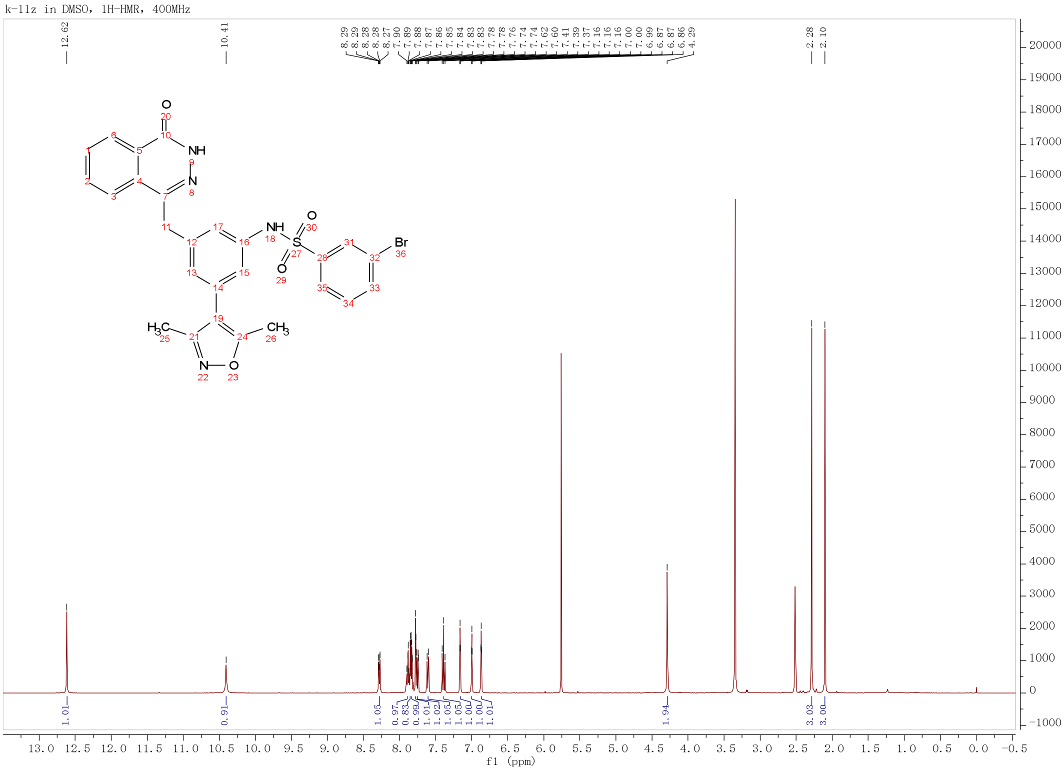

^{1}H-NMR spectrum of compound DDT92

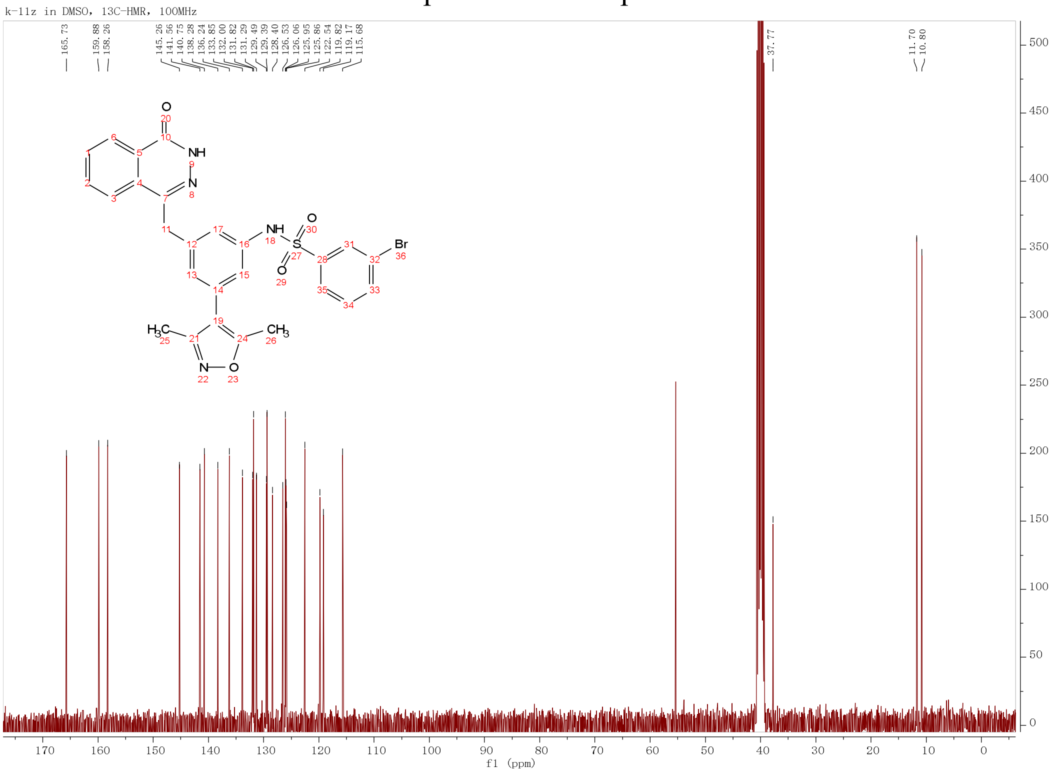

^{13}C-NMR spectrum of compound DDT92

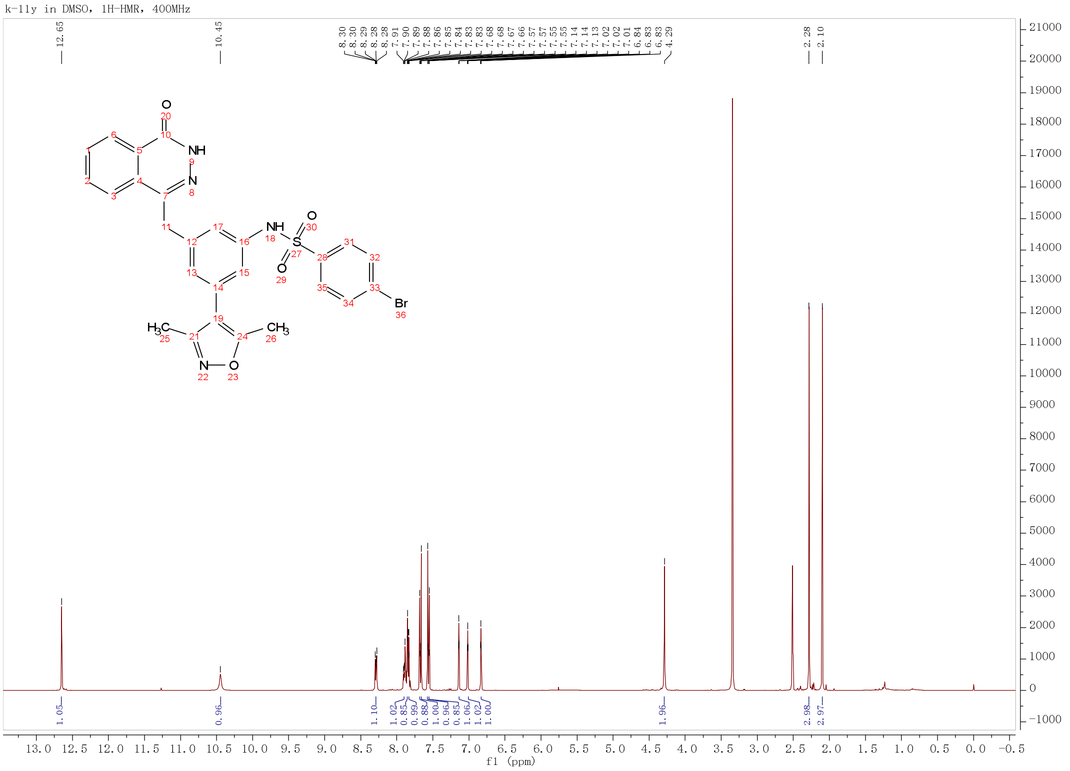

^{1}H-NMR spectrum of compound DDT93

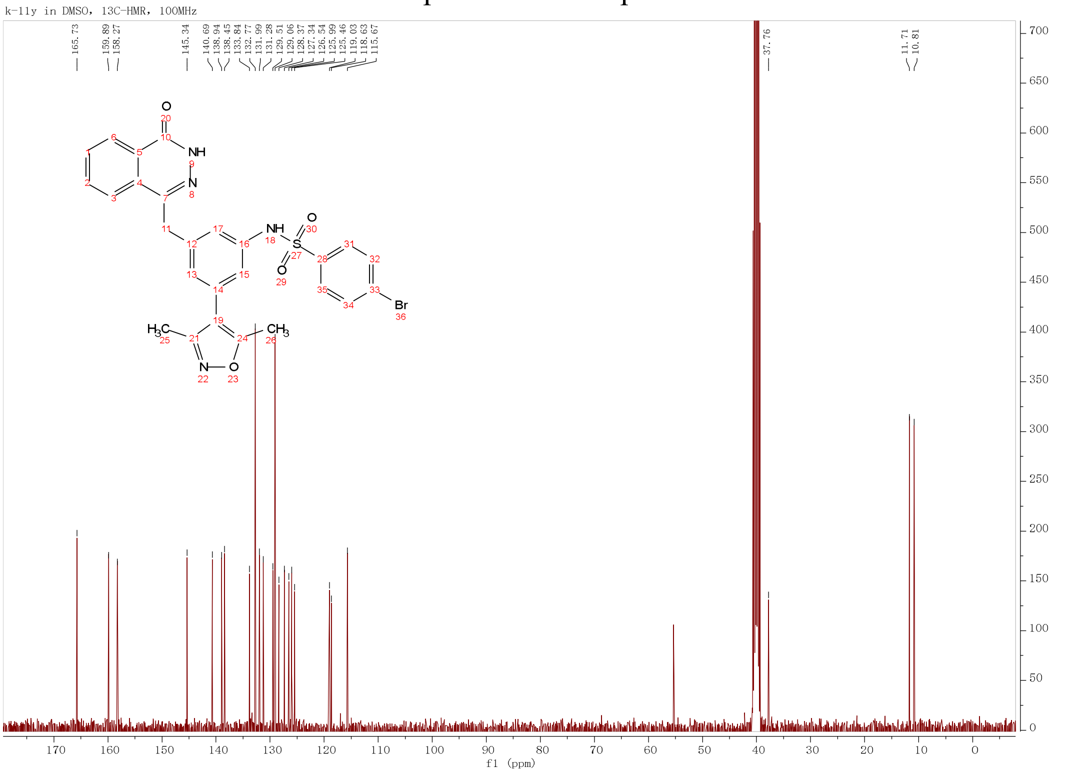

^{13}C-NMR spectrum of compound DDT93

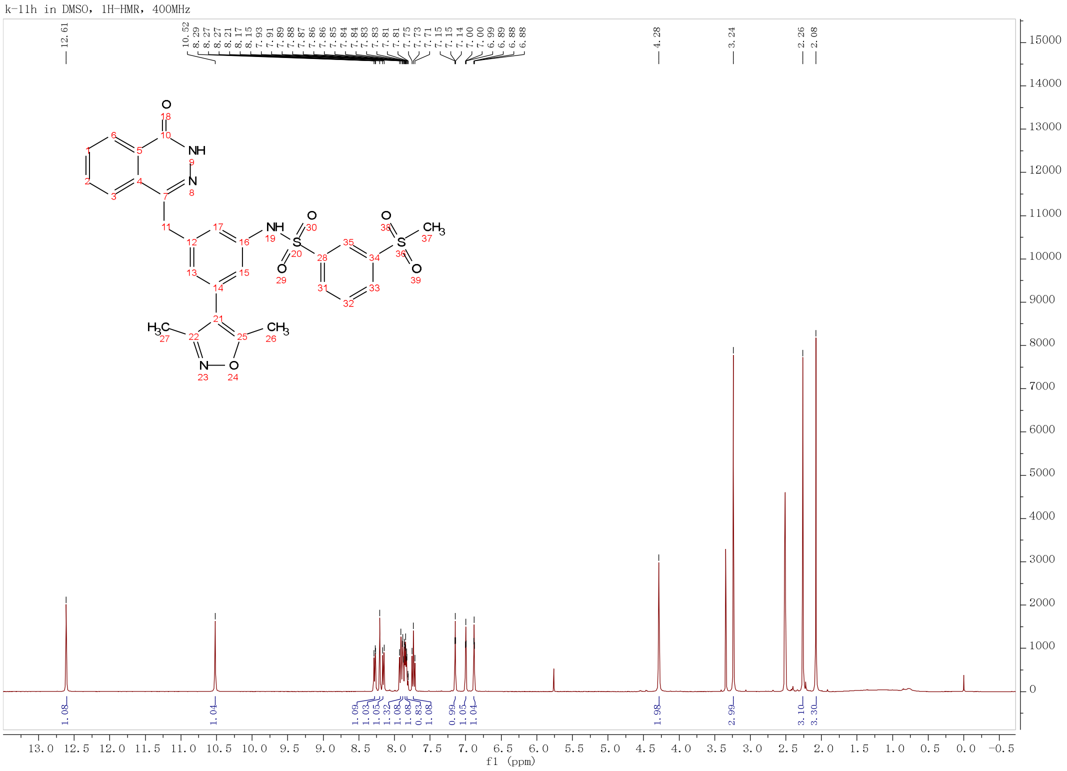

^{1}H-NMR spectrum of compound DDT94

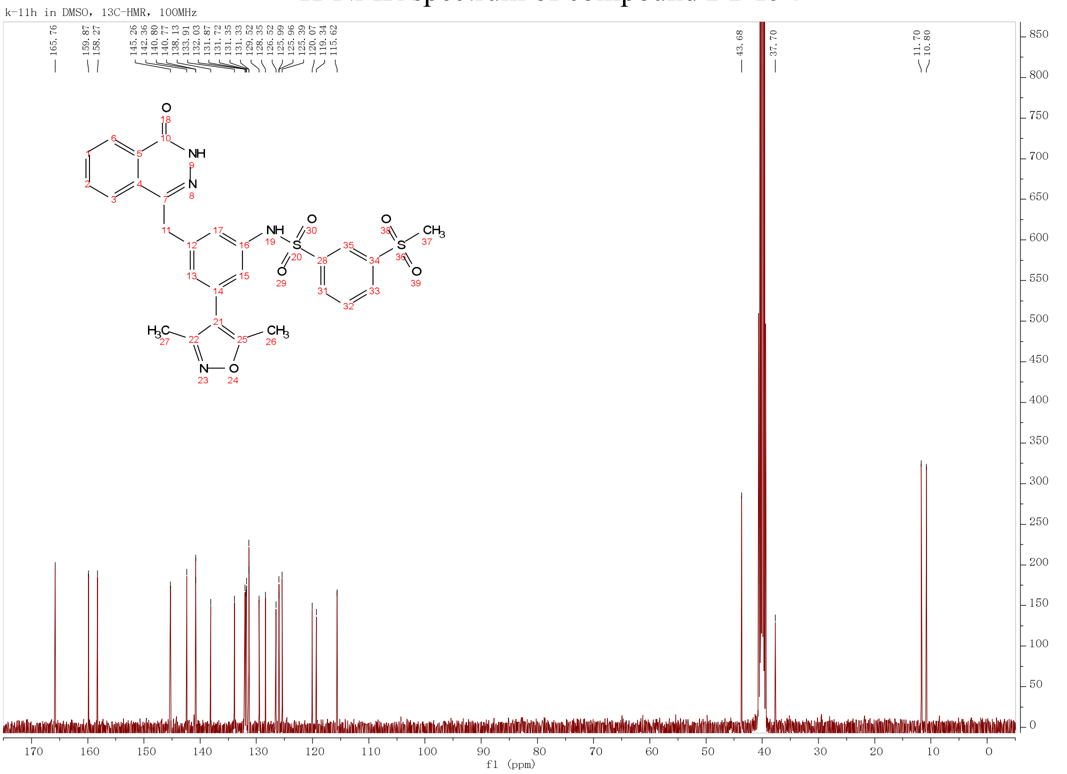

^{13}C-NMR spectrum of compound DDT94
